# General, Modular Access toward Immobilized Chiral Phosphoric Acid Catalysts and Their Application in Flow Chemistry

**DOI:** 10.1021/acscatal.4c00985

**Published:** 2024-03-29

**Authors:** Michael Laue, Maximilian Schneider, Markus Gebauer, Winfried Böhlmann, Roger Gläser, Christoph Schneider

**Affiliations:** † Institute of Organic Chemistry, University of Leipzig, 04103 Leipzig, Germany; ‡ Institute of Chemical Technology, University of Leipzig, 04103 Leipzig, Germany; § Division of Superconductivity and Magnetism, Felix-Bloch Institute for Solid-State Physics, University of Leipzig, 04103 Leipzig, Germany

**Keywords:** organocatalysis, heterogeneous catalysis, immobilization, chiral phosphoric acids, stereoselective synthesis, continuous flow chemistry

## Abstract

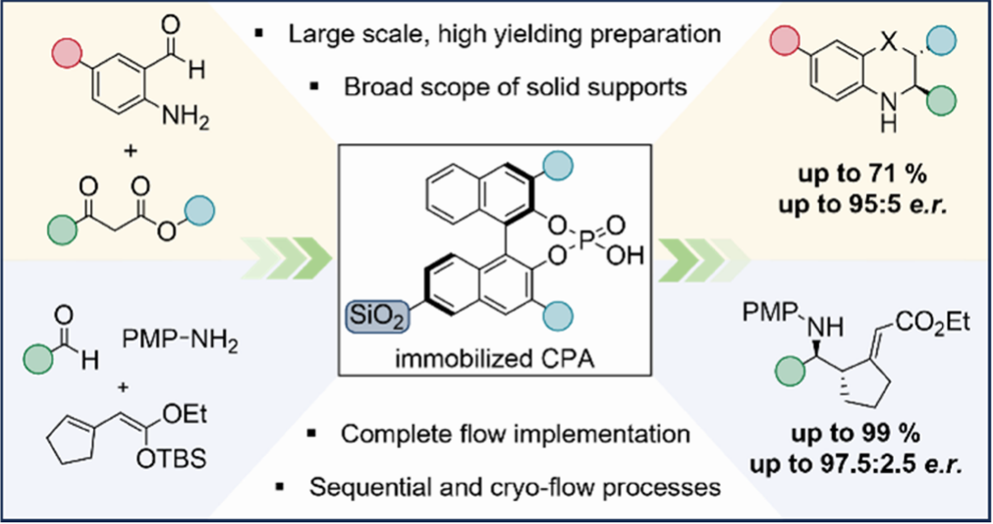

Chiral phosphoric acids (CPAs) are among the most frequently used organocatalysts, with an ever-increasing number of applications. However, these catalysts are only obtained in a multistep synthesis and are poorly recyclable, which significantly deteriorates their environmental and economic performance. We herein report a conceptually different, general strategy for the direct immobilization of CPAs on a broad scope of solid supports including silica, polystyrene, and aluminum oxide. Solid-state catalysts were obtained in high yields and thoroughly characterized with elemental analysis by inductively coupled plasma-optical emission spectrometry (ICP-OES), nitrogen sorption measurements, thermogravimetric analysis, scanning transmission electron microscopy/energy-dispersive X-ray spectroscopy (STEM/EDX) images, and solid-state NMR spectroscopy. Further, the immobilized catalysts were applied to a variety of synthetically valuable, highly stereoselective transformations under batch and flow conditions including transfer hydrogenations, a Friedländer condensation/transfer hydrogenation sequence, and Mannich reactions under cryogenic flow conditions. Generally, high yields and stereoselectivities were observed along with robust catalyst stability and reusability. After being used for 10 runs under batch conditions, no loss of selectivity or catalytic activity was observed. Under continuous-flow conditions, the heterogeneous system was in operation for 19 h and the high enantioselectivity remained unchanged throughout the entire process. We expect our approach to extend the applicability of CPAs to a higher level, with a focus on flow chemistry and a more environmentally friendly and resource-efficient use of these powerful catalysts.

## Introduction

Pioneered by independent studies of Akiyama^1^ and Terada^2^ in 2004, BINOL-derived chiral phosphoric acids (CPAs) established themselves as privileged Brønsted acid organocatalysts. Due to their high efficiency and versatility, a plethora of highly selective asymmetric transformations, e.g., Friedel–Crafts,^3−8^ Mannich,^9−11^ Diels–Alder,^12−14^ and Strecker^15−17^ reactions, was developed over the past two decades. Inherently, such catalysts combine a Brønsted acidic P–OH functionality and a Lewis basic P=O site. By exploiting the resulting cooperative activation mode, a large variety of organic reactions can be promoted under typically mild reaction conditions.^18−20^ However, CPAs are only obtained in complex, multistep syntheses, and in general, relatively high catalyst loadings are required. Combined with their poor recovery, this results in high costs and poor environmental performance, severely limiting their practical applications. Especially with respect to climate change and global energy and resource management, it is highly desirable to overcome these drawbacks by immobilization of CPAs. Transitioning to heterogeneous catalysis paves the road to broaden the use of CPAs because immobilized catalysts feature advantages such as facile separation, high recyclability, simple product isolation, and the possibility of an implementation into continuous-flow reactors.^21−23^

In contrast to conventional batch applications, flow systems generally benefit from enhanced heat- and mass transfer, improved safety, higher sustainability, and broad scalability.^24−30^ Moreover, flow reactions employing solid-state catalysts are perfectly suited to meet the high standards of recent “green sustainable chemistry” concepts.^31−33^

Although heterogeneous catalysis has played a crucial role in the chemical industry for decades, the impact of solid-state organocatalysts on the stereoselective synthesis of chiral products is often overlooked.^34,35^ In recent years, however, the field of heterogeneous organocatalysis has expanded considerably.

In 2010, Rueping et al. reported the immobilization of a polystyrene-supported CPA by cross-linking radical copolymerization, which was successfully used in an asymmetric transfer hydrogenation.^36^ One year later, Blechert and co-workers built up on this work and introduced 3-(anthracen-9-yl)-thiophene moieties in the 3,3′-positions of the BINOL backbone in order to obtain a microporous CPA by oxidative coupling under FeCl_3_ catalysis.^37^ The Pericas group has accomplished significant contributions in the whole field of immobilized catalysts for stereoselective transformations in batch and flow.^38^ In 2014, they prepared a solid CPA-based catalyst by immobilization of a BINOL-derivative on polystyrene, which was subsequently converted to the corresponding phosphoric acid over two steps in the presence of the support material. The catalyst was applied in a Friedel–Crafts reaction of indoles under batch and flow conditions and showed remarkable results in terms of activity, selectivity, and stability.^39^

An efficient strategy for the immobilization of the TRIP-CPA catalyst onto polystyrene and its use for batch and flow applications was also developed by the same group.^40^ This polymer-supported TRIP catalyst was successfully employed in different highly stereoselective reactions including allylation reactions of aldehydes^40^ and, very recently, a Pictet-Spengler cyclization.^41^ Further, Pericàs et al. could also show the preparation and application of SPINOL-derived CPAs immobilized also onto polystyrene.^42^

In 2022, the You group reported another copolymerization approach using external cross-linkers and applied their catalysts in an asymmetric transfer hydrogenation and dearomatization of β-naphthols, however, under batch conditions only.^43^

**Figure 1:**
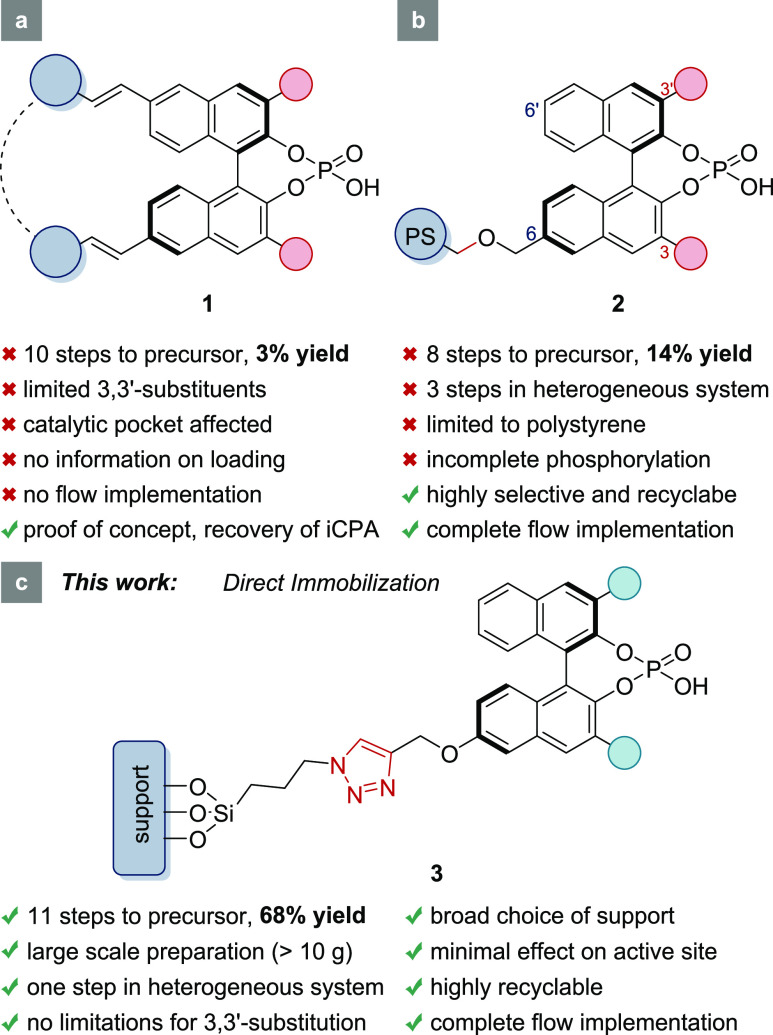
Comparison of the conceptually different methods for the synthesis of supported CPAs. (a) Copolymerization approach by Rueping^36^, (b) immobilization of protected BINOLs by Pericàs,^39^ and (c) our new strategy. Bonds highlighted in red are formed for the fixation of the catalyst.

Conceptually, the reported immobilization methods can be divided into two different classes: (i) a copolymerization-like approach and (ii) the immobilization of protected BINOLs onto a solid support and subsequent introduction of the phosphoric acid on these modularly constructed immobilized diols. All these methods inevitably suffer from limitations regarding 3,3′-substituents, low-yielding precursor syntheses, difficult reaction monitoring during catalyst synthesis, undesirable swelling properties, and most importantly, their limitation to polystyrene as support material. A comparison as shown in Figure 1 clearly highlights the significant drawbacks of the known strategies. On the other hand, a direct immobilization of molecular CPAs is currently unprecedented but would allow for the preparation of immobilized chiral phosphoric acids (iCPAs) without all these limitations. We therefore envisioned to develop a conceptually novel strategy for the direct attachment of CPAs onto variable support materials that is completely modular, high yielding, and robust. Further, the catalysts should demonstrate their potential under both batch and flow conditions.

## Results and Discussion

### Synthesis of Immobilized CPAs (iCPAs)

In order to avoid any perturbation of the catalytically active site, the CPAs were immobilized at a remote position, namely, the 6-position of the BINOL backbone.^39^

**Figure 2:**
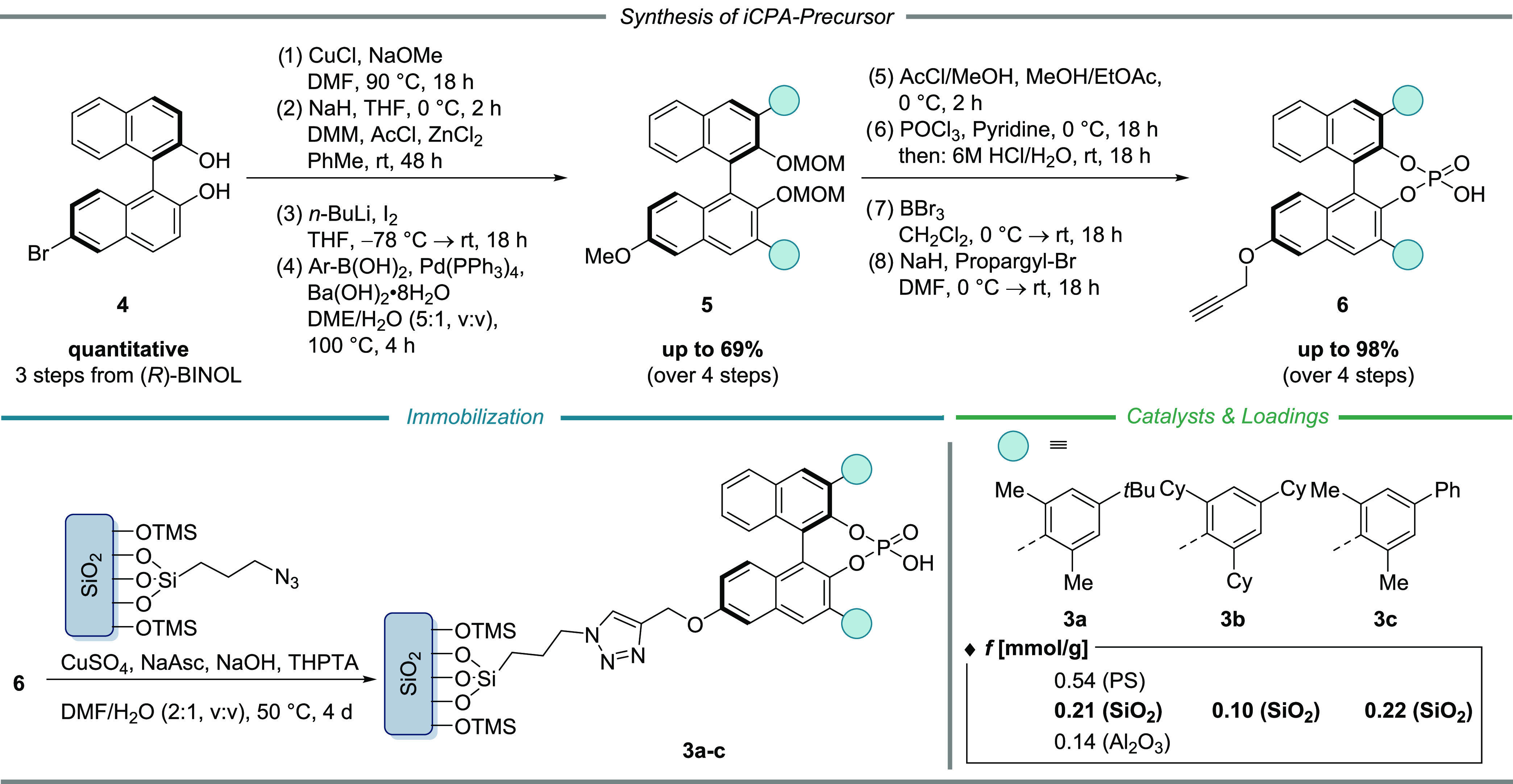
Schematic overview of the synthesis of immobilized chiral phosphoric acids according to our new route.

Starting from commercially available, enantiomerically pure (*R*)-BINOL, the (*R*)-6-bromo-[1,1′-binaphthalene]-2,2′-diol, was synthesized on large scale and in quantitative yield over three steps by a reported procedure.^44^ We reasoned that the modification of the BINOL-scaffold should be as minimal as possible, and therefore, (*R*)-6-bromo-BINOL **4** was converted to the methoxy-derivative in an Ullmann-type reaction (Figure 2). After MOM protection of the BINOL hydroxy groups, subsequent *ortho*-iodination, and cross-coupling, a suitable precursor **5** was obtained up to 69% yield over four steps, with the iodination step as the bottleneck.

Different cross-coupling reactions could be employed to introduce all common 3,3′-substituents. In our hands, a Suzuki reaction and a Kumada cross-coupling worked best. Next, the MOM-protecting groups were selectively cleaved off in quantitative yield, and then, the phosphoric acid was installed. For the synthesis of silica-supported catalysts, the phosphoric acid moiety must be introduced prior to immobilization to avoid an undesired achiral phosphorylation of the silica surface, resulting in a racemic background reaction when used as a catalyst.

A key step of our new strategy is the deprotection of the 6-methoxy group. Ishihara et al. reported a boron tribromide-assisted CPA catalyst.^45^ Thus, it was assumed that chiral phosphoric acids are compatible with BBr_3_ deprotection. Indeed, 6-hydroxy CPA was obtained in quantitative yield and further converted to propargyl ether **6**. The overall yield for the complete sequence was exceptionally high, giving rise to the immobilizable CPA **6** in 68% over 11 steps.

In contrast to known procedures,^36,39^ the complete synthesis of the CPA proceeded under homogeneous conditions and was easily monitored by standard analytical methods. Functionalized CPA **6** represents the first catalytically active phosphoric acid that can be directly immobilized on a solid support. This, in turn, highlights one of the main advantages of this strategy: the catalytic performance before and after immobilization is directly comparable. Therefore, the influence of the backbone modification and solid support can be evaluated separately for the first time.

CPA **6** was then directly immobilized via an alkyne–azide click reaction under copper(I) catalysis (CuAAC). All solid support materials were pretreated (see Supporting Information) and functionalized with an azide-linker-unit according to reported procedures.^46^

Optimal conditions for the click reaction employed CuSO_4_ as a precatalyst under aqueous conditions, NaOH as a base, and the THPTA ligand. Sodium ascorbate was added to generate the Cu(I)-species in situ.^47,48^ Even though click chemistry has been applied as a late-stage immobilization method for various organocatalysts,^49^ including the BINOL-skeleton,^50^ it has not yet been applied to CPAs directly because the corresponding precursors (e.g., CPA **6**) were not accessible. Conveniently, unreacted CPA **6** could be completely recovered from the solution after separation of the solid catalyst by filtration, preventing any loss of the precious chiral catalyst. Three differently substituted iCPAs were synthesized with excellent loadings of up to 0.22 mmol·g^–1^. All common 3,3′- moieties can be installed, and further, the solid support can be freely chosen. The latter was highlighted by employing our procedure to polystyrene (PS) and aluminum oxide supports, as well. Both support materials were readily functionalized, yielding the corresponding iCPAs with loadings of 0.54 mmol·g^–1^ (PS) and 0.10 mmol·g^–1^ (Al_2_O_3_), respectively. Differences in loadings are based on the azide loading of the support. The immobilization procedure that was optimized for silica could be applied directly to aluminum oxide without further modifications. Polystyrene, however, had to be treated differently due to its swelling properties. In order to efficiently functionalize the polymer, it must swell properly in the solvent. Therefore, we employed a strategy that was originally used for the immobilization of a MacMillan organocatalyst.^51^ In a mixture of DMF/THF with DIPEA as a base and catalytic amounts of CuI, the polystyrene support was easily functionalized, whereas the click reaction under aqueous conditions was unsuccessful. This observation during catalyst synthesis already implies a drawback of polystyrene-based iCPAs: the solvent-depending swelling properties can affect the accessibility of the catalytically active sites and thus the performance of the catalyst. Swelling is also accompanied by significant changes in the material volume, which led to overpressure and reactor blocking during preliminary flow experiments (especially solvent screening in flow). For these reasons, we focused our studies on silica supports that do not suffer from such behavior.

In summary, a novel strategy for the direct immobilization of CPAs was developed that distinguishes itself conceptually from known methods. Extraordinarily high yields for the precursor synthesis were achieved as well as broad flexibility with respect to the support material and CPA substitution pattern.

### Characterization

The properties of the iCPA catalysts were then investigated thoroughly. The catalyst loading was determined with high precision by three different techniques, with elemental analysis by inductively coupled plasma-optical emission spectrometry (ICP-OES) being the main analytical method.

Since the formation of the phosphoric acid occurred in the absence of the solid support, it can be safely assumed that only one phosphorus species is present on the surface of the material. Therefore, the phosphorus content derived from ICP-OES directly correlates with the CPA loading. All results were further validated by thermogravimetric and elemental analysis. Figure 2 shows the average loading for all three catalysts.

Nitrogen sorption was performed to characterize the textural properties of the material throughout the immobilization. The average pore width of ca. 100 Å was preserved until CPA was introduced. After immobilization, the pore width was reduced to ca. 35 Å, which is in accordance with the expected spatial requirement of the CPA (deduced from density functional theory (DFT) optimized molecular structures, see SI). Moreover, the mesoporosity of the material remained intact throughout the process.

**Figure 3:**
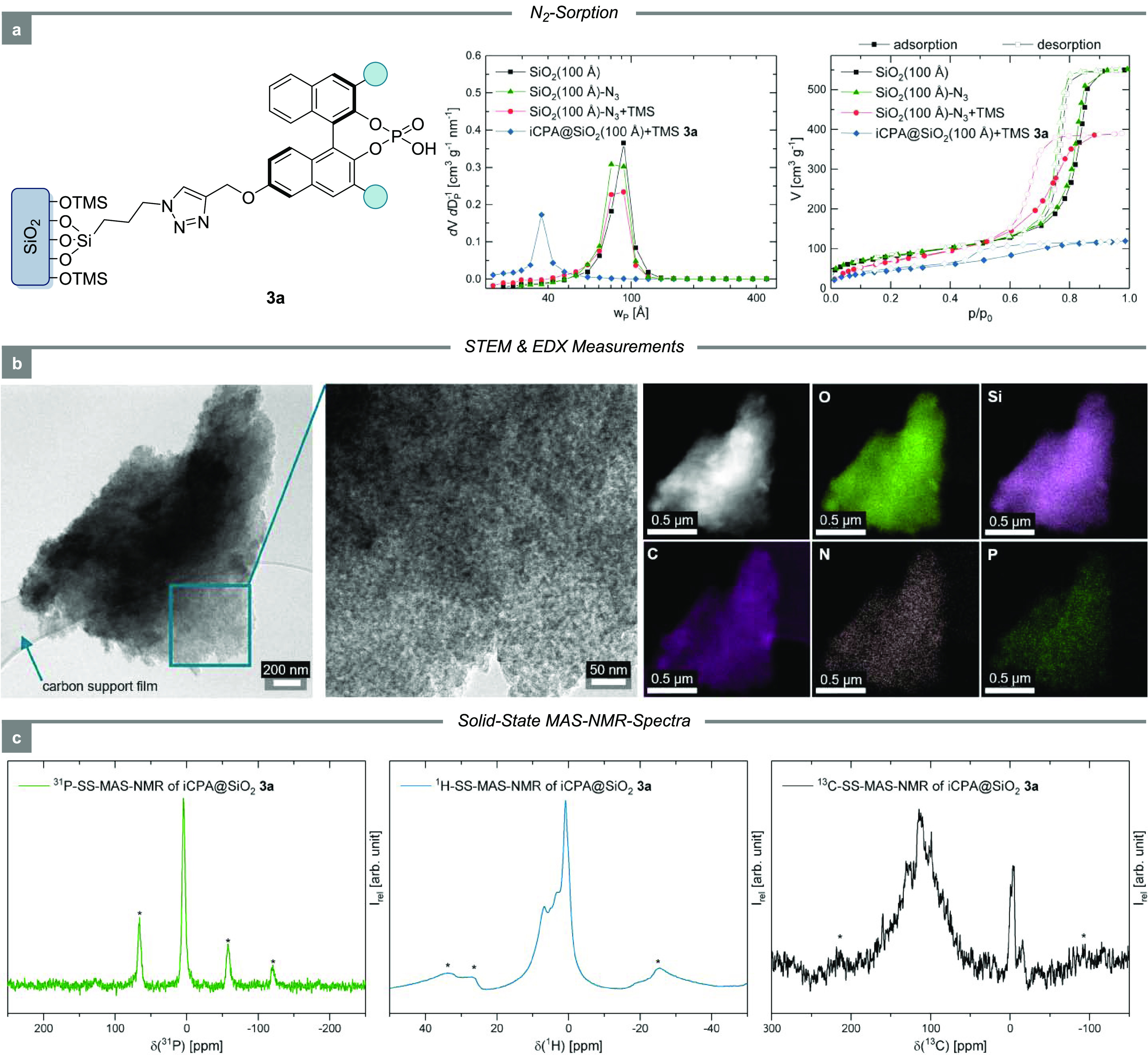
Analysis of the solid-state catalyst **3a**. (a) Nitrogen sorption isotherms and pore width distribution for the iCPA **3a** and the support material. (b) STEM/EDX images of **3a** with the elemental distribution of O, Si, C, N, and P. (c) Solid-state-NMR of **3a** (rotational side bands are marked with an asterisk).

According to the scanning transmission electron microscopy/energy-dispersive X-ray spectroscopy (STEM/EDX) images (Figure 3b) of iCPA **3a**, the morphology was demonstrated to be very homogeneous. Material contrast imaging for O, Si, C, N, and P also revealed a homogeneous elemental distribution.

Finally, solid-state-MAS NMR spectra on various nuclei (^31^P, ^1^H, ^13^C, ^29^Si, and ^27^Al) were recorded, proving a truly covalent immobilization of the CPAs.^52^ A representative ^31^P NMR spectrum of iCPA **3a** depicted in Figure 3c shows a single peak at 4.29 ppm. To our delight, this observation gives clear evidence that only one phosphorus species is tightly anchored to the solid support, and further, the chemical shift is in excellent agreement with the data of the molecular precursor. Moreover, ^1^H and ^13^C NMR spectra confirmed the successful immobilization process (see SI for further detail). NMR spectra of the iCPAs immobilized onto PS and aluminum oxide (see SI) show similar results with single ^31^P signals in the expected range of chemical shift (3.73 ppm (PS) and 3.67 ppm (Al_2_O_3_), respectively).

### Transfer Hydrogenation of 2-Substituted Quinolines

We first applied our iCPAs to an asymmetric transfer hydrogenation (TH) of 2-substituted quinoline derivatives **7** as published by Rueping et al.^53^

#### Batch Conditions and Recyclability

Prior to any flow experiments, the reaction was optimized under batch conditions (Table S1). A molecular version of catalyst **3a** in chloroform was found to be optimal for this reaction. To our delight, the reaction proceeded smoothly at 60 °C giving rise to an excellent *e.r*. of >99:1 (S1, entry 1) at full conversion after only 0.5 h. With the use of co-catalytic amounts of HOAc as described by Antilla,^54^ the reaction temperature could be decreased to room temperature and the tetrahydroquinoline **8a** was isolated with 92% yield and 99:1 *e.r*. (S1, entry 2).

To evaluate the influence of the linker, molecular CPA **SI-3b** was also tested under the same conditions and gave full conversion with a slightly diminished *e.r*. of 98:2. The same results were obtained by using iCPA **3a** (S1, entries 3 and 4). It can therefore be safely concluded that the immobilization of the catalyst itself does not interfere with an effective stereoinduction.

Our initial concern was that the basic triazole unit could affect the catalytic performance of the iCPA. However, we did not observe any significant deactivation issues in our test reactions.

**Figure 4:**
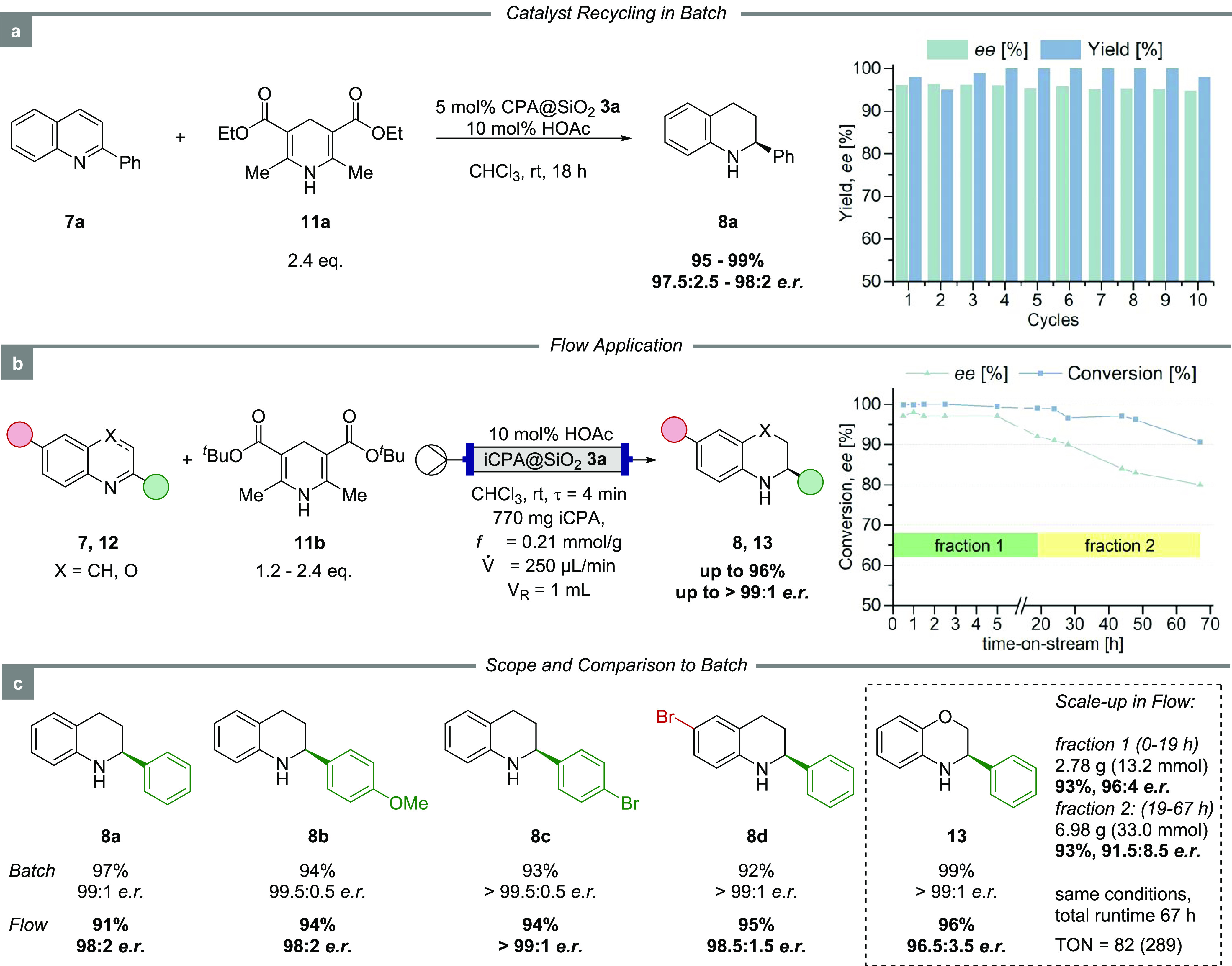
Stereoselective transfer hydrogenation of 2-substituted quinolines with an iCPA under batch and flow conditions; TON in parentheses refers to the complete process (67 h). Conversion over time of the flow process was determined by HPLC. Optimized flow conditions: c(**7,12**) = 0.05 mol·L^–1^, *V̇* = 250 μL·min^–1^ (τ = 4 min), rt, 10 mol % HOAc and *t*Bu based dihydropyridine **11b**. The absolute configuration of all products was assigned by comparison of optical rotation to the literature.^53^

Once the optimal catalytic system was identified, one of the main objectives of this work was addressed, namely, the reusability of the iCPAs. In this respect, the silica-supported catalyst **3a** was recycled ten times, giving consistently excellent results in terms of yield and enantioselectivity (Figure 4a). It is important to mention that no reactivation of the catalyst through an acidic wash between the recycling cycles was necessary. Thus, the immobilized catalyst was proven to be robust and highly recyclable. Using immobilized catalysts for this transformation saves considerable resources in the form of the precious CPA catalyst and, therefore, drastically improves both the environmental and economic performance of the catalytic reaction.

#### Application of Additional Solid Supports

Further, we envisioned the newly developed method to be applicable to a broad range of support materials. To confirm this hypothesis, the influence of the solid support on the catalytic activity was investigated (S1, entries 5 and 6). Both the aluminum oxide- and polystyrene-supported iCPAs **9** and **10** catalyzed the transfer hydrogenation of **7a** with the same yield and enantioselectivity as the silica-supported iCPA **3a** (>98%, 98:2 *e.r*.). Consequently, this strategy is fully applicable to all three support materials, which in turn allows for an accurate adjustment of the solid support to the specific needs of any given transformation.

In order to conduct the reaction under continuous-flow conditions, *t*Bu-substituted Hantzsch ester **11b** was used instead of the ethyl ester-based derivative. This modification not only resulted in an improved solubility and shorter reaction times but also gave rise to even superior results of 97% isolated yield and >99:1 *e.r*. (S1, entry 7).

#### Flow Conditions

With the optimized batch reaction conditions established, the process was transferred to flow conditions (Table S2). In the continuous-flow setup, a commercially available HPLC column (ø 4 mm, length 100 mm) was employed as a fixed-bed reactor and packed with 770 mg of iCPA **3a** (*f* = 0.21 mmol·g^–1^). Initially, a solution of quinoline and dihydropyridine was pumped through the reactor at a total flow rate of 100 μL·min^–1^ and 70 °C. Pleasingly, full conversion and an enantiomeric ratio of 96.5:3.5 were obtained. Nevertheless, *e.r*. decreased over time. This is likely the result of catalyst inhibition by the pyridine byproduct, deteriorating the catalyst performance. However, the addition of 10 mol % HOAc solved this issue.

After extensive optimization (SI), the product was obtained in 91% yield with an *e.r*. of 98:2 within only 4 min residence time (at 250 μL·min^–1^), highlighting the dramatic acceleration of this reaction with the new iCPA under continuous-flow conditions (Figure 4b). Interestingly, lowering the flow rate led to decreased enantioselectivities in this case, which is most likely attributed to less effective mixing.

With the optimized batch and flow conditions in hand, we next investigated the substrate scope. Variation of the aryl component in the 2-position of the quinolines was easily possible as demonstrated by introducing electron-neutral (Ph), as well as electron-donating (PMP) and -withdrawing (*p*-Br-Ph) substituents. All examples gave rise to the corresponding tetrahydroquinolines in over 90% yield and excellent enantioselectivity of 98:2 → 99:1 *e.r*. both in batch and flow experiments. In addition, 1,4-benzoxazine **12** performed likewise in this process with yields >90%, an *e.r*. of >99:1 (batch) and 96.5:3.5 (flow).

To highlight the potential of this process for a large-scale synthesis, the reaction was conducted for a 26-fold extended time-on-stream. The system was operated in continuous-flow for 19 h, and the enantioselectivity remained excellent throughout the process with complete conversion. Starting with 14.2 mmol of **12**, the highly enantioenriched dihydro-1,4-benzoxazine **13** was obtained with an overall yield of 93% (corresponding to 2.78 g) and 96:4 *e.r*. This translates into a remarkable turnover number (TON) of 82 and a productivity of 4.5 mmol(product)·mmol(Cat.)^−1^·h^–1^. In other words, the catalyst loading of the global process was as low as 1 mol %, representing a 5-fold decrease with respect to the batch conditions. We then attempted to push our system to its limits by further extending the time-on-stream to 67 h. After 19 h, the enantiomeric ratio started to decline slowly, as indicated by HPLC measurements (Figure 4b), which is potentially attributed to a slow accumulation of the pyridine byproduct and subsequent salt formation with the iCPA in the reactor. Therefore, a second fraction of the product was collected separately. The same overall yield of 93% was obtained for the second fraction, but the enantioselectivity dropped to 91.5:8.5 *e.r*. Nevertheless, we obtained another 6.98 g of the enantiomerically enriched product **13**, leading to a total yield of 9.76 g and an accumulated TON of 289. Compared with the literature, the observed TONs are on par with other examples in the field of heterogeneous Brønsted acid catalysis. For example, Pericàs et al. reported an accumulated TON of 102 for their aza-Friedel–Crafts addition of indoles to *N*-tosylimines,^39^ 243 for a desymmetrization of 3,3-disubstituted oxetanes with supported SPINOL-CPAs,^42^ 282 for their asymmetric allylation of aldehydes,^40^ and 43 for a Pictet-Spengler cyclization^41^ (both latter reactions catalyzed by supported TRIP).

To further investigate if the slight deactivation during the long-term experiment is reversible, the complete setup was flushed for 30 min (at 250 μL·min^–1^) with CHCl_3_ (+HOAc additive). To our delight, a subsequent test run with the reactivated catalyst showed the same results as those observed at the beginning of the large-scale reaction. A reactivation phase in between flow reactions is a common solution to counteract reversible deactivation processes during upscaling of flow reactions.^41^ In addition, transferring the reaction to continuous-flow heavily impacts the space-time yield (STY). For the homogeneous batch process, a STY of 30 mmol·L^–1^·h^–1^ was observed (reaction time 1.5 h, *t*Bu-substituted Hantzsch ester **11b** was used). Based upon the short residence time (τ = 4 min) and small reactor volume, the STY is significantly increased by a factor of 23 to 700 mmol·L^–1^·h^–1^. It is important to note that the exact same catalyst material was used for all flow experiments, i.e., optimization, substrate scope, and large-scale reaction, leading to a cumulated total running time of over 100 h and a TON of 372. After this extended time of use, iCPA was again submitted to elemental analysis to quantify possible leaching. It was found that under our reaction conditions, the loading only slightly decreased by 5% (from 0.21 to 0.20 mmol·g^–1^). Besides, a ^31^P NMR of the used catalyst was measured, indicating no chemical modification of the CPA. In combination with the excellent performance of the catalyst even after it was used for this extended time, this proves that leachability is negligible.

### Friedländer/TH-Reaction

**Figure 5:**
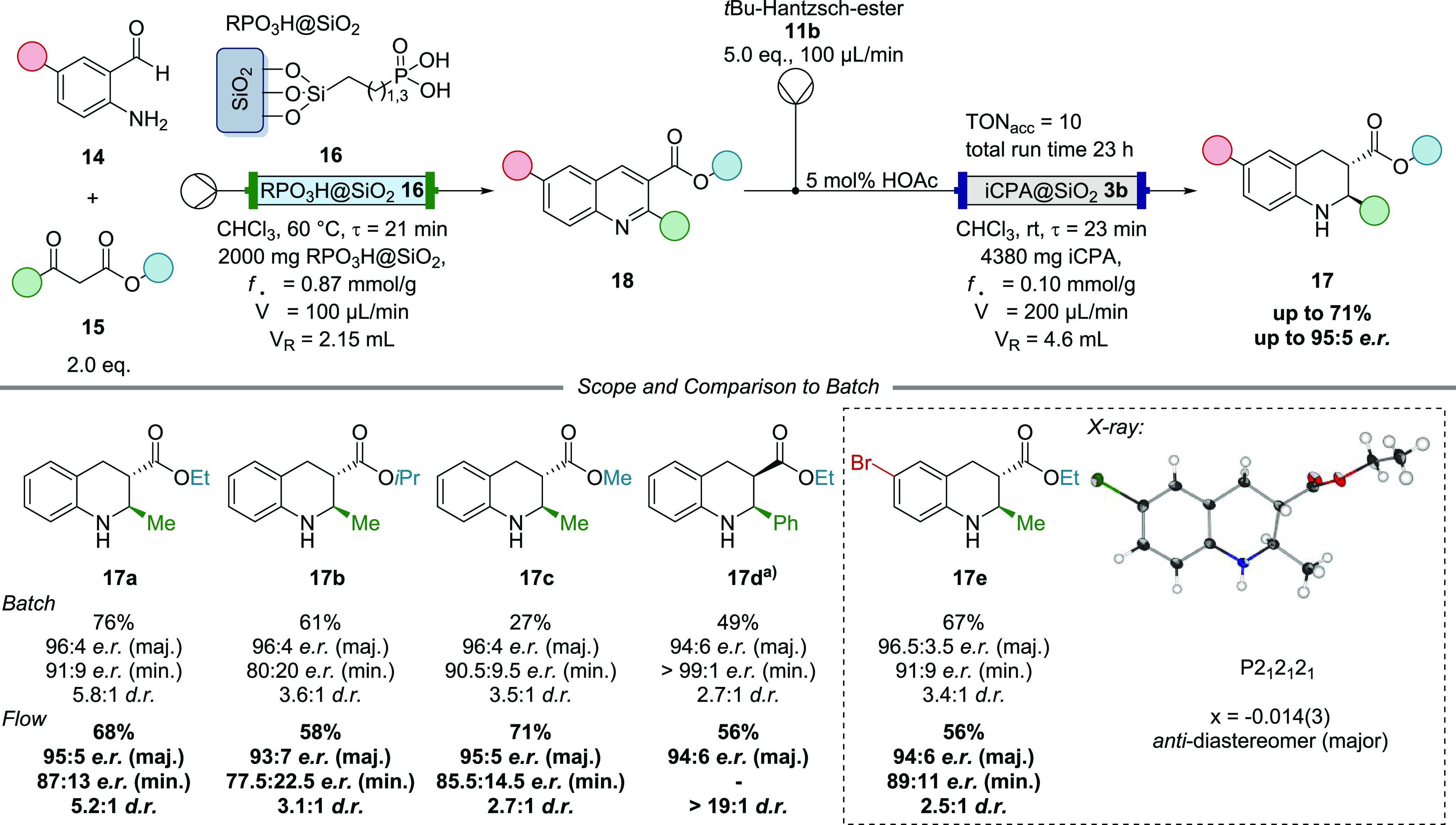
Friedländer-transfer hydrogenation sequence for the synthesis of 2,3-substituted tetrahydroquinolines in flow. The best results were obtained using 5 mol % HOAc as a cocatalyst and 5.0 equiv of dihydropyridine **11a** in chloroform (*c* = 0.05 mol·L^–1^). a) The 2-Ph-substituted product **17d** was identified to be *cis*-configured in accordance with spectroscopic data from literature.^56^

We then extended our studies to a sequential two-step flow process, the Friedländer quinoline synthesis–transfer hydrogenation cascade affording a variety of 2,3-disubstituted tetrahydroquinolines. This reaction was developed by Gong et al., who used a mixture of Mg(OTf)_2_ and a CPA as catalysts for the two individual steps.^55^ For this scenario, flow chemistry is ideally suited as one can employ two different fixed-bed reactors with two immobilized molecular catalysts sequentially (Figure 5). After extensive screening, we found commercially available immobilized phosphonic acid **16** to be an ideal catalyst for the Friedländer condensation giving rise to complete consumption of the starting material in short time under metal-free conditions (Table S3). Since both transformations occurred separately in two different reactors, it was possible to employ a strong achiral acid for the first step without any impact on the stereoselectivity of the asymmetric transfer hydrogenation. Under batch conditions, this would require additional filtration and workup steps, rendering it rather inconvenient. For the hydrogenation step, 2,4,6-Cy_3_-Ph-substituted CPA was identified as the optimal catalyst (Table S4). Upon further optimization of reaction parameters, including Hantzsch esters, solvents, additives, and stoichiometry, we established the ideal conditions for the batch process (Tables S5 and S6) giving rise to the product in 76% yield, with 96:4 *e.r*. (major diastereomer), 91:9 *e.r*. (minor diastereomer), and 5.8:1 *d.r*.

In the flow setup, the first column (ø 4 mm, length 250 mm) was packed with 2000 mg of immobilized phosphonic acid **16** to catalyze the Friedländer reaction of *ortho*-amino benzaldehydes **14** and β-keto esters **15** with a flow rate of 100 μL·min^–1^ and a residence time of 21 min at 60 °C. Subsequently, Hantzsch ester **11b** was added to the stream at 100 μL·min^–1^ and the mixture was passed through the second column (ø 4 mm, length 550 mm) containing 4380 mg of iCPA **3b**. A combined flow rate of 200 μL·min^–1^ led to a total residence time of 23 min for the transfer hydrogenation step. In this continuous-flow operation, product **17a** was isolated in 68% yield, 95:5 *e.r*. (major) and 5.2:1 *d.r*. (S7, entries 3–4).

We next investigated the substrate scope (Figure 5) in comparison between reactions run with the molecular catalyst in batch and the immobilized catalyst in flow. The first examples reveal a broad variability of the ester substituents giving rise to the 2,3-disubstituted tetrahydroquinolines in typically good yields (batch: 27–76%, flow: 58–71%), very good enantioselectivities for the major diastereomer (batch: 96:4 *e.r*., flow: 93:7 to 95:5 *e.r*.) and moderate *d.r*. (batch: 3.5:1 to 5.8:1, flow: 2.7:1 to 5.2:1). Introducing a methyl ester in the 3-position (**17c**) led to diminished yields under batch conditions due to the formation of side products. In the flow process, the reaction proceeded more cleanly and gave rise to an improved yield of 71%. The 2-substituent could also be changed to a phenyl group. Notably, in the batch reaction of this quinoline, product **17d** was obtained with only 49% yield, 2.7:1 *d.r*., and 94:6 *e.r*. (major diastereomer). In the corresponding flow process, however, this tetrahydroquinoline was isolated as a single diastereomer in 56% yield with 94:6 *e.r*. Thus, the migration of the process into continuous-flow resulted not only in an increased yield, but more importantly, the diastereoselectivity was significantly improved.

An X-ray crystal structure analysis of product **17e** revealed the *trans*-configuration of the two stereocenters, which was adopted for all other 2-methyl-substituted products as well.

### Mannich Reaction

Finally, the versatility of the iCPAs was assessed in a low-temperature C–C-bond-forming reaction. To the best of our knowledge, a continuous-flow reaction with an immobilized chiral Brønsted acid catalyst under cryogenic conditions has not been reported yet.

Based on our interest in Brønsted acid-catalyzed Mannich reactions,^9,10^ we studied the reaction of in situ formed aliphatic imines **19** and cyclic silyl dienolates **20**. An extensive optimization under batch conditions showed that this reaction worked best in ethereal solvents, especially THF, with co-catalytic amounts of DMPU at temperatures below −50 °C (Table S8). It was found to be crucial to keep the temperature below −25 °C to avoid side reactions. The best results were obtained in the presence of a 2,6-Me_2_-4-Ph-Ph-based CPA (10 mol %) and 20 mol % of DMPU at −50 °C in THF, giving rise to γ-amino ester **21** in quantitative yield as a single diastereomer with an excellent enantiomeric ratio of >99:1. Under the optimized conditions, the reaction proceeded to full conversion in less than 10 min.

Drawing on preliminary experiments with conventional column-based flow reactors, it became clear that mixing of the imine and nucleophile **20** must occur in the presence of the chiral catalyst to prevent a racemic background reaction. Thus, a self-made flow reactor with an integrated mixing unit was designed to solve this issue. In the flow process, first, amine **22** (*c* = 0.20 mol·L^–1^) and aldehyde **23** (*c* = 0.27 mol·L^–1^) were pumped through a mixer at a flow rate of 100 μL·min^–1^ each to rapidly generate the imine at −50 °C (residence time 60 s). Afterward, the imine solution (combined flow rate 200 μL·min^–1^) was passed through the self-made T-shaped stainless-steel reactor (loaded with 5515 mg of iCPA **3c**) at the same temperature. Using a third pump, the silyl dienolate was introduced inside the reactor (flow rate 200 μL·min^–1^, *c* = 0.4 mmol·L^–1^) to ensure that imine and the nucleophile react with each other only in the presence of the catalyst.

With this setup, the flow reaction was optimized regarding flow rates, stoichiometry, and temperature (see SI).

**Figure 6:**
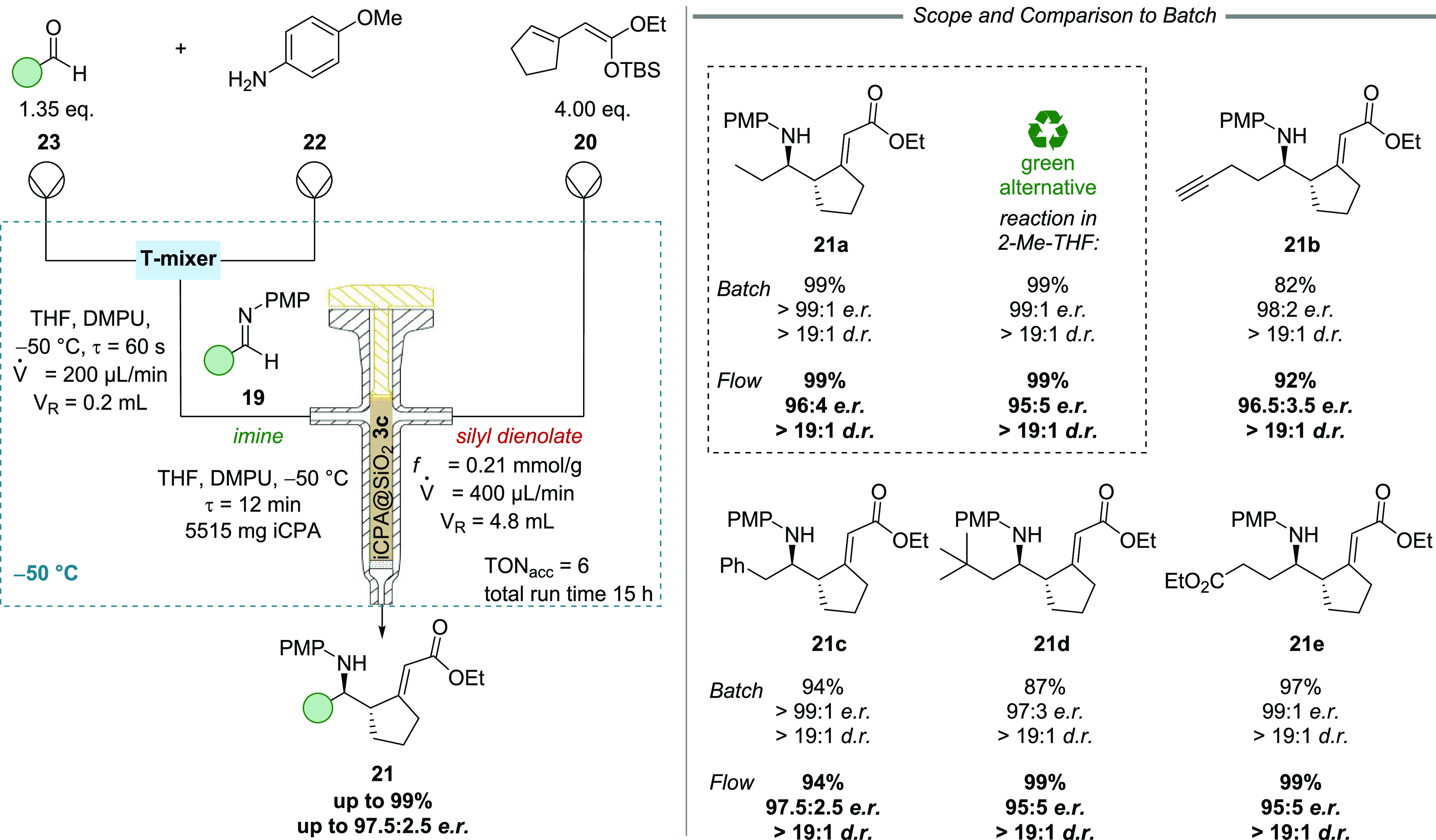
Vinylogous Mukaiyama–Mannich reaction with β,γ-bridged silyl dienolates and in situ generated aliphatic imines under cryogenic flow conditions.

For the substrate scope (Figure 6), several aliphatic aldehydes were submitted to the optimized homogeneous batch and heterogeneous flow conditions. In general, all products were obtained as single diastereomers and with excellent yields and enantioselectivities from the flow process, which were very similar in comparison to the batch process (batch: 97:3 to >99:1 *e.r*., flow: 95:5 to 97.5:2.5 *e.r*.) To our delight, very similar results were obtained when 2-Me-THF, a green solvent alternative, was used, allowing for environmentally yet more benign reaction conditions. Our studies represent the first successful implementation of an immobilized chiral catalyst into an automated continuous-flow processes under cryogenic temperatures.^57^

## Conclusions

In summary, a very robust, high yielding, and fully modular route toward immobilized chiral BINOL-phosphoric acids was developed. This conceptually unprecedented approach features prominently a broad flexibility with respect to the solid support material and catalyst substitution pattern. The iCPAs were fully characterized with ICP-OES and nitrogen sorption measurements, thermogravimetric and elemental analyses, STEM/EDX images, and solid-state NMR spectroscopy. Subsequently, the iCPAs were employed in three different continuous-flow processes including C–H and C–C-bond-forming reactions and a sequential C–C-/C–H-bond-forming reaction. In all cases, they gave rise to outstanding levels of productivity, space-time yields, and enantioselectivity. This strategy represents a breakthrough in the transition of stereoselective CPA catalysis to environmentally more benign and economically more valuable continuous-flow conditions and has the potential to expand the scope of applications significantly.

## Supporting Information Available

Additional experimental details; NMR spectra of all compounds and materials; HPLC chromatograms; crystallographic details; details on DFT calculations (PDF)

## Abbreviations

CPA: chiral phosphoric acid
iCPA: immobilized chiral phosphoric acid
TH: transfer hydrogenation
DMPU: *N*,*N*′-dimethylpropyleneurea
