# Supplementary material for: General, Modular Access toward Immobilized Chiral Phosphoric Acid Catalysts and Their Application in Flow Chemistry
Source: ACS Catal. 2024 Mar 29;14(8):5550–9. doi: 10.1021/acscatal.4c00985 (PMC11036403; doi:10.1021/acscatal.4c00985)
Supplement: Supplementary file 1 — cs4c00985_si_001.pdf [file cs4c00985_si_001.pdf]

Supplementary Information

A General, Modular Access towards Immobilized Chiral Phosphoric Acid Catalysts and their Application in Flow Chemistry

Michael Laue[a], Maximilian Schneider[a], Markus Gebauer[b], Winfried Böhlmann[c], Roger Gläser[b] and Christoph Schneider[*a]

a) Institute of Organic Chemistry, University of Leipzig, 04103 Leipzig, Germany,
b) Institute of Chemical Technology, University of Leipzig, 04103 Leipzig, Germany,
c) Division of Superconductivity and Magnetism, Felix-Bloch Institute for Solid-State Physics, University of Leipzig, 04103 Leipzig, Germany.

Corresponding author: Christoph Schneider, e-mail: schneider@chemie.uni-leipzig.de

Contents

1. Methods 1

1.1 General Information 1

1.2 Additional Information on reaction development 4

1.3 Experimental Procedures for Catalyst Synthesis and Characterization of molecular Catalyst Precursors 13

1.4 Preparation and characterization of solid supports 33

1.5 Synthesis and Characterization of immobilized Catalysts 37

1.6 Experimental Procedures for Batch and Flow Reactions 55

1.7 Characterization of Products 59

2. NMR Spectra of Catalyst Precursors 72

3. NMR Spectra of Products 100

4. HPLC Chromatograms 118

5. Crystallographic Data 147

6. DFT Calculations 154

References 159

1. Methods

1.1 General Information

All reactions in dry solvents were conducted under argon or nitrogen atmosphere, liquid reagents and solvents were transferred via syringe using standard Schlenk techniques. Dichloromethane, diethyl ether, tetrahydrofuran and toluene were dried by a solvent purification system (MB SPS-800 Braun). Chloroform (HPLC grade) and 2-Me-tetrahydrofuran (Emplura grade) were used without further purification. Solvents for extraction, column chromatography, preparative as well as analytical TLC were distilled from appropriate drying agents (hexane, diethyl ether: KOH, dichloromethane: CaH_2). Ethyl acetate and Methyl-*tert*-butyl ether were used without further purification. Flash chromatography was performed using Merck silica gel 60 230-400 mesh (0.040-0.063 mm). Preparative and analytical TLC was performed using silica gel pre coated plates ALUGRAM Xtra SIL G/ UV_{254} and aluminum oxide pre coated plates ALUGRAM ALOX N/ UV_{254} by Macherey Nagel. The spots were visualized by UV light (λ = 254 nm) and were treated with a vanillin/ sulfuric acid solution in MeOH (HPLC grade). Reactions that require heating were heated using Lab Armor metal beads.

^{1}H, ^{13}C and ^{31}P NMR spectra were recorded in $CDCl_3$ or DMSO-d^{6} using a Bruker Avance III HD (^{1}H: 400 MHz; ^{13}C: 100 MHz, ^{31}P: 162 MHz), a Varian MERCURYplus 400 (^{1}H: 400 MHz; ^{13}C: 100 MHz, ^{31}P: 162 MHz) and a Varian MERCURYplus 300 (^{1}H: 300 MHz; ^{13}C: 75 MHz, ^{31}P: 121 MHz) spectrometer. The signals were referenced to residual chloroform (δ = 7.26 ppm for ^{1}H NMR, δ = 77.16 ppm for ^{13}C NMR) or dimethyl sulfoxide (δ = 2.50 ppm for ^{1}H NMR, δ = 39.52 ppm for ^{13}C NMR). Chemical shifts are reported in ppm, multiplicities are indicated by s (singlet), d (doublet), t (triplet), q (quartet), hept (heptet), dd (doublet of doublet), dt (doublet of triplet), dq (doublet of quartet), td (triplet of doublet), qd (quartet of doublet), qt (quartet of triplet), ddd (doublet of doublet of doublets), ddt (doublet of doublet of triplets), dtd (doublet of triplet of doublets), dddd (doublet of doublet of doublet of doublets), m (multiplet) and the prefix br (broad). Solid state MAS-NMR spectra were measured on a Bruker Avance 400 with single pulse excitation. Melting points were determined with a Büchi M-560 melting point apparatus and are uncorrected. IR spectra were obtained with a FTIR spectrometer (JASCO FT/IR-4100) and are reported in frequency of absorption (cm^{-1}). Optical rotations were measured using a Polarotronic polarimeter (Schmidt & Haensch). All ESI mass spectra were recorded on a Bruker ESI-TOF microTOF. Elemental analysis was obtained with a Vario micro cube. ICP-OES was performed on

an Optima 8000 Dual-View from Perkin Elmer. Nitrogen sorption isotherms were recorded using a Belsorp Mini-X from Microtrac MRB. TGA was carried out using a STA 449 F1 Jupiter from Netzsch. Enantiomeric ratios (*e.r.*) were determined via HPLC on a JASCO MD-4015 instrument with a chiral stationary phase (Daicel Chiralpak IB, IE, column). Diastereomeric ratios were determined by HPLC or ^{1}H NMR analysis of the crude reaction mixture. TEM and EDX mapping were conducted using a Jeol JEM2100Plus device operated at 200 kV. For the Click-reactions a shaker and incubator (Heidolph Unimax 1010, Incubator 1010) was used. Flow reactions were done using an integrated flow chemistry system (Vapourtec E series, easy scholar). The used fixed bed reactors were either commercially available stainless-steel columns or in case of the Mannich reaction a self-build T-shaped stainless-steel reactor which is described in more detail in section 1.2.

Reactor Volume (V_R) calculated with:

$$V_R = \frac{(m_{flooded\ Reactor} - m_{dry\ Reactor})}{\rho_{solvent}}$$

The reactor was packed with the corresponding iCPA and the total mass ($m_{dry\ Reactor}$) was determined. After 0.5 h of flushing with the corresponding solvent (at 0.2 ml/min) the mass of the flushed reactor ($m_{flooded\ reactor}$) was determined again. Finally, the reactor volume V_R was calculated using the density of the solvent.

Space-Time-Yield (STY) calculated with:

$$STY_{Batch} = \frac{n_{Pr}}{V_r * t}$$

$$STY_{Flow} = \frac{\dot{n}_{Pr}}{V_R}$$

n_{Pr} = amount of product in mmol

V_r = reaction volume in L

t = time in h

$\dot{n}_{Pr}$ = product flow in mmol/h

V_R = reactor volume in L

Turnover-number (TON) calculated with:

$$TON = \frac{n_{Pr}}{n_{Cat}}$$

n_{Pr} = amount of product in mmol

n_{Cat} = amount of catalyst in mmol

Productivity ($Prod$) calculated with:

$$Prod = \frac{n_{Pr}}{n_{Cat} * t}$$

n_{Pr} = amount of product in mmol

n_{Cat} = amount of catalyst in mmol

t = time in h

1.2 Additional Information on reaction development

<u>Supplementary table S1:</u> Batch reaction development – transfer hydrogenation

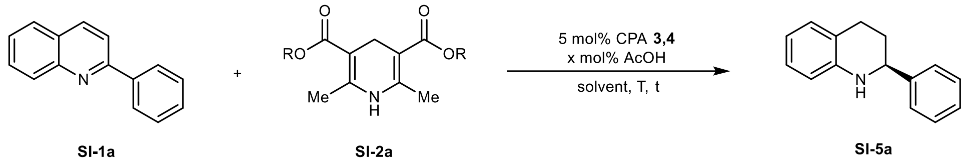

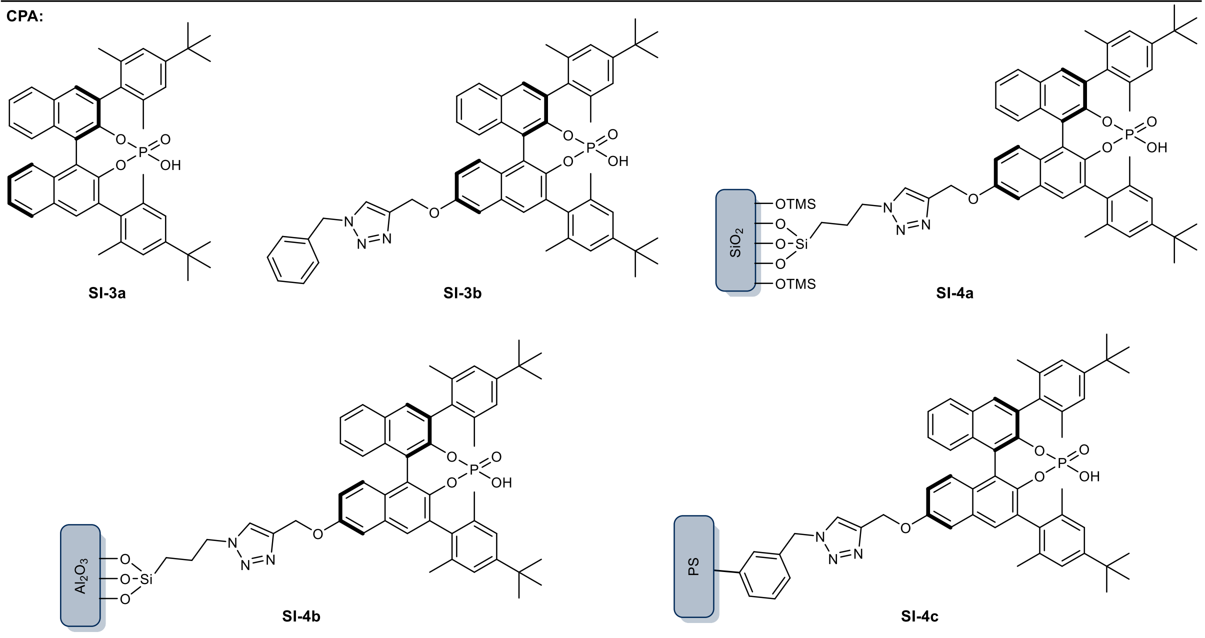

	Catalyst	t [h]	T [°C]	AcOH [mol%]	solvent	Yield	*e.r.*
**Lit[1]**	9-Phen-CPA	18	60	-	C_6H_6	92%[b)]	98.5:1.5
**1**	CPA **SI-3a**	0.5	60	-	$CHCl_3$	> 98%[c)]	> 99:1
**2**	CPA **SI-3a**	4	rt	10	$CHCl_3$	92%[b)]	99:1
**3**	CPA **SI-3b**	20	rt	10	$CHCl_3$	> 98%[c)]	98:2
**4**	iCPA@SiO_2 **SI-4a**	20	rt	10	$CHCl_3$	> 98%[c)]	98:2
**5**	iCPA@Alox **SI-4b**	20	rt	10	$CHCl_3$	> 98%[c)]	98:2
**6**	iCPA@PS **SI-4c**	20	rt	10	$CHCl_3$	> 98%[c)]	98:2
**7[a)]**	CPA **SI-3a**	2	rt	10	$CHCl_3$	97%[b)]	> 99:1

Reactions conducted on 0.10 mmol scale, 2.4 eq. of Hantzsch ester were used, 0.05 M in the corresponding solvent, *e.r.* determined by HPLC, **a)** O*t*Bu-HE (**SI-2b**) used instead of **SI-2a**, **b)** isolated yield, **c)** conversion was determined by ^1H NMR using 1,3,5-$(OMe)_3$ benzene as internal standard.

Supplementary table S2: Flow reaction development – transfer hydrogenation

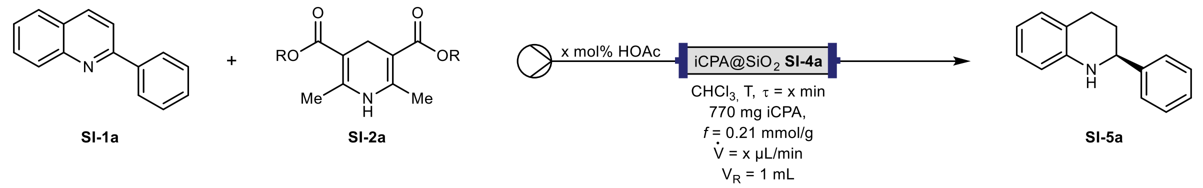

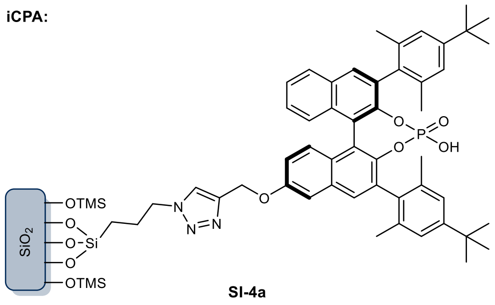

	flow rate [µl/min]	τ [min]	T [°C]	AcOH [mol%]	Yield	*e.r.*
**1**	100	10	70	-	> 98%[c)]	96.5:3.5[d)]
**2**	100	10	70	10	> 98%[c)]	93.5:6.5
**3**	100	10	rt	10	> 98%[c)]	96.5:3.5
**4**	100	10	rt	5	94%[b)]	96.5:3.5
**5**	200	5	rt	10	89%[b)]	97.5:2.5
**6**	250	4	rt	10	87%[b)]	98:2
**7**	250	4	rt	5	75%[c)]	98:2
**8**	250	4	rt	20	85%[c)]	97.5:2.5
**9**	500	2	rt	10	80%[b)]	97.5:2.5
**10[a)]**	**250**	**4**	**rt**	**10**	**91%[b)]**	**98:2**

Reactions conducted on 0.50 mmol scale, 2.4 eq. Hantzsch ester were used, 0.05 M in chloroform, *e.r.* determined by HPLC, **a)** O*t*Bu-HE (**SI-2b**) used instead of **SI-2a**, **b)** isolated yield, **c)** conversion was determined by ^{1}H NMR using 1,3,5-(OMe)$_3$ benzene as internal standard, **d)** *e.r.* decreasing over time.

Supplementary table S3: Batch reaction development – Friedländer reaction

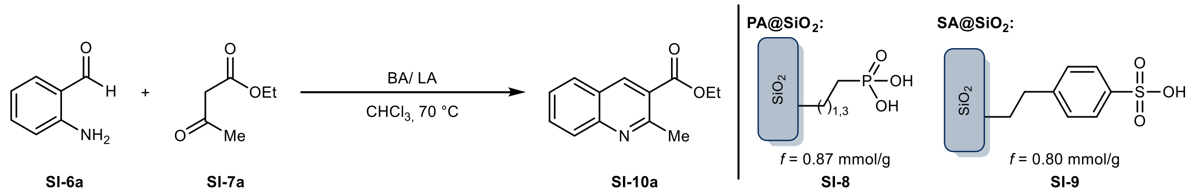

	AcOH [mol%]	$Mg(OTf)_2$ [mol%]	CPA [mol%]	$PA@SiO_2$ [mol%]	$SA@SiO_2$ [mol%]	Conversion[a)] after 20 h
**1**	-	-	5	-	-	35%
**2**	10	-	5	-	-	35%
**3**	-	10	5	-	-	80%
**4**	10	10	5	-	-	95%
**5**	10	-	-	-	-	5%
**6**	10	-	-	17.5	-	82%
**7**	10	-	-	175	-	> 98%[b)]
**8**	10	-	-	-	200	93%[b) c)]

Reactions conducted on 0.10 mmol scale, 2.0 eq. of keto ester were used, 0.05 M in chloroform, **a)** conversion was determined by ^1H NMR using 1,3,5-$(OMe)_3$ benzene as internal standard, **b)** Conversion after 5 h, **c)** $SA@SiO_2$ gets deactivated over time.

Supplementary table S4: Batch reaction development – transfer hydrogenation of Friedländer products – catalyst screening

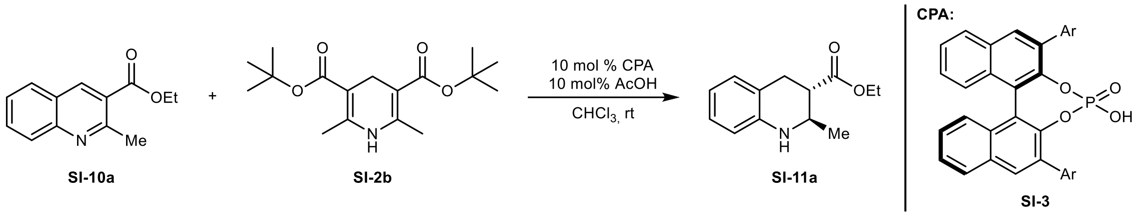

	Ar in CPA	Yield [%]	*e.r.* major	*e.r.* minor	*d.r.*
**1**	2,6-Me2-Ph	89	85:15	74:26	1.8:1
**2**	2,4,6-Me3-Ph	89	83:17	69:31	2.3:1
**3**	2,3,5,6-Me4-Ph	93	81:19	58:42	3.1:1
**4**	2,3,4,5,6-Me5-Ph	91	69:31	52:48	3.6:1
**5**	2,4,6-Et3-Ph	90	84:16	66:34	2.3:1
**6**	2,4,6-iPr3-Ph	96	96:4	83:17	3.3:1
**7**	**2,4,6-Cy3-Ph**	**96**	**96:4**	**86.5:13.5**	**4.3:1**
**8**	3,5-(CF3)2-Ph	87	75:25	62:38	1.2:1
**9**	9-anthracenyl	89	76:24	65:35	6.8:1
**10**	9-phenanthrenyl	88	88:12	74:26	6.4:1
**11**	2-triphenylenyl	88	65:35	33:67	2.3:1
**12**	4-NO2-Ph	87	54:46	45:55	1.6:1
**13**	2,6-Me2-4-*t*Bu-Ph	78	85:15	73:27	2.0:1
**14**	2,6-Me2-4-Naph-Ph	88	82:18	78:22	1.2:1
**15**	2,6-Me2-4-Anthr-Ph	90	91:9	91:9	1:1.2
**16**	2,6-Me2-4-Phen-Ph	88	83:17	79:21	1.1:1
**17**	2,6-Et2-4-Ph-Ph	89	80:20	66:34	1.7:1
**18**	2,6-Me2-4-Ph-Ph	87	78:22	69:31	1.7:1

Reactions conducted on 0.10 mmol scale, 2.4 eq. of Hantzsch ester were used, 0.05 M in chloroform, Yield was determined by ^{1}H NMR using 1,3,5-(OMe)$_3$ benzene as internal standard, *e.r.* determined by HPLC, *d.r.* determined by crude NMR.

Supplementary table S5: Batch reaction development – transfer hydrogenation of Friedländer products – Hantzsch ester and solvent screening

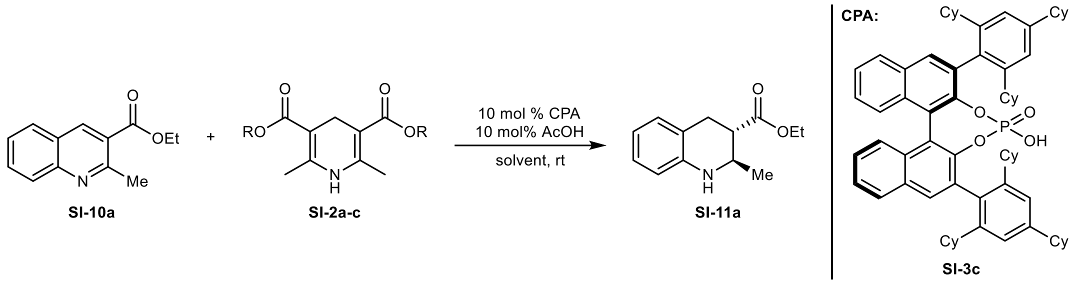

	R	solvent	Yield [%]	*e.r.* major	*e.r.* minor	*d.r.*
**1**	***t*Bu**	**$CHCl_3$**	**96**	**96:4**	**86.5:13.5**	**4.3:1**
**2**	Et	$CHCl_3$	90	97.5:2.5	83:17	3.2:1
**3**	Me	$CHCl_3$	91	95.5:4.5	81:19	3.8:1
**4**	*t*Bu	CCl_4	76	94:6	61:39	3.5:1
**5**	*t*Bu	CH_2Cl_2	99	93.5:6.5	85:15	3.8:1
**6**	*t*Bu	DCE	94	94.5:5.5	86:14	3.8:1
**7**	*t*Bu	toluene	99	95:5	77:23	3.9:1

Reactions conducted on 0.10 mmol scale, 2.4 eq. of Hantzsch ester were used, 0.05 M in the corresponding solvent, Yield was determined by ^{1}H NMR using 1,3,5-$(OMe)_3$ benzene as internal standard, *e.r.* determined by HPLC, *d.r.* determined by crude NMR.

Supplementary table S6: Batch reaction development – transfer hydrogenation of Friedländer products – acid additive and Hantzsch ester equivalent screening

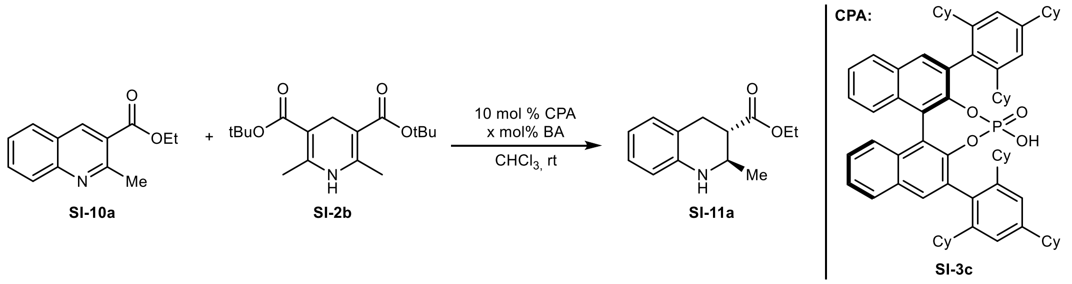

	BA	x mol%	HE [eq.]	pK_a	*e.r.* major	*e.r.* minor	*d.r.*
**1**	AcOH	10	2.4	4.8	96:4	86.5:13.5	4.3:1
**2**	*m*-$(CF_3)_2$-Ph-$B(OH)_2$	10	2.4	7.2	96:4	86:14	5.1:1
**3**	Ph-$B(OH)_2$	10	2.4	8.9	96.5:3.5	94:6	5.1:1
**4**	*p*-F-Ph-$B(OH)_2$	10	2.4	9.1	96.5:3.5	94:6	5.1:1
**5**	Ph-OH	10	2.4	10	96:4	91:9	5.1:1
**6**	Ph-$B(OH)_2$	10	5.0	8.9	97:3	94:6	5.5:1
**7**	**AcOH**	**5**	**5.0**	**4.8**	**96:4**	**91:9**	**5.8:1**

Reactions conducted on 0.10 mmol scale, 0.05 M in chloroform, *e.r.* was determined by HPLC, *d.r.* determined by crude NMR.

Supplementary table S7: Flow reaction development – Friedländer reaction transfer hydrogenation Cascade

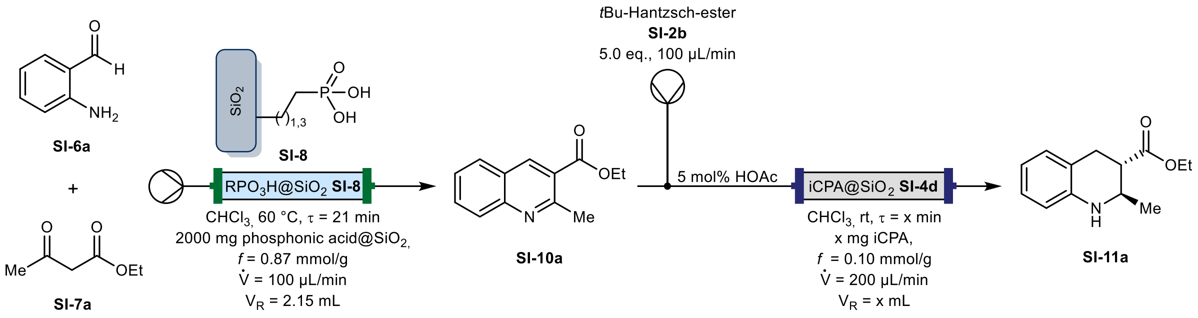

	BA	mol%	m_{iCPA} [mg]	τ [min]	Yield [%]	*e.r.* major	*e.r.* minor	*d.r.*
**1**	AcOH	5	2048	10.5	55	95:5	88.5:11.5	5.0:1
**2**	Ph-B(OH)$_2$	10	2048	10.5	25	92:8[a)]	76:24[a)]	4.0:1
**3**	AcOH	5	2900	15	65	95:5	87:13	5.2:1
**4**	**AcOH**	**5**	**4381**	**23**	**68**	**95:5**	**87:13**	**5.2:1**

Reactions conducted on 0.25 mmol scale, 2.0 eq. keto ester were used, first step 0.05 M, second step 0.025 M in chloroform, isolated yields, *e.r.* was determined by HPLC, *d.r*. determined by crude NMR, τ corresponds to reactor 2 (transfer hydrogenation), **a)** *e.r.* was decreasing over time.

Supplementary table S8: Batch reaction development – Mannich reaction

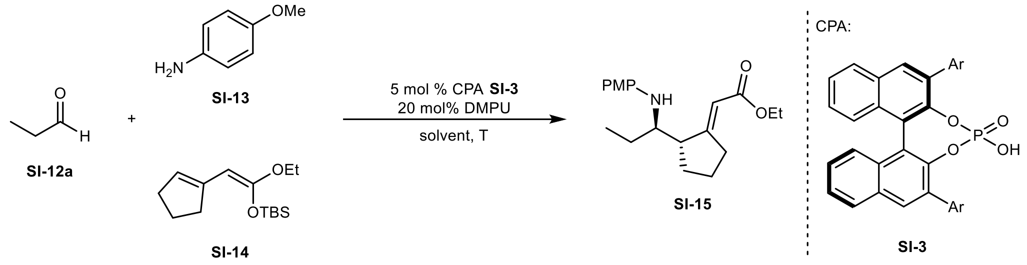

	CPA	solvent	T [°C]	Yield [%]	*e.r.*	*d.r.*
**1**	2,6-Me_2-4-*t*Bu-Ph	THF	-40	90	96:4	> 19:1
**2**	2,6-Me_2-4-*t*Bu-Ph	CPME	-40	90	96:4	> 19:1
**3**	2,6-Me_2-4-*t*Bu-Ph	THF	0	decomp.	-	-
**4**	2,6-Me_2-4-*t*Bu-Ph	THF	-15	42	92.5:7.5	> 19:1
**5**	2,6-Me_2-4-*t*Bu-Ph	THF	-25	87	95:5	> 19:1
**6**	2,6-Me_2-4-*t*Bu-Ph	THF	-50	92	96.5:3.5	> 19:1
**7**	2,4,6-*i*Pr_3-Ph	THF	-50	89	90:10	> 19:1
**8**	3,5-$(CF_3)_2$-Ph	THF	-50	78	46:54	10:1
**9**	9-anthracenyl	THF	-50	92	98.5:1.5	> 19:1
**10**	**2,6-Me_2-4-Ph-Ph**	**THF**	**-50**	**99**	**> 99:1**	**> 19:1**
**11**	2,6-Me_2-4-Ph-Ph	2-Me THF	-50	99	99:1	> 19:1

Reactions conducted on 0.20 mmol scale, 1.25 eq. Aldehyde and 2.00 eq. nucleophile were used, *p*-anisidine 0.10 M in the corresponding solvent, isolated yields, *e.r.* was determined by HPLC, *d.r.* was determined by HPLC of the crude product.

Supplementary table S9: Flow reaction development – Mannich reaction

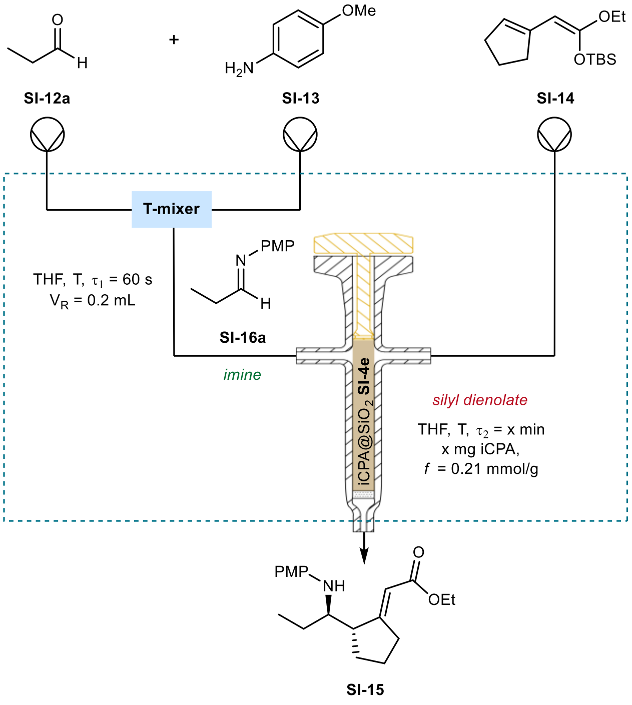

	n (An:Ald:Nu) [eq]	V̇ (An:Ald:Nu) [µl/min]	m_{iCPA} [mg]	τ_2 [min]	T [°C]	Yield [%]	*e.r.*	*d.r.*
**1**	1 : 1.5 : 3	100 : 100 : 100	3330	10	-30	78	93:7	> 19:1
**2**	1 : 1.5 : 4	100 : 100 : 100	3330	10	-30	99	94:6	> 19:1
**3**	1 : 1.5 : 8	100 : 100 : 200	3330	7.5	-30	99	95:5	> 19:1
**4**	**1 : 1.3 : 4**	**100 : 100 : 200**	**5515**	**12**	**-50**	**99**	**96:4**	**> 19:1**
**5[a)]**	1 : 1.3 : 4	100 : 100 : 200	5515	12	-50	99	95:5	> 19:1

Reactions conducted on 0.50 mmol scale with 1.0 eq. DMPU, isolated yields, *e.r.* was determined by HPLC, *d.r.* was determined by HPLC of the crude product, imine formation 0.10 M in THF, Mannich reaction 0.05 M in THF, **a)** reaction in 2-Me-THF.

<u>Supplementary figure SF1:</u> Technical illustration and picture T-shaped flow reactor

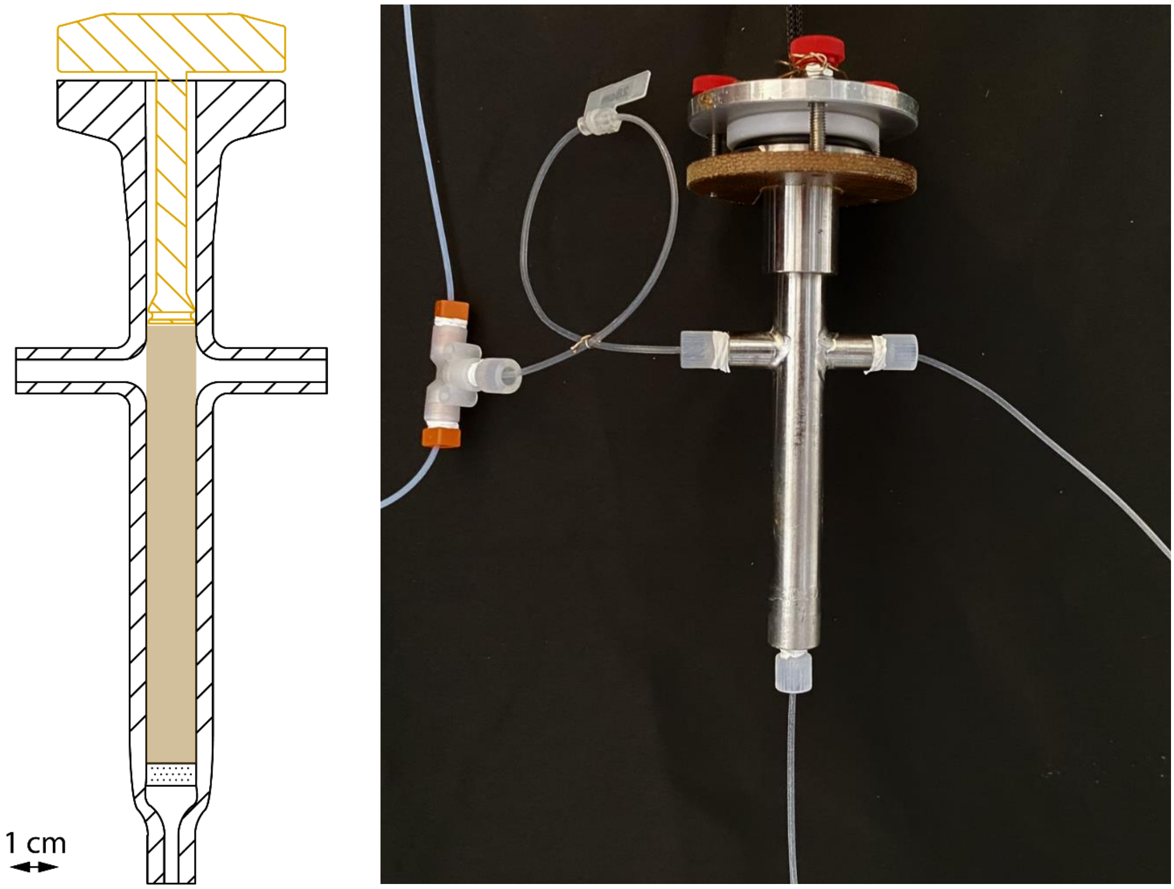

A stainless-steel reactor (diameter 1 cm, ca. 10 cm length) with an integrated mixing unit was build. The reactor features two inlets (1/8" screw connectors) and one outlet. At the bottom of the reactor a Teflon frit is located. The catalyst is loaded from the top and a flange with Teflon stopper is used to seal the apparatus.

1.3 Experimental Procedures for Catalyst Synthesis and Characterization of molecular Catalyst Precursors

(*R*)-2′-Hydroxy[1,1′-binaphthalen]-2-yl 2,2-dimethylpropanoate **SI-18**:

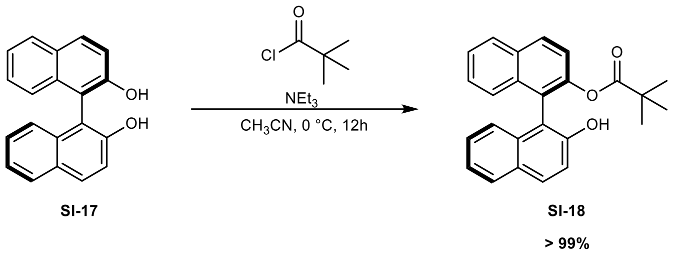

Following a procedure from Jasra[2], to a solution of 100.0 g (349.3 mmol) (*R*)-BINOL in 1000 ml CH_3CN (0.35 M) was added 49.2 ml (352 mmol, 1.01 eq.) NEt_3 at 0 °C. Afterwards 43.4 ml (352 mmol, 1.01 eq.) pivaloyl chloride was added dropwise over three hours under stirring. After addition the mixture was stirred for 12 hours at ambient temperature (TLC). The reaction was stopped by addition of 250 ml saturated aqueous $NaHCO_3$ and the water phase was extracted with 200 ml EtOAc three times. The organic phase was washed with 1 M HCl and brine, dried over $MgSO_4$, filtered and the solvent was evaporated under reduced pressure. 130.0 g (**quant. yield, 95% purity**) of the desired product were obtained as a yellow solid and used without further purification.

$\mathbf{R_f}$ = 0.61 (Hex/ EtOAc 5:1).

^{1}H NMR (400 MHz, $CDCl_3$): δ = 8.08 (d, J = 8.9 Hz, 1H), 7.98 (d, J = 8.2 Hz, 1H), 7.89 (d, J = 8.9 Hz, 1H), 7.83 (d, J = 8.2 Hz, 1H), 7.51 (ddd, J = 8.2, 6.2, 1.9 Hz, 1H), 7.41 – 7.29 (m, 5H), 7.28 – 7.22 (m, 1H), 7.06 (d, J = 8.4 Hz, 1H), 5.14 (s, 1H), 0.78 (s, 9H).

^{13}C (APT) NMR (100 MHz, $CDCl_3$): δ = 177.9, 151.9, 148.4, 133.7, 133.6, 132.3, 130.8, 130.4, 129.1, 128.4, 128.0, 127.5, 126.8, 126.3, 125.8, 124.7, 123.6, 123.2, 121.9, 118.3, 114.3, 38.84, 26.56.

(*R*)-6′-Bromo-2′-hydroxy[1,1′-binaphthalen]-2-yl 2,2-dimethylpropanoate **SI-19**:

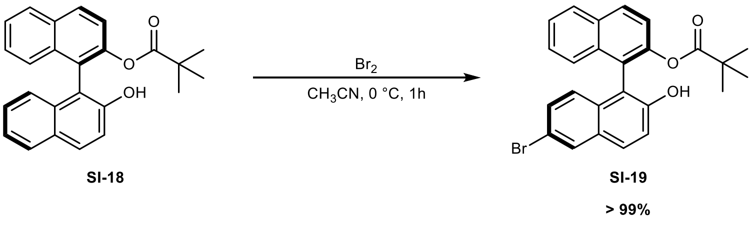

In accordance to Jasra[2], a stirred solution of 130.0 g (349.3 mmol) **SI-18** in 1000 ml CH_3CN (0.35 M) was cooled to 0 °C and 36.0 ml (702 mmol, 2.00 eq.) Br_2 was added dropwise over one hour. After complete addition the reaction was stopped by addition of 200 ml aqueous $Na_2S_2O_3$ solution, phases were separated and the water phase was extracted three times with EtOAc. The organic phase was washed with aqueous $NaHCO_3$, brine and 1 M HCl, dried over $MgSO_4$ and filtered. The solvent was evaporated under reduced pressure to give 160.0 g (**quant. yield, 95% purity**) of the desired product as a yellow solid, which was used in the next step without further purification.

R_f = 0.45 (Hex/ EtOAc 5:1).

^{1}H NMR (400 MHz, $CDCl_3$): δ = 8.08 (d, J = 8.1 Hz, 1H), 8.01 – 7.95 (m, 2H), 7.79 (d, J = 9.0 Hz, 1H), 7.52 (ddd, J = 8.1, 6.8, 1.3 Hz, 1H), 7.41 – 7.24 (m, 5H), 6.93 (d, J = 9.0 Hz, 1H), 5.20 (s, 1H), 0.81 (s, 9H).

^{13}C (APT) NMR (100 MHz, $CDCl_3$): δ = 178.0, 152.3, 148.4, 133.5, 132.3, 131.2, 130.2, 130.0, 130.0, 129.5, 128.5, 127.7, 126.6, 126.5, 125.0, 122.5, 122.0, 119.6, 117.4, 114.6, 38.91, 26.63.

<u>(*R*)-6-Bromo[1,1′-binaphthalene]-2,2′-diol **SI-20**:</u>

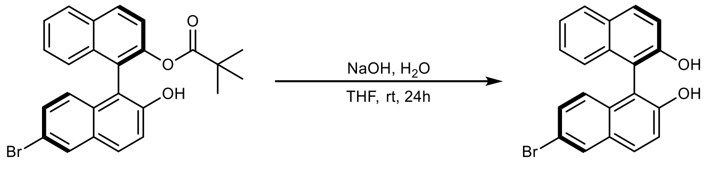

SI-19

SI-20
> 99%

As described in literature[2], 160.0 g (349.3 mmol) of compound **SI-19** were dissolved in 1000 ml (0.35 M) THF under stirring. then 160 ml H_2O and 534 ml 2 M NaOH (1.14 mol, 3.00 eq.) were added and the brown solution was stirred at rt until full consumption was indicated by TLC. The mixture was diluted with EtOAc, phases were separated and the aqueous phase was extracted three times with 200 ml EtOAc. The organic phases were washed with 1 M HCl, saturated aqueous $NaHCO_3$ and NH_4Cl-solution. After drying over $MgSO_4$ and filtration the solvent was evaporated under reduced pressure to give 130.1 g (**> 99%**) *R*-6-Bromo-BINOL as a yellow solid.

R_f = 0.72 (Hex/ EtOAc 1:1).

^{1}H NMR (400 MHz, $CDCl_3$): δ = 8.05 (d, J = 2.1 Hz, 1H), 7.98 (d, J = 8.6 Hz, 1H), 7.92 – 7.85 (m, 2H), 7.42 – 7.29 (m, 5H), 7.10 (dd, J = 8.3, 1.2 Hz, 1H), 7.02 (d, J = 8.9 Hz, 1H), 5.11 (s, 2H).

^{13}C (APT) NMR (100 MHz, $CDCl_3$): δ = 153.1, 152.8, 133.5, 132.2, 131.7, 130.7, 130.6, 130.4, 130.4, 129.5, 128.6, 127.7, 126.3, 124.2, 124.2, 119.1, 117.9, 117.9, 111.6, 110.5.

(*R*)-6-Methoxy-[1,1′-binaphthalene]-2,2′-diol **SI-21**:

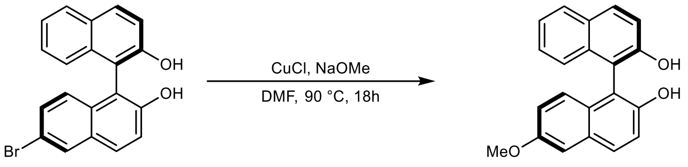

SI-20 **SI-21** **> 99%**

To a stirred solution of 119.6 g (327.7 mmol) (*R*)-6-Bromo[1,1′-binaphthalene]-2,2′-diol in 1000 ml (0.33 M) DMF were added 97.3g (983.2 mmol, 3.00 eq.) CuCl and 1050 mL (17.0 eq., 4.4 M in MeOH) sodium methoxide under an argon atmosphere. After being stirred for 16 h at 90 °C, the mixture was cooled to room temperature. Ice-water was poured into the reaction mixture, and 6 M HCl was added to neutralize the solution. The whole mixture was transferred to a separating funnel where it was extracted with Et_2O. The organic phase was washed with aqueous 1 M LiCl, dried over Na_2SO_4, filtered, and concentrated in vacuo. The residue was purified by flash silica gel column chromatography (Hex /EtOAc = 3:1 $^{v}/_{v}$) to give the desired product as a white solid in 103.7 g (**> 99%**).

R_f = 0.50 (Hex/ EtOAc 4:1).

^{1}H NMR (400 MHz, $CDCl_3$): δ = 7.96 (d, J = 9.0 Hz, 1H), 7.90 – 7.84 (m, 2H), 7.40 – 7.33 (m, 3H), 7.33 – 7.28 (m, 1H), 7.22 (d, J = 2.6 Hz, 1H), 7.15 (dd, J = 8.4, 1.1 Hz, 1H), 7.06 (d, J = 9.2 Hz, 1H), 6.98 (dd, J = 9.2, 2.6 Hz, 1H), 5.10 (s, 1H), 4.93 (s, 1H), 3.91 (s, 3H).

^{13}C (APT) NMR (100 MHz, $CDCl_3$): δ = 156.6, 152.8, 151.2, 133.6, 131.5, 130.6, 130.2, 129.6, 128.7, 128.5, 127.6, 126.0, 124.4, 124.2, 120.0, 118.3, 117.9, 111.3, 111.2, 107.1, 55.52.

HRMS (+ESI) m/z: [M+Na] Calc. for $C_{21}H_{16}O_3Na$: 339.0992, found 339.0994.

IR (KBr) ṽ [cm^{-1}] = 3476, 3434, 3059, 2960, 2937, 2837, 1619, 1599, 1512, 1465, 1427, 1368, 1345, 1313, 1236, 1214, 1168, 1142, 1123, 1070, 1032, 947, 853, 820, 751, 681, 650, 617, 579, 557, 423.

[α_D^{23}] = - 65° (c = 1.00, $CHCl_3$).

MP = 64 °C.

(*R*)-6-Methoxy-2,2′-bis(methoxymethoxy)-1,1′-binaphthalene **SI-22**:

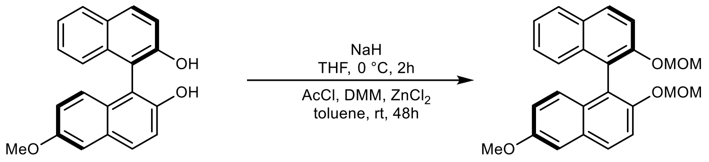

SI-21 **SI-22**
> 99%

In a first flaks under Ar-atmosphere 103.7 g (327.7 mmol) (*R*)-6-Methoxy-BINOL was dissolved in 1600 ml (0.2 M) THF and cooled to 0 °C. Then 39.3 g (983.1 mmol, 3.00 eq.) NaH (60%wt dispersion in mineral oil) was added portion wise under stirring over a period of one hour and the mixture was stirred for two additional hours.
In a second flask 447.6 mg (3.3 mmol, 0.01 eq.) $ZnCl_2$ was flame-dried under vacuum. After cooling down to ambient temperature 145.0 ml (1.64 mol, 5.00 eq.) Dimethoxymethane (DMM) and 435 ml (3.0 vol-eq. in relation to DMM) toluene were added under Ar-atmosphere. Then 116.9 ml (1.64 mol, 5.00 eq.) acetyl chloride was added dropwise over a period of 30 minutes (exothermic reaction, the temperature should not exceed 50 °C). Afterwards the mixture was stirred for one hour while cooling down to ambient temperature. The colorless solution of MOMCl was then added dropwise to the mixture in the first flask at 0 °C.
The mixture was stirred for one day (TLC) while warming up to ambient temperature and was stopped by addition of H_2O and saturated aqueous NH_4Cl. The phases were separated and the aqueous phase was extracted three times with dichloromethane. The organic phase was washed with saturated aqueous NH_4Cl and brine and dried over $MgSO_4$. After filtration and evaporation of the solvent the crude product was obtained as an emulsion in mineral oil which was washed and sonicated with pentane at 0 °C several times to remove the mineral oil. Then 132.5 g (**> 99%**) of the MOM-protected BINOL could be obtained as a white solid.

R_f = 0.50 (Hex/ EtOAc 7:1).

^{1}H NMR (400 MHz, $CDCl_3$): δ = 7.95 (d, J = 8.6 Hz, 1H), 7.91 – 7.82 (m, 2H), 7.56 (dd, J = 11.2, 9.0 Hz, 2H), 7.35 (ddd, J = 8.1, 6.6, 1.3 Hz, 1H), 7.26 – 7.14 (m, 3H), 7.08 (d, J = 9.0 Hz, 1H), 6.92 (dd, J = 9.2, 2.6 Hz, 1H), 5.11 (d, J = 6.7 Hz, 1H), 5.04 (d, J = 6.7 Hz, 1H), 4.98 (d, J = 6.7 Hz, 1H), 4.94 (d, J = 6.7 Hz, 1H), 3.91 (s, 3H), 3.17 (s, 3H), 3.14 (s, 3H).

^{13}C (APT) NMR (100 MHz, $CDCl_3$): δ = 156.6, 152.7, 151.3, 134.2, 131.1, 130.0, 129.5, 129.5, 128.1, 127.9, 127.3, 126.4, 125.7, 124.2, 122.0, 121.5, 119.1, 118.4, 117.4, 106.0, 95.71, 95.33, 55.97, 55.90, 55.39.

HRMS (+ESI) m/z: [M+Na] Calc. for $C_{25}H_{24}O_5Na$: 427.1516, found 427.1527.

IR (KBr) ṽ [cm^{-1}] = 3436, 3060, 2996, 2955, 2899, 2826, 1625, 1595, 1506, 1466, 1435, 1376, 1354, 1333, 1305, 1241, 1198, 1149, 1069, 1035, 1012, 961, 921, 890, 850, 811, 751, 693, 597.

[α_D^{23}] = - 42° (c = 1.00, $CHCl_3$).

MP = 102 °C.

(*R*)-3,3’-Diiodo-6-methoxy-2,2′-bis(methoxymethoxy)-1,1′-binaphthalene **SI-23**:

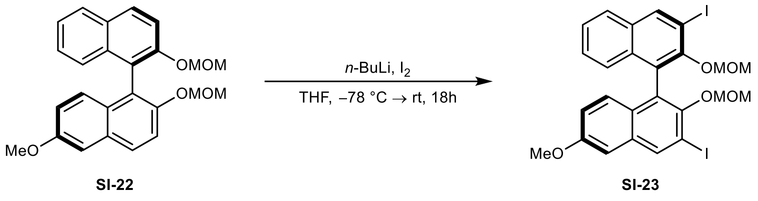

Under Ar-atmosphere a solution of 42.0 g (103.8 mmol) of **SI-22** in 1040 ml (0.1 M) THF was cooled to -78 °C, 100 ml (249.2 mmol, 2.4 eq.) of a 2.5 M *n*-BuLi solution in hexane was added dropwise and the mixture was stirred for one hour while warming to ambient temperature. Then the mixture was cooled to -78 °C again and 79.1 g (311.5 mmol, 3.00 eq.) iodine was added dropwise as a 2 M solution in 150 ml THF over a period of 30 minutes. The solution was stirred over 14 hours (TLC) while warming to ambient temperature. The reaction was stopped by addition of saturated aqueous $Na_2S_2O_3$, phases were separated and the aqueous phase was extracted with EtOAc three times. The organic phase was washed with brine, dried over $MgSO_4$ and filtered. The solvent was evaporated under reduced pressure and the crude product was purified by flash column chromatography (Hex/ EE 20:1 v/v). The diiodo-BINOL could be obtained in 47.0 g (**69%**) as a yellow solid.

R_f = 0.53 (Hex/ EtOAc 2:1).

^{1}H NMR (400 MHz, $CDCl_3$): δ = 8.53 (s, 1H), 8.43 (s, 1H), 7.77 (d, J = 8.2 Hz, 1H), 7.42 (ddd, J = 8.2, 6.8, 1.2 Hz, 1H), 7.30 (ddd, J = 8.2, 6.8, 1.3 Hz, 1H), 7.17 (d, J = 8.2 Hz 1H), 7.11 – 7.02 (m, 2H), 6.96 (dd, J = 9.4, 2.5 Hz, 1H), 4.81 (d, J = 5.6 Hz, 1H), 4.79 (d, J = 5.6 Hz, 1H), 4.70 (d, J = 5.6 Hz, 1H), 4.66 (d, J = 5.6 Hz, 1H), 3.90 (s, 3H), 2.65 (s, 3H), 2.58 (s, 3H).

^{13}C (APT) NMR (100 MHz, $CDCl_3$): δ = 157.6, 152.2, 150.6, 140.1, 138.7, 134.0, 133.6, 132.3, 129.3, 128.2, 127.2, 126.9, 126.6, 126.5, 126.5, 126.0, 120.0, 104.8, 99.55, 99.51, 93.30, 92.61, 56.70, 56.58, 55.49.

HRMS (+ESI) m/z: [M+Na] Calc. for $C_{25}H_{22}I_2O_5Na$: 678.9449, found 678.9499 .

IR (KBr) ṽ [cm^{-1}] = 3444, 2996, 2957, 2931, 2903, 2826, 1622, 1562, 1493, 1463, 1419, 1370, 1346, 1231, 1200, 1159, 1084, 1031, 998, 961, 927, 897, 825, 751, 528.

[α_D^{23}] = - 23° (c = 1.00, $CHCl_3$).

MP = decomposes >150 °C.

<u>(3,5-Dimethyl-[1,1′-biphenyl]-4-yl)boronic acid **SI-25**:</u>

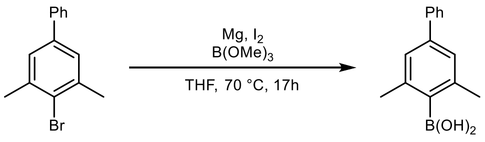

SI-24 **SI-25**

85%

<u>Preparation of the Grignard solution:</u> To a suspension of 3.57 g (147 mmol, 1.50 eq.) Mg in 10 ml THF was added 0.01 eq. Iodine under Ar-atmosphere. Then a solution of 25.6 g (98.0 mmol) **SI-24** in 190 ml (0.5M) THF was added slowly. After complete addition the mixture was stirred at 70 °C for two hours.

<u>Boronic acid synthesis:</u> The mixture was cooled to 0 °C and a solution of 32.8 ml (294.1 mmol, 3.00 eq.) trimethyl borate in 20 ml THF was added slowly. The mixture was stirred for half an hour at this temperature followed by 17 h at ambient temperature. Afterwards 125 ml 2 M HCl was added at 0 °C and the mixture was stirred for another three hours. The mixture was extracted four times with CH_2Cl_2, the organic phase was washed with 1 M HCl, dried over Na_2SO_4 and filtered. Evaporation of the solvent under reduced pressure gives the crude product which was purified by trituration with cold hexane (four times, - 20 °C). After evaporation of solvent residues

under reduced pressure, 18.8 g (**85%**) of boronic acid **SI-25** was isolated as a white solid.

^{1}H NMR (300 MHz, DMSO-d$_6$): δ = 8.17 (s, 2H), 7.65 – 7.57 (m, 2H), 7.48 – 7.39 (m, 2H), 7.38 – 7.29 (m, 1H), 7.22 (s, 2H), 2.34 (s, 6H).

^{13}C (APT) NMR (75 MHz, DMSO-d$_6$): δ = 140.6, 139.3, 139.2, 128.8, 127.1, 126.5, 124.1, 22.06.

HRMS (+ESI) m/z: [M+Na] Calc. for $C_{14}H_{15}BO_2Na$: 248.1094, found 248.1093.

IR (KBr) ṽ [cm^{-1}] = 3348, 3029, 2922, 1635, 1606, 1546, 1499, 1439, 1403, 1338, 1176, 1123, 1078, 1016, 873, 826, 765, 746, 702, 678, 665, 551.

MP = 329 °C.

General procedure Suzuki-coupling:

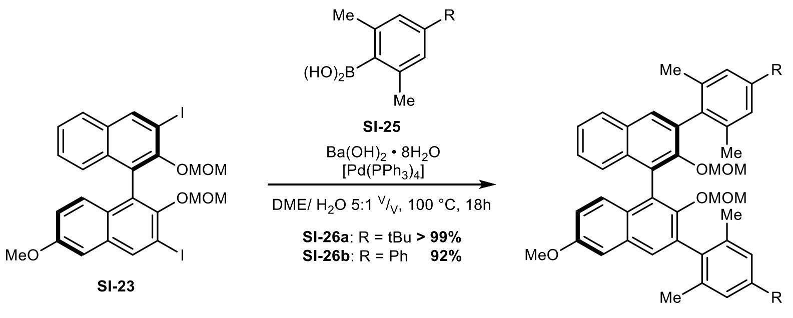

Prepared according to literature procedure.[3] Under Ar-atmosphere 12.5 mmol of the diiodo-BINOL **SI-23**, 50 mmol (4.00 eq.) boronic acid, 50 mmol (4.00 eq.) $Ba(OH)_2$•$8H_2O$ and 2.50 mmol (20 mol%) [$Pd(PPh_3)_4$] were dissolved in a mixture of degassed DME /H_2O 5:1 $^{v}/_{v}$ (0.1 M) and the yellow mixture was stirred at 100 °C for 18 h (TLC). The reaction was stopped by addition of saturated aqueous NH_4Cl solution at ambient temperature, extracted three times with CH_2Cl_2, washed with water, dried over $MgSO_4$ and filtered. The solvent was evaporated under reduced pressure before the crude product was purified by flash column chromatography on silica (Hex/ EtOAc 20:1 $^{v}/_{v}$).

(*R*)-3,3'-Bis(4-(*tert*-butyl)-2,6-dimethylphenyl)-6-methoxy-2,2'-bis(methoxymethoxy)-1,1'-binaphthalene **SI-26a**:

Prepared and isolated according to general procedure Suzuki-coupling as a white solid with a yield of > **99%**.

R_f = 0.43 (Hex/ EtOAc 4:1).

^{1}H NMR (400 MHz, $CDCl_3$): δ = 7.83 (dd, J = 8.1, 1.2 Hz, 1H), 7.75 (s, 1H), 7.64 (s, 1H), 7.40 (ddd, J = 8.1, 6.4, 1.6 Hz, 1H), 7.36 – 7.22 (m, 3H), 7.18 – 7.11 (m, 5H), 7.00 – 6.95 (m, 1H), 4.34 – 4.22 (m, 4H), 3.91 (s, 3H), 2.32 (s, 3H), 2.27 (s, 3H), 2.25 (s, 3H), 2.24 (s, 3H), 2.18 (s, 3H), 2.17 (s, 3H), 1.34 (s, 18H).

^{13}C (APT) NMR (100 MHz, $CDCl_3$): δ = 157.2, 152.0, 150.3, 150.3, 136.9, 136.9, 136.4, 136.4, 135.7, 135.6, 135.5, 134.9, 133.7, 132.1, 131.0, 130.7, 129.4, 129.0, 127.9, 127.9, 126.6, 126.5, 126.3, 126.2, 125.0, 124.4, 124.4, 124.4, 124.3, 118.9, 105.9, 98.15, 55.64, 55.55, 55.44, 34.47, 31.58, 21.48, 21.15.

HRMS (+ESI) m/z: [M+Na] Calc. for $C_{49}H_{56}O_5Na$: 747.4020, found 747.4066.

IR (KBr) ṽ [cm^{-1}] = 3443, 3060, 2961, 2865, 2823, 1626, 1597, 1493, 1463, 1447, 1391, 1377, 1362, 1351, 1248, 1228, 1200, 1156, 1119, 1080, 1035, 999, 973, 937, 926, 905, 869, 830, 751, 674, 541.

[α_D^{23}] = - 12° (c = 1.00, $CHCl_3$).

MP = 147 °C.

(*R*)-3,3'-Bis(3,5-dimethyl-[1,1'-biphenyl]-4-yl)-6-methoxy-2,2'-bis(methoxymethoxy)-1,1'-binaphthalene **SI-26b**:

Prepared and isolated according to general procedure Suzuki-coupling as a white solid with a yield of **92%**.

R_f = 0.55 (Hex/ EtOAc 3:1).

^{1}H NMR (400 MHz, $CDCl_3$): δ = 7.89 (d, J = 8.1 Hz, 1H), 7.80 (s, 1H), 7.71 – 7.64 (m, 5H), 7.50 – 7.33 (m, 13H), 7.30 (d, J = 9.2 Hz, 1H), 7.20 (d, J = 2.6 Hz, 1H), 7.03 (dd, J = 9.2, 1.8 Hz, 1H), 4.47 – 4.29 (m, 4H), 3.94 (s, 3H), 2.41 (s, 3H), 2.36 (d, J = 1.0 Hz, 6H), 2.35 (s, 3H), 2.29 (s, 3H), 2.28 (s, 3H).

^{13}C (APT) NMR (100 MHz, $CDCl_3$): δ = 157.3, 152.0, 150.3, 141.3, 140.3, 140.2, 138.0, 138.0, 137.9, 137.8, 137.5, 137.4, 135.1, 134.6, 133.8, 132.1, 131.0, 130.7, 129.4, 129.1, 128.9, 128.0, 127.9, 127.3, 127.2, 127.2, 126.6, 126.6, 126.4, 126.3, 126.2, 126.2, 126.2, 125.2, 119.2, 106.0, 98.35, 98.33, 55.82, 55.73, 55.46, 21.41, 21.06.

HRMS (+ESI) m/z: [M+Na] Calc. for $C_{53}H_{48}O_5Na$: 787.3394, found 787.3386.

IR (KBr) ṽ [cm^{-1}] = 3433, 2952, 2921, 1625, 1599, 1502, 1495, 1477, 1465, 1447, 1377, 1351, 1248, 1228, 1156, 1130, 1077, 1033, 998, 972, 930, 873, 764, 756, 698.

[α_D^{23}] = - 66° (c = 1.00, $CHCl_3$).

MP = 136-137 °C.

(*R*)-6-Methoxy-2,2'-bis(methoxymethoxy)-3,3'-bis(2,4,6-tricyclohexylphenyl)-1,1'-binaphthalene **SI-26c**:

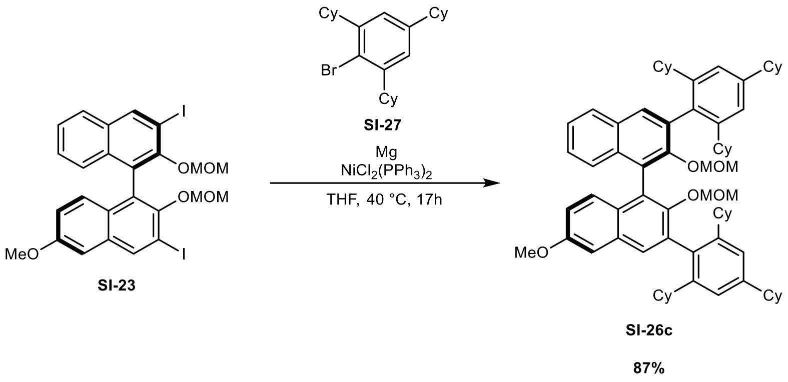

Prepared according to literature procedure.[4]

Preparation of the Grignard solution: To a suspension of 1.79 g (73.5 mmol, 7.50 eq.) Mg in 5 ml THF was added 0.01 eq. iodine under Ar-atmosphere. Then a quarter of a suspension of 19.8 g (49.0 mmol 5.00 eq.) aryl bromide in 90 ml (0.5 M) THF was added and the reaction was started by addition of 0.2 ml 1,2-dibromo ethane. The rest of the suspension was added dropwise in a speed that kept the reaction refluxing. After complete addition the mixture was stirred at 70 °C for 7 h.

Kumada-coupling: In a second flask 6.44 g (9.80 mmol) diiodo-BINOL **SI-23** and 0.64 g (0.98 mmol, 10 mol%) $NiCl_2(PPh_3)_2$ were suspended in 5 ml THF and the Grignard solution was added dropwise over a period of 10 min (THF all together 100 ml, 0.1 M). The mixture was stirred for 17 h at 40 °C until full consumption was indicated by TLC. The mixture was poured into saturated aqueous NH_4Cl solution, extracted with CH_2Cl_2, dried over Na_2SO_4 and filtered. The solvent was evaporated under reduced pressure and the crude product was purified by flash column chromatography (Hex/ CH_2Cl_2 4:1 $^v/_v$) to give 8.92g (**87%**) of the product as a white solid.

R_f = 0.45. (Hex/ CH_2Cl_2 2:1).

^{1}H NMR (300 MHz, $CDCl_3$): δ = 7.83 (d, J = 8.1 Hz, 1H), 7.74 (s, 1H), 7.63 (s, 1H), 7.41 (dt, J = 8.1, 4.0 Hz, 1H), 7.31 (d, J = 4.0 Hz, 2H), 7.20 (d, J = 9.2 Hz, 1H), 7.12

(d, J = 2.6 Hz, 1H), 7.05 (s, 4H), 6.98 (dd, J = 9.2, 2.6 Hz, 1H), 4.38 (d, J = 5.3 Hz, 1H), 4.32 (d, J = 5.3 Hz, 1H), 4.27 (d, J = 5.3 Hz, 1H), 4.23 (d, J = 5.3 Hz, 1H), 3.93 (s, 3H), 2.65 – 2.33 (m, 6H), 2.30 (s, 3H), 2.25 (s, 3H), 1.99 – 1.04 (m, 60H).

^{13}C (APT) NMR (75 MHz, $CDCl_3$): δ = 157.1, 152.1, 150.4, 147.4, 147.3, 146.5, 146.4, 146.4, 146.3, 134.9, 134.4, 133.9, 133.7, 133.6, 131.6, 131.0, 130.5, 129.8, 129.1, 127.9, 127.8, 126.2, 126.2, 126.1, 126.1, 125.0, 122.1, 118.7, 106.0, 97.41, 55.45, 55.39, 55.29, 45.00, 42.22, 42.17, 41.80, 41.78, 36.19, 36.12, 34.79, 33.27, 27.39, 27.32, 27.18, 27.00, 26.96, 26.70, 26.49, 26.42, 26.38.

HRMS (+ESI) m/z: [M+H] Calc. for $C_{73}H_{92}O_5$: 1048.6945, found 1048.7296.

IR (KBr) ṽ [cm^{-1}] = 3433, 2925, 2850, 1626, 1606, 1502, 1463, 1447, 1350, 1228, 1158, 1133, 1079, 1000, 972, 930, 862, 749.

[α_D^{23}] = +43° (c = 1.00, $CHCl_3$).

MP = 195-196 °C.

<u>General procedure MOM-deprotection:</u>

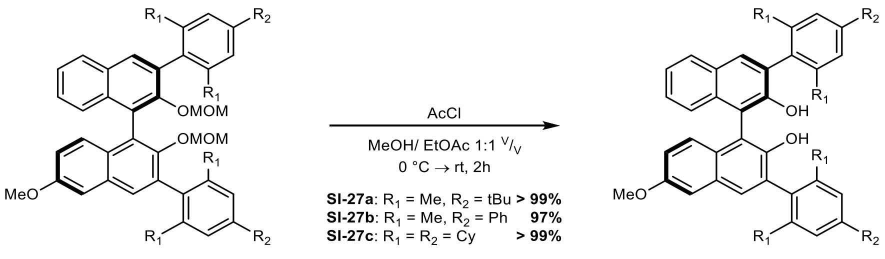

SI-26a-c SI-27a-c

In a flask a 2.0 M solution of AcCl in 100 ml MeOH was prepared at 0 °C by dropwise addition. The solution was stirred for 20 min at this temperature and afterwards added dropwise to a 0.1 M solution of the MOM-protected BINOLs **SI-26a-c** in 100 ml EtOAc at 0 °C. The mixture was stirred at this temperature for another two hours. After full consumption was indicated by TLC the solvents were evaporated under reduced pressure to give the pure products **SI-27a-c** (if there are multiple spots on TLC a flash column chromatography at this step is highly recommended).

<u>(*R*)-3,3'-Bis(4-(*tert*-butyl)-2,6-dimethylphenyl)-6-methoxy-[1,1'-binaphthalene]-2,2'-diol **SI-27a**:</u>

Prepared according to general procedure MOM-deprotection with a yield of **> 99%** as a white solid. The crude product was used in the next step without further purification.

R_f = 0.35 (Hex/ EtOAc 4:1).

^{1}H NMR (400 MHz, $CDCl_3$): δ = 7.86 (dd, J = 8.1, 1.5 Hz, 1H), 7.76 (s, 1H), 7.66 (s, 1H), 7.38 (ddd, J = 8.1, 6.7, 1.5 Hz, 1H), 7.32 (ddd, J = 8.1, 6.7, 1.5 Hz, 1H), 7.28 – 7.24 (m, 1H), 7.22 – 7.13 (m, 6H), 7.00 (dd, J = 9.0, 2.7 Hz, 1H), 5.03 (s, 1H), 4.86 (s, 1H), 3.92 (s, 3H), 2.20 (s, 3H), 2.19 (s, 3H), 2.12 (s, 3H), 2.11 (s, 3H), 1.36 (s, 18H).

^{13}C (APT) NMR (100 MHz, $CDCl_3$): δ = 156.4, 150.9, 150.1, 148.4, 136.9, 136.8, 136.8, 133.6, 133.1, 133.1, 130.7, 130.4, 130.1, 129.7, 129.5, 129.4, 128.8, 128.3, 126.9, 126.3, 125.6, 124.9, 124.8, 124.7, 123.9, 119.4, 113.4, 106.7, 55.49, 34.56, 31.53, 21.03, 20.95.

HRMS (+ESI) m/z: [M+Na] Calc. for $C_{45}H_{48}O_3Na$: 659.3496, found 659.3518.

IR (KBr) ṽ [cm^{-1}] = 3527, 3444, 3058, 2962, 2920, 2865, 1604, 1500, 1439, 1380, 1362, 1327, 1305, 1256, 1228, 1176, 1165, 1146, 1118, 1064, 1035, 995, 922, 894, 869, 828, 749, 682, 669, 603.

[α_D^{23}] = - 7° (c = 1.00, $CHCl_3$).

MP = 210-215 °C.

(*R*)-3,3'-Bis(3,5-dimethyl-[1,1'-biphenyl]-4-yl)-6-methoxy-[1,1'-binaphthalene]-2,2'-diol **SI-27b**:

Prepared according to general procedure MOM-deprotection, the crude product was purified by flash column chromatography on silica (Hex/ EtOAc 20:1 to 9:1 $^v/_v$) and isolated as a slightly yellow solid with a yield of **97%**.

R_f = 0.51 (Hex/ EtOAc 3:1).

^{1}H NMR (400 MHz, $CDCl_3$): δ = 7.93 (d, J = 7.6 Hz, 1H), 7.83 (s, 1H), 7.73 (s, 1H), 7.67 (d, J = 7.6 Hz, 4H), 7.50 – 7.41 (m, 9H), 7.41 – 7.34 (m, 3H), 7.32 (d, J = 7.0 Hz, 1H), 7.26 – 7.19 (m, 2H), 7.07 (dd, J = 9.2, 2.5 Hz, 1H), 5.11 (s, 1H), 4.94 (s, 1H), 3.95 (s, 3H), 2.30 (s, 3H), 2.29 (s, 3H), 2.23 (s, 3H), 2.22 (s, 3H).

^{13}C (APT) NMR (100 MHz, $CDCl_3$): δ = 156.6, 150.0, 148.4, 141.2, 141.2, 141.1, 141.1, 137.8, 137.8, 137.7, 135.4, 135.3, 133.6, 130.8, 130.5, 129.8, 129.6, 129.6, 129.4, 128.8, 128.8, 128.5, 127.4, 127.3, 127.3, 127.2, 126.7, 126.6, 126.2, 124.7, 124.2, 119.8, 113.3, 113.2, 106.8, 55.53, 20.97, 20.88, 20.86.

HRMS (-ESI) m/z: [M-H] Calc. for $C_{49}H_{39}O_3$: 675.2894, found 675.2889.

IR (KBr) ṽ [cm^{-1}] = 3524, 3059, 3030, 2952, 2918, 1601, 1500, 1477, 1440, 1381, 1366, 1255, 1228, 1174, 1165, 1126, 1034, 873, 824, 764, 753, 697, 599.

[α_D^{23}] = -23° (c = 1.00, $CHCl_3$).

MP = 236-238 °C.

<u>(*R*)-6-Methoxy-3,3'-bis(2,4,6-tricyclohexylphenyl)-[1,1'-binaphthalene]-2,2'-diol **SI-27c**:</u>

Prepared according to general procedure MOM-deprotection with a yield of **> 99%** as a white solid. The crude product was used in the next step without further purification.

R_f = 0.61 (Hex/ EtOAc 14:1).

^{1}H NMR (400 MHz, $CDCl_3$): δ = 7.87 (d, J = 8.0 Hz, 1H), 7.72 (s, 1H), 7.61 (s, 1H), 7.37 (ddd, J = 8.0, 6.3, 1.8 Hz, 1H), 7.35 – 7.27 (m, 2H), 7.21 – 7.17 (m, 2H), 7.11 (s, 2H), 7.08 (s, 2H), 7.00 (dd, J = 9.1, 2.6 Hz, 1H), 4.83 (s, 1H), 4.67 (s, 1H), 3.93 (s, 3H), 2.61 – 2.41 (m, 4H), 2.32 – 2.20 (m, 2H), 2.02 – 1.02 (m, 60H).

^{13}C (APT) NMR (100 MHz, $CDCl_3$): δ = 156.3, 150.7, 149.1, 148.1, 148.1, 147.0, 147.0, 146.8, 133.4, 131.1, 131.0, 130.5, 130.0, 129.8, 129.4, 129.2, 128.7, 128.5, 126.6, 125.9, 124.4, 123.7, 122.5, 119.2, 113.4, 113.4, 106.8, 55.46, 44.96, 42.00, 41.94, 41.89, 34.87, 34.84, 34.69, 34.64, 34.62, 34.39, 34.36, 34.09, 27.30, 27.27, 27.18, 27.16, 27.11, 27.02, 27.01, 26.95, 26.91, 26.41, 26.38, 26.34, 26.20.

HRMS (-ESI) m/z: [M-H] Calc. for $C_{69}H_{83}O_3$: 959.6337, found 959.6257.

IR (KBr) ṽ [cm^{-1}] = 3525, 3426, 2925, 2850, 1618, 1604, 1508, 1500, 1463, 1448, 1426, 1384, 1255, 1228, 1177, 1166, 1130, 1117, 1036, 952, 893, 862, 828, 747.

[α_D^{23}] = +105° (c = 1.00, $CHCl_3$).

MP = 218-220 °C.

General procedure Phosphorylation:

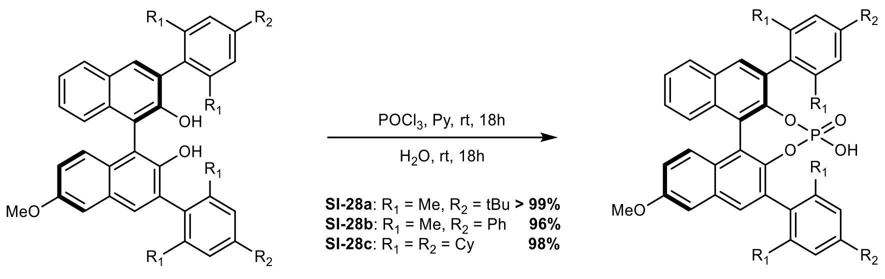

SI-27a-c SI-28a-c

To a solution of BINOLs **SI-27a-c** in abs. pyridine (0.1 M) was added 3.50 eq. $POCl_3$ dropwise under Ar-atmosphere at 0 °C. The solution was stirred for 18 h while warming to ambient temperature. After full consumption of BINOL was indicated by TLC the mixture was cooled to 0 °C and water (1 ml per mmol BINOL) was added dropwise. The reaction was stirred for another 18 h while warming to ambient temperature. Then 1 M HCl (10 ml per mmol BINOL) was added and the reaction was stirred for 30 mins. The mixture was extracted three times with CH_2Cl_2, the organic phase was washed with HCl (3x 1 M, 2x 2 M, 1x 6 M), dried over Na_2SO_4 and filtered. The solvent was evaporated under reduced pressure to give the BINOL phosphoric acids as typically off-white solids which were used in the next step without further purification.

(*R*)-2,6-Bis(4-(*tert*-butyl)-2,6-dimethylphenyl)-4-hydroxy-9-methoxydinaphtho-[1,3,2] dioxaphosphepine 4-oxide **SI-28a**:

Prepared according to general procedure Phosphorylation, isolated as a white solid with a yield of **> 99%**.

R_f = 0.02 (Hex/ EtOAc 9:1).

^{1}H NMR (400 MHz, $CDCl_3$): δ = 7.89 (d, J = 8.2 Hz, 1H), 7.77 (s, 1H), 7.67 (s, 1H), 7.49 (ddd, J = 8.2, 6.6, 1.4 Hz, 1H), 7.39 – 7.29 (m, 2H), 7.28 – 7.24 (m, 1H), 7.18 (d, J = 2.6 Hz, 1H), 6.99 (dd, J = 9.3, 2.6 Hz, 1H), 6.97 – 6.93 (m, 4H), 3.96 (s, 3H), 2.18 (s, 3H), 2.17 (s, 3H), 2.02 (s, 3H), 2.01 (s, 3H), 0.97 (s, 9H), 0.96 (s, 9H).

^{31}P NMR (162 MHz, $CDCl_3$): δ = 5.93.

^{13}C (APT) NMR (75 MHz, $CDCl_3$): δ = 157.5, 150.1, 150.0, 136.4, 136.4, 136.2, 133.6, 133.6, 133.2, 133.2, 133.0, 132.2, 131.8, 131.8, 130.5, 128.8, 128.3, 127.4, 127.2, 126.4, 125.7, 124.9, 124.0, 123.9, 122.6, 122.5, 119.1, 106.1, 55.53, 34.09, 31.15, 21.50, 21.04, 21.01.

HRMS (-ESI) m/z: [M-H] Calc. for $C_{45}H_{46}O_5P$: 697.3077, found 697.3201.

IR (KBr) ṽ [cm^{-1}] = 3434, 3055, 2962, 2867, 1628, 1505, 1463, 1395, 1378, 1362, 1303, 1281, 1248, 1229, 1200, 1157, 1119, 1096, 1023, 999, 967, 925, 896, 870, 847, 828, 751, 710, 608, 583, 505.

[α_D^{23}] = - 20° (c = 1.00, $CHCl_3$).

MP = 340-350 °C.

(*R*)-2,6-Bis(3,5-dimethyl-[1,1'-biphenyl]-4-yl)-4-hydroxy-9-methoxydinaphtho-[1,3,2] dioxaphosphepine 4-oxide **SI-28b**:

Prepared according to general procedure Phosphorylation, isolated as a white solid with a yield of **96%**.

R_f = 0.00 (Hex/ EtOAc 4:1).

^{1}H NMR (400 MHz, $CDCl_3$): δ = 10.11 (s, 1H), 7.96 (d, J = 8.2 Hz, 1H), 7.83 (s, 1H), 7.72 (s, 1H), 7.57 (t, J = 7.7 Hz, 1H), 7.47 – 7.37 (m, 2H), 7.37 – 7.29 (m, 5H), 7.25 (d, J = 2.6 Hz, 1H), 7.17 (s, 4H), 7.07 (dd, J = 9.3, 2.6 Hz, 1H), 7.03 (br-s, 2H), 6.87 (br-s, 4H), 3.99 (s, 3H), 2.28 (s, 3H), 2.25 (s, 3H), 1.80 (s, 3H), 1.78 (s, 3H).

^{31}P NMR (162 MHz, $CDCl_3$): δ = 5.86.

^{13}C (APT) NMR (100 MHz, $CDCl_3$): δ = 157.8, 145.2, 145.1, 143.5, 143.4, 141.5, 140.7, 140.6, 137.5, 137.3, 137.3, 135.0, 134.8, 133.3, 133.2, 133.1, 132.9, 132.8, 132.1, 131.8, 131.6, 130.3, 129.0, 128.5, 128.3, 127.4, 127.3, 127.3, 126.9, 126.8, 126.7, 126.6, 126.1, 125.9, 122.5, 122.5, 122.4, 119.4, 106.2, 55.61, 21.31, 20.48.

HRMS (-ESI) m/z: [M-H] Calc. for $C_{49}H_{38}O_5P$: 737.2451, found 737.2448.

IR (KBr) ṽ [cm^{-1}] = 3432, 3058, 3030, 2957, 2922, 1627, 1601, 1567, 1504, 1478, 1446, 1434, 1399, 1378, 1319, 1281, 1253, 1229, 1208, 1200, 1177, 1149, 1142, 1131, 1091, 1025, 997, 966, 897, 876, 839, 764, 755, 712, 698, 607, 580.

[α_D^{23}] = +11° (c = 1.00, $CHCl_3$).

MP = 345 °C.

(*R*)-4-Hydroxy-9-methoxy-2,6-bis(2,4,6-tricyclohexylphenyl)dinaphtho-[1,3,2] dioxaphosphepine 4-oxide **SI-28c**:

Prepared according to general procedure Phosphorylation, isolated as an off-white solid with a yield of **98%**.

R_f = 0.04 (Hex/ EtOAc 9:1).

^{1}H NMR (400 MHz, $CDCl_3$): δ = 7.83 (d, J = 8.2 Hz, 1H), 7.69 (s, 1H), 7.58 (s, 1H), 7.44 (ddd, J = 8.2, 5.9, 2.0 Hz, 1H), 7.32 – 7.22 (m, 2H), 7.16 – 7.08 (m, 2H), 6.94 (dd, J = 9.3, 2.5 Hz, 1H), 6.92 – 6.86 (m, 4H), 3.93 (s, 3H), 2.50 – 2.37 (m, 2H), 2.21 – 2.05 (m, 4H), 1.98 – 0.50 (m, 60H).

^{31}P NMR (162 MHz, $CDCl_3$): δ = 1.21.

^{13}C (APT) NMR (100 MHz, $CDCl_3$): δ = 157.3, 147.0, 146.6, 146.4, 146.2, 144.9, 132.8, 132.3, 132.2, 131.9, 131.8, 131.0, 130.7, 128.4, 128.2, 127.5, 126.8, 126.2, 125.5, 122.5, 122.4, 121.9, 121.8, 121.6, 118.8, 106.3, 55.54, 44.87, 42.28, 42.24, 41.90, 37.14, 35.23, 35.17, 34.77, 34.33, 33.29, 32.75, 27.48, 27.29, 27.26, 27.13, 26.95, 26.51, 26.41.

HRMS (-ESI) m/z: [M-H] Calc. for $C_{69}H_{82}O_5P$: 1021.5894, found 1021.5886.

IR (KBr) ṽ [cm^{-1}] = 3544, 3416, 2925, 2850, 1627, 1605, 1504, 1448, 1282, 1229, 1200, 1019, 999, 971, 897, 862, 750.

[$α_D^{23}$] = -31° (c = 1.00, $CHCl_3$).

MP = 367 °C.

General procedure Me-deprotection:

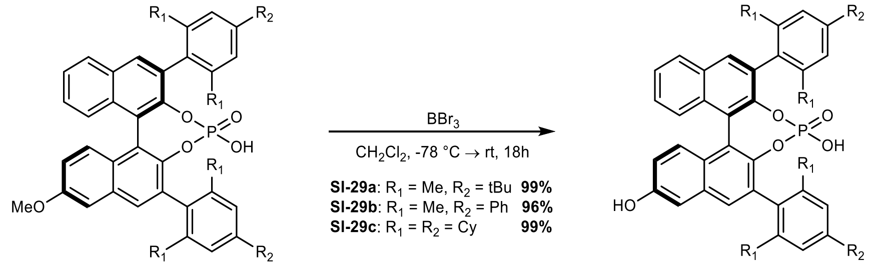

SI-28a-c SI-29a-c

In a flame-dried flask under Ar-atmosphere CPA (**SI-28a-c**) was dissolved in 0.05 M CH_2Cl_2 and cooled to -78 °C. Then 3.00 eq. of a 1 M solution of BBr_3 in CH_2Cl_2 was added at this temperature. The mixture was stirred for two hours at -78 °C and 18 h

while warming to ambient temperature. After full consumption (TLC) the reaction was stopped by addition of water (10 ml per mmol CPA). The mixture was extracted with CH_2Cl_2 three times, washed with 1 M HCl and water, dried over Na_2SO_4 and filtered. Evaporation of solvent gives the CPAs **SI-29a-c** typically as off-white solids which were used in the next step without further purification.

(*R*)-2,6-Bis(4-(*tert*-butyl)-2,6-dimethylphenyl)-4,9-dihydroxydinaphtho-[1,3,2] dioxaphosphepine 4-oxide **SI-29a**:

Prepared according to general procedure Me-deprotection, isolated as an off-white solid with a yield of **99%**.

R_f = 0.39 (Hex/ EtOAc/ MeOH 6:3:1).

^{1}H NMR (400 MHz, $CDCl_3$): δ = 7.90 (d, J = 8.1 Hz, 1H), 7.79 (s, 1H), 7.59 (s, 1H), 7.49 (ddd, J = 8.1, 6.7, 1.3 Hz, 1H), 7.38 (d, J = 8.5 Hz, 1H), 7.31 (ddd, J = 8.5, 6.7, 1.3 Hz, 1H), 7.26 (d, J = 9.2 Hz, 1H), 7.19 (d, J = 2.6 Hz, 1H), 7.01 – 6.94 (m, 4H), 6.91 (dd, J = 9.2, 2.6 Hz, 1H), 2.19 (s, 6H), 2.03 (s, 6H), 0.97 (s, 9H), 0.96 (s, 9H).

^{31}P NMR (162 MHz, $CDCl_3$): δ = 6.20.

^{13}C (APT) NMR (100 MHz, $CDCl_3$): δ = 153.5, 150.2, 150.2, 145.2, 145.1, 143.6, 143.5, 136.3, 136.2, 136.1, 136.1, 133.8, 133.7, 133.2, 133.1, 132.9, 132.9, 132.1, 132.0, 131.9, 130.3, 129.2, 128.3, 127.3, 127.2, 126.5, 125.9, 125.0, 124.9, 124.0, 124.0, 122.5, 122.5, 122.4, 118.4, 109.8, 34.08, 34.07, 31.10, 21.49, 21.47, 21.05, 21.01.

HRMS (-ESI) m/z: [M-H] Calc. for $C_{44}H_{44}O_5P$: 683.2921, found 683.3001.

IR (KBr) ṽ [cm^{-1}] = 3442, 2958, 2925, 2853, 1771, 1626, 1609, 1485, 1461, 1362, 1246, 1199, 1159, 1096, 1025, 999, 970, 925, 896, 870, 752, 584.

[α_D^{23}] = +52 ° (c = 1.00, $CHCl_3$).

MP = 362 °C.

(*R*)-2,6-Bis(3,5-dimethyl-[1,1'-biphenyl]-4-yl)-4,9-dihydroxydinaphtho-[1,3,2] dioxaphosphepine 4-oxide **SI-29b**:

Prepared according to general procedure Me-deprotection, isolated as an off-white solid with a yield of **96%**.

R_f = 0.31 (Hex/ EtOAc/ MeOH 6:3:1).

^{1}H NMR (400 MHz, $CDCl_3$): δ = 7.97 (d, J = 8.2 Hz, 1H), 7.84 (s, 1H), 7.63 (s, 1H), 7.56 (ddd, J = 8.2, 6.4, 1.5 Hz, 1H), 7.45 – 7.29 (m, 7H), 7.23 (d, J = 2.6 Hz, 1H), 7.20 – 7.11 (m, 4H), 7.06 – 6.99 (m, 2H), 6.96 (dd, J = 9.2, 2.6 Hz, 1H), 6.86 (q, J = 7.4 Hz, 4H), 2.26 (s, 6H), 1.78 (s, 6H).

^{31}P NMR (162 MHz, $CDCl_3$): δ = 5.77.

^{13}C (APT) NMR (100 MHz, $CDCl_3$): δ = 153.6, 145.1, 145.0, 143.5, 143.4, 141.5, 140.8, 140.7, 137.4, 137.4, 137.2, 137.2, 134.7, 134.7, 133.5, 133.4, 133.1, 132.9, 132.8, 132.1, 131.8, 131.7, 130.0, 129.5, 128.5, 128.3, 127.4, 127.3, 127.3, 126.9, 126.7, 126.7, 126.1, 125.9, 125.9, 122.5, 122.4, 118.5, 110.0, 21.32, 21.29, 20.47, 20.44.

HRMS (-ESI) m/z: [M-H] Calc. for $C_{48}H_{36}O_5P$: 723.2295, found 723.2297.

IR (KBr) ṽ [cm^{-1}] = 3426, 3058, 3031, 2922, 1628, 1601, 1567, 1504, 1478, 1445, 1434, 1405, 1378, 1276, 1231, 1210, 1191, 1179, 1150, 1143, 1091, 1025, 998, 970, 898, 877, 855, 842, 825, 764, 713, 698, 654, 608, 592, 580, 511, 481.

[α_D^{23}] = +12° (c = 1.00, $CHCl_3$).

MP = 310 °C.

(*R*)-4,9-Dihydroxy-2,6-bis(2,4,6-tricyclohexylphenyl)dinaphtho-[1,3,2] dioxaphosphepine 4-oxide **SI-29c**:

Prepared according to general procedure Me-deprotection, isolated as an off-white solid with a yield of **99%**.

R_f = 0.66 (Hex/ EtOAc/ MeOH 6:3:1).

^{1}H NMR (400 MHz, $CDCl_3$): δ = 7.85 (d, J = 8.2 Hz, 1H), 7.73 (s, 1H), 7.56 (s, 1H), 7.45 (ddd, J = 8.2, 6.4, 1.5 Hz, 1H), 7.33 – 7.22 (m, 2H), 7.18 – 7.09 (m, 2H), 7.00 – 6.81 (m, 5H), 2.59 – 2.40 (m, 2H), 2.22 – 2.03 (m, 4H), 2.01 – 0.71 (m, 60H).

^{31}P NMR (162 MHz, $CDCl_3$): δ = 1.24.

^{13}C (APT) NMR (100 MHz, $CDCl_3$): δ = 147.2, 146.4, 146.3, 146.1, 144.7, 133.0, 132.4, 132.2, 131.6, 131.5, 131.1, 130.6, 128.9, 128.3, 127.5, 126.9, 126.3, 125.7,

122.5, 121.7, 121.1, 44.82, 42.29, 42.23, 42.09, 41.80, 37.09, 35.10, 34.77, 34.42, 33.51, 33.09, 27.48, 27.26, 27.23, 27.06, 26.72, 26.50, 26.38, 24.97.

HRMS (-ESI) m/z: [M-H] Calc. for $C_{68}H_{80}O_5P$: 1007.5738, found 1007.5815.

IR (KBr) ṽ [cm^{-1}] = 3421, 2925, 2850, 1626, 1605, 1565, 1504, 1448, 1411, 1312, 1281, 1230, 1198, 1188, 1149, 1137, 1118, 1019, 1000, 970, 952, 900, 880, 862, 843, 750, 730, 515.

[α_D^{23}] = -27° (c = 1.00, $CHCl_3$).

MP = 394 °C.

General procedure etherification:

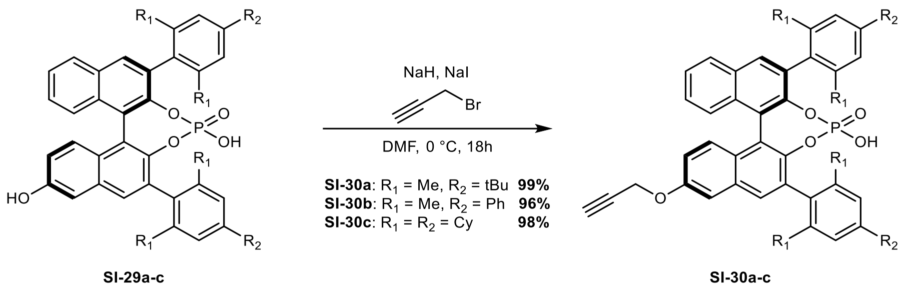

In a flame-dried flask under Ar-atmosphere CPA (**SI-29a-c**) was dissolved in 0.1 M DMF, cooled to 0 °C and 0.1 eq. NaI was added. Then 4.00 eq. NaH (without mineral oil) was added portion wise and the mixture was stirred for two hours at 0 °C. Then 4.0 eq. of a solution of propargyl bromide in toluene (80%wt) was added and the mixture was stirred for another 18 h while warming to ambient temperature. After complete consumption (TLC) the reaction was stopped by addition of water and 2 M HCl at 0 °C. After three times extraction with Et_2O, the organic phase was washed with a 1 M LiCl solution three times and dried by filtration through a phase separator filter paper. The solvent was evaporated and residual DMF was removed by suspending the crude product in heptane followed by removal of the solvent under reduced pressure (repeated until DMF was completely removed). The catalyst precursors were typically obtained as off-white solids and used in the Click-reaction without further purification.

(*R*)-2,6-Bis(4-(*tert*-butyl)-2,6-dimethylphenyl)-4-hydroxy-9-(prop-2-yn-1-yloxy) dinaphtho-[1,3,2] dioxaphosphepine 4-oxide **SI-30a**:

Prepared according to general procedure etherification, isolated as an off-white solid with a yield of **99%**.

R_f = 0.40 (Hex/ EtOAc/ MeOH 6:3:1).

^{1}H NMR (400 MHz, $CDCl_3$): δ = 9.74 (s, 1H), 7.90 (d, J = 8.2 Hz, 1H), 7.78 (s, 1H), 7.69 (s, 1H), 7.50 (ddd, J = 8.2, 6.6, 1.4 Hz, 1H), 7.41 – 7.28 (m, 4H), 7.04 (dd, J = 9.4, 2.6 Hz, 1H), 6.97 – 6.93 (m, 4H), 4.85 (t, J = 2.4 Hz, 2H), 2.58 (t, J = 2.4 Hz, 1H), 2.20 (s, 3H), 2.18 (s, 3H), 2.02 (s, 6H), 0.97 (s, 9H), 0.96 (s, 9H).

^{31}P NMR (162 MHz, $CDCl_3$): δ = 6.18.

^{13}C (APT) NMR (100 MHz, $CDCl_3$): δ = 155.4, 150.2, 136.2, 133.8, 133.2, 132.8, 132.2, 132.1, 130.8, 129.0, 128.3, 127.8, 127.2, 126.4, 125.8, 124.9, 124.0, 122.5, 119.1, 107.8, 75.98, 56.10, 34.09, 31.12, 21.49, 21.01.

HRMS (+ESI) m/z: [M+H] Calc. for $C_{47}H_{48}O_5P$: 723.3234, found 723.3263.

IR (KBr) ṽ [cm^{-1}] = 3456, 3293, 3056, 2962, 2866, 1666, 1627, 1598, 1504, 1463, 1437, 1361, 1303, 1269, 1243, 1221, 1198, 1155, 1119, 1021, 999, 968, 924, 896, 870, 847, 752, 711, 673, 584, 508.

[α_D^{23}] = -36 ° (c = 1.00, $CHCl_3$).

MP = decomposes >280 °C.

(*R*)-2,6-Bis(3,5-dimethyl-[1,1'-biphenyl]-4-yl)-4-hydroxy-9-(prop-2-yn-1-yloxy) dinaphtho-[1,3,2] dioxaphosphepine 4-oxide **SI-30b**:

Prepared according to general procedure etherification, isolated as an off-white solid with a yield of **96%**.

R_f = 0.35 (Hex/ EtOAc/ MeOH 6:3:1).

^{1}H NMR (400 MHz, DMSO-d_6): δ = 8.11 (d, J = 8.1 Hz, 1H), 8.00 (s, 1H), 7.86 (s, 1H), 7.73 (d, J = 7.7 Hz, 4H), 7.60 (d, J = 2.3 Hz, 1H), 7.56 (ddd, J = 8.1, 6.8, 1.1 Hz, 1H), 7.50 – 7.30 (m, 12H), 7.25 (d, J = 8.6 Hz, 1H), 7.18 – 7.09 (m, 2H), 4.95 (d, J = 2.4 Hz, 2H), 3.64 (t, J = 2.4 Hz, 1H), 2.30 (s, 3H), 2.29 (s, 3H), 2.05 (s, 6H).

^{31}P NMR (162 MHz, DMSO-d_6): δ = 1.69.

^{13}C (APT) NMR (100 MHz, DMSO-d_6): δ = 154.9, 145.8, 145.7, 144.3, 144.2, 140.1, 140.0, 139.2, 139.1, 137.6, 137.5, 137.0, 137.0, 136.0, 136.0, 132.8, 132.7, 132.2, 132.1, 131.5, 131.2, 131., 129.9, 128.9, 128.6, 127.6, 127.3, 127.0, 126.9, 126.6,

126.6, 125.9, 125.6, 125.0, 121.9, 121.9, 121.9, 121.8, 119.3, 108.4, 79.07, 78.51, 55.66, 21.13, 20.08, 20.05.

HRMS (-ESI) m/z: [M-H] Calc. for $C_{51}H_{38}O_5P$: 761.2462, found 761.2471.

IR (KBr) ṽ [cm^{-1}] = 3434, 3293, 2922, 1661, 1626, 1600, 1503, 1478, 1436, 1417, 1400, 1380, 1317, 1218, 1193, 1181, 1151, 1142, 1132, 1091, 1025, 998, 965, 894, 875, 836, 765, 755, 712, 699, 580.

[α_D^{23}] = -21° (c = 1.00, $CHCl_3$).

MP = 388 °C.

(*R*)-4-Hydroxy-9-(prop-2-yn-1-yloxy)-2,6-bis(2,4,6-tricyclohexylphenyl)dinaphtho-[1,3,2] dioxaphosphepine 4-oxide **SI-30c**:

Prepared according to general procedure etherification, isolated as a white solid with a yield of **98%**.

R_f = 0.74 (Hex/ EtOAc/ MeOH 6:3:1).

^{1}H NMR (400 MHz, DMSO-d_6): δ = 7.97 (d, J = 8.2 Hz, 1H), 7.74 (s, 1H), 7.60 (s, 1H), 7.46 (s, 1H), 7.40 (t, J = 7.5 Hz, 1H), 7.29 (t, J = 7.5 Hz, 1H), 7.12 (d, J = 8.5 Hz, 1H), 7.09 – 6.97 (m, 4H), 6.94 (s, 2H), 4.96 – 4.75 (m, 2H), 3.57 (t, J = 2.3 Hz, 1H), 2.17 – 1.91 (m, 6H), 1.92 – 0.90 (m, 60H).

^{31}P NMR (162 MHz, DMSO-d_6): δ = 2.48.

^{13}C (APT) NMR (100 MHz, DMSO-d_6): δ = 154.0, 146.5, 146.4, 146.1, 145.5, 145.5, 133.4, 132.0, 130.7, 129.7, 128.1, 127.5, 127.1, 125.7, 125.4, 121.9, 121.7, 120.7, 108.2, 103.0, 79.17, 78.24, 78.01, 62.93, 55.52, 44.15, 44.10, 41.14, 40.83, 36.33, 34.80, 34.13, 34.11, 34.08, 32.49, 32.44, 32.12, 28.99, 27.02, 26.82, 26.76, 26.51, 25.97, 25.88, 25.69, 18.38.

HRMS (-ESI) m/z: [M-H] Calc. for $C_{71}H_{82}O_5P$: 1045.5894, found 1045.5951.

IR (KBr) ṽ [cm^{-1}] = 3420, 3312, 2925, 2850, 1626, 1606, 1500, 1447, 1420, 1260, 1233, 1218, 1200, 1100, 1030, 1001, 972, 862, 837, 750.

[α_D^{23}] = -14° (c = 1.00, $CHCl_3$).

MP = 154 °C.

1.4 Preparation and characterization of solid supports

The parent silica material MB100-75/200 (A(BET) = 290 m^2/g; V(P) = 0.85 cm^3/g) was purchased from Fuji Silysia. This silica has spherical particles (particle size fraction from 75 to 200 µm) with a pore width of 100 Å. The 100 to 150 µm fraction was separated by sieving and only this fraction was used in the following steps (for textural properties from nitrogen sorption isotherms of the material see 1.5).

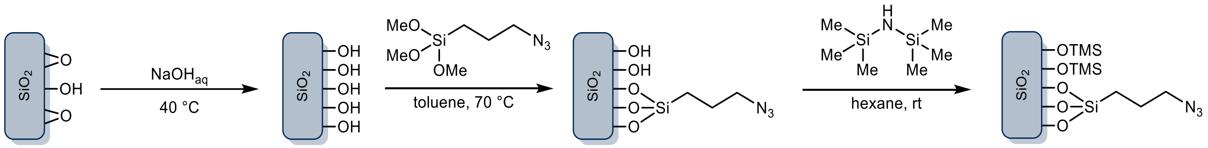

Alkaline treatment:

Since commercial silica materials are typically calcined at high temperatures, the silanol group density of the material was increased by treatment with NaOH as described in the literature.[5] For this 1.00 g of the silica was stirred in 30 ml of a 0.5 mM $NaOH_{aq}$ for four hours. Afterwards the material was filtered and washed with 20 ml water, ethanol and dichloromethane and dried under reduced pressure.

Azide functionalization:

To a solution of 1.00 ml (1.09 g, 5.32 mmol) azidosilane in 30 ml (0.18 M) toluene was added 1.00 g silica under argon atmosphere and the suspension was stirred at 70 °C for 24 h. Subsequently the mixture was filtered, washed with 20 ml toluene, ethanol and dichloromethane and dried under reduced pressure. The material was characterized by CHN-elemental analysis, TGA and nitrogen sorption.

f_{azide} = 0.51 mmol/g (TGA, CHN-elemental analysis).

IR (KBr) ṽ [cm^{-1}] = 2125 (N_3).

End-capping:

To a solution of 2.00 ml (1.54 g, 9.54 mmol) HMDS in 30 ml (~ 0.3 M) hexane was added 1.00 g of the azide-functionalized silica under argon atmosphere and the suspension was stirred for 24h at ambient temperature. The mixture was filtered, washed with 20 ml hexane, ethanol and dichloromethane and dried under reduced pressure. The material was characterized by solid state MAS-NMR, CHN-elemental analysis, TGA and nitrogen sorption.

Characterization:

Solid-state-MAS-NMR:

Supplementary figure SF2: ^{1}H solid state NMR spectrum azido functionalized silica:

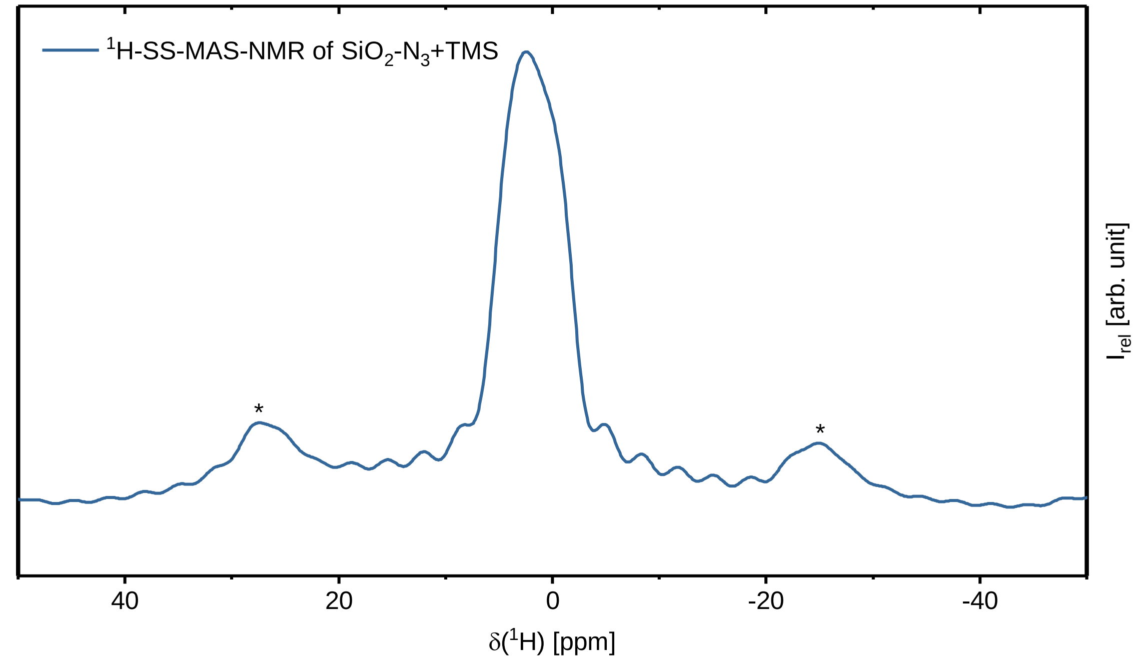

Supplementary figure SF3: ^{29}Si solid state NMR spectrum azido functionalized silica:

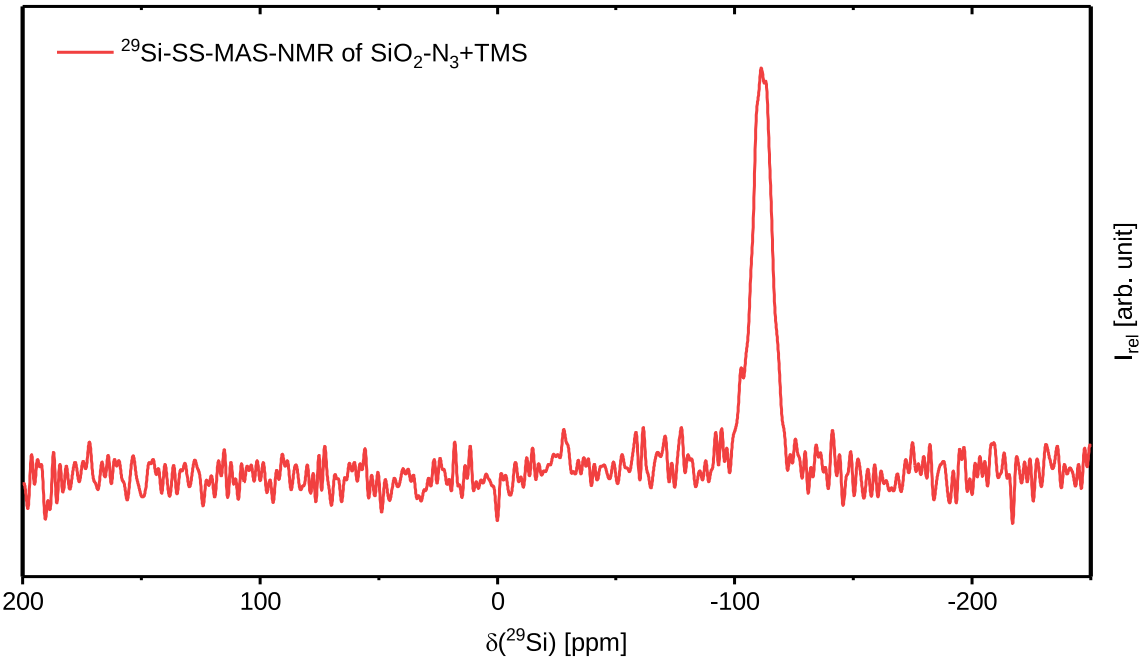

Supplementary figure SF4: Nitrogen sorption of azido functionalized silica (red):

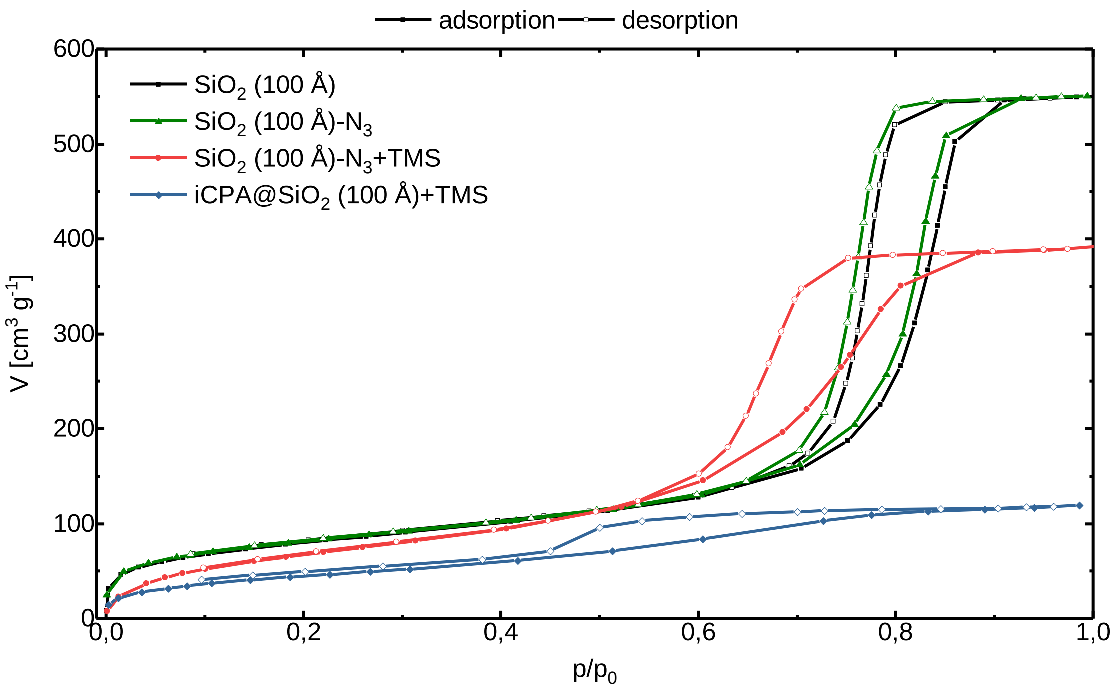

Azido+TMS-SiO2 (red): A(BET) = 267 m^2/g; V(P) = 0.70 cm^3/g

Supplementary figure SF5: Pore width distribution of azido functionalized silica (from BJH model) (red)

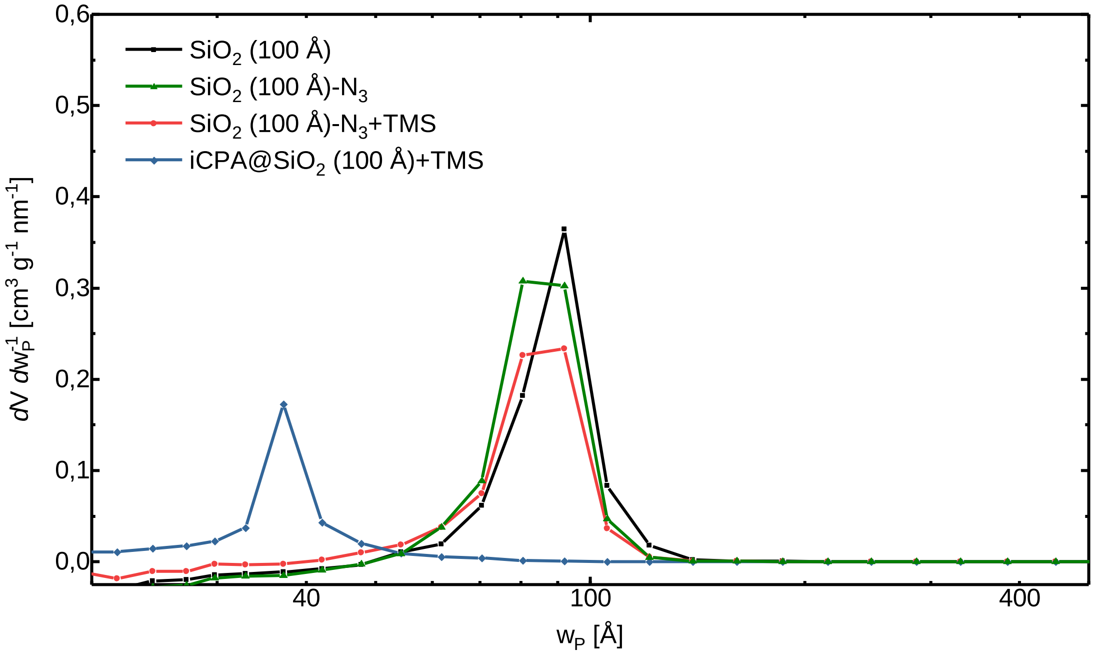

Supplementary figure SF6: TGA of azido functionalized silica (red):

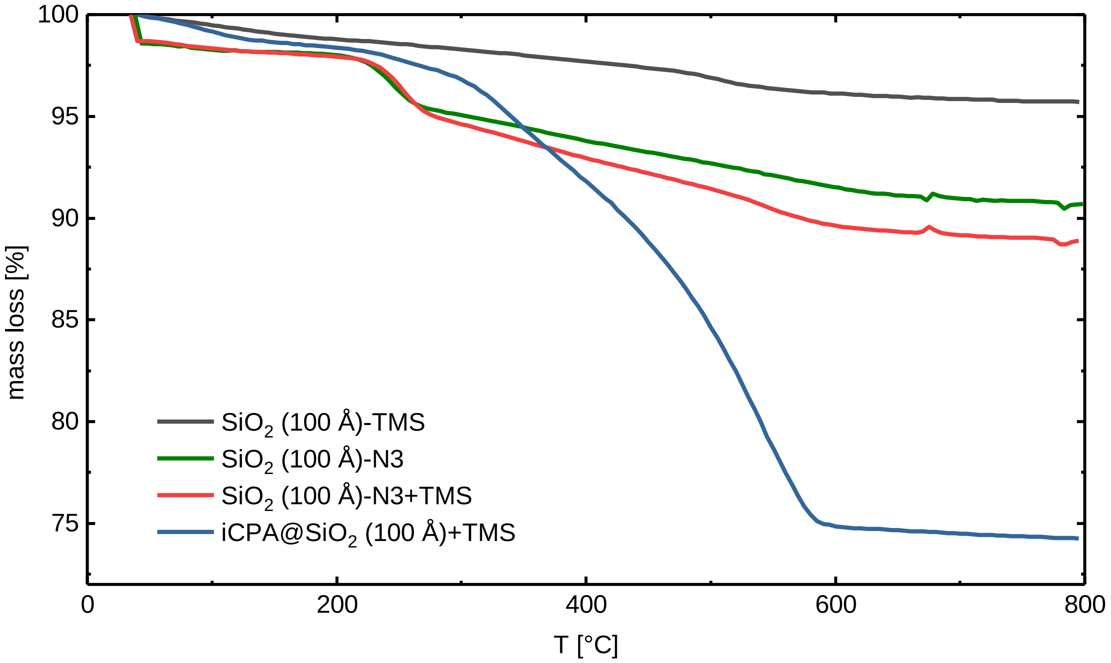

1.5 Synthesis and Characterization of immobilized Catalysts

Synthesis of molecular catalyst analogue:

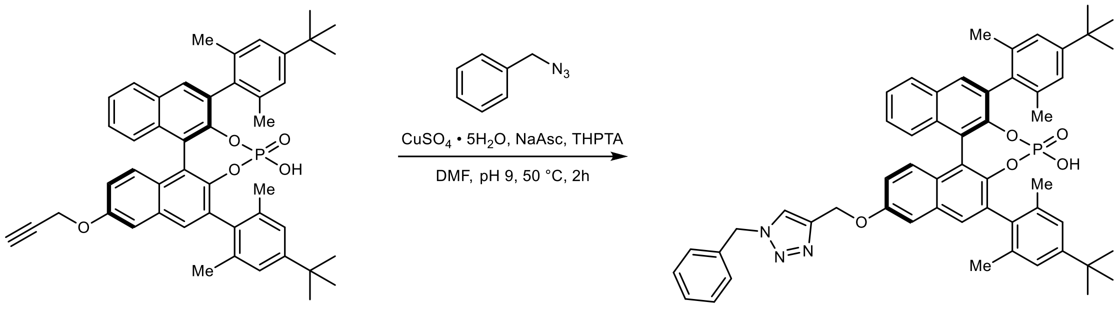

SI-30a **SI-3b**

An aqueous $CuSO_4$ solution (0.15 mmol, 0.3 eq., 0.1 M) was mixed with the aqueous tris(3-hydroxypropyltriazolylmethyl)amine (THPTA) solution (0.15 mmol, 0.3 eq., 0.1 M) and aqueous NaAsc solution (0.20 mmol, 0.4 eq., 1.0 M) and adjusted to pH 9 with 1 M NaOH. Then incubation was performed at 50 °C for 15 min, followed by degassing for another 15 min.

A solution of 361.4 mg (0.50 mmol, 1.00 eq.) CPA **SI-30a** in 5 ml DMF (0.1 M) was prepared and degassed with argon (30 minutes).

Then the Cu(I) mix was added to 99.9 mg (0.75 mmol, 1.50 eq.) azide (under Ar) and the solution of CPA **SI-30a** was added. The reaction mixture was shaken (360 rpm) at 50 °C for two hours.

It was extracted 3x with CH_2Cl_2 and the organic phase was washed several times (> 5x) with 1 M LiCl solution. Subsequently, it was washed with saturated aqueous Na_2EDTA solution, 1 M HCl and H_2O, and the organic phase was dried by filtration through a phase separator filter paper. The solvent was removed under reduced pressure and the product was obtained as a light-brown solid (if DMF is still in the product: removed through azeotrope formation with heptane under reduced pressure) with a yield of **78%**.

$\mathbf{R_f}$ = 0.28 (Hex/ EtOAc/ MeOH 6:3:1).

^{1}H NMR (400 MHz, $CDCl_3$): δ = 7.87 (d, J = 8.2 Hz, 1H), 7.74 (s, 1H), 7.63 (s, 1H), 7.58 (s, 1H), 7.47 (t, J = 7.6 Hz, 1H), 7.41 – 7.23 (m, 9H), 7.02 – 6.86 (m, 5H), 5.55 (s, 2H), 5.33 – 5.23 (m, 2H), 2.12 (s, 6H), 1.98 (s, 6H), 1.03 (s, 18H).

^{31}P NMR (162 MHz, $CDCl_3$): δ = 6.81.

^{13}C (APT) NMR (100 MHz, $CDCl_3$): δ = 156.0, 150.0, 144.6, 134.5, 133.8, 133.2, 132.8, 132.2, 131.8, 131.7, 130.6, 129.3, 129.2, 129.0, 129.0, 128.9, 128.5, 128.4, 128.3, 127.7, 127.2, 126.3, 125.6, 124.9, 124.0, 122.7, 122.5, 119.0, 107.6, 62.29, 54.48, 34.17, 31.23, 21.46, 20.93.

HRMS (+ESI) m/z: [M-H] Calc. for $C_{54}H_{53}N_3O_5P$: 854.3717, found 854.3727.

IR (KBr) ṽ [cm^{-1}] = 3434, 2962, 2925, 2867, 1626, 1606, 1499, 1456, 1436, 1362, 1245, 1224, 1200, 1119, 1046, 1015, 998, 970, 869, 752, 718, 710, 583.

[α_D^{23}] = -56° (c = 0.50, $CHCl_3$).

MP = 302 °C.

General procedure Click-reaction:

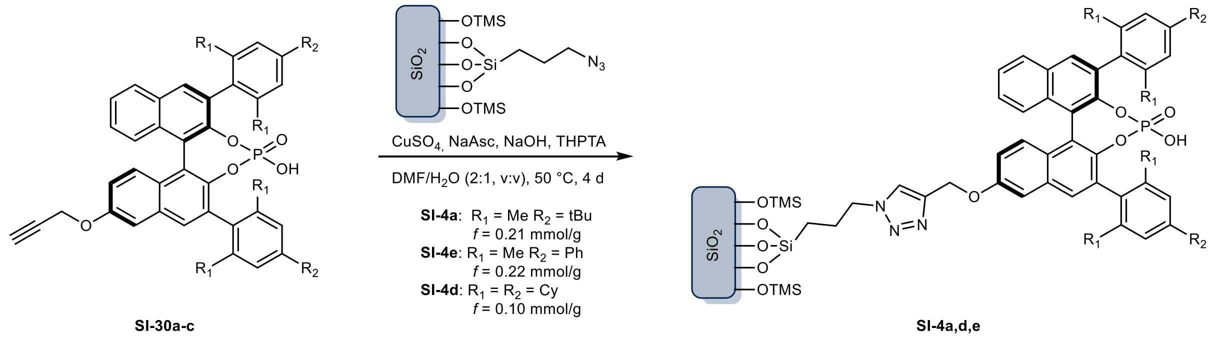

An aqueous $CuSO_4$ solution (0.90 mmol, 0.3 eq., 0.1 M) was mixed with the aqueous THPTA solution (0.90 mmol, 0.3 eq., 0.1 M) and aqueous NaAsc solution (1.20 mmol, 0.4 eq., 1.0 M) and adjusted to pH 9 with 1 M NaOH. Then incubation was performed at 50 °C for 15 min, followed by degassing for another 15 min.

A solution of 3.00 mmol (1.00 eq.) CPA **SI-30a-c** in 30 ml DMF (0.1 M) was prepared and degassed with argon (30 minutes).

Then the Cu(I) mix was added to a flask with 3.00 g of the azide-functionalized solid support (under Ar) and the solution of CPA **SI-30a-c** was added. The reaction mixture was shaken (360 rpm) at 50 °C for three days. Then another 30 ml DMF was added and the mixture was shaken (360 rpm) at 50 °C for one more day.

The mixture was filtered, washed with DMF, then air was suctioned through the solid material (to oxidize copper for EDTA treatment) for two hours, before it was washed with saturated aqueous Na_2EDTA solution, 1 M HCl, H_2O, acetone, THF and CH_2Cl_2. Solvent residues were evaporated under reduced pressure to give the immobilized iCPAs **SI-4a-e** as a light-brown solid.

General procedure Click-reaction-PS:

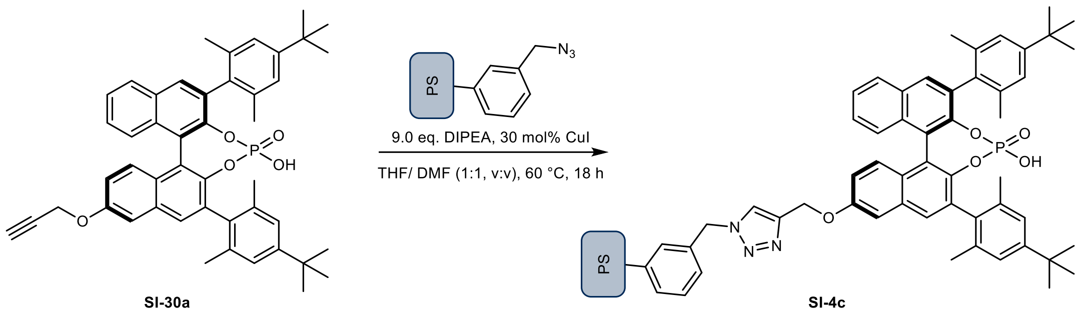

Following a procedure from Pericas *et al.*[15]:

500 mg (f_N = 1.25 mmol/g) of Azido-functionalized Polystyrene were suspended in 15 ml of a dry THF/DMF mixture (1:1 $^v/_v$). Then 500 mg (0.69 mmol) of **SI-30a**, 9.0 eq. DIPEA and 30 mol% CuI were added. The mixture was shaken at 60 °C for 18 h and cooled to rt afterwards.

The mixture was filtered and washed with DMF, EDTA, 1 M HCl, H_2O, THF and MeOH. The immobilized catalyst was reacidified by stirring with 2 M HCl in EtOAc for 10 mins and then washed with EtOAc and CH_2Cl_2.

Solvent residues were evaporated under reduced pressure to give the immobilized iCPA **SI-4c** as a light-brown solid.

Characterization of immobilized Catalysts:

iCPA@SiO2 **SI-4a**:

Synthesized after general procedure Click-reaction and isolated as a light-brown solid. The catalyst loading was determined by ICP-OES (phosphorus-content) *f* = 0.20 mmol/g, CHN elemental analysis *f* = 0.21 mmol/g and thermogravimetric analysis *f* = 0.21 mmol/g. After a total runtime of 109 h in the Transfer Hydrogenation the catalyst loading was again determined by CHN-EA (f_{used} = 0.20 mmol/g) showing the stability of the iCPA.

Supplementary figure SF7: ^{1}H Solid-state (SS)-MAS-NMR of **SI-4a**:

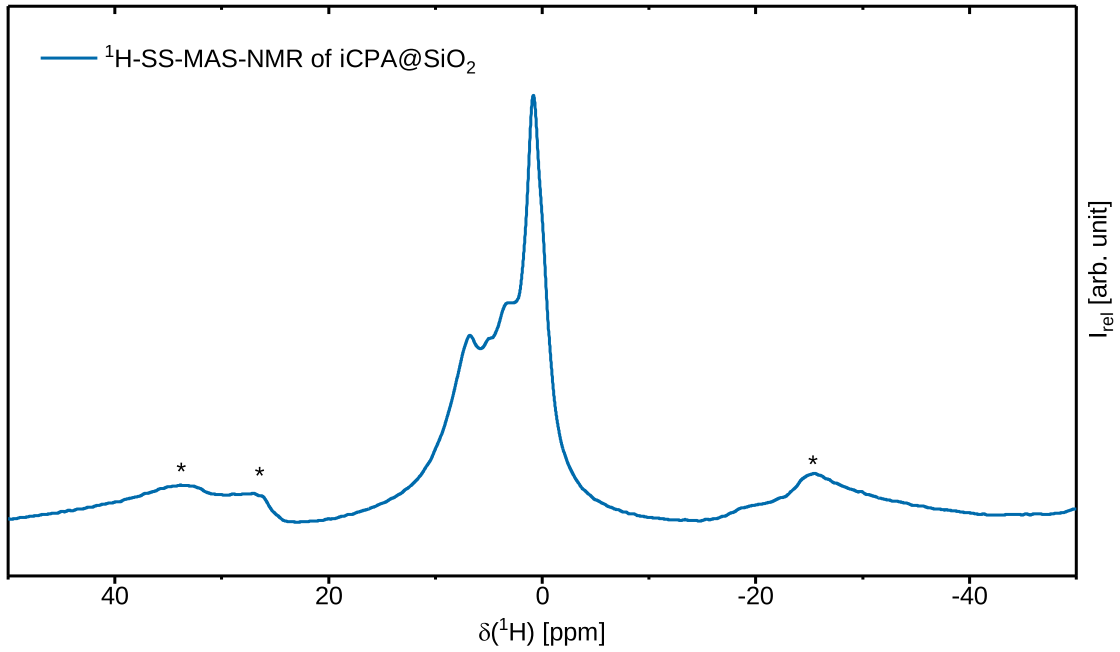

Supplementary figure SF8: ^{13}C Solid-state (SS)-MAS-NMR of **SI-4a**:

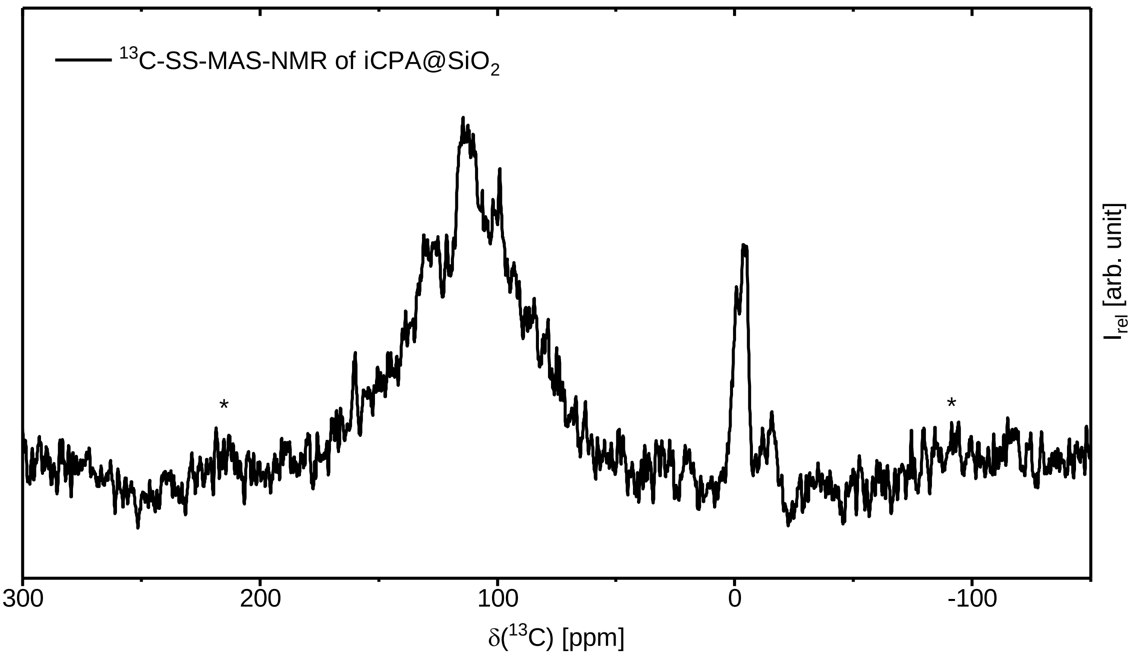

Supplementary figure SF9: ^{29}S Solid-state (SS)-MAS-NMR of **SI-4a**:

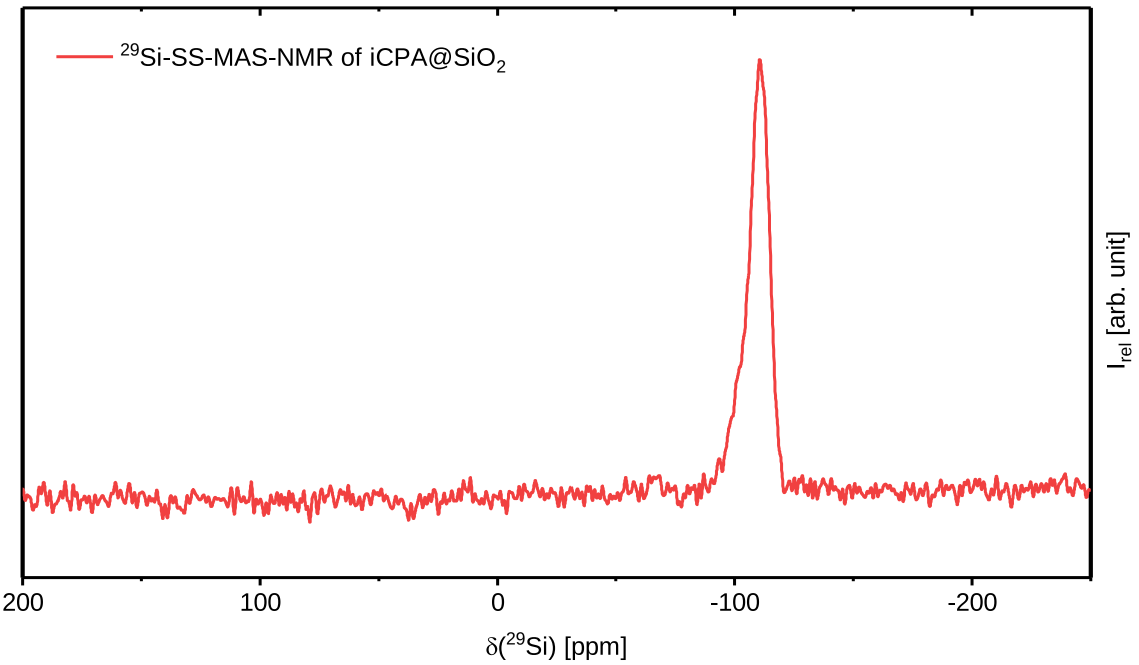

Supplementary figure SF10: ^{31}P Solid-state (SS)-MAS-NMR of **SI-4a**:

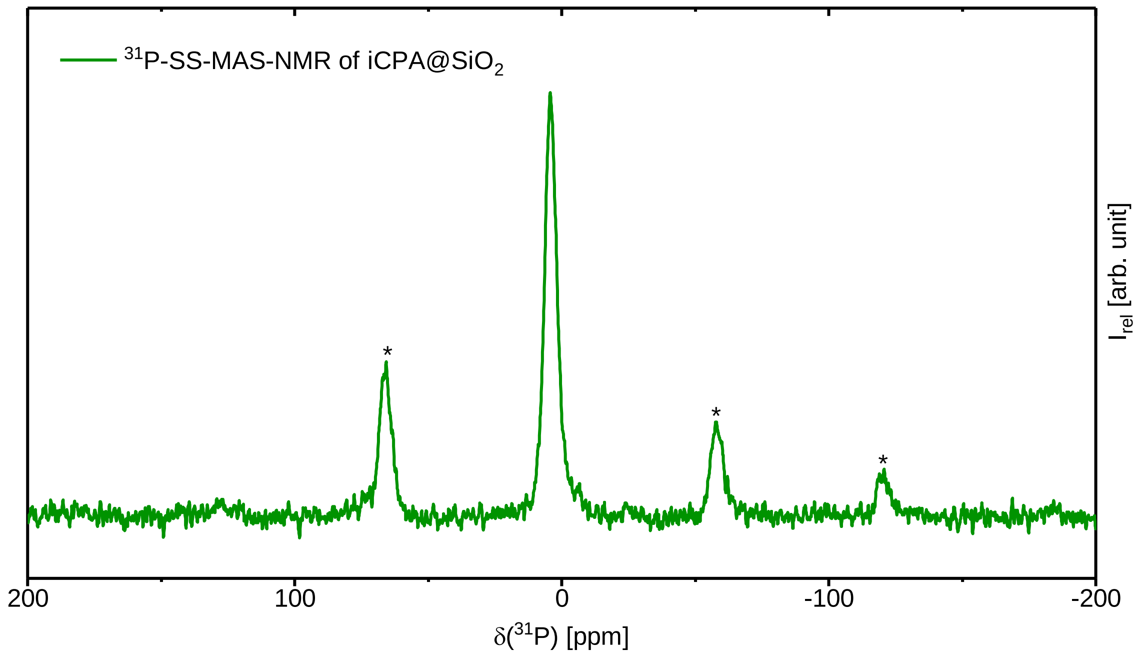

Supplementary figure SF11: ^{31}P Solid-state (SS)-MAS-NMR of **SI-4a** after 109 h of usage:

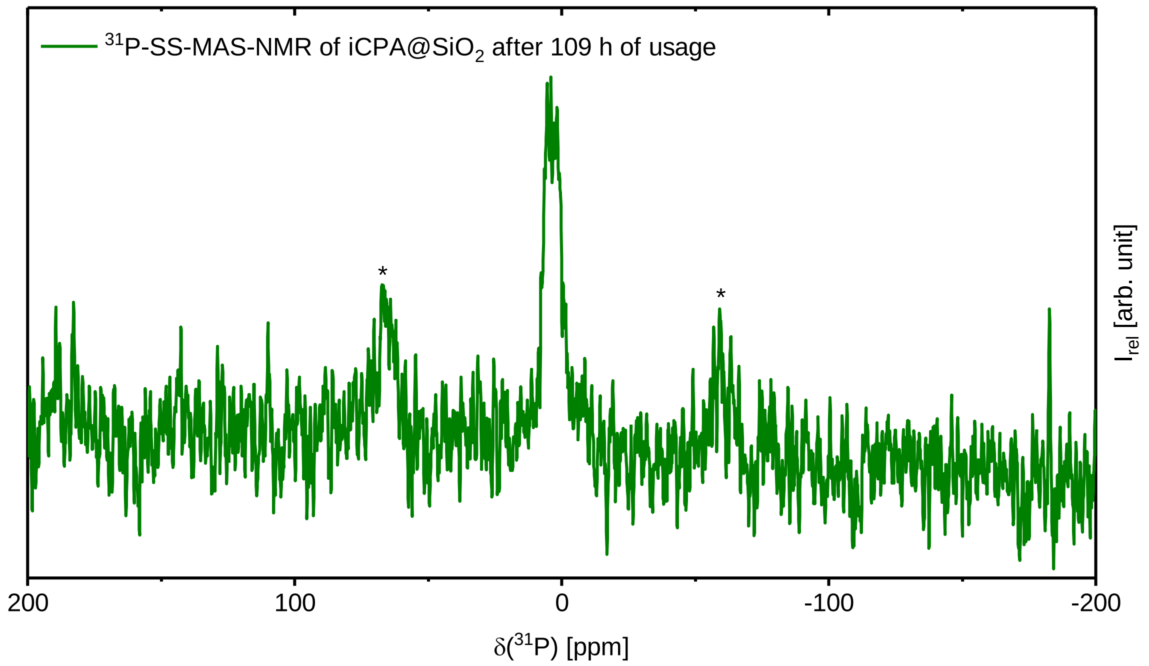

Supplementary figure SF12: Nitrogen sorption of **SI-4a** (blue):

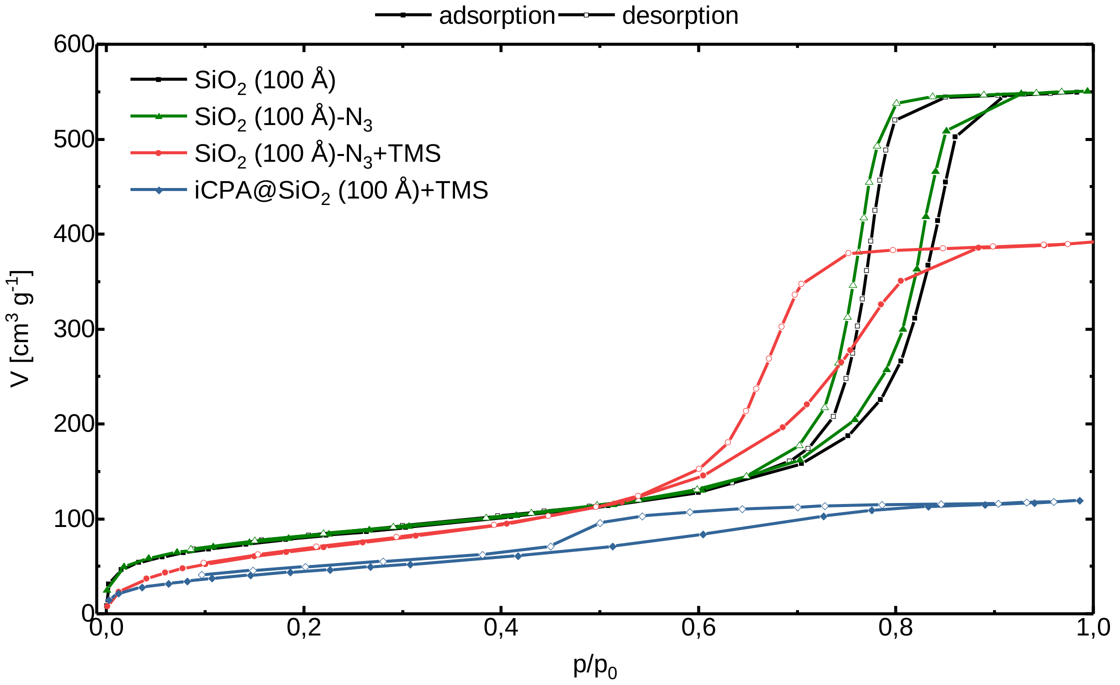

SI-4a (blue): A(BET) = 166 m^2/g; V(P) = 0.19 cm^3/g

Supplementary figure SF13: Pore width distribution (from BJH model) of **SI-4a** (blue)

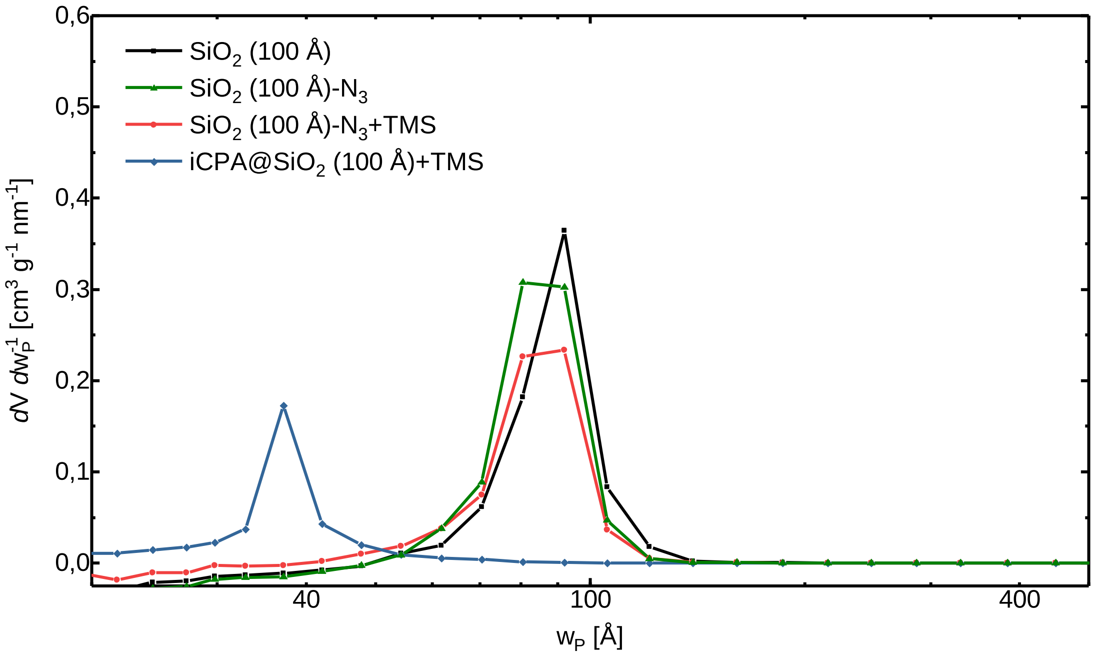

Supplementary figure SF14: TGA of **SI-4a** (blue):

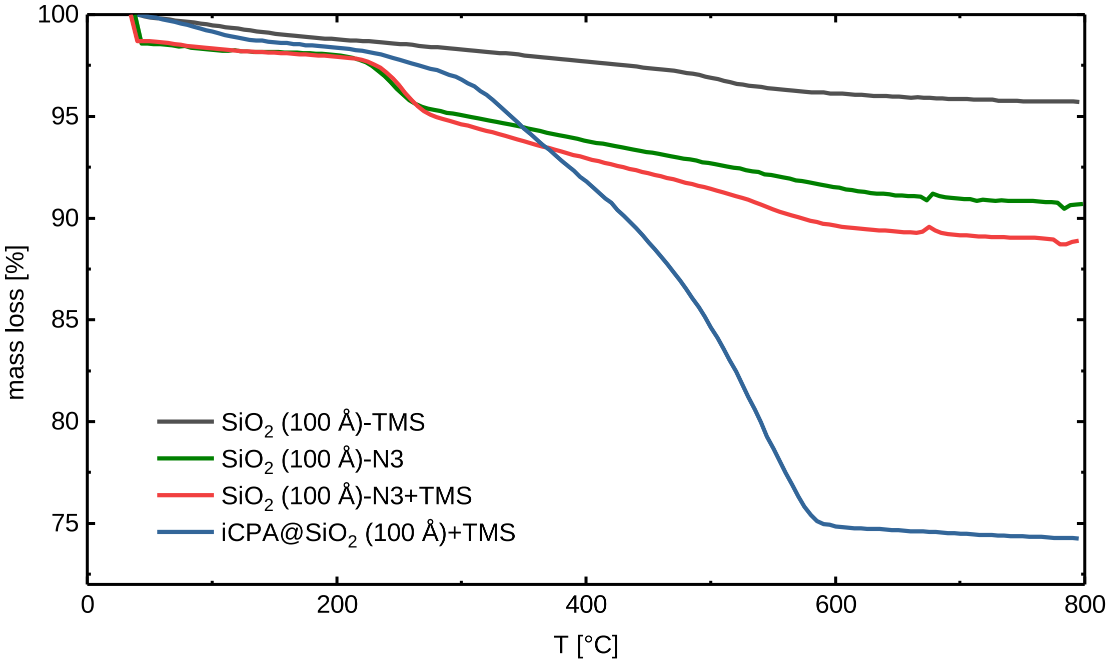

STEM:

The sample was mortared in ethanol for 1 min, after 5 min of segregation a droplet with dispersed particles was supported on a lacey carbon on TEM grid.

Supplementary figure SF15 a): STEM image of **SI-4a**

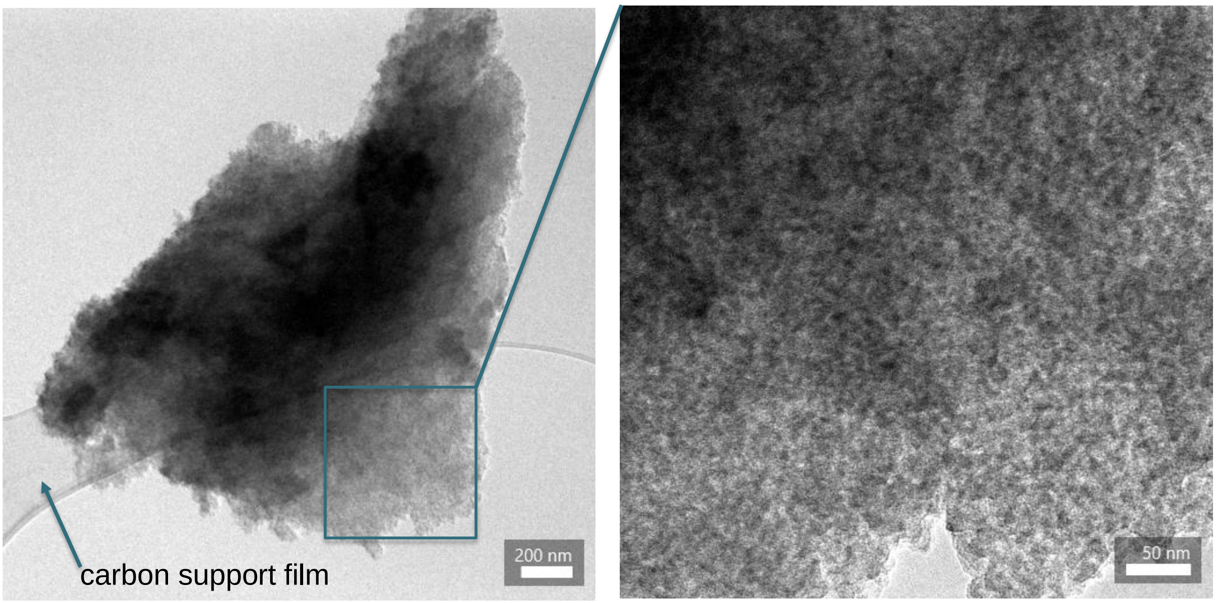

Supplementary figure SF15 b): STEM-EDX mapping with elemental distribution of **SI-4a**:

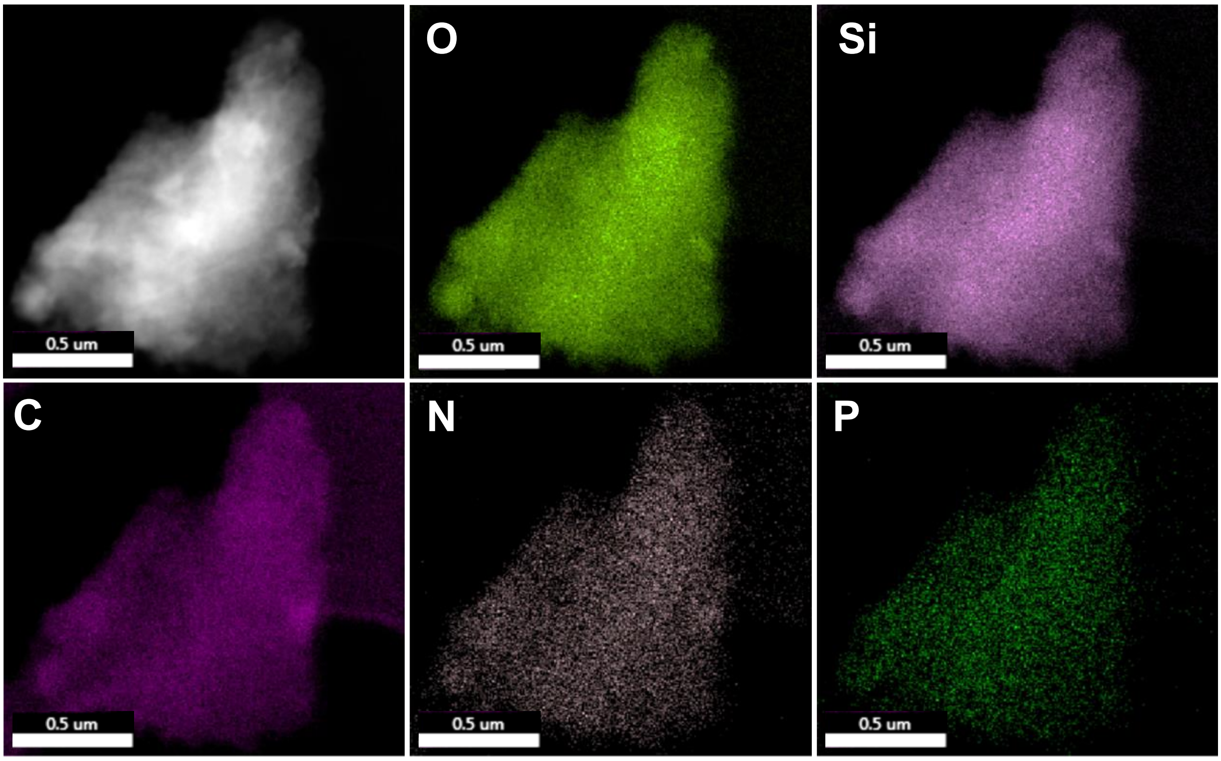

iCPA@ALOX **SI-4b**:

Synthesized after general procedure Click-reaction, instead of silica 3.00g of azide functionalized ALOX were used. The immobilized catalyst was isolated as a light-brown solid. The catalyst loading was determined by ICP-OES (phosphorus-content) *f* = 0.14 mmol/g.

Supplementary figure SF16: ^{1}H Solid-state-MAS-NMR of **SI-4b**:

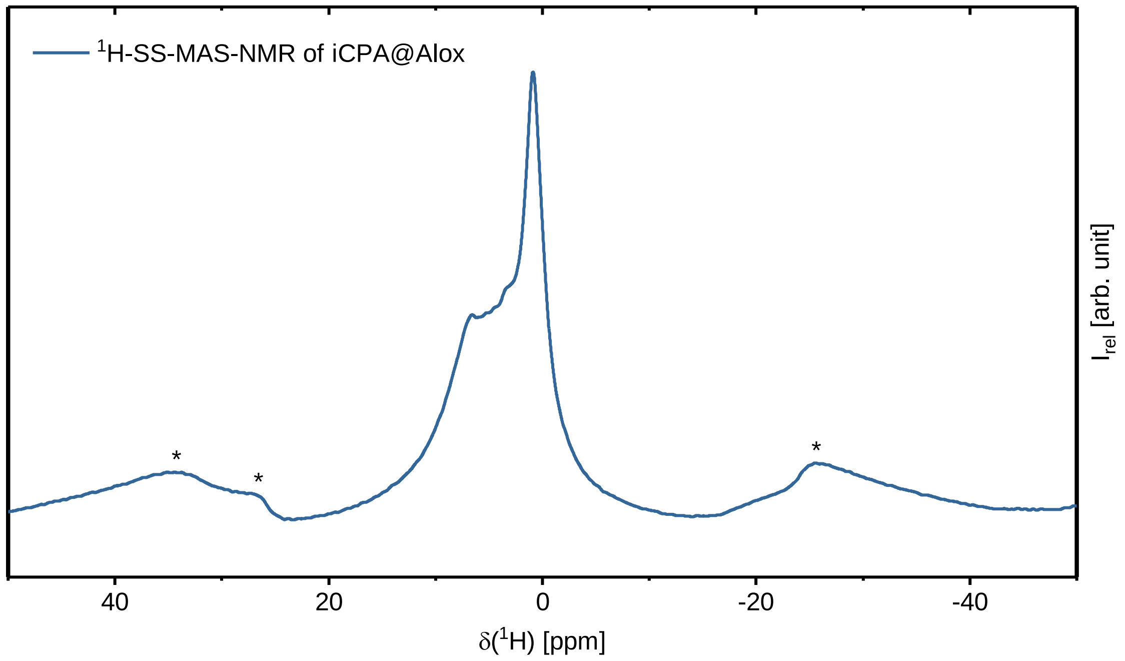

Supplementary figure SF17: ^{13}C Solid-state-MAS-NMR of **SI-4b**:

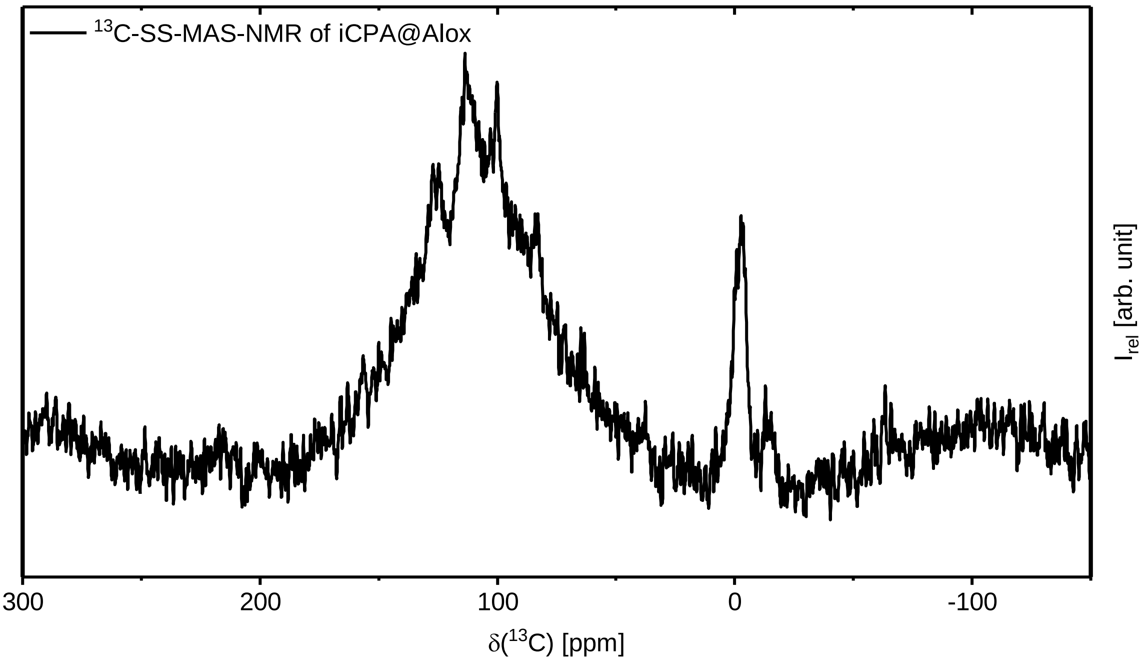

Supplementary figure SF18: ^{27}Al Solid-state-MAS-NMR of **SI-4b**:

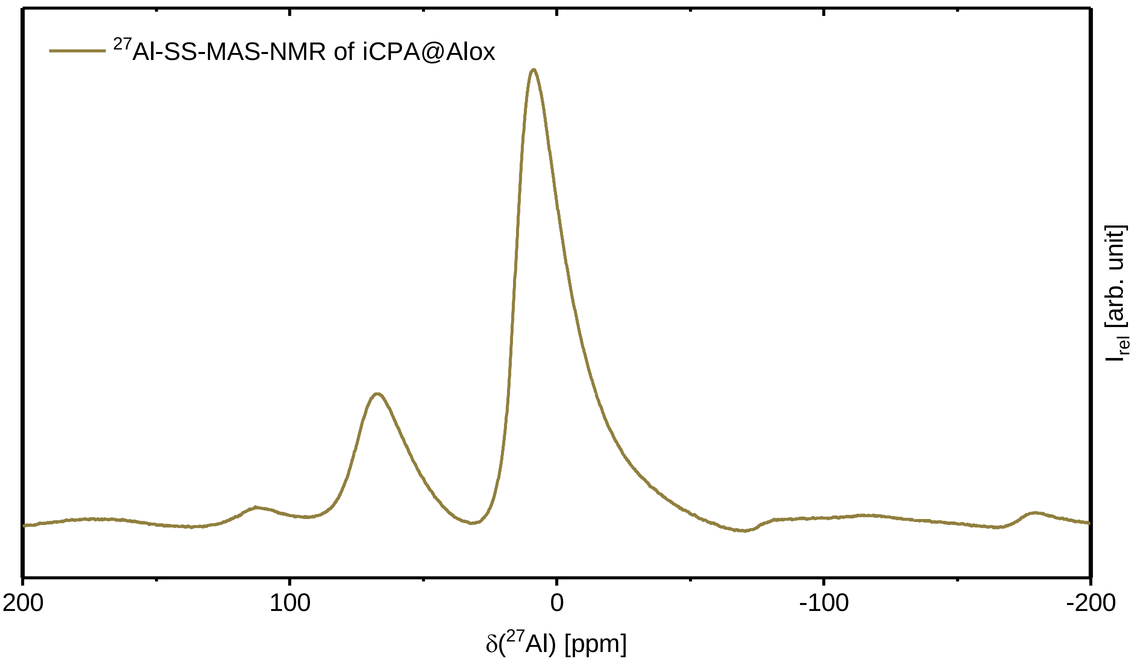

Supplementary figure SF19: ^{31}P Solid-state-MAS-NMR of **SI-4b**:

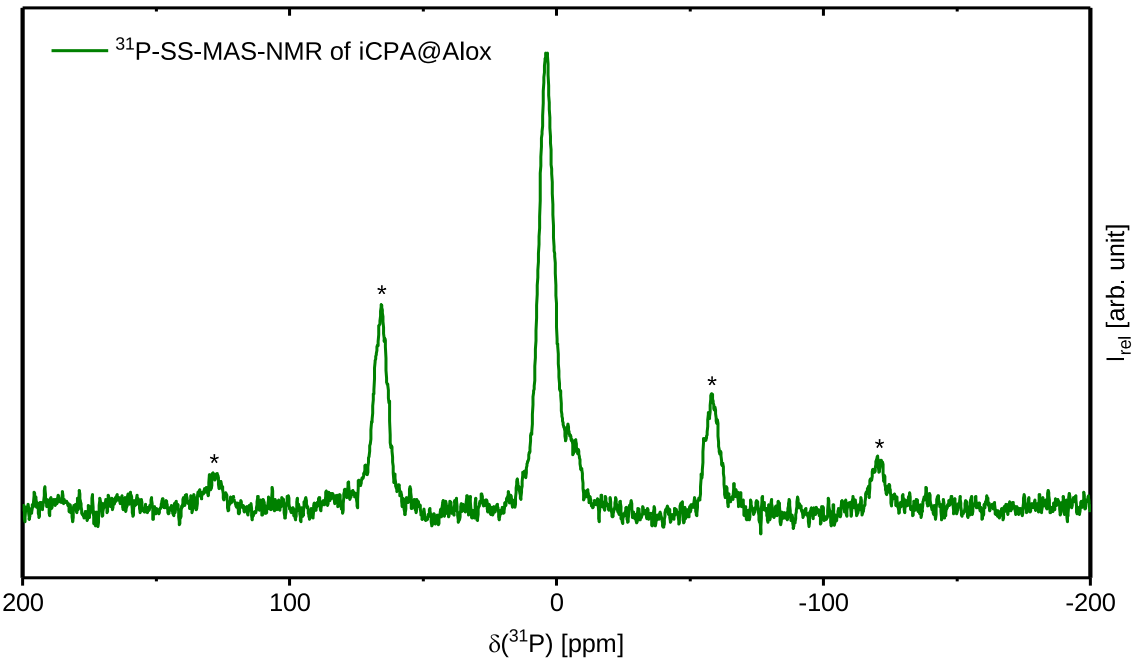

iCPA@PS **SI-4c**:

Synthesized after general procedure Click-reaction-PS. The immobilized catalyst was isolated as a light-brown solid. The catalyst loading was determined by ICP-OES (phosphorus-content) *f* = 0.54 mmol/g and CHN elemental analysis *f* = 0.51 mmol/g.

Supplementary figure SF20: ^{31}P Solid-state-MAS-NMR of **SI-4c**:

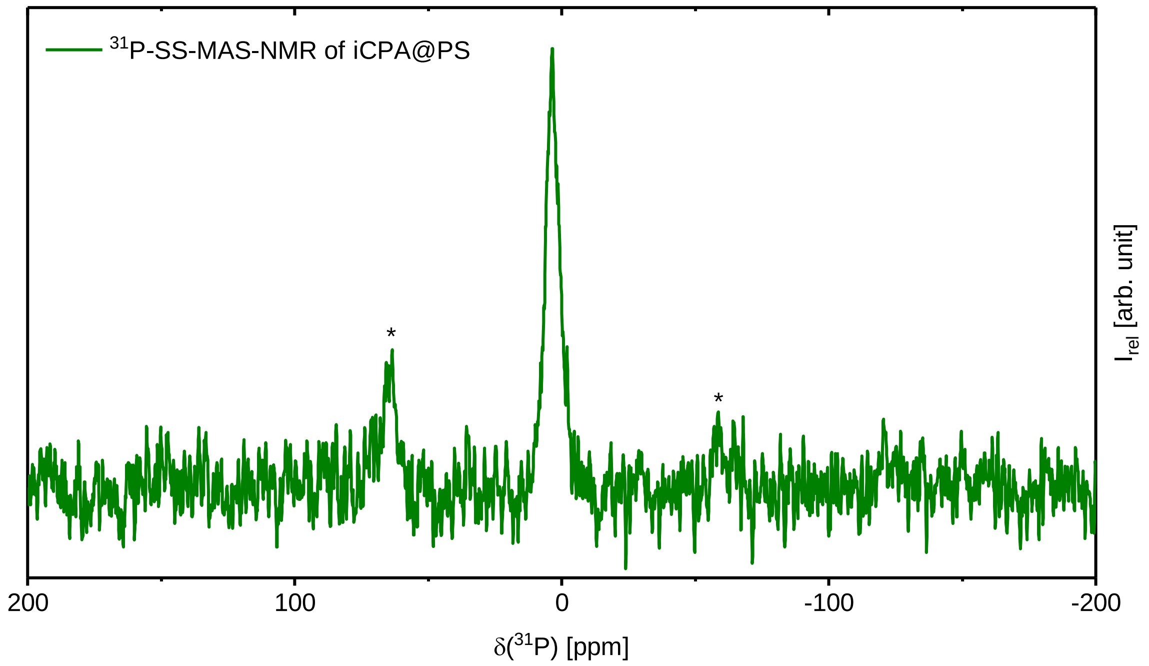

iCPA@SiO2 **SI-4e**:

Synthesized after general procedure Click-reaction and isolated as a light-brown solid. The catalyst loading was determined by ICP-OES (phosphorus-content) *f* = 0.21 mmol/g, CHN elemental analysis *f* = 0.23 mmol/g and thermogravimetric analysis *f* = 0.23 mmol/g.

Supplementary figure SF21: ^{1}H Solid-state-MAS-NMR of **SI-4e**:

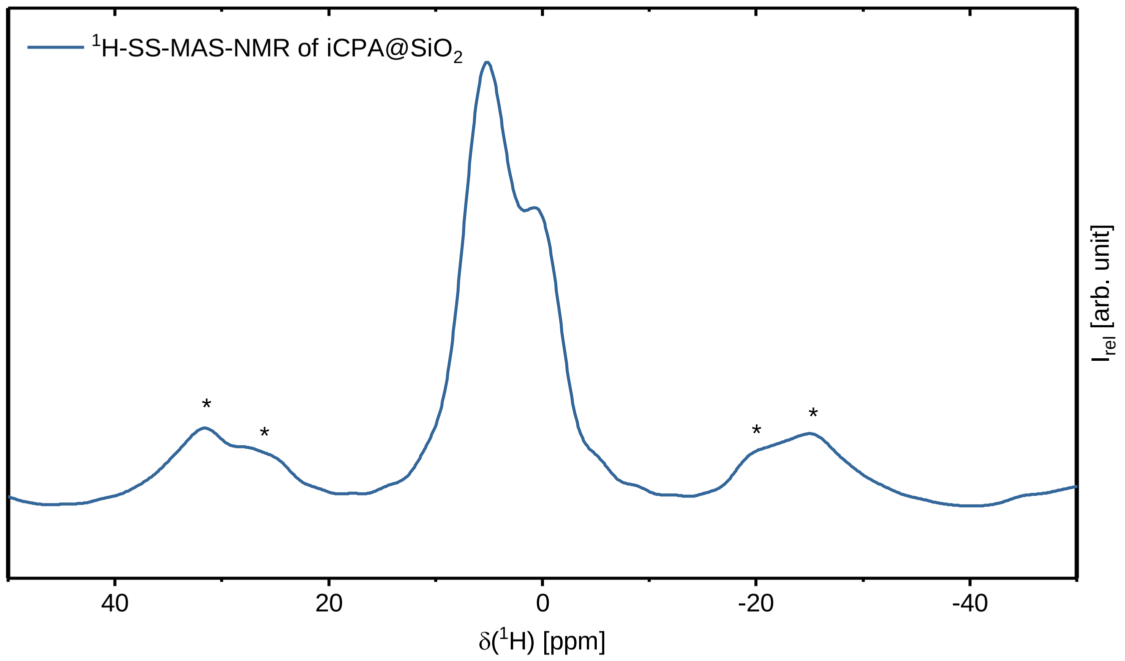

Supplementary figure SF22: ^{31}P Solid-state-MAS-NMR of **SI-4e**:

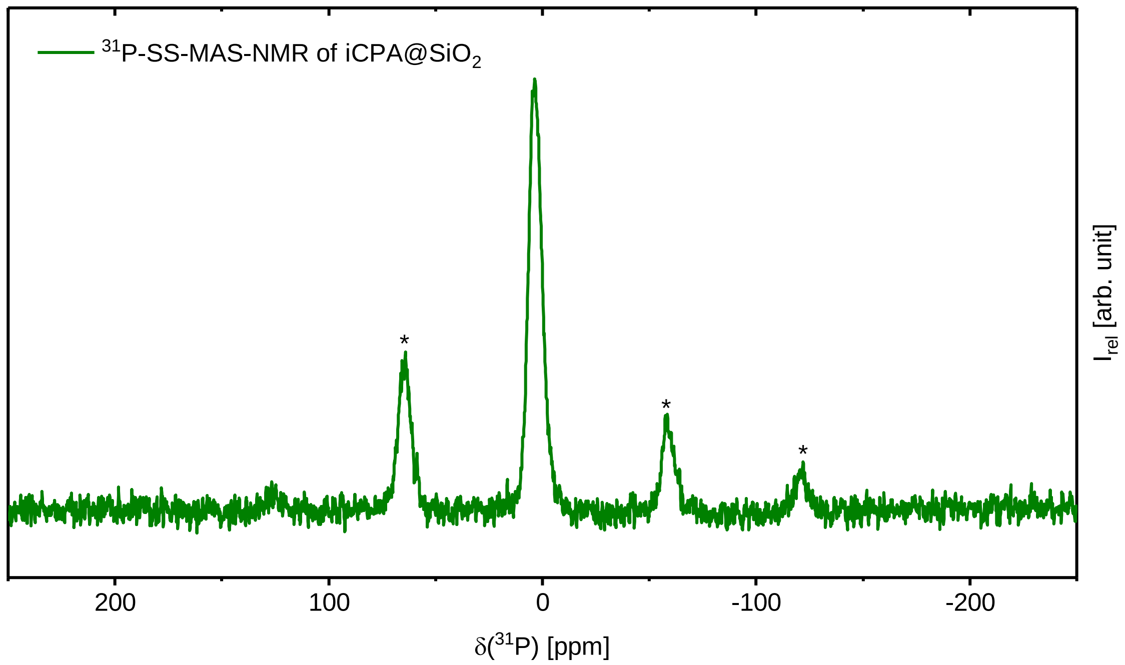

Supplementary figure SF23: Nitrogen sorption of **SI-4e**:

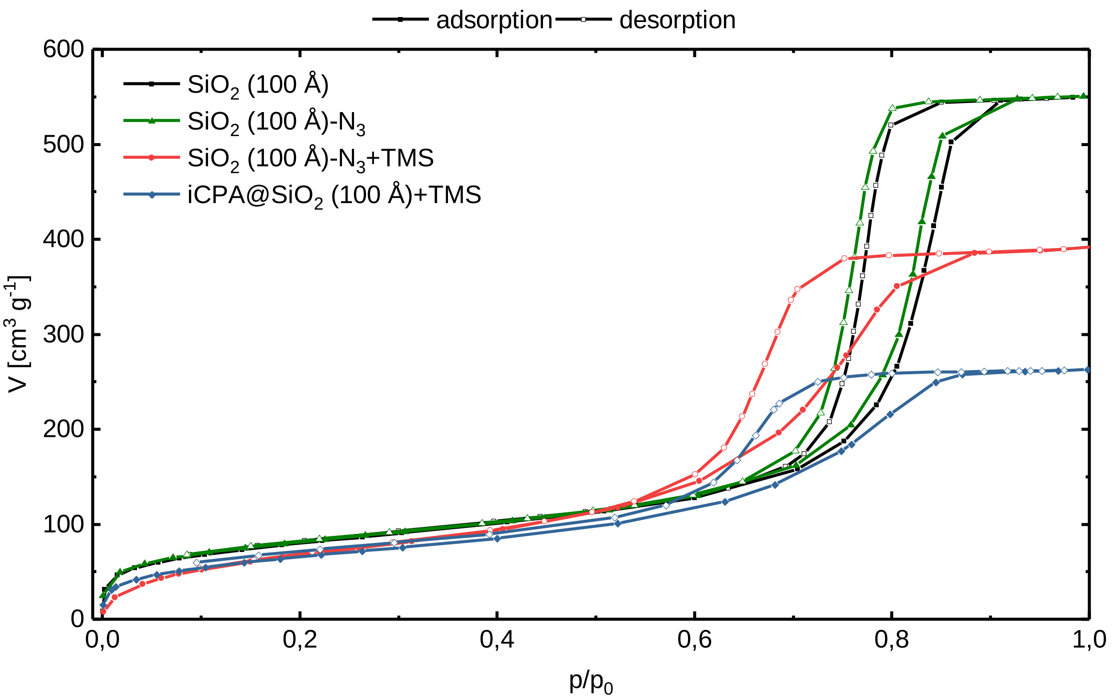

SI-4e (blue): A(BET) = 239 m^2/g; V(P) = 0.41 cm^3/g

Supplementary figure SF24: Pore width distribution (from BJH model) of **SI-4e**

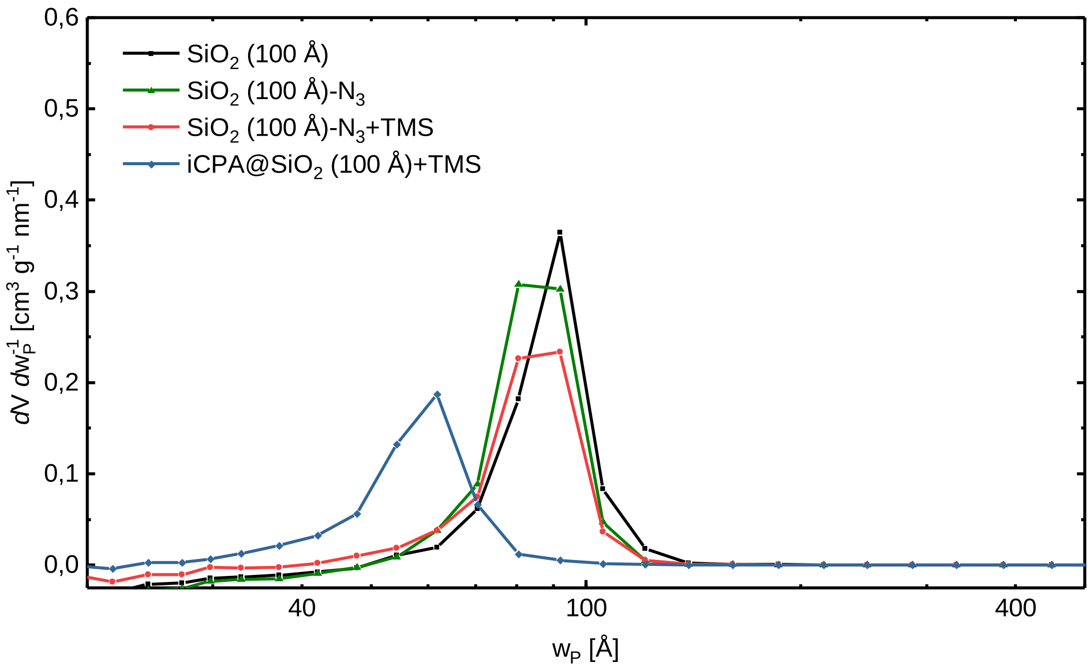

Supplementary figure SF25: TGA of **SI-4e**:

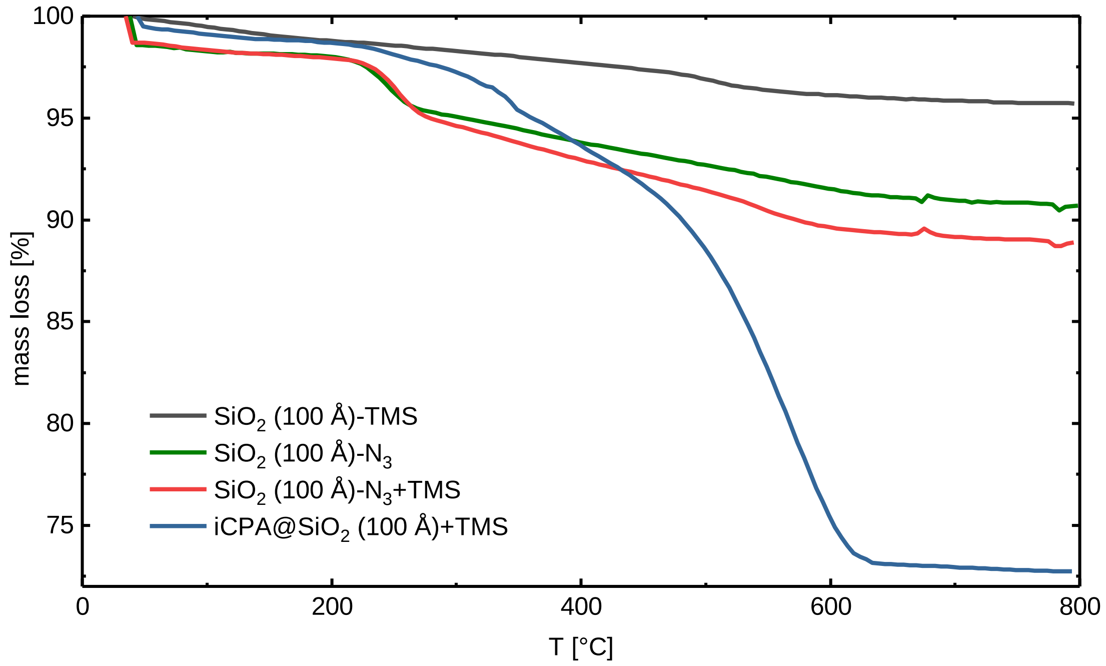

iCPA@SiO2 **SI-4d**:

Synthesized after general procedure Click-reaction and isolated as a light-brown solid. The catalyst loading was determined by ICP-OES (phosphorus-content) *f* = 0.10 mmol/g, CHN elemental analysis *f* = 0.10 mmol/g and thermogravimetric analysis *f* = 0.10 mmol/g.

Supplementary figure SF26: ^{1}H Solid-state-MAS-NMR of **SI-4d**:

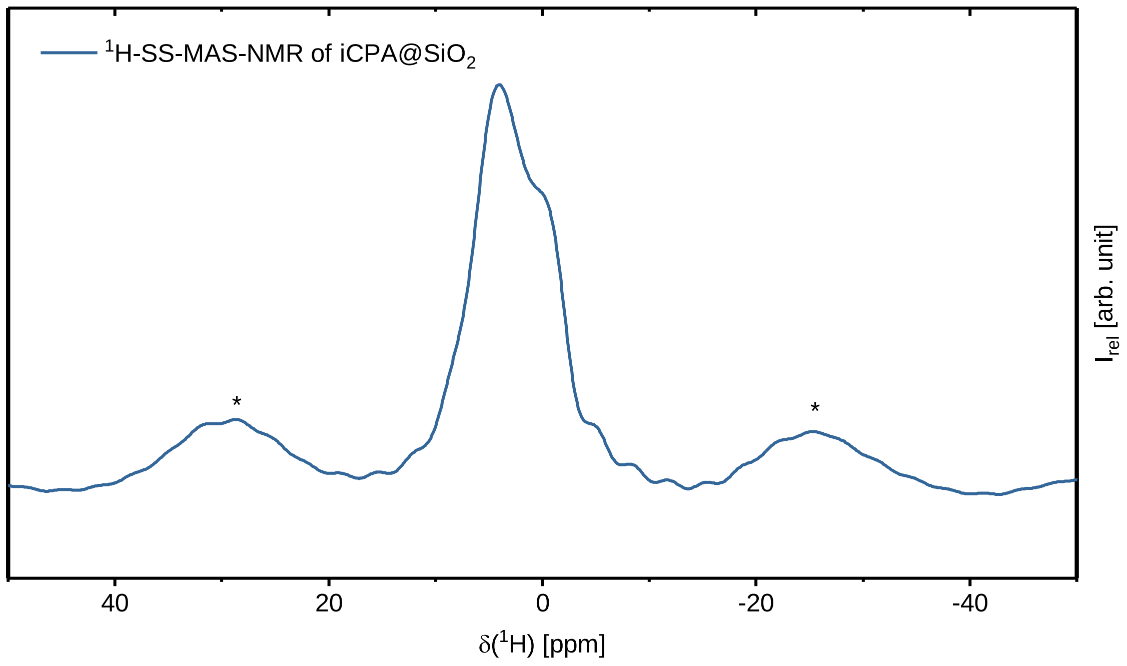

Supplementary figure SF27: ^{31}P Solid-state-MAS-NMR of **SI-4d**:

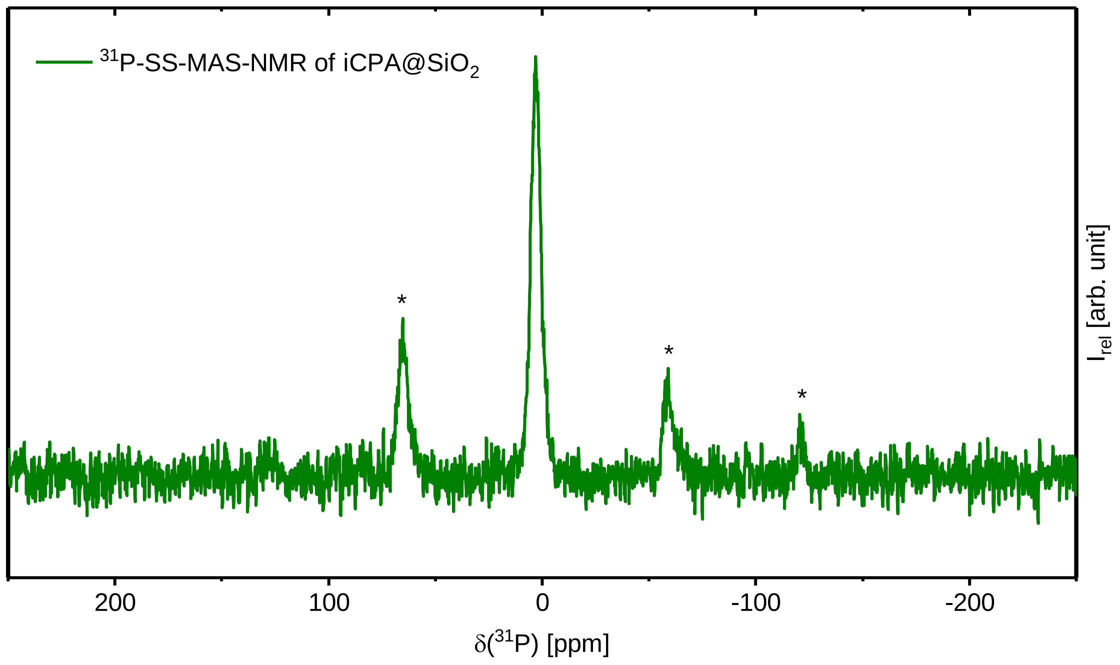

Supplementary figure SF28: Nitrogen sorption of **SI-4d**:

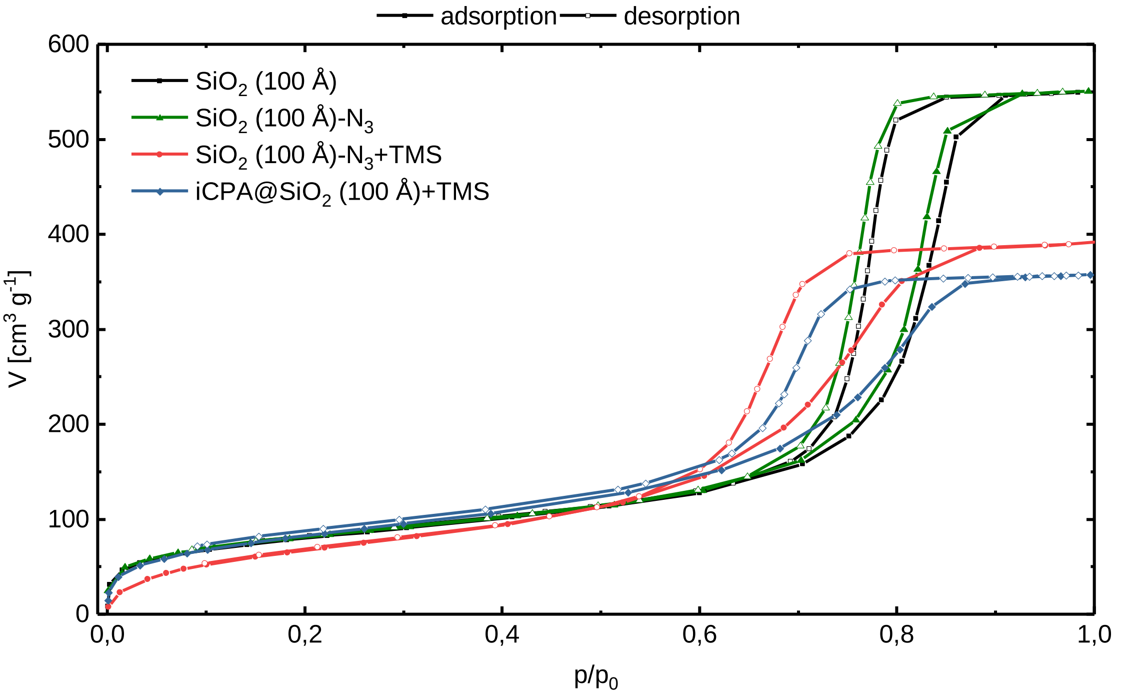

SI-4d (blue): A(BET) = 202 m^2/g; V(P) = 0.55 cm^3/g

Supplementary figure SF29: Pore width distribution (from BJH model) of **SI-4d**

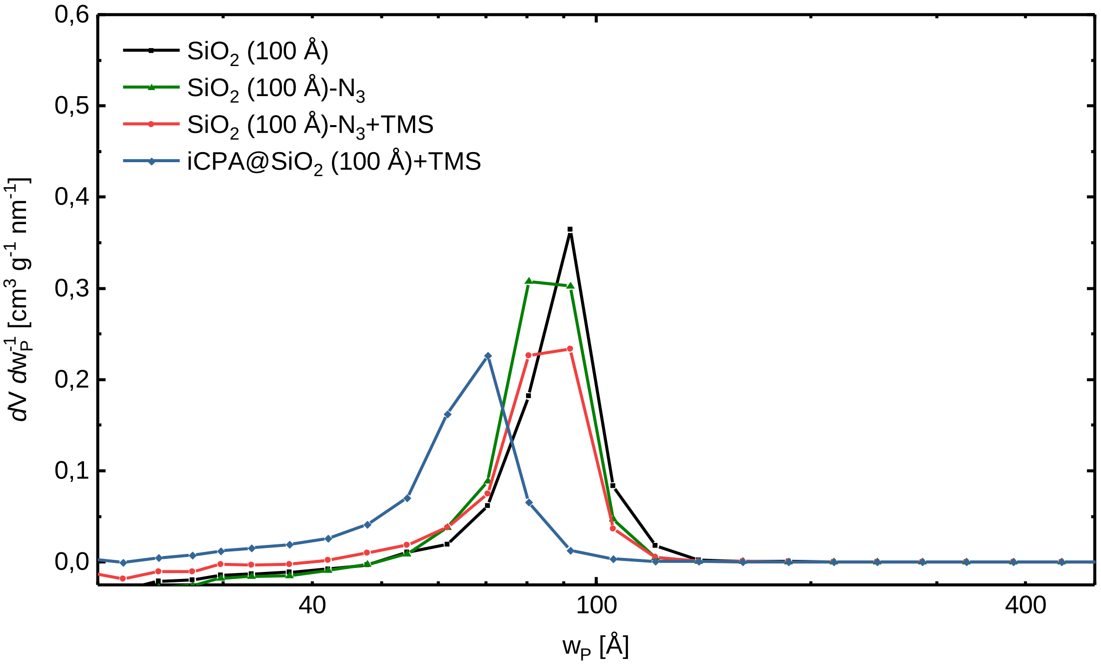

Supplementary figure SF30: TGA-data of **SI-4d**:

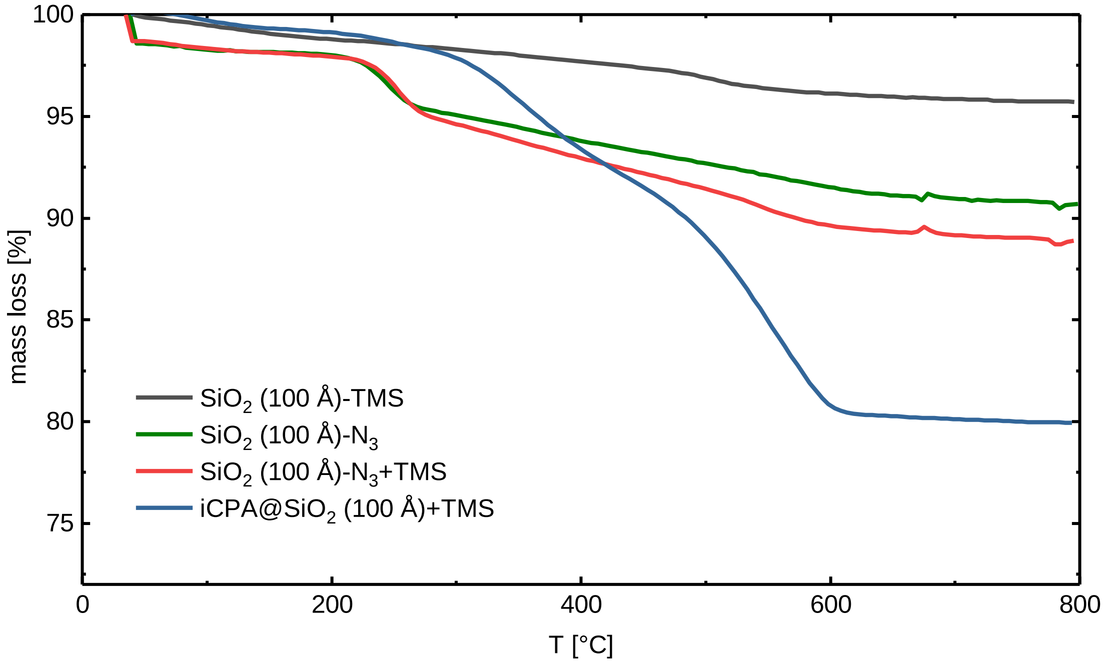

1.6 Experimental Procedures for Batch and Flow Reactions

General Procedure Transfer Hydrogenation:

General Information: Quinolines **SI-1a**, **SI-1c** and **SI-1d** were purchased from commercial suppliers. Before use they were dissolved in $CHCl_3$, insoluble residues were filtered off to avoid blocking of the frits in the flow process and the solvent was evaporated. Quinoline **SI-1b** and benzoxazine **SI-31** were prepared according to literature procedures.[6,7] Hantzsch esters **SI-2a-c** were also prepared as described in the literature and used after recrystallization from MeOH/ EtOH.[8,9]

Homogeneous Batch Reactions:

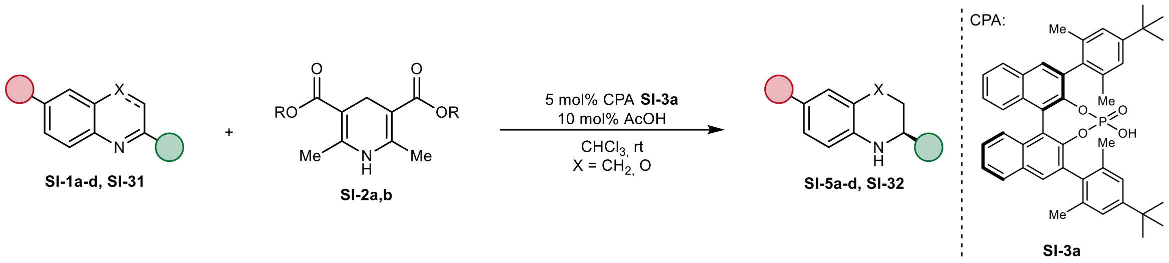

In a screw-cap vial equipped with a magnetic stirring bar 1.0 eq. (100 µmol) of the corresponding quinoline/ benzoxazine, 2.4 eq. Hantzsch ester (1.2 eq. in case of the benzoxazine) and 5 mol% of the molecular phosphoric acid catalyst were dissolved in chloroform (0.05 M, with 10 mol% AcOH) and stirred at ambient temperature. After full consumption was indicated by TLC the crude product was purified by flash column chromatography to give the pure tetrahydroquinolines.

Heterogeneous Batch Reactions:

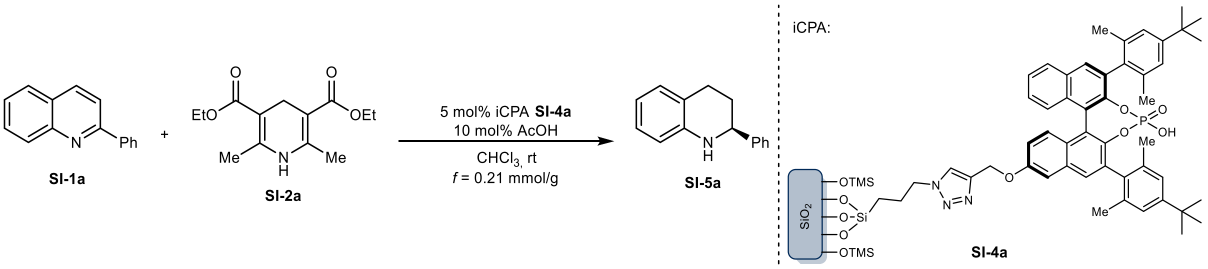

In a screw-cap vial 1.0 eq. (100 µmol) of the corresponding quinoline, 2.4 eq. Hantzsch ester and 5 mol% of the immobilized phosphoric acid catalyst (iCPA **SI-4a-c**) was suspended in chloroform (0.05 M, with 10 mol% AcOH) and shaken at a vibratory plate at ambient temperature. After full consumption was indicated by TLC the catalyst was filtered off and the crude product was submitted to crude-q-NMR and HPLC.

Heterogeneous Flow Reactions:

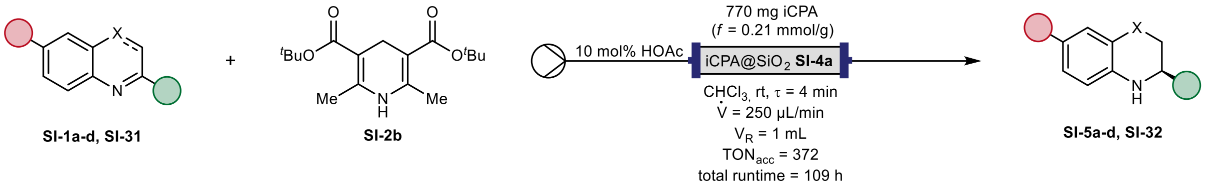

In a pointed flask 1.0 eq. (500 µmol) of the corresponding quinoline or benzoxazine and 2.4 eq. Hantzsch ester (1.2 eq. in case of the benzoxazine) were dissolved in chloroform (0.05 M, with 10 mol% AcOH). The mixture was pumped through a fixed-bed reactor (stainless steel column) charged with 770 mg of iCPA **SI-4a** (*f* = 0.21 mmol/g) at a flow rate of 250 µl/min for 44 mins and collected in a flask (the reactor was flushed with Hantzsch ester solution in $CHCl_3$ and AcOH 5 mins before the product entered and 7.5 mins after the product left the reactor). The solvent was evaporated and the reaction mixture was purified via flash column chromatography to give the pure tetrahydroquinolines.

General Procedure Friedländer-Reaction Transfer Hydrogenation Cascade:

General Information: Amino-benzaldehydes **SI-6a,b** were purchased from commercial suppliers. Before use they were dissolved in $CHCl_3$, insoluble residues were filtered off to avoid blocking of the frits in the flow process and the solvent was evaporated. The β- keto esters **SI-7a-d** were bought from commercial suppliers and distilled prior to use. Hantzsch esters **SI-2a-c** were prepared as described in the literature and used after recrystallization from MeOH/ EtOH.[8,9] The immobilized phosphonic acid **SI-8** was purchased from Sigma Aldrich, ICP-OES indicated a catalyst loading of *f* = 0.87 mmol/g. The material was used without further purification.

Homogeneous Batch Reactions:

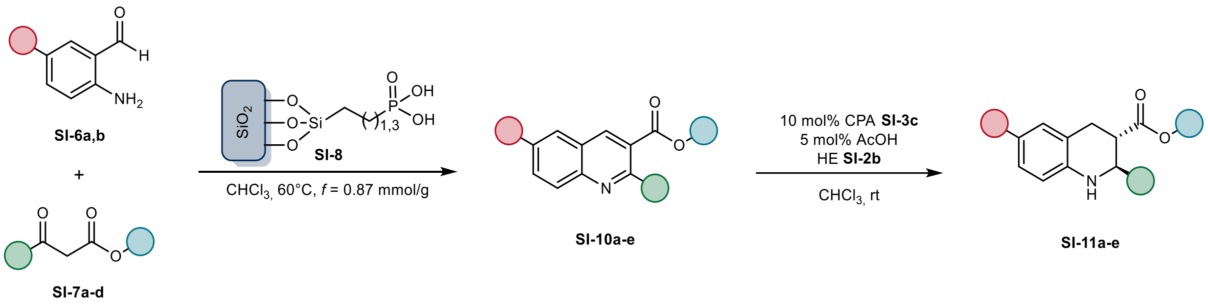

In a screw-cap vial equipped with a magnetic stirring bar 1.0 eq. (100 µmol) of the corresponding 2-amino-benzaldehyde and 200 mg of the immobilized phosphonic

acid **SI-8** (*f* = 0.87 mmol/g) were dissolved in chloroform (0.05 M, with 5 mol% AcOH). Then 2.0 eq. of the ketoester was added and the mixture was stirred at 60 °C overnight (18 h). After full conversion (TLC) the mixture was cooled to ambient temperature, the phosphonic acid was filtered off and the solvent was removed under vacuum. Afterwards 5.0 eq. of Hantzsch ester **SI-2b** as well as 10 mol% of phosphoric acid **SI-3c** were added, the mixture was dissolved in chloroform (0.05 M, with 5 mol% AcOH) and stirred for 24h (TLC). After completion the solvent was evaporated at ambient temperature and the crude product was purified via flash column chromatography to afford the desired tetrahydroquinolines **SI-11a-e**. For separation of diastereomers an additional preparative TLC (SiO_2, ALOX) is recommended.

Heterogeneous Flow Reactions:

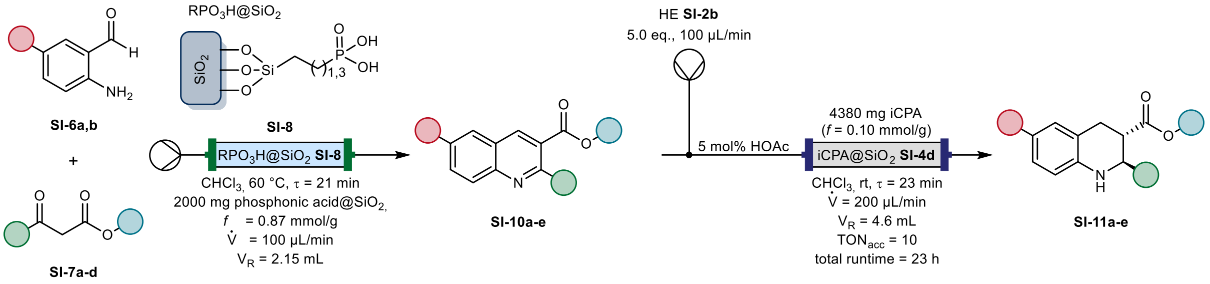

In a pointed flask 1.0 eq. (250 µmol) of the corresponding 2-amino-benzaldehyde was dissolved in chloroform (0.05 M, with 5 mol% AcOH) and 2.0 eq. of the ketoester was added. In a second pointed flask 5.0 eq. of the Hantzsch ester was dissolved in chloroform (0.05 M, with 5 mol% AcOH). The mixture with the aminobenzaldehyde was pumped through a fixed-bed reactor (stainless steel column, heated to 60 °C) charged with 2000 mg of the immobilized phosphonic acid **SI-8** (*f* = 0.87 mmol/g) with a flow rate of 100 µl/min for 72 mins. After leaving the reactor the mixture was allowed to cool down to ambient temperature before it was mixed with the Hantzsch ester solution and pumped through the second fixed-bed reactor (stainless steel column) charged with 4380 mg of iCPA **SI-4d** (*f* = 0.10 mmol/g) at a combined flow rate of 200 µl/min for 73 mins (the reactor was flushed with Hantzsch ester solution in $CHCl_3$ and AcOH 5 mins before the product entered and 7.5 mins after the product left the reactor). The reaction mixture was collected in a flask, the solvent was evaporated at ambient temperature and the crude product was purified via flash column chromatography to afford the desired tetrahydroquinolines **SI-11a-e**. For separation of diastereomers an additional preparative TLC (SiO_2, ALOX) is recommended.

General Procedure Mannich Reaction:

General Information: Aldehydes **SI-12a-e** were purchased from commercial suppliers and distilled prior to use, *para*-Anisidine was recrystallized from hexane, DMPU (anhydrous, >99%) was used without further purification.

Homogeneous Batch Reactions:

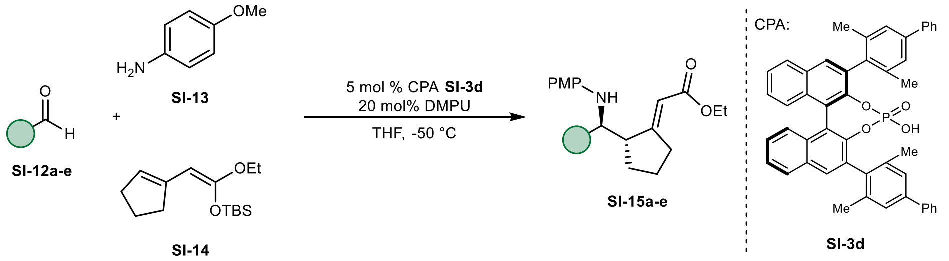

In a screw-cap vial equipped with a magnetic stirring bar 1.00 eq. *para*-anisidine (200 µmol) and 5 mol% of phosphoric acid **SI-3d** were dissolved in THF (0.1 M with 20 mol% DMPU), 1.25 eq. of the aldehyde was added and the mixture was cooled to -50 °C. Then 2.00 eq. of the silyl dienolate was added and the reaction was stirred at this temperature until completion (TLC). The solvent was evaporated and the crude product was purified by flash column chromatography to give the pure amino esters.

Heterogeneous Flow Reactions:

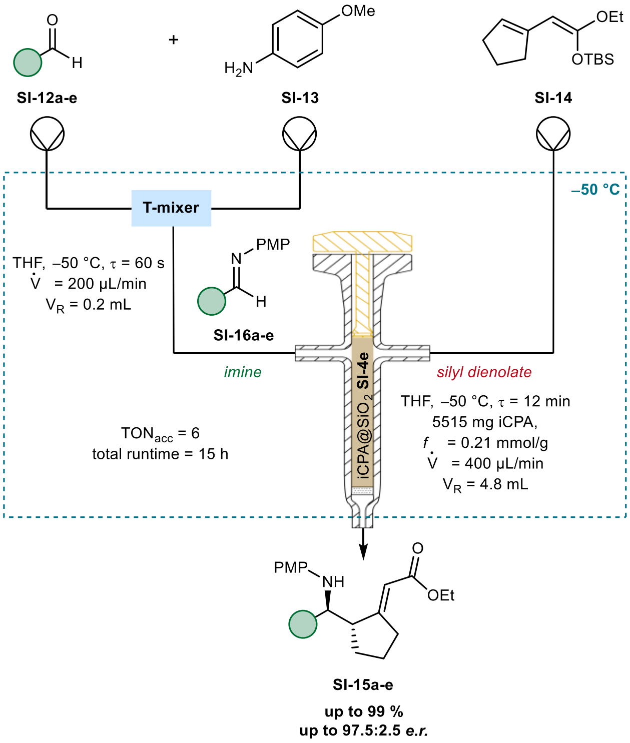

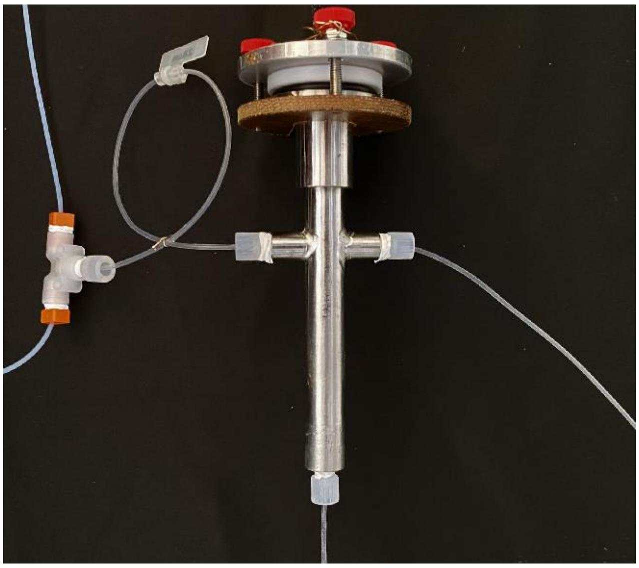

In three pointed flasks (A, B, C) 1.0 eq. (500 µmol) of *para*-anisidin (A), 2.0 eq. of the corresponding aldehyde (B) and 6.0 eq. of silyl dienolate (C) were dissolved in THF (A: 0.20 M, B: 0.27 M, C: 0.40 M, together containing 1.0 eq. DMPU). Mixture A and B were pumped to a mixer cooled to -50 °C at a flow rate of 100 µl/min each (the mixer and following tube were flushed with mixture B 5 mins before the anisidine entered and 7.5 mins after it left the mixer). In this tube of 0.2 ml volume the imine was formed and afterwards together with mixture C pumped through the fixed bed reactor (T-shaped stainless steel) charged with 5515 mg of iCPA **SI-4e** (f = 0.21 mmol/g) with a combined flow rate of 400 µl/min at -50 °C for 37 mins (the reactor was flushed with mixture C 5 mins before the imine entered and 7.5 mins after it left). The reaction mixture was collected in a flask, the solvent was evaporated at ambient temperature and the crude product was purified via flash column chromatography to afford the desired pure amino esters.

1.7 Characterization of Products

(*S*)-2-Phenyl-1,2,3,4-tetrahydroquinoline **SI-5a**:

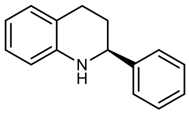

Synthesized after general procedure transfer hydrogenation, the crude product was purified by flash column chromatography (Hex/ MTBE 49:1 $^{v}/_{v}$) and isolated as a white solid with a yield of **97%** (hom. Batch) or **91%** (het. Flow). In case of the het. Batch the Yield was determined by q-NMR analysis of the crude product (**95 - >99%**).

R_f = 0.50 (Hex/ MTBE 14:1).

^{1}H NMR (400 MHz, $CDCl_3$): δ = 7.43 – 7.32 (m, 4H), 7.32 – 7.27 (m, 1H), 7.06 – 6.94 (m, 2H), 6.65 (td, J = 7.4, 1.2 Hz, 1H), 6.55 (d, J = 8.4 Hz, 1H), 4.45 (dd, J = 9.3, 3.3 Hz, 1H), 4.04 (br-s, 1H), 2.93 (ddd, J = 16.3, 10.6, 5.5 Hz, 1H), 2.74 (dt, J = 16.3, 4.7 Hz, 1H), 2.13 (dddd, J = 13.0, 5.5, 4.7, 3.3 Hz, 1H), 2.00 (dddd, J = 13.0, 10.6, 9.3, 5.0 Hz, 1H).

^{13}C (APT) NMR (100 MHz, $CDCl_3$): δ = 145.0, 144.9, 129.4, 128.7, 127.6, 127.0, 126.7, 121.0, 117.3, 114.1, 56.39, 31.11, 26.50.

HRMS (+ESI) m/z: [M+H] Calc. for $C_{15}H_{16}N$: 210.1277, found 210.1289.

IR (KBr) ṽ [cm^{-1}] = 3364, 3062, 3045, 3031, 2968, 2927, 2857, 1605, 1581, 1504, 1483, 1455, 1444, 1310, 1278, 1255 1169, 1109, 759, 742, 714, 700, 449.

HPLC: (IB, *n*-hexanes/2-propanol 95:5, 0.5 ml/min, λ = 300 nm) t_R = 15.5 min (major), 19.9 min (minor). hom. Batch: 99:1 *e.r.*, het. Batch: between 97.5:2.5 and 98:2 *e.r.*, het Flow: 98:2 *e.r.*

[α_D^{23}] = -37° (c = 1.00, $CHCl_3$, 98:2 *e.r.*).

MP = 58-60 °C.

(*S*)-2-(4-Methoxyphenyl)-1,2,3,4-tetrahydroquinoline **SI-5b**:

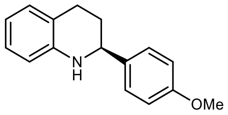

Synthesized after general procedure transfer hydrogenation, the crude product was purified by flash column chromatography (Hex/ MTBE 20:1 $^v/_v$) and isolated as a yellow solid with a yield of **94%** (Batch) or **94%** (Flow).

R_f = 0.66 (Hex/MTBE 4:1)

^{1}H NMR (400 MHz, $CDCl_3$): δ = 7.31 (d, *J* = 8.6 Hz, 2H), 7.04 – 6.97 (m, 2H), 6.89 (d, *J* = 8.6 Hz, 2H), 6.65 (td, *J* = 7.4, 1.2 Hz, 1H), 6.53 (dd, *J* = 8.3, 1.2 Hz, 1H), 4.39 (dd, *J* = 9.5, 3.2 Hz, 1H), 3.99 (br-s, 1H), 3.82 (s, 3H), 2.93 (ddd, *J* = 16.4, 10.9, 5.5 Hz, 1H), 2.75 (dt, *J* = 16.4, 4.4 Hz, 1H), 2.09 (dddd, *J* = 13.0, 5.5, 4.4, 3.2 Hz, 1H), 1.97 (dddd, *J* = 13.0, 10.9, 9.6, 5.1 Hz, 1H).

^{13}C (APT) NMR (100 MHz, $CDCl_3$): δ = 159.1, 145.0, 137.0, 129.4, 127.8, 127.0, 121.0, 117.2, 114.1, 114.0, 55.85, 55.45, 31.23, 26.69.

HRMS (+ESI) m/z: [M+H] Calc. for $C_{16}H_{18}NO$: 240.1383, found 240.1401.

IR (KBr) ṽ [cm^{-1}] = 3365, 2838, 1606, 1583, 1514, 1506, 1485, 1465, 1442, 1432, 1311, 1302, 1288, 1250, 1181, 1167, 1105, 1044, 1033, 831, 820, 747.

HPLC: (IB, *n*-hexanes/2-propanol 90:10, 1.0 ml/min, λ = 250 nm) t_R = 7.4 min (major), 10.1 min (minor). Batch: 99.5:0.5 *e.r.*, Flow: 98:2 *e.r.*

[α_D^{23}] = -26° (c = 1.00, $CHCl_3$, 98:2 *e.r.*).

MP = 94-96 °C.

(*S*)-2-(4-Bromophenyl)-1,2,3,4-tetrahydroquinoline **SI-5c**:

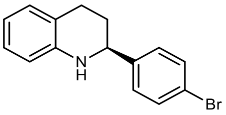

Synthesized after general procedure transfer hydrogenation, the crude product was purified by flash column chromatography (Hex/ MTBE 49:1 $^{v}/_{v}$) and isolated as a white solid with a yield of **93%** (Batch) or **94%** (Flow).

R_f = 0.44 (Hex/MTBE 14:1)

^{1}H NMR (400 MHz, $CDCl_3$): δ = 7.47 (d, *J* = 8.4 Hz, 2H), 7.27 (d, *J* = 8.4 Hz, 2H), 7.06 – 6.97 (m, 2H), 6.67 (td, *J* = 7.4, 1.2 Hz, 1H), 6.55 (dd, *J* = 7.9, 1.2 Hz, 1H), 4.42 (dd, *J* = 9.1, 3.3 Hz, 1H), 4.01 (br-s, 1H), 2.91 (ddd, *J* = 16.2, 10.4, 5.3 Hz, 1H), 2.71 (dt, *J* = 16.2, 5.0 Hz, 1H), 2.10 (dtd, *J* = 13.2, 5.3, 3.3 Hz, 1H), 1.95 (dddd, *J* = 13.2, 10.4, 9.1, 5.0 Hz, 1H).

^{13}C (APT) NMR (100 MHz, $CDCl_3$): δ = 144.5, 144.0, 131.8, 129.4, 128.4, 127.1, 121.2, 120.9, 117.6, 114.2, 55.76, 31.06, 26.23.

HRMS (+ESI) m/z: : [M+H] Calc. for $C_{15}H_{15}BrN$: 288.0382, found 288.0408.

IR (KBr) ṽ [cm^{-1}] = 3380, 1604, 1489, 1313, 1281, 1007, 807, 754.

HPLC: (IB, *n*-hexanes/2-propanol 90:10, 1.0 ml/min, λ = 244 nm) t_R = 8.9 min (major), 13.6 min (minor). Batch: > 99.5:0.5 *e.r.*, Flow: > 99:1 *e.r.*

[α_D^{23}] = -39° (c = 1.00, $CHCl_3$, > 99:1 *e.r.*).

MP = 122-124 °C.

(*S*)-6-Bromo-2-phenyl-1,2,3,4-tetrahydroquinoline **SI-5d**:

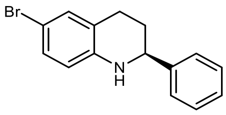

Synthesized after general procedure transfer hydrogenation, the crude product was purified by flash column chromatography (Hex/ MTBE 49:1 $^{v}/_{v}$) and isolated as a white solid with a yield of **92%** (Batch) or **95%** (Flow).

R_f = 0.40 (Hex/MTBE 14:1).

^{1}H NMR (400 MHz, $CDCl_3$): δ = 7.36 (d, J = 4.7 Hz, 4H), 7.33 – 7.27 (m, 1H), 7.13 – 7.06 (m, 2H), 6.42 (d, J = 8.4 Hz, 1H), 4.43 (dd, J = 9.1, 3.4 Hz, 1H), 4.07 (br-s, 1H), 2.87 (ddd, J = 16.5, 10.3, 5.2 Hz, 1H), 2.70 (dt, J = 16.5, 5.0 Hz, 1H), 2.11 (dtd, J = 13.3, 5.2, 3.4 Hz, 1H), 1.96 (dddd, J = 13.3, 10.3, 9.1, 5.0 Hz, 1H).

^{13}C (APT) NMR (100 MHz, $CDCl_3$): δ = 13C NMR (101 MHz, CDCl3) δ 144.5, 143.8, 131.8, 129.7, 128.8, 127.7, 126.6, 123.1, 115.5, 108.6, 56.19, 30.52, 26.19.

HRMS (+ESI) m/z: : [M+H] Calc. for $C_{15}H_{15}BrN$: 288.0382, found 288.0398.

IR (film) ṽ [cm^{-1}] = 3410, 2925, 1599, 1577, 1490, 1470, 1452, 1433, 1336, 1298, 1271, 1251, 1073, 805, 755, 700.

HPLC: (IB, *n*-hexanes/2-propanol 95:5, 1.0 ml/min, λ = 256 nm) t_R = 10.0 min (major), 15.9 min (minor). Batch: > 99:1 *e.r.*, Flow: 98.5:1.5 *e.r.*

[α_D^{23}] = -4° (c = 1.00, $CHCl_3$, 98:2 *e.r.*).

MP = 62-63 °C.

(*R*)-3-Phenyl-3,4-dihydro-2H-benzo[b][1,4]oxazine **SI-32**:

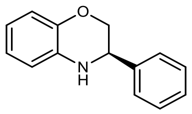

Synthesized after general procedure transfer hydrogenation, the crude product was purified by flash column chromatography (Hex/ MTBE 49:1 $^{v}/_{v}$) and isolated as a yellow oil with a yield of **99%** (Batch) or **96%** (Flow). The Up-Scaling was performed in a scale of 14 mmol, the product was purified by flash column chromatography (Hex/ MTBE 49:1 $^{v}/_{v}$) and isolated as a yellow oil with a yield of **93%** (Flow-Up-Scaling).

R_f = 0.45 (Hex/MTBE 14:1).

^{1}H NMR (400 MHz, $CDCl_3$): δ = 7.44 – 7.32 (m, 5H), 6.90 – 6.78 (m, 2H), 6.74 – 6.66 (m, 2H), 4.52 (dd, J = 8.6, 3.0 Hz, 1H), 4.30 (dd, J = 10.6, 3.0 Hz, 1H), 4.01 (dd, J = 10.6, 8.6 Hz, 1H), 3.65 (br-s, 1H).

^{13}C (APT) NMR (100 MHz, $CDCl_3$): δ = 143.7, 139.3, 134.0, 129.0, 128.5, 127.3, 121.6, 119.1, 116.7, 115.5, 71.10, 54.36.

HRMS (+ESI) m/z: [M+H] Calc. for $C_{14}H_{14}NO$: 212.1070, found 212.1098.

IR (film) ṽ [cm^{-1}] = 3362, 1608, 1592, 1500, 1480, 1454, 1350, 1312, 1280, 1210, 1128, 1056, 1038, 745, 701.

HPLC: (IB, *n*-hexanes/2-propanol 90:10, 1.0 ml/min, λ = 246 nm) t_R = 9.5 min (major), 12.8 min (minor). Batch: > 99:1 *e.r.*, Flow: 96.5:3.5 *e.r.*, Flow (scale up): 96:4 *e.r.*

[α_D^{23}] = -155° (c = 1.00, $CHCl_3$, 96:4 *e.r.*).

(2*R*,3*S*) 3-Ethylcarboxylate 2-methyl-1,2,3,4-tetrahydroquinoline **SI-11a**:

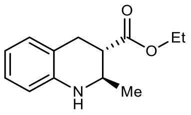

Synthesized after general procedure Friedländer-Reaction transfer hydrogenation Cascade, the crude product was purified by flash column chromatography (Hex/CH_2Cl_2 1:1 $^v/_v$) and isolated as a colorless oil and mixture of diastereomers with a yield of **76%** (5.8:1 *d.r.* Batch) or **68%** (5.2:1 *d.r.* Flow).

R_f = 0.50 (major), 0.44(minor) (Hex/MTBE 2:1).

^{1}H NMR (400 MHz, $CDCl_3$, major diastereomer): δ = 6.99 (td, J = 7.5, 1.1 Hz, 2H), 6.63 (td, J = 7.5, 1.2 Hz, 1H), 6.50 (dt, J = 7.5, 1.2 Hz, 1H), 4.20 (qd, J = 7.1, 1.8 Hz, 2H), 3.72 (br-s, 1H), 3.53 (dq, J = 9.3, 6.2 Hz, 1H), 3.05 (ddt, J = 16.0, 11.5, 1.1 Hz, 1H), 2.92 (dd, J = 16.0, 4.9 Hz, 1H), 2.47 (ddd, J = 11.5, 9.3, 4.9 Hz, 1H), 1.29 (t, J = 7.1 Hz, 3H), 1.24 (d, J = 6.2 Hz, 3H).

^{1}H NMR (400 MHz, $CDCl_3$, minor diastereomer): δ = 7.05 – 6.95 (m, 2H), 6.65 (td, J = 7.4, 1.2 Hz, 1H), 6.51 (dd, J = 8.0, 1.2 Hz, 1H), 4.19 (qq, J = 7.3, 3.7 Hz, 2H), 3.97 – 3.80 (m, 2H), 3.12 – 2.86 (m, 3H), 1.28 (t, J = 7.3 Hz, 3H), 1.14 (d, J = 6.5 Hz, 3H).

^{13}C (APT) NMR (75 MHz, $CDCl_3$): δ = 174.6, 143.7, 129.2, 127.2, 119.6, 117.4, 114.0, 60.72, 49.24, 45.80, 30.76, 20.69, 14.42.

HRMS (+ESI) m/z: : [M+Na] Calc. for $C_{13}H_{17}NO_2Na$: 242.1151, found 242.1158.

IR (film) ṽ [cm^{-1}] = 3395, 2977, 2930, 1730, 1608, 1588, 1496, 1451, 1377, 1306, 1262, 1248, 1179, 1149, 1033, 747.

HPLC: (IB, *n*-hexanes/2-propanol 95:5, 1.0 ml/min, λ = 248 nm) $t_{R\text{-}major}$ = 6.5 min (major), 7.2 min (minor) and $t_{R\text{-}minor}$ = 8.8 min (minor), 9.8 min (major). Batch: 96:4 *e.r.* (major), 91:9 *e.r.* (minor), Flow: 95:5 *e.r.* (major), 87:13 *e.r.* (minor).

[α_D^{23}] = +96° (c = 1.00, $CHCl_3$, 95:5 *e.r.*).

(2*R*,3*S*) 3-*iso*-Propylcarboxylate 2-methyl-1,2,3,4-tetrahydroquinoline **SI-11b**:

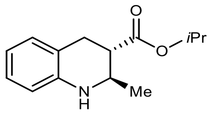

Synthesized after general procedure Friedländer-Reaction transfer hydrogenation Cascade, the crude product was purified by flash column chromatography (Hex/CH_2Cl_2 1:1 $^v/_v$) and isolated as a yellow oil and mixture of diastereomers with a yield of **61%** (3.6:1 *d.r.* Batch) or **58%** (3.1:1 *d.r.* Flow).

R_f = 0.63 (major), 0.57(minor) (Hex/MTBE 2:1).

^{1}H NMR (400 MHz, $CDCl_3$, major diastereomer): δ = 7.03 – 6.95 (m, 2H), 6.63 (t, J = 6.8 Hz, 1H), 6.50 (d, J = 7.8 Hz, 1H), 5.08 (hept, J = 6.3 Hz, 1H), 3.70 (br-s, 1H), 3.52 (dq, J = 9.3, 6.2 Hz, 1H), 3.04 (dd, J = 16.0, 11.5 Hz, 1H), 2.90 (dd, J = 16.0, 4.8 Hz, 1H), 2.43 (ddd, J = 11.5, 9.3, 4.8 Hz, 1H), 1.27 (d, J = 6.3 Hz, 6H), 1.24 (d, J = 6.2 Hz, 3H).

^{1}H NMR (400 MHz, $CDCl_3$, minor diastereomer): δ = 7.07 – 6.95 (m, 2H), 6.64 (qd, J = 7.4, 1.2 Hz, 1H), 6.57 – 6.47 (m, 1H), 5.07 (hept, J = 6.3 Hz, 1H), 3.95 – 3.80 (m, 2H), 3.10 – 2.98 (m, 1H), 2.96 – 2.85 (m, 2H), 1.25 (d, J = 6.3 Hz, 6H), 1.13 (d, J = 6.5 Hz, 3H).

^{13}C (APT) NMR (75 MHz, $CDCl_3$): δ = 174.1, 143.7, 129.2, 127.2, 119.7, 117.4, 113.9, 68.04, 49.26, 45.97, 30.77, 22.01, 21.93, 20.64.

HRMS (+ESI) m/z: : [M+H] Calc. for $C_{14}H_{20}NO_2$: 234.1489, found 234.1494.

IR (film) ṽ [cm^{-1}] = 3394, 2978, 2931, 1726, 1608, 1588, 1496, 1454, 1376, 1306, 1267, 1248, 1180, 1150, 1107, 747.

HPLC: (IB, *n*-hexanes/2-propanol 95:5, 1.0 ml/min, λ = 248 nm) $t_{R\text{-major}}$ = 6.1 min (major), 6.7 min (minor) and $t_{R\text{-minor}}$ = 7.9 min (minor), 8.5 min (major). Batch: 96:4 *e.r.* (major), 80:20 *e.r.* (minor), Flow: 93:7 *e.r.* (major), 77.5:22.5 *e.r.* (minor).

[α_D^{23}] = +92° (c = 1.00, $CHCl_3$, 93:7 *e.r.*).

(2*R*,3*S*) 3-Methylcarboxylate 2-methyl-1,2,3,4-tetrahydroquinoline **SI-11c**:

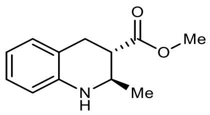

Synthesized after general procedure Friedländer-Reaction transfer hydrogenation Cascade, the crude product was purified by flash column chromatography (Hex/CH_2Cl_2 1:1 $^v/_v$) and isolated as a yellow oil and mixture of diastereomers with a yield of **27%** (3.5:1 *d.r.* Batch) or **71%** (2.7:1 *d.r.* Flow). Diastereomers were separated via preparative TLC (SiO_2, Hex/Et_2O 5:1 $^v/_v$) after column chromatography.

R_f = 0.48 (major), 0.40 (minor) (100% CH_2Cl_2).

^{1}H NMR (400 MHz, $CDCl_3$, major diastereomer): δ = 7.03 – 6.96 (m, 2H), 6.64 (td, J = 7.4, 1.5 Hz, 1H), 6.51 (dd, J = 8.1, 1.5 Hz, 1H), 3.75 (s, 3H), 3.70 (br-s, 1H), 3.54 (dq, J = 9.3, 6.2 Hz, 1H), 3.06 (ddt, J = 16.1, 11.4, 1.2 Hz, 1H), 2.93 (dd, J = 16.1, 4.9 Hz, 1H), 2.51 (ddd, J = 11.4, 9.3, 4.9 Hz, 1H), 1.24 (d, J = 6.2 Hz, 3H).

^{1}H NMR (400 MHz, $CDCl_3$, minor diastereomer): δ = 7.04 – 6.96 (m, 2H), 6.66 (td, J = 7.4, 1.2 Hz, 1H), 6.51 (dd, J = 8.0, 1.2 Hz, 1H), 3.97 – 3.81 (m, 2H), 3.73 (s, 3H), 3.14 – 2.87 (m, 3H), 1.14 (d, J = 6.5 Hz, 3H).

^{13}C (APT) NMR (100 MHz, $CDCl_3$): δ = 175.0, 143.7, 129.2, 127.3, 119.5, 117.5, 114.0, 51.93, 49.23, 45.71, 30.73, 20.74.

HRMS (+ESI) m/z: : [M+H] Calc. for $C_{12}H_{16}NO_2$: 206.1176, found 206.1176.

IR (film) ṽ [cm^{-1}] = 3395, 2952, 2928, 2850, 1733, 1608, 1589, 1496, 1437, 1381, 1371, 1307, 1266, 1248, 1195, 1173, 1150, 748.

HPLC: (IB, *n*-hexanes/2-propanol 98:2, 1.0 ml/min, λ = 248 nm) $t_{R\text{-major}}$ = 10.2 min (major), 12.1 min (minor) and $t_{R\text{-minor}}$ = 17.5 min (minor), 20.4min (major). Batch: 96:4 *e.r.* (major), 90.5:9.5 *e.r.* (minor), Flow: 95:5 *e.r.* (major), 85.5:14.5 *e.r.* (minor).

[α_D^{23}] = +100° (c = 1.00, $CHCl_3$, 95:5 *e.r.*).

(2*S*,3*R*) 3-Ethylcarboxylate 2-phenyl-1,2,3,4-tetrahydroquinoline **SI-11d**:

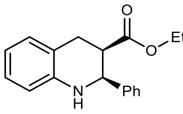

Synthesized after general procedure Friedländer-Reaction transfer hydrogenation Cascade, the crude product was purified by flash column chromatography (Hex/CH_2Cl_2 1:1 $^v/_v$) and isolated as a yellow oil and mixture of diastereomers with a yield of **49%** (2.7:1 *d.r.* Batch) or **56%** (single diastereomer Flow). Diastereomers were separated via preparative TLC (ALOX, Hex/CH_2Cl_2 1:1 $^v/_v$) after column chromatography. Configuration assigned via comparison of NMR coupling constants and α_D with the literature.[10]

R_f = 0.67 (minor), 0.58 (major) (100% CH_2Cl_2).

^{1}H NMR (400 MHz, $CDCl_3$, major diastereomer): δ = 7.30 – 7.21 (m, 3H), 7.21 – 7.14 (m, 2H), 7.10 – 7.01 (m, 2H), 6.69 (td, J = 7.4, 1.2 Hz, 1H), 6.59 (dd, J = 8.0, 1.2 Hz, 1H), 4.98 (d, J = 4.4 Hz, 1H), 4.42 (br-s, 1H), 4.09 (qd, J = 7.1, 2.4 Hz, 2H), 3.24 (ddd, J = 10.5, 5.7, 4.4 Hz, 1H), 2.99 – 2.83 (m, 2H), 1.20 (t, J = 7.1 Hz, 3H).

^{1}H NMR (400 MHz, $CDCl_3$, minor diastereomer): δ = 7.43 – 7.38 (m, 2H), 7.37 – 7.29 (m, 3H), 7.08 – 6.99 (m, 2H), 6.68 (td, J = 7.4, 1.2 Hz, 1H), 6.55 (dd, J = 8.4, 1.2 Hz, 1H), 4.53 (d, J = 8.8 Hz, 1H), 4.04 (br-s, 1H), 3.93 (qd, J = 7.1, 2.6 Hz, 2H), 3.21 (td, J = 13.3, 12.8, 4.0 Hz, 1H), 3.00 – 2.87 (m, 2H), 0.96 (t, J = 7.1 Hz, 3H).

^{13}C (APT) NMR (75 MHz, $CDCl_3$): δ = 172.2, 143.6, 142.3, 133.9, 129.7, 128.9, 128.7, 128.4, 127.8, 127.5, 127.1, 119.0, 117.3, 113.6, 60.69, 56.09, 43.44, 25.05, 14.25.

HRMS (+ESI) m/z: : [M+Na] Calc. for $C_{18}H_{19}NO_2Na$: 304.1308, found 304.1302.

IR (film) ṽ [cm^{-1}] = 3400, 2980, 2926, 1728, 1608, 1587, 1494, 1454, 1376, 1318, 1269, 1245, 1179, 1156, 1115, 1031, 751, 702.

HPLC: (IB, *n*-hexanes/2-propanol 98:2, 1.0 ml/min, λ = 300 nm) $t_{R\text{-minor}}$ = 10.9 min (major), 12.5 min (minor) and $t_{R\text{-major}}$ = 25.1 min (major), 36.7 min (minor). Batch: > 99.5:0.5 *e.r.* (minor), 94:6 *e.r.* (major), Flow: 94:6 *e.r.* (major).

[α_D^{23}] = +144° (c = 1.00, $CHCl_3$, 94:6 *e.r.*).

(2*R*,3*S*) 6-Bromo 3-ethylcarboxylate 2-methyl-1,2,3,4-tetrahydroquinoline **SI-11e**:

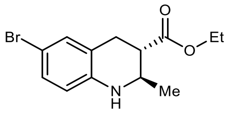

Synthesized after general procedure Friedländer-Reaction transfer hydrogenation Cascade, the crude product was purified by flash column chromatography (Hex/CH_2Cl_2 1:1 $^v/_v$) and isolated as a white solid and mixture of diastereomers with a yield of **67%** (3.4:1 *d.r.* Batch) or **56%** (2.5:1 *d.r.* Flow). Diastereomers were separated via preparative TLC (SiO_2, Hex/Et_2O 5:1 $^v/_v$) after column chromatography.

R_f = 0.52 (major), 0.44 (minor) (Hex/MTBE 2:1 $^v/_v$).

^{1}H NMR (400 MHz, $CDCl_3$, major diastereomer): δ = 7.13 – 7.03 (m, 2H), 6.37 (d, J = 8.4 Hz, 1H), 4.19 (qt, J = 7.1, 2.7 Hz, 2H), 3.73 (br-s, 1H), 3.51 (dq, J = 9.0, 6.2 Hz, 1H), 3.01 (ddt, J = 16.2, 11.1, 1.1 Hz, 1H), 2.87 (dd, J = 16.2, 4.9 Hz, 1H), 2.43 (ddd, J = 11.1, 9.0, 4.9 Hz, 1H), 1.28 (t, J = 7.1 Hz, 3H), 1.23 (d, J = 6.2 Hz, 3H).

^{1}H NMR (400 MHz, $CDCl_3$, minor diastereomer): δ = 7.13 (d, J = 2.3 Hz, 1H), 7.06 (dd, J = 8.5, 2.3 Hz, 1H), 6.38 (d, J = 8.5 Hz, 1H), 4.18 (qq, J = 7.0, 3.7 Hz, 2H), 3.93 (br-s, 1H), 3.84 (qd, J = 6.5, 4.0 Hz, 1H), 3.07 – 2.96 (m, 1H), 2.95 – 2.84 (m, 2H), 1.27 (t, J = 7.0 Hz, 3H), 1.13 (d, J = 6.5 Hz, 3H).

^{13}C (APT) NMR (100 MHz, $CDCl_3$): δ = 174.1, 142.7, 131.6, 129.9, 121.6, 115.4, 108.8, 60.86, 49.14, 45.22, 30.21, 20.64, 14.39.

HRMS (+ESI) m/z: : [M+H] Calc. for $C_{13}H_{17}BrNO_2$: 298.0437, found 298.0452.

IR (film) ṽ [cm^{-1}] = 3396, 2977, 2929, 1730, 1601, 1492, 1444, 1377, 1303, 1264, 1245, 1180, 1150, 1029, 807.

HPLC: (IB, *n*-hexanes/2-propanol 95:5, 1.0 ml/min, λ = 256 nm) $t_{R\text{-major}}$ = 8.6 min (major), 9.7 min (minor) and $t_{R\text{-minor}}$ = 11.5 min (major), 12.3min (minor). Batch: 96.5:3.5 *e.r.* (major), 91:9 *e.r.* (minor), Flow: 94:6 *e.r.* (major), 89:11 *e.r.* (minor).

[α_D^{23}] = +66° (c = 1.00, $CHCl_3$, 94:6 *e.r.*).

MP = 112-114 °C.

(*E*)-2-((*S*)-2-((*R*)-1-((4-Methoxyphenyl)amino)propyl) cyclopentylidene) ethylacetate **SI-15a**:

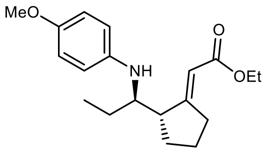

Synthesized after general procedure Mannich reaction the crude product was purified by flash column chromatography (Hex/Et_2O 6:1 $^v/_v$) and isolated as a yellow oil and single diastereomer with a yield of **>99%** (Batch and Flow).

R_f = 0.37 (Hex/Et_2O 7:3 $^v/_v$).

^{1}H NMR (400 MHz, $CDCl_3$) δ = 6.81 – 6.74 (m, 2H), 6.62 – 6.55 (m, 2H), 5.83 (q, J = 2.5 Hz, 1H), 4.17 (qd, J = 7.1, 2.5 Hz, 2H), 3.74 (s, 3H), 3.46 (ddd, J = 9.6, 4.4, 3.1 Hz, 1H), 3.08 (dddd, J = 18.2, 8.3, 4.0, 2.2 Hz, 1H), 3.02 – 2.93 (m, 1H), 2.69 – 2.57 (m, 1H), 1.89 – 1.79 (m, 1H), 1.79 – 1.69 (m, 1H), 1.58 – 1.44 (m, 3H), 1.30 (t, J = 7.1 Hz, 3H), 0.99 (t, J = 7.3 Hz, 3H).

^{13}C (APT) NMR (100 MHz, $CDCl_3$): δ = 170.2, 166.9, 152.1, 141.7, 115.1, 114.7, 112.0, 59.66, 58.56, 55.83, 48.72, 34.26, 26.18, 24.58, 24.10, 14.40, 11.69.

HRMS (+ESI) m/z: : [M+H] Calc. for $C_{19}H_{28}NO_3$: 318.2064, found 318.2068.

IR (film) ṽ [cm^{-1}] = 3388, 2959, 2935, 2904, 2874, 2831, 1709, 1645, 1512, 1464, 1444, 1371, 1351, 1300, 1242, 1232, 1198, 1150, 1127, 1040, 821.

HPLC: (IE, *n*-hexanes/2-propanol 95:5, 0.5 ml/min, λ = 300 nm) $t_{R\text{-major}}$ = 18.4 min (minor), 23.9 min (major), $t_{R\text{-minor}}$ = 19.6 min, 21.1 min Batch: > 99:1 *e.r.*, Flow: 96:4 *e.r.* (THF), 95:5 *e.r.* (2-Me THF).

[α_D^{23}] = -87° (c = 1.00, $CHCl_3$, 96:4 *e.r.*).

(*E*)-2-((*S*)-2-((*R*)-1-((4-Methoxyphenyl)amino)-2-phenylethyl) cyclopentylidene) ethylacetate **SI-15b**:

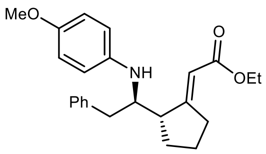

Synthesized after general procedure Mannich reaction the crude product was purified by flash column chromatography (Hex/Et_2O 6:1 $^v/_v$) and isolated as a yellow oil and single diastereomer with a yield of **>99%** (Batch) and **94%** (Flow).

R_f = 0.39 (Hex/Et_2O 7:3 $^v/_v$).

^{1}H NMR (400 MHz, $CDCl_3$) δ = 7.30 – 7.15 (m, 5H), 6.78 – 6.70 (m, 2H), 6.58 – 6.50 (m, 2H), 5.94 (q, J = 2.5 Hz, 1H), 4.19 (qd, J = 7.2, 2.5 Hz, 2H), 3.85 (dt, J = 9.3, 4.0 Hz, 1H), 3.73 (s, 3H), 3.20 – 3.02 (m, 2H), 2.82 (dd, J = 14.2, 3.7 Hz, 1H), 2.75 – 2.55 (m, 2H), 1.95 – 1.80 (m, 2H), 1.70 – 1.50 (m, 2H), 1.32 (t, J = 7.2 Hz, 3H).

^{13}C (APT) NMR (100 MHz, $CDCl_3$): δ = 169.7, 166.9, 152.5, 141.2, 139.3, 129.1, 128.6, 126.5, 115.2, 115.1, 112.7, 59.87, 58.41, 55.91, 48.49, 37.22, 34.46, 26.80, 24.69, 14.53.

HRMS (+ESI) m/z: : [M+H] Calc. for $C_{24}H_{30}NO_3$: 380.2220, found 380.2209.

IR (film) ṽ [cm^{-1}] = 3485, 3405, 2955, 2935, 1713, 1644, 1623, 1513, 1464, 1285, 1265, 1234, 1199, 1147, 1057, 1045, 974, 827, 801, 740, 700, 632, 562, 563, 471, 448, 415.

HPLC: IE, *n*-hexanes/2-propanol 95:5, 0.5 ml/min, λ = 250 nm) t_R = 23.0 min (minor), 29.5 min (major) Batch: > 99.5:0.5 *e.r.*, Flow: 97.5:2.5 *e.r.*

[α_D^{23}] = -123° (c = 1.00, $CHCl_3$, 97.5:2.5 *e.r.*).

(*E*)-2-((*S*)-2-((*R*)-1-((4-Methoxyphenyl)amino)pent-4-yn-1-yl) cyclopentylidene) ethylacetate **SI-15c**:

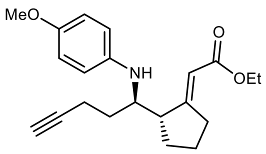

Synthesized after general procedure Mannich reaction the crude product was purified by flash column chromatography (Hex/Et_2O 6:1 $^v/_v$) and isolated as a yellow oil and single diastereomer with a yield of **82%** (Batch) and **92%** (Flow).

R_f = 0.36 (Hex/Et_2O 7:3 $^v/_v$).

^{1}H NMR (400 MHz, $CDCl_3$) δ = 6.81 – 6.75 (m, 2H), 6.64 – 6.58 (m, 2H), 5.83 (q, J = 2.4 Hz, 1H), 4.17 (qd, J = 7.1, 0.9 Hz, 2H), 3.74 (s, 3H), 3.73 – 3.69 (m, 1H), 3.14 –

2.93 (m, 2H), 2.68 – 2.54 (m, 1H), 2.46 – 2.26 (m, 2H), 2.00 (t, J = 2.6 Hz, 1H), 1.90 – 1.80 (m, 1H), 1.80 – 1.64 (m, 2H), 1.57 – 1.41 (m, 3H), 1.30 (t, J = 7.1 Hz, 3H).

^{13}C (APT) NMR (100 MHz, $CDCl_3$): δ = 169.2, 166.8, 152.4, 141.3, 115.1, 115.1, 112.4, 84.11, 68.92, 59.69, 55.81, 48.50, 34.21, 30.14, 25.88, 24.54, 16.18, 14.40.

HRMS (+ESI) m/z: : [M+H] Calc. for $C_{21}H_{28}NO_3$: 342.2064, found 342.2038.

IR (film) ṽ [cm^{-1}] = 3375, 3287, 2954, 2872, 2832, 1705, 1644, 1620, 1510, 1465, 1444, 1371, 1351, 1302, 1287, 1237, 1198, 1126, 1094, 1039, 822, 751, 640, 591, 487, 462, 448, 413.

HPLC: (IB, *n*-hexanes/2-propanol 95:5, 0.5 ml/min, λ = 250 nm)) $t_{R\text{-major}}$ = 19.0 min (major), 23.6 min (minor), $t_{R\text{-minor}}$ = 17.2 min, 23.6 min Batch: > 99:1 *e.r.*, Flow: 96.5:3.5 *e.r.*

[α_D^{23}] = -67° (c = 1.00, $CHCl_3$, 96.5:3.5 *e.r.*).

(*R*)-4-((*S*,*E*)-2-(2-Ethoxy-2-oxoethylidene)cyclopentyl)-4-((4-methoxyphenyl)amino) ethylbutanoate **SI-15d**:

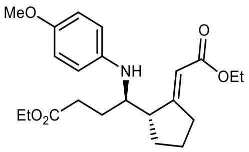

Synthesized after general procedure Mannich reaction the crude product was purified by flash column chromatography (Hex/Et_2O 2:1 $^v/_v$) and isolated as a yellow oil and single diastereomer with a yield of **97%** (Batch) and **> 99%** (Flow).

R_f = 0.46 (Hex/Et_2O 1:1 $^v/_v$).

^{1}H NMR (400 MHz, $CDCl_3$) δ = 6.80 – 6.74 (m, 2H), 6.59 – 6.54 (m, 2H), 5.79 (q, J = 2.4 Hz, 1H), 4.17 (qd, J = 7.2, 1.8 Hz, 2H), 4.10 (qd, J = 7.1, 1.2 Hz, 2H), 3.74 (s, 3H), 3.60 (dt, J = 10.8, 3.6 Hz, 1H), 3.24 (s, 1H), 3.12 – 2.95 (m, 2H), 2.69 – 2.57 (m, 1H), 2.54 – 2.36 (m, 2H), 1.88 – 1.61 (m, 4H), 1.56 – 1.46 (m, 2H), 1.30 (t, J = 7.2 Hz, 3H), 1.21 (t, J = 7.1 Hz, 3H).

^{13}C (APT) NMR (100 MHz, $CDCl_3$): δ = 174.0, 169.7, 166.9, 152.4, 141.3, 115.2, 114.9, 112.3, 60.60, 59.85, 56.60, 55.95, 48.70, 34.34, 32.13, 26.01, 25.81, 24.69, 14.52, 14.35.

HRMS (+ESI) m/z: : [M+H] Calc. for $C_{22}H_{32}NO_5$: 390.2275, found 390.2276.

IR (film) ṽ [cm^{-1}] = 3368, 2979, 2957, 2939, 2905, 2873, 2832, 1731, 1714, 1645, 1513, 1465, 1445, 1372, 1302, 1239, 1199, 1127, 1094, 1039, 822, 755.

HPLC: (IB, *n*-hexanes/2-propanol 95:5, 0.5 ml/min, λ = 250 nm)) $t_{R\text{-major}}$ = 22.7 min (major), 26.6 min (minor), $t_{R\text{-minor}}$ = 20.7 min, 24.9 min Batch: 99:1 *e.r.*, Flow: 95:5 *e.r.*

[α_D^{23}] = -77° (c = 1.00, $CHCl_3$, 95.5:4.5 *e.r.*).

(*E*)-2-((*S*)-2-((*R*)-1-((4-Methoxyphenyl)amino)-3,3-dimethylbutyl) cyclopentylidene) ethylacetate **SI-15e**:

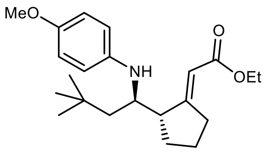

Synthesized after general procedure Mannich reaction the crude product was purified by flash column chromatography (Hex/Et_2O 2:1 $^v/_v$) and isolated as a yellow oil and single diastereomer with a yield of **> 99%** (Batch) and **> 99%** (Flow).

R_f = 0.48 (Hex/Et_2O 7:3 $^v/_v$).

^{1}H NMR (400 MHz, $CDCl_3$) δ = 6.81 – 6.75 (m, 2H), 6.58 – 6.52 (m, 2H), 5.78 (q, J = 2.4 Hz, 1H), 4.18 (qd, J = 7.1, 4.5 Hz, 2H), 3.74 (s, 3H), 3.75 – 3.67 (m, 1H), 3.22 (br-s, 1H), 3.18 – 3.08 (m, 1H), 3.07 – 2.96 (m, 1H), 2.63 – 2.50 (m, 1H), 1.88 – 1.72 (m, 2H), 1.56 – 1.38 (m, 2H), 1.30 (d, J = 7.1 Hz, 3H), 1.23 – 1.18 (m, 2H), 0.93 (s, 9H).

^{13}C (APT) NMR (100 MHz, $CDCl_3$): δ = 170.2, 167.0, 151.9, 141.3, 115.3, 114.0, 112.2, 59.74, 55.95, 53.00, 49.49, 45.27, 34.76, 30.63, 30.40, 25.64, 24.66, 14.52.

HRMS (+ESI) m/z: : [M+H] Calc. for $C_{22}H_{34}NO_3$: 360.2533, found: 360.2558.

IR (film) ṽ [cm^{-1}] = 2955, 2904, 2870, 1709, 1644, 1512, 1466, 1370, 1245, 1234, 1198, 1129, 1040, 819, 756.

HPLC: (IE, *n*-hexanes/2-propanol 98:2, 0.5 ml/min, λ = 250 nm)) $t_{R\text{-major}}$ = 17.2 min (minor), 23.0 min (major), $t_{R\text{-minor}}$ = 20.2 min, 25.8 min Batch: 97:3 *e.r.*, Flow: 95:5 *e.r.*

[α_D^{23}] = -58° (c = 1.00, $CHCl_3$, 95:5 *e.r.*).

2. NMR Spectra of Catalyst Precursors

1H NMR (400 MHz, $CDCl_3$) **SI-18**

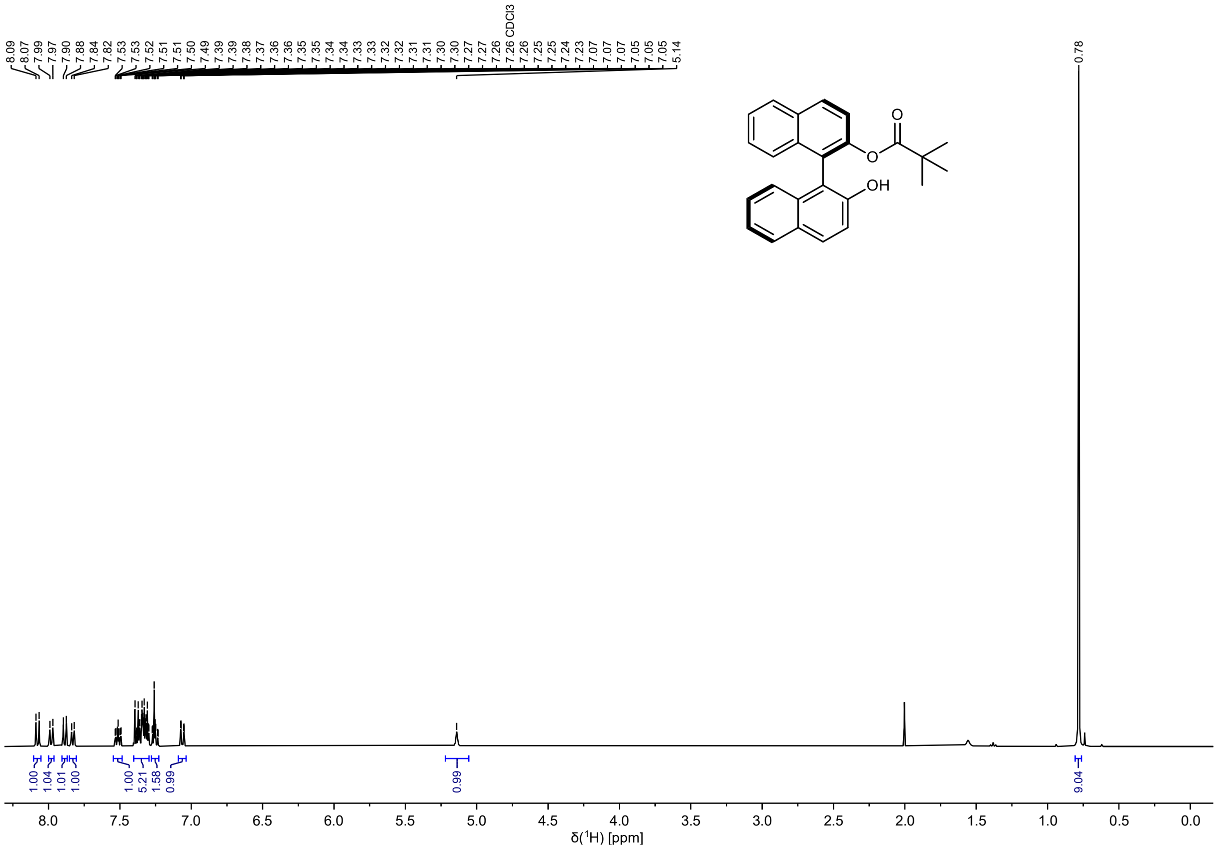

13C (APT) NMR (100 MHz, $CDCl_3$) **SI-18**

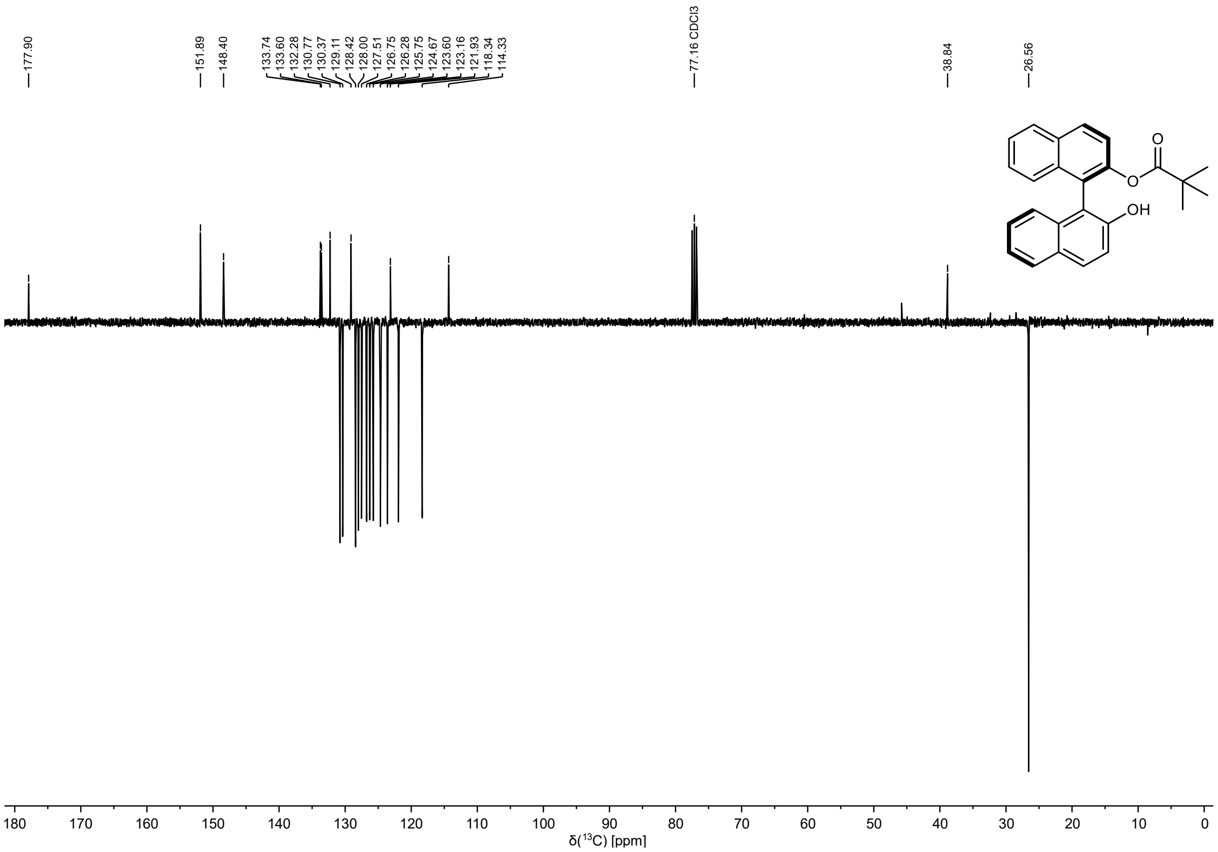

1H NMR (400 MHz, $CDCl_3$) **SI-19**

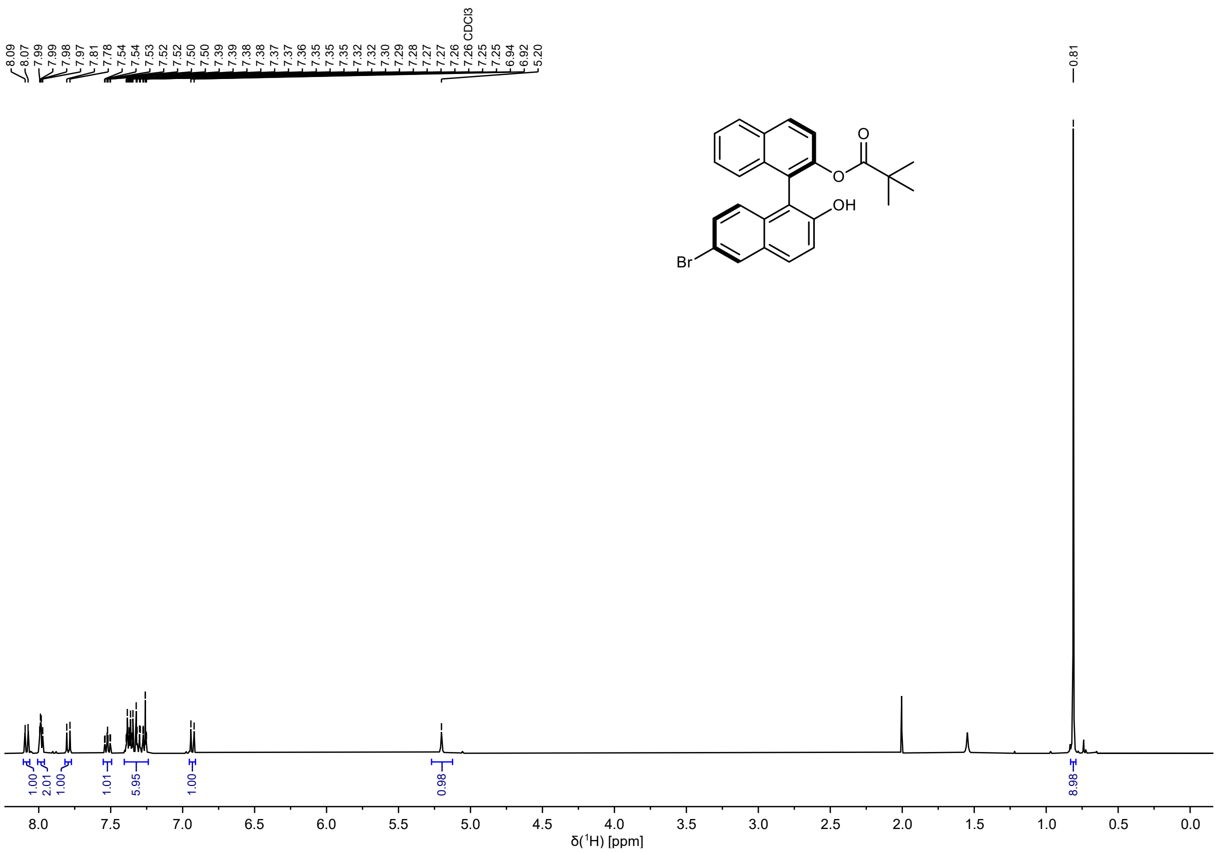

13C (APT) NMR (100 MHz, $CDCl_3$) **SI-19**

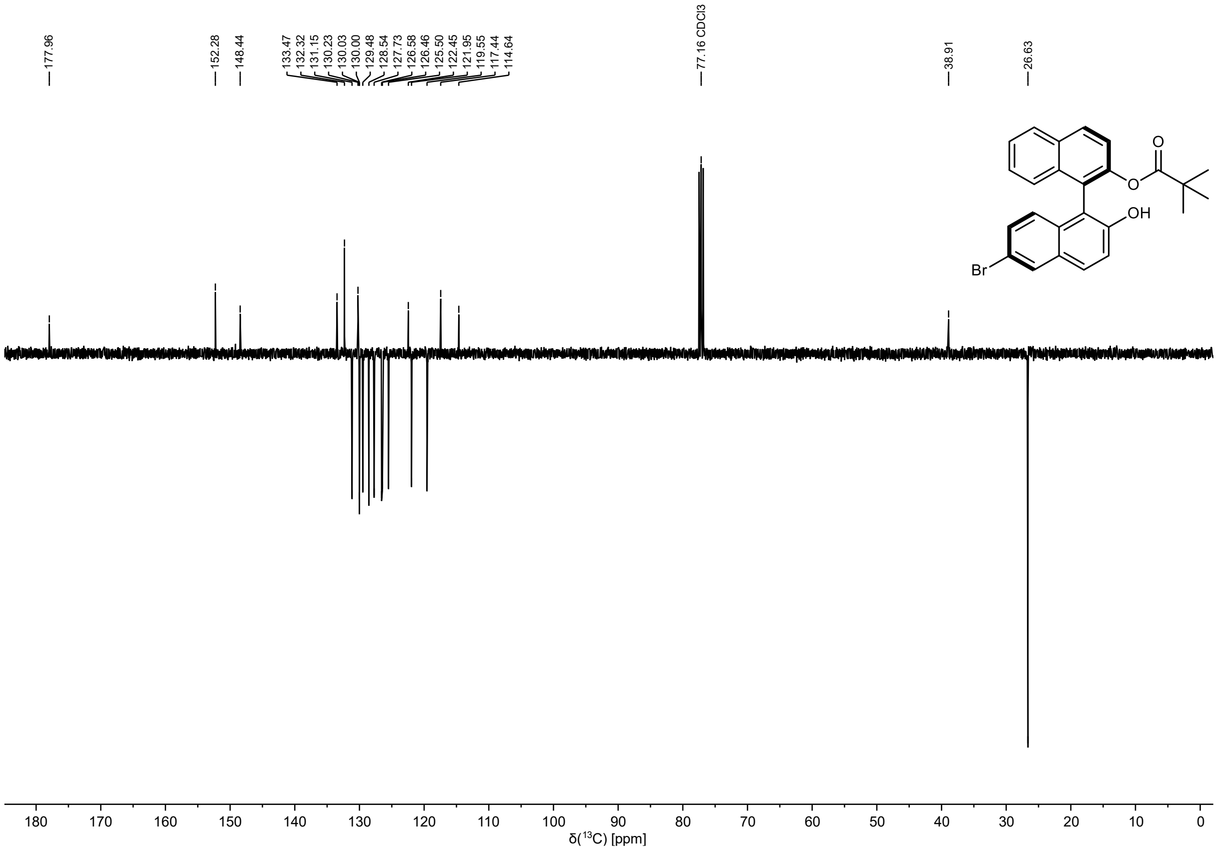

1H NMR (400 MHz, $CDCl_3$) **SI-20**

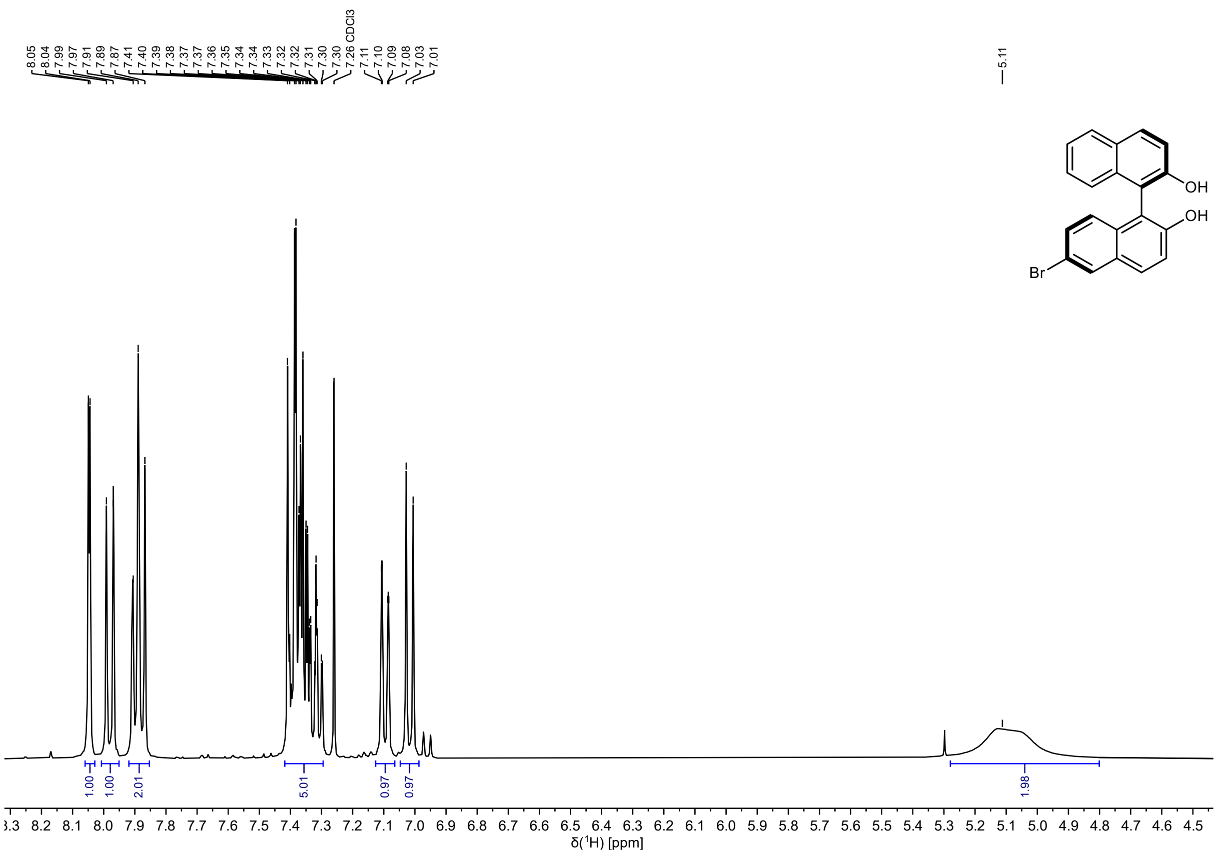

13C (APT) NMR (75 MHz, $CDCl_3$) **SI-20**

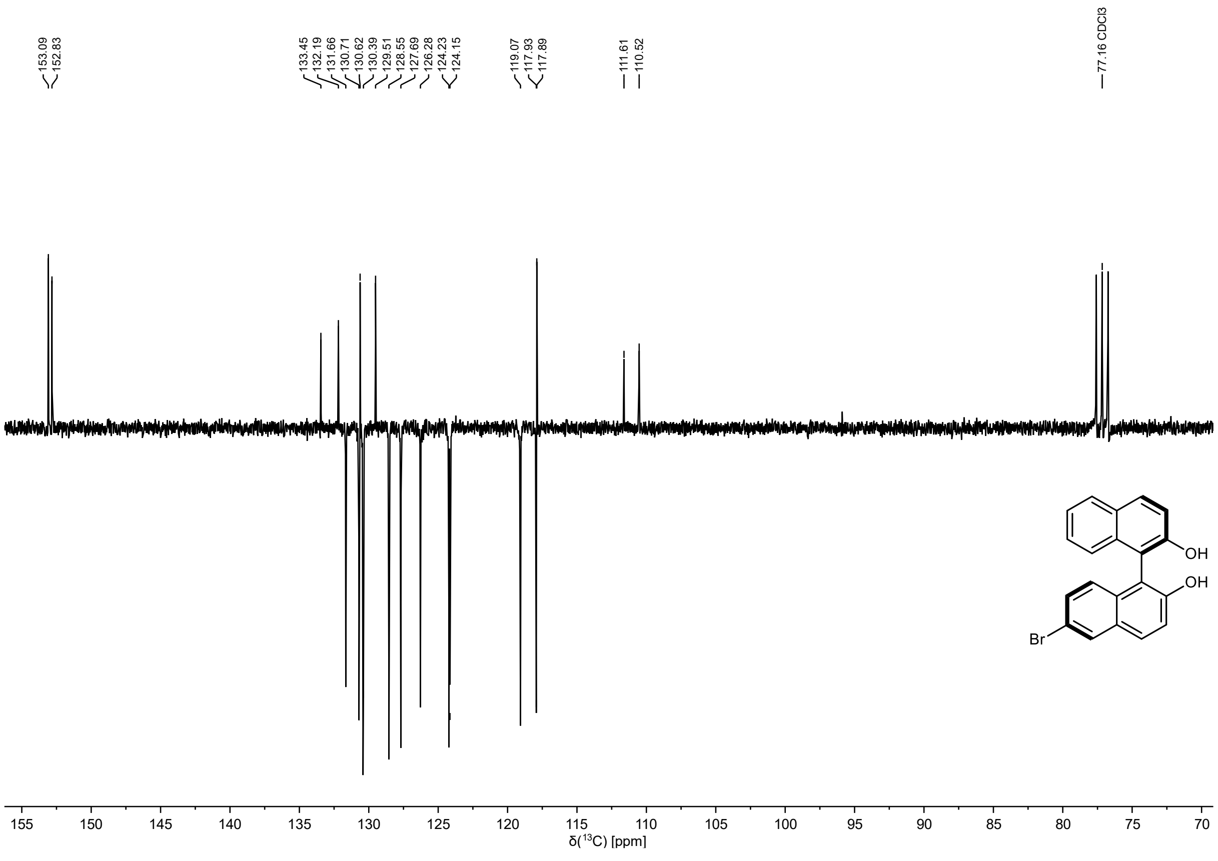

1H NMR (400 MHz, $CDCl_3$) **SI-21**

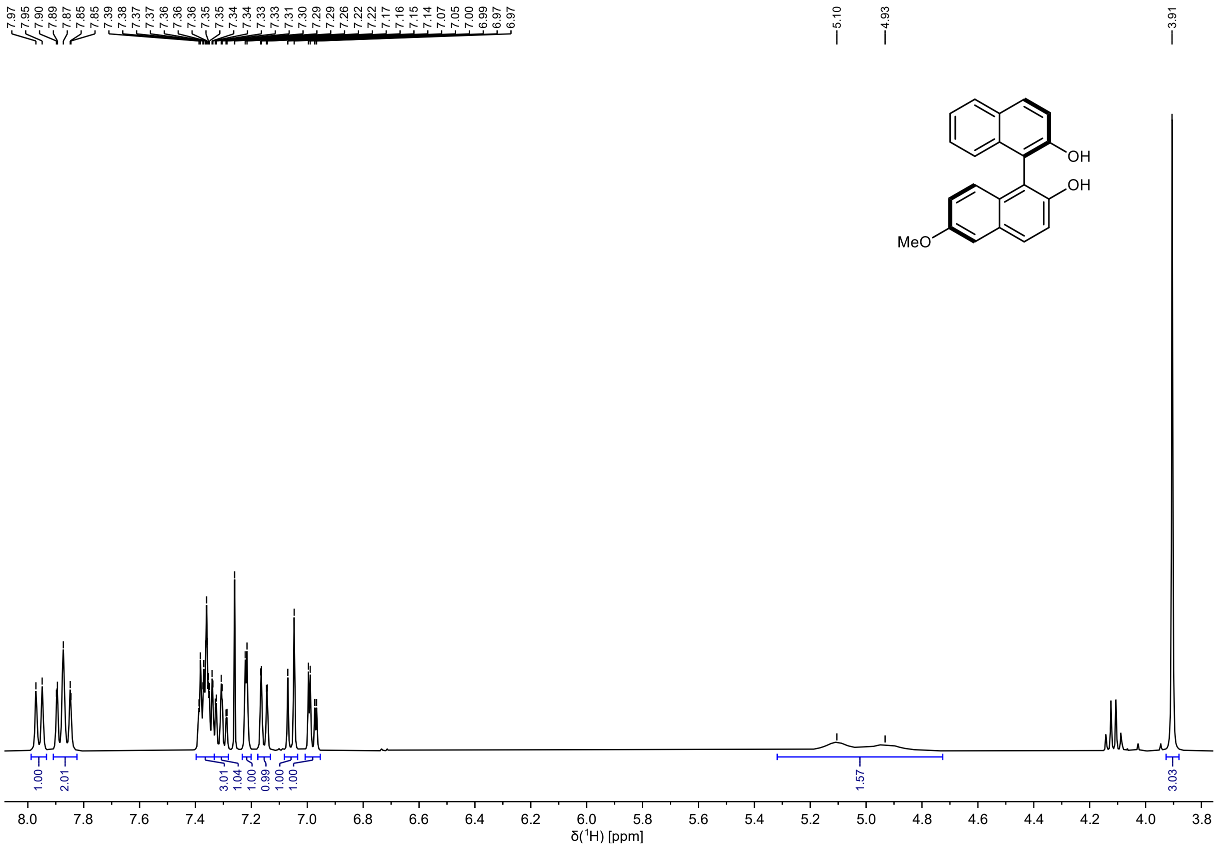

13C (APT) NMR (100 MHz, $CDCl_3$) **SI-21**

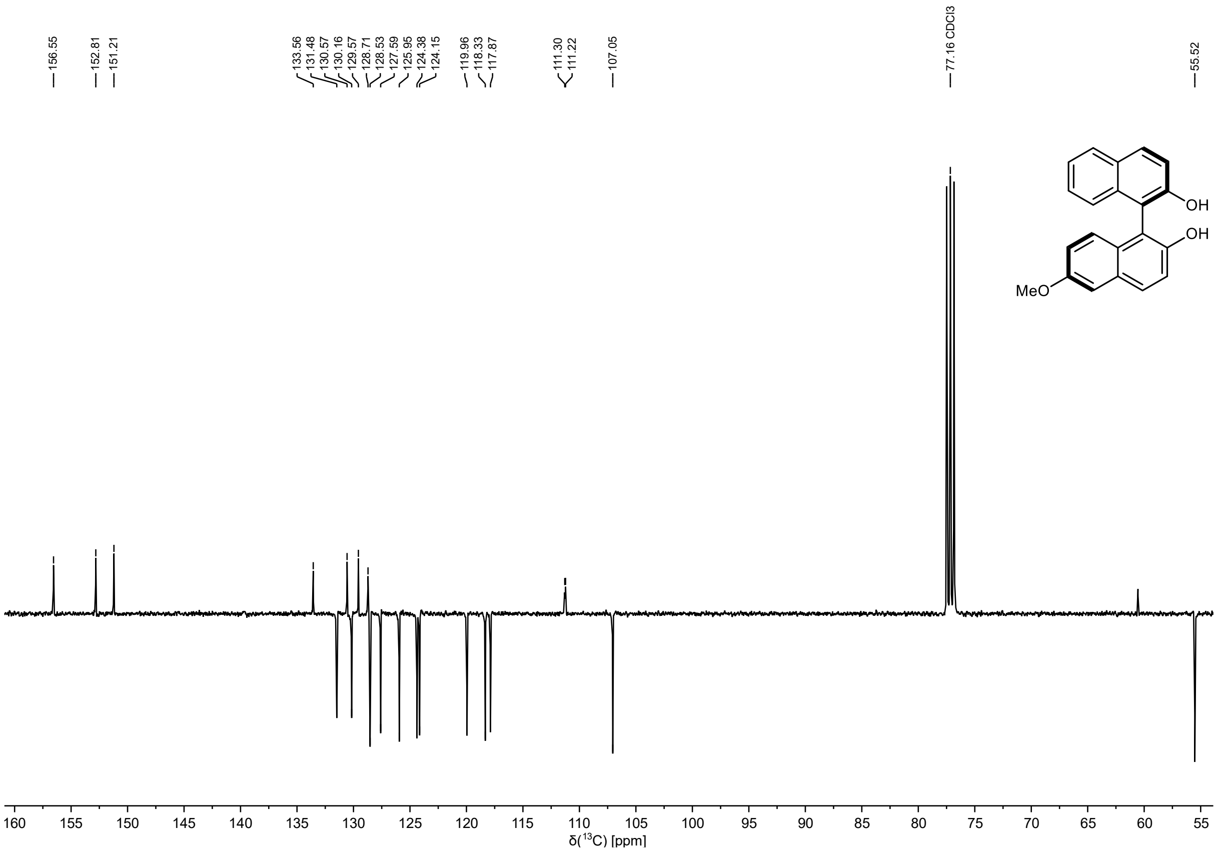

1H NMR (400 MHz, CDCl3) **SI-22**

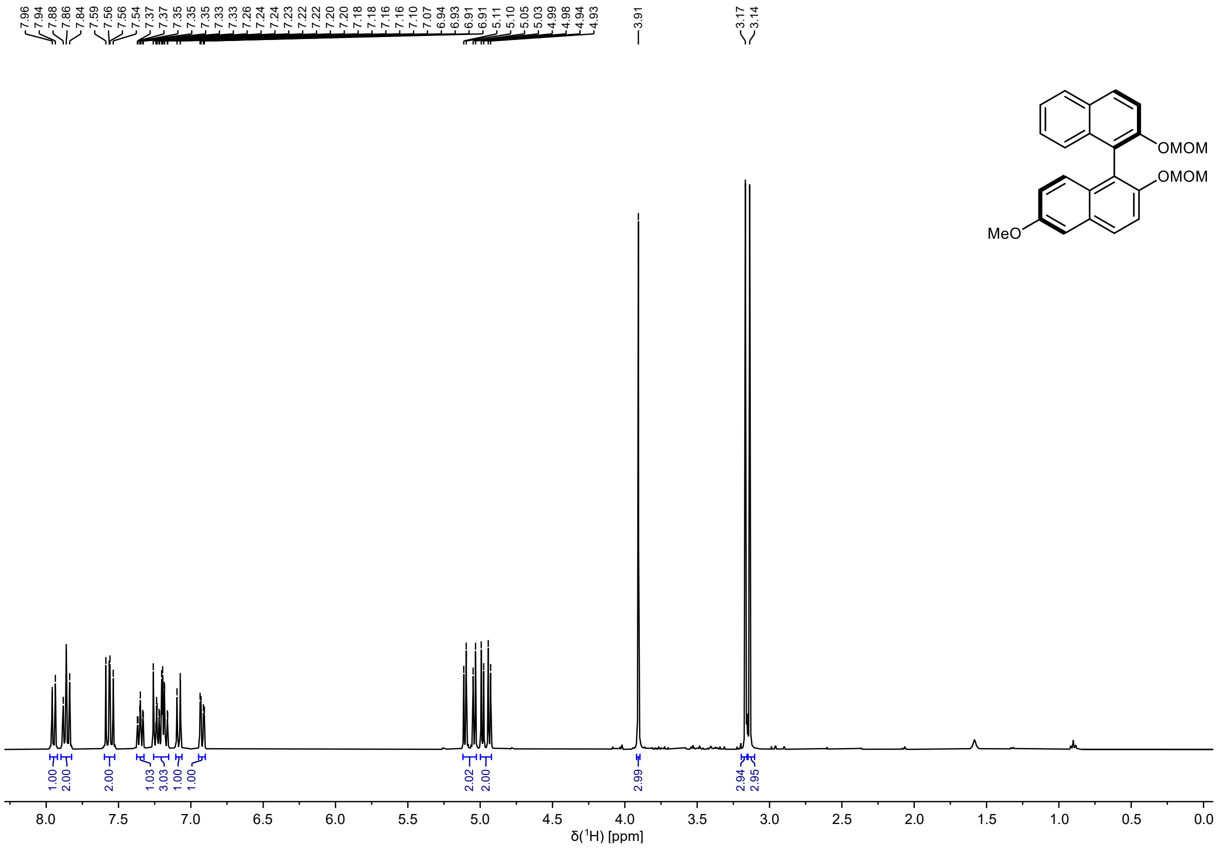

13C (APT) NMR (100 MHz, CDCl3) **SI-22**

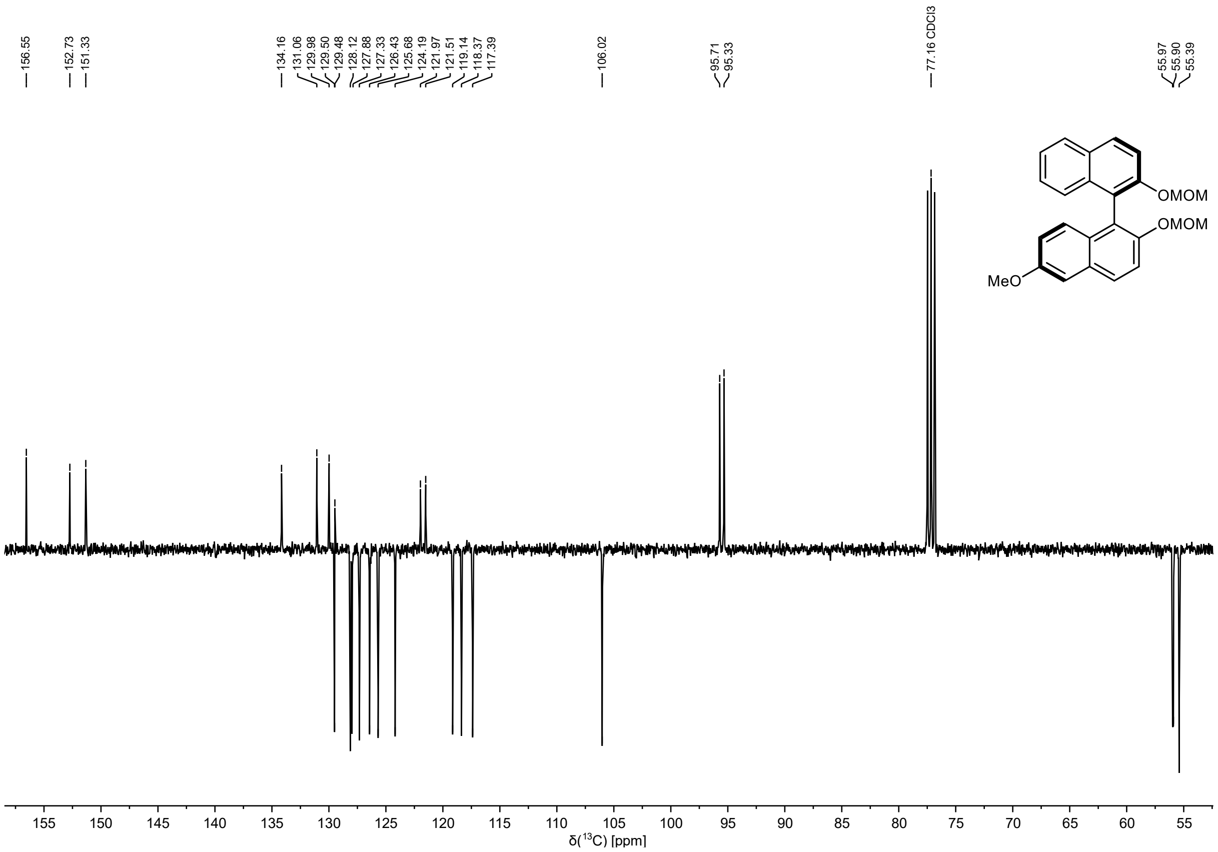

1H NMR (400 MHz, $CDCl_3$) **SI-23**

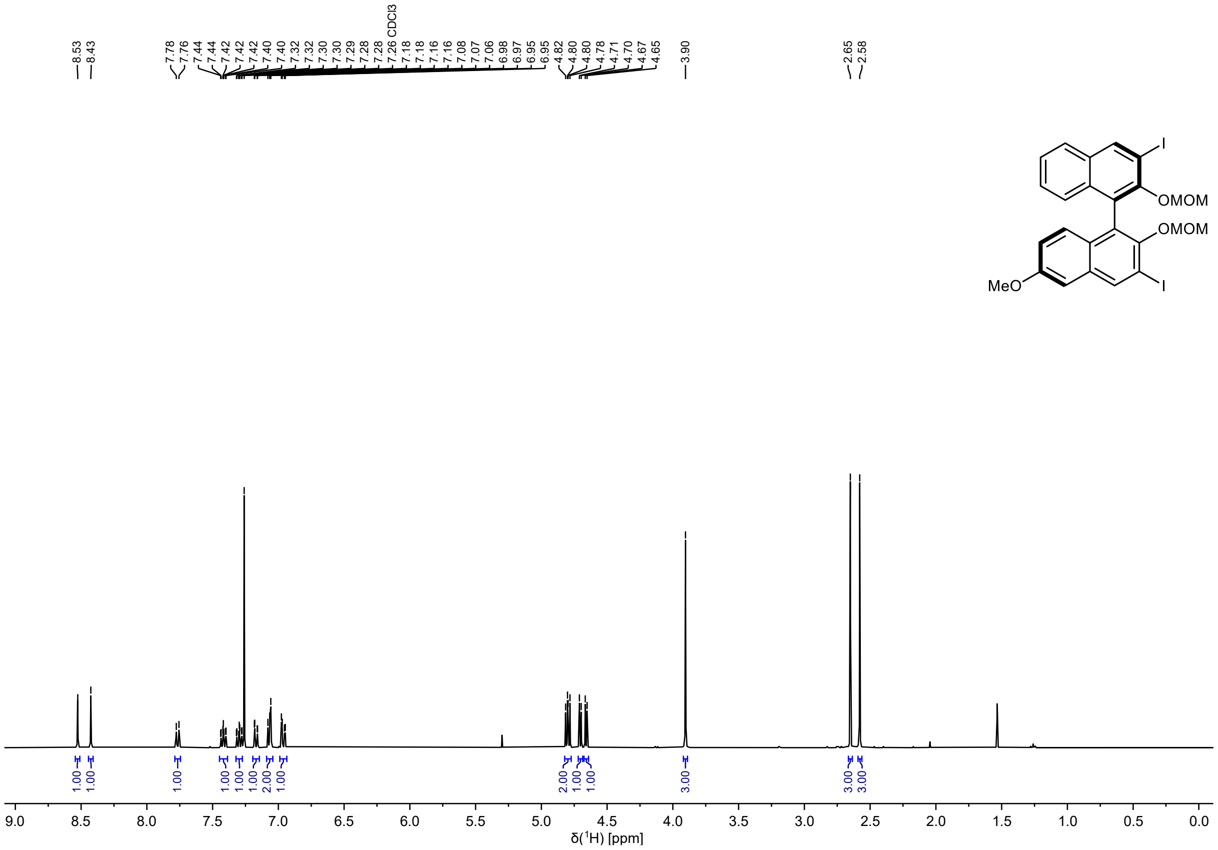

13C (APT) NMR (100 MHz, $CDCl_3$) **SI-23**

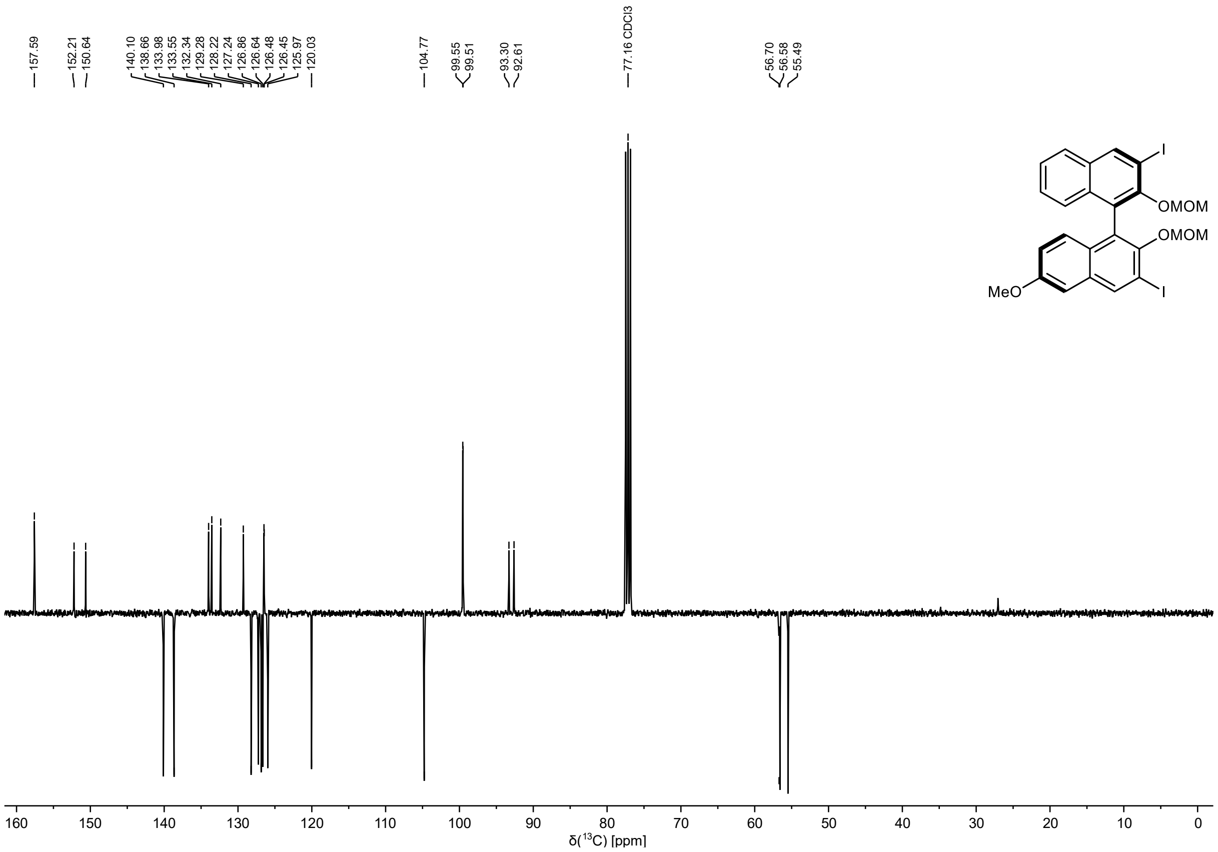

1H NMR (400 MHz, $CDCl_3$) **SI-26a**

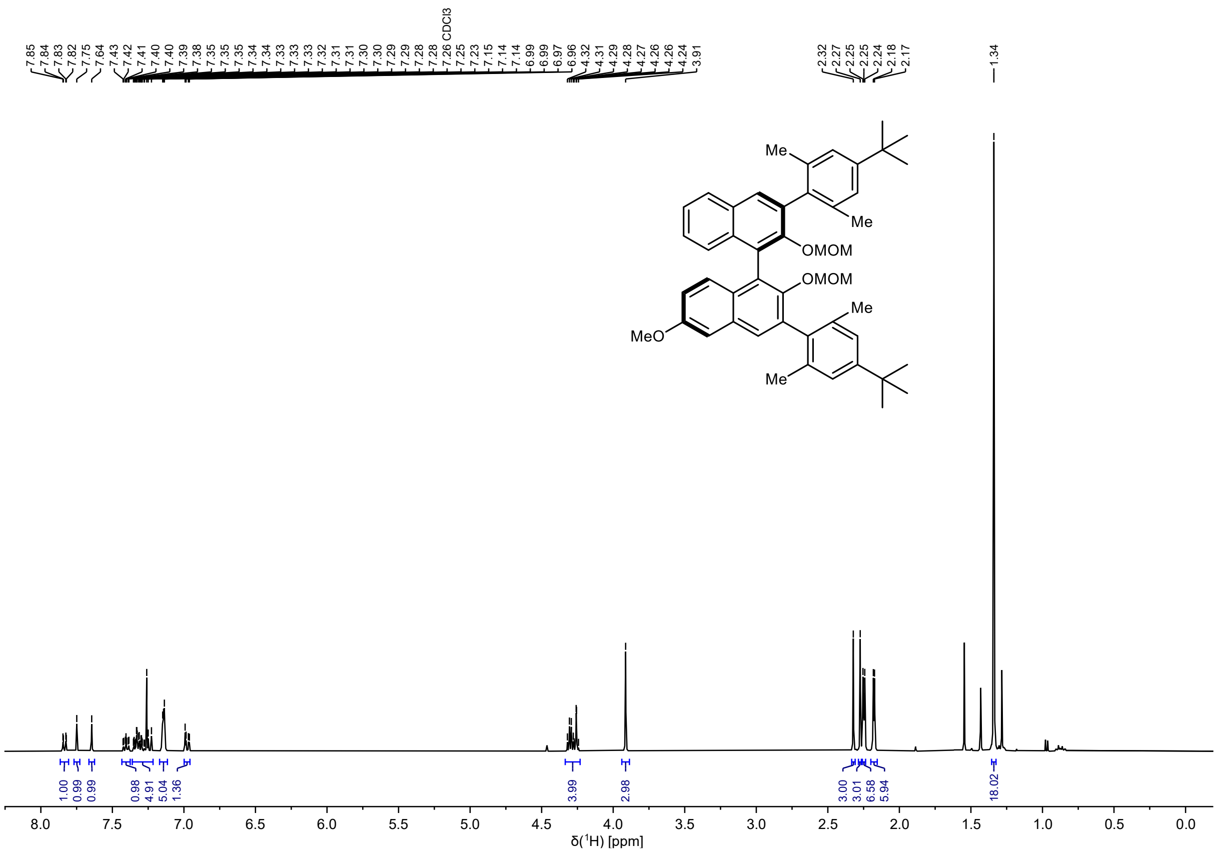

13C (APT) NMR (100 MHz, $CDCl_3$) **SI-26a**

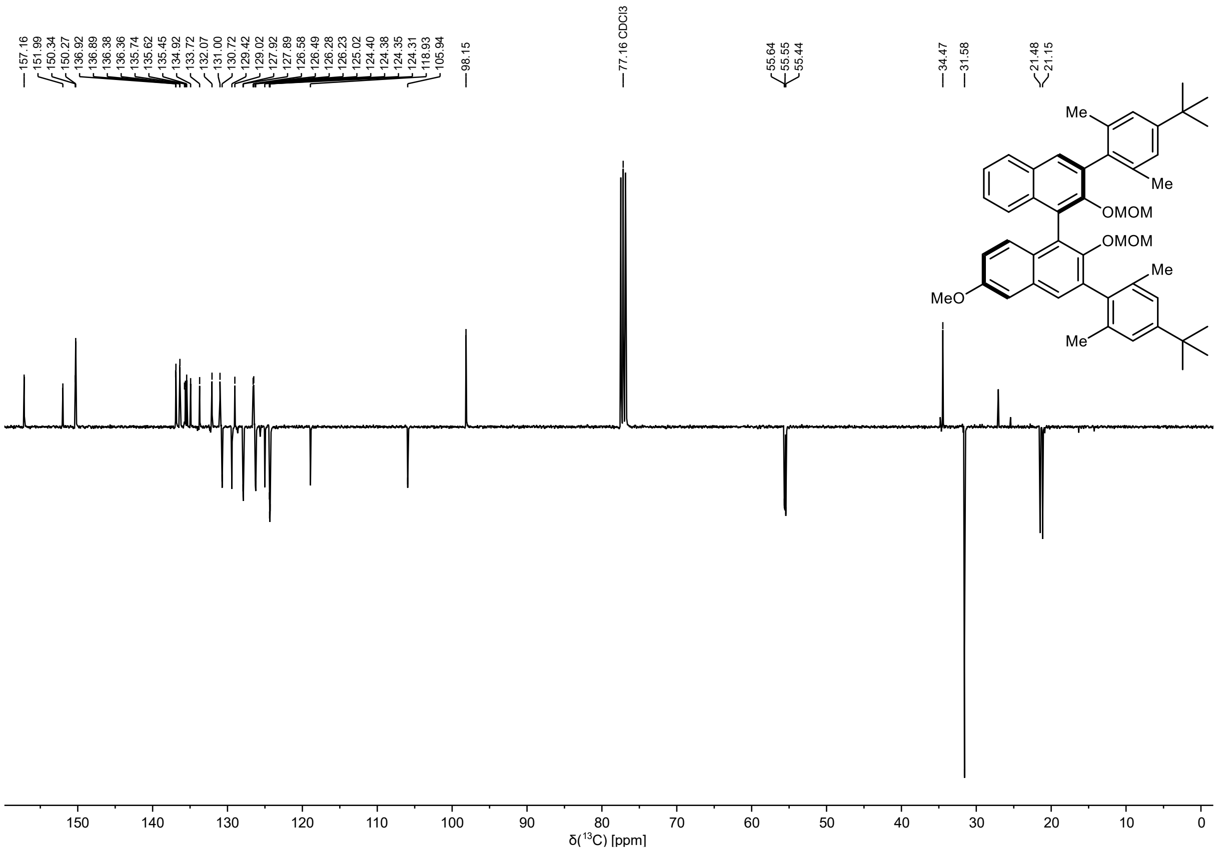

1H NMR (400 MHz, CDCl3) **SI-27a**

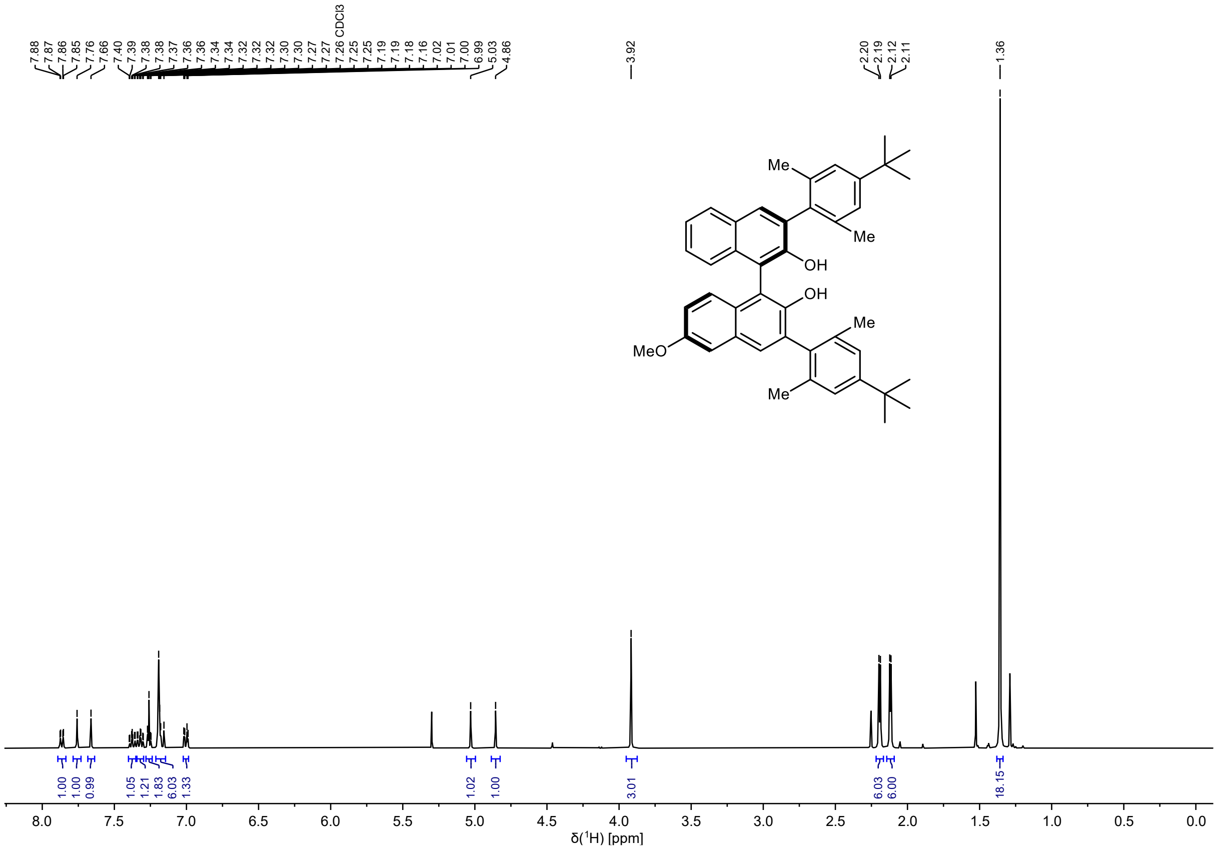

13C (APT) NMR (100 MHz, CDCl3) **SI-27a**

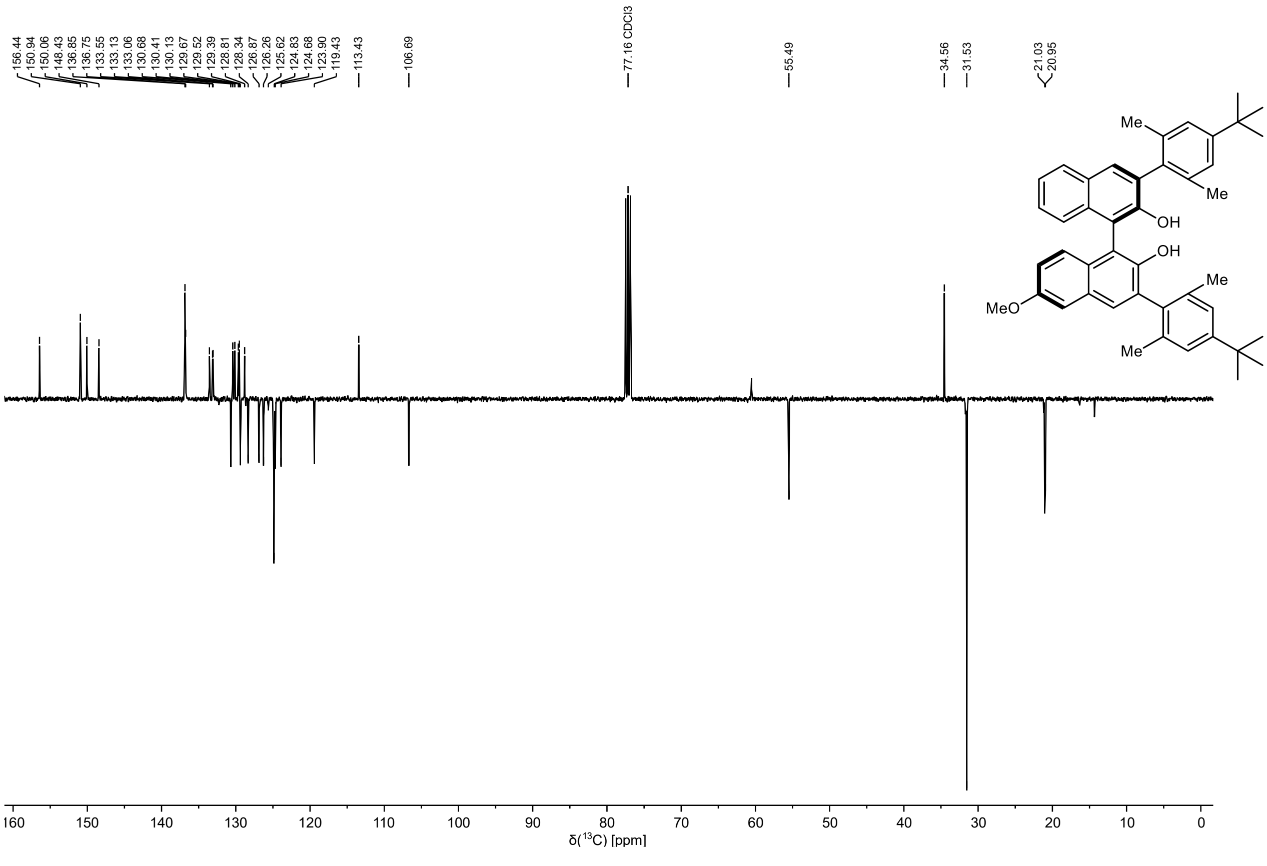

1H NMR (400 MHz, CDCl3) **SI-28a**

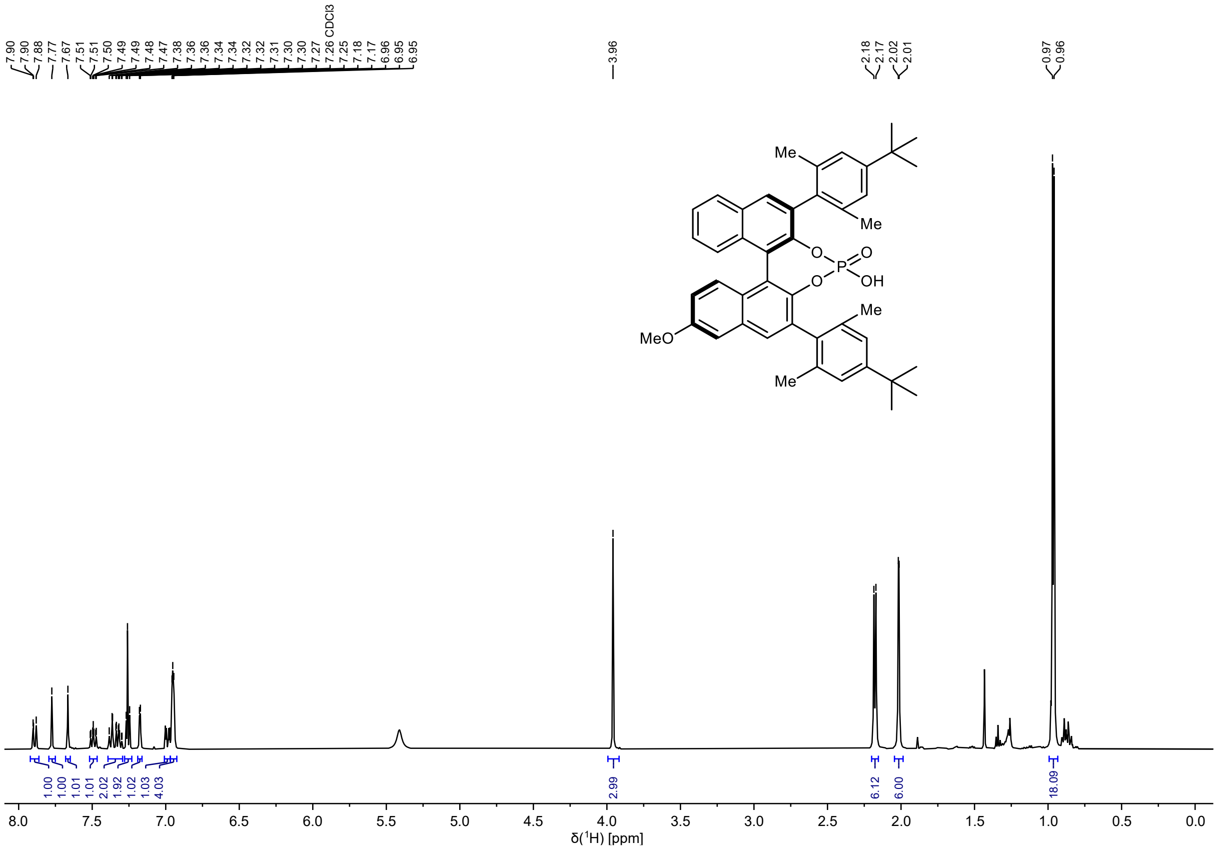

31P NMR (162 MHz, CDCl3) **SI-28a**

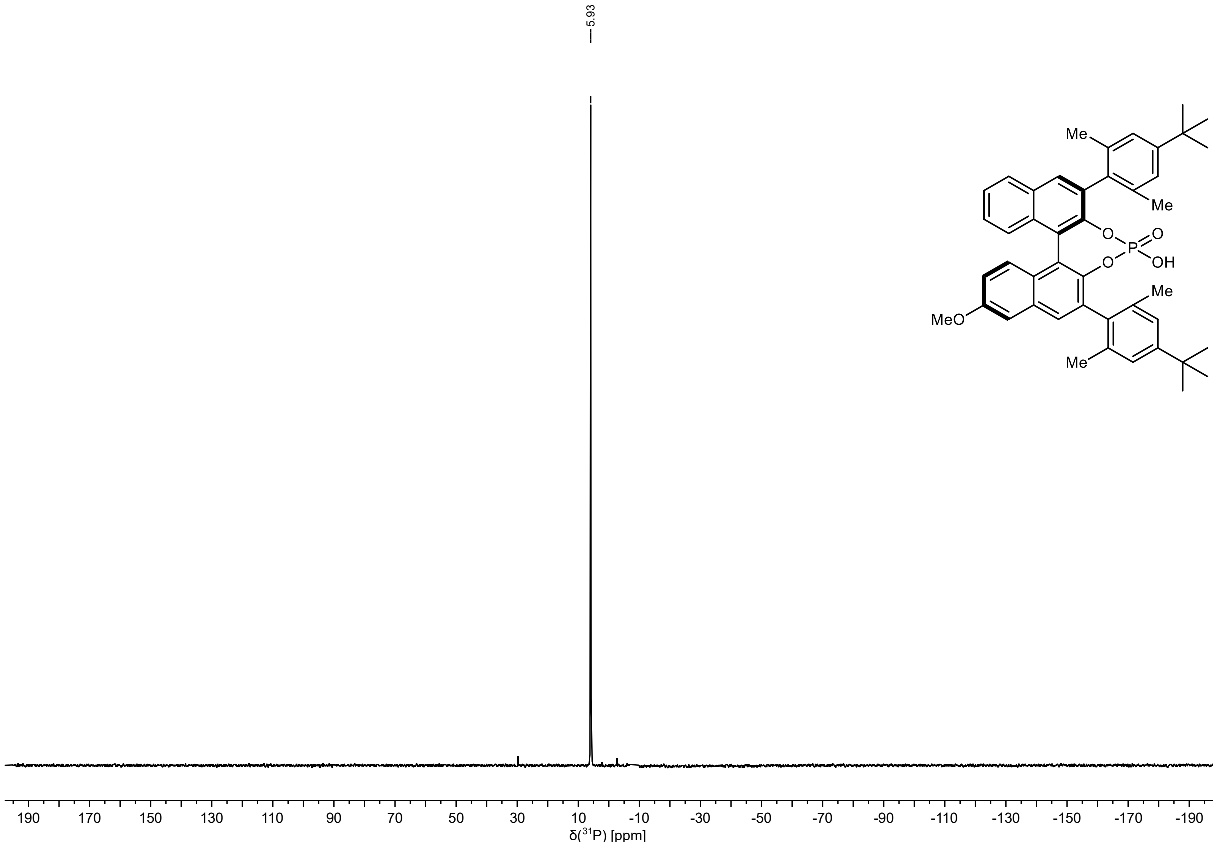

13C (APT) NMR (75 MHz, CDCl3) **SI-28a**

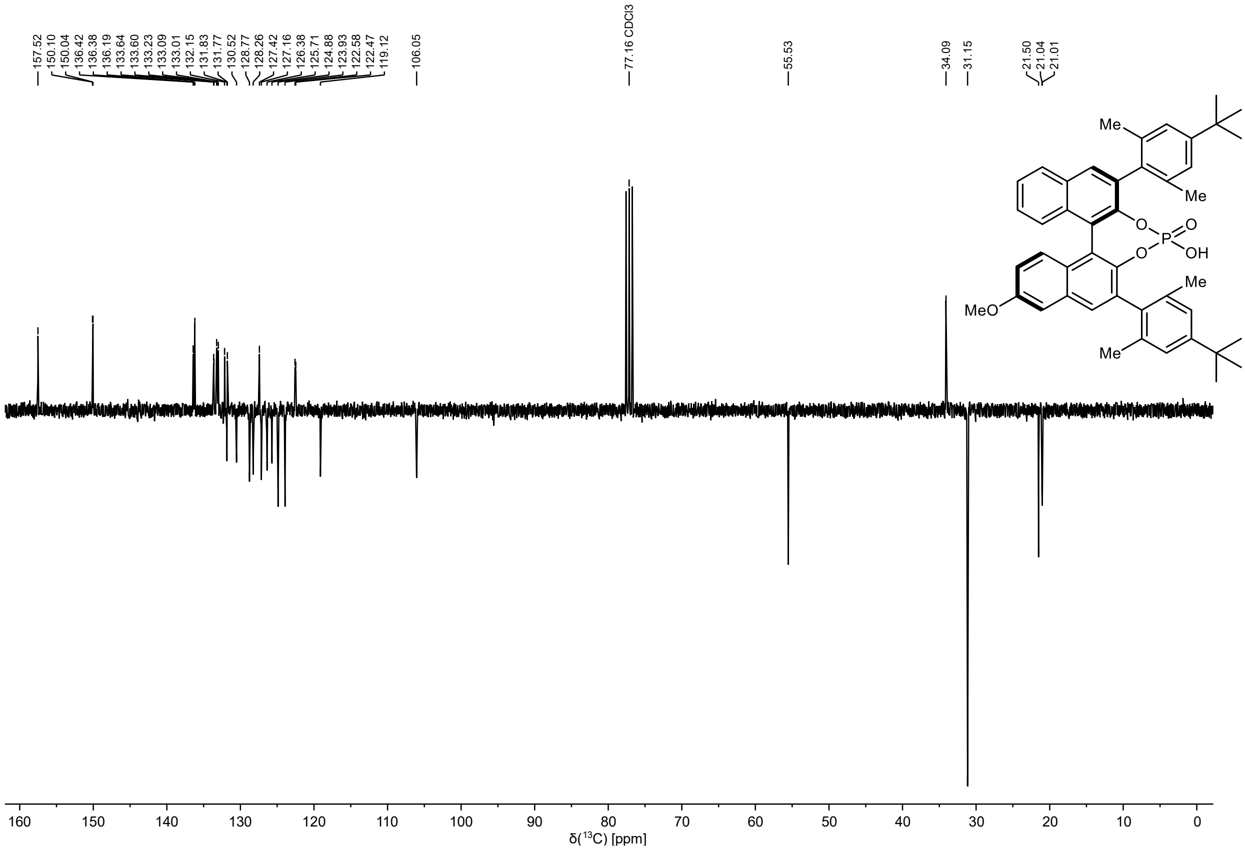

1H NMR (300 MHz, CDCl3) **SI-29a**

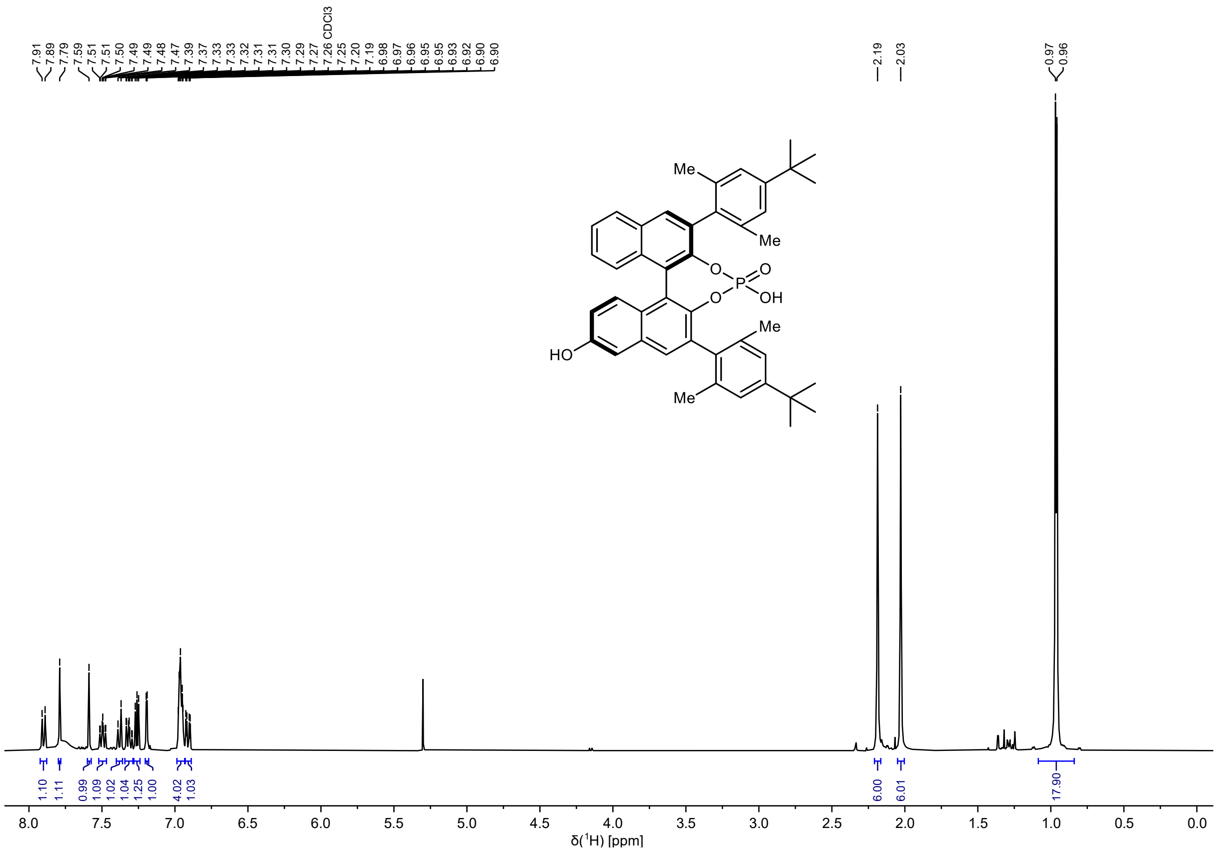

31P NMR (162 MHz, CDCl3) **SI-29a**

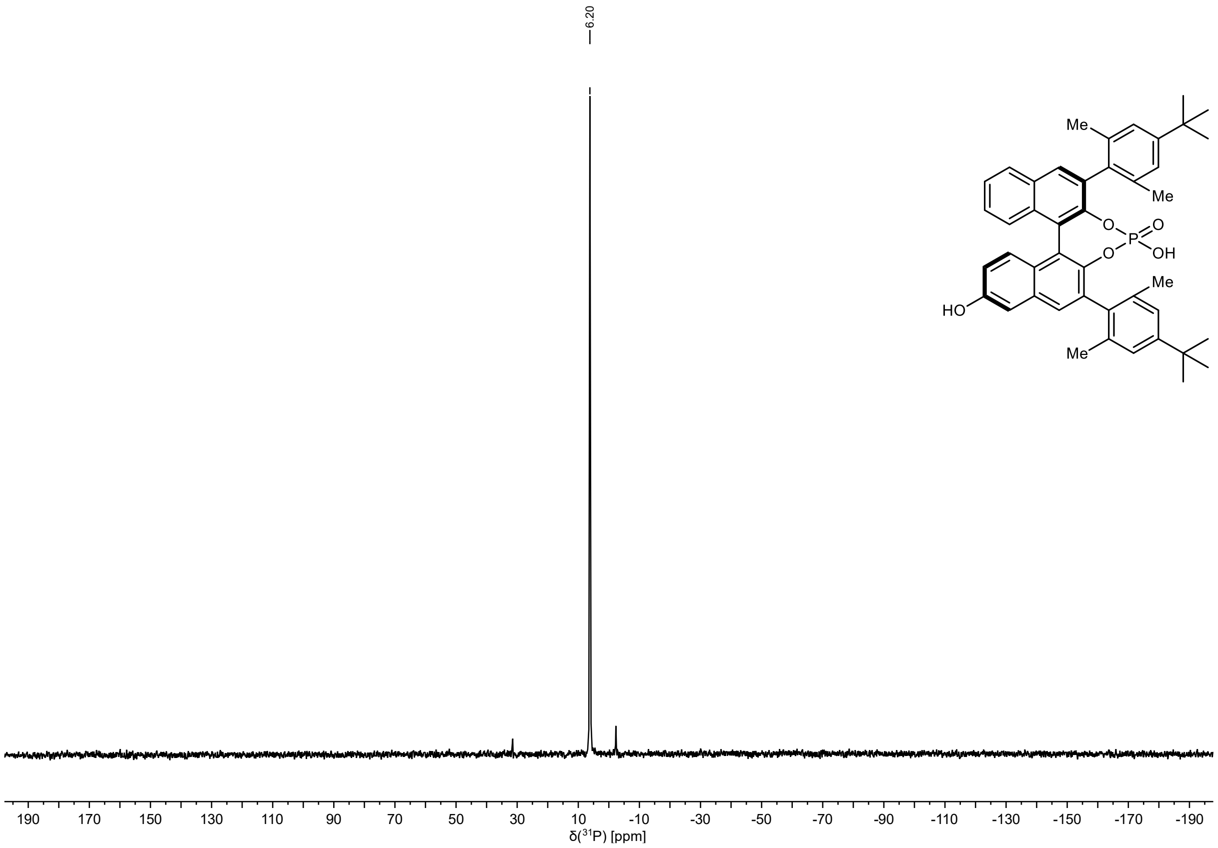

13C (APT) NMR (100 MHz, CDCl3) **SI-29a**

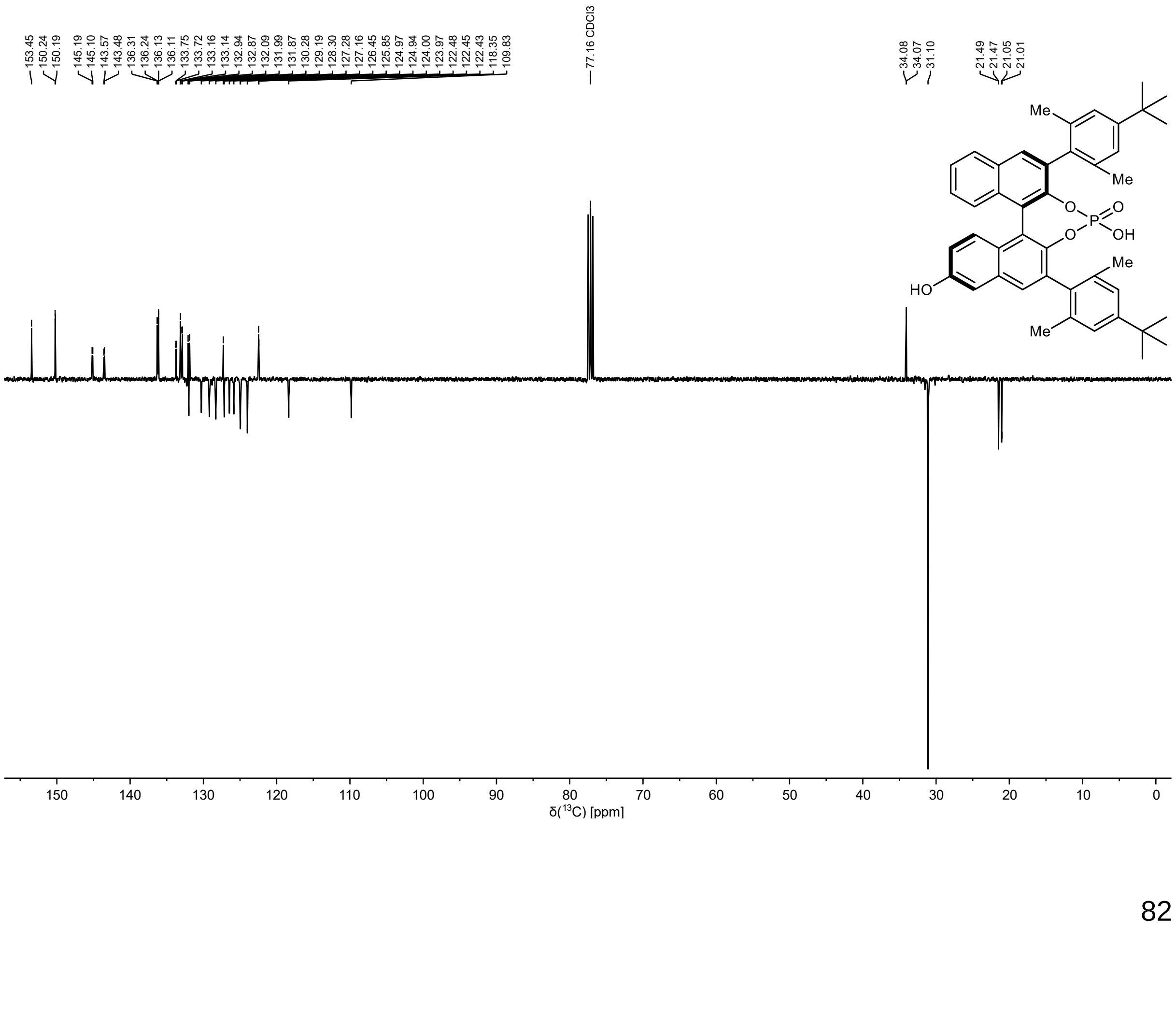

1H NMR (400 MHz, CDCl3) **SI-30a**

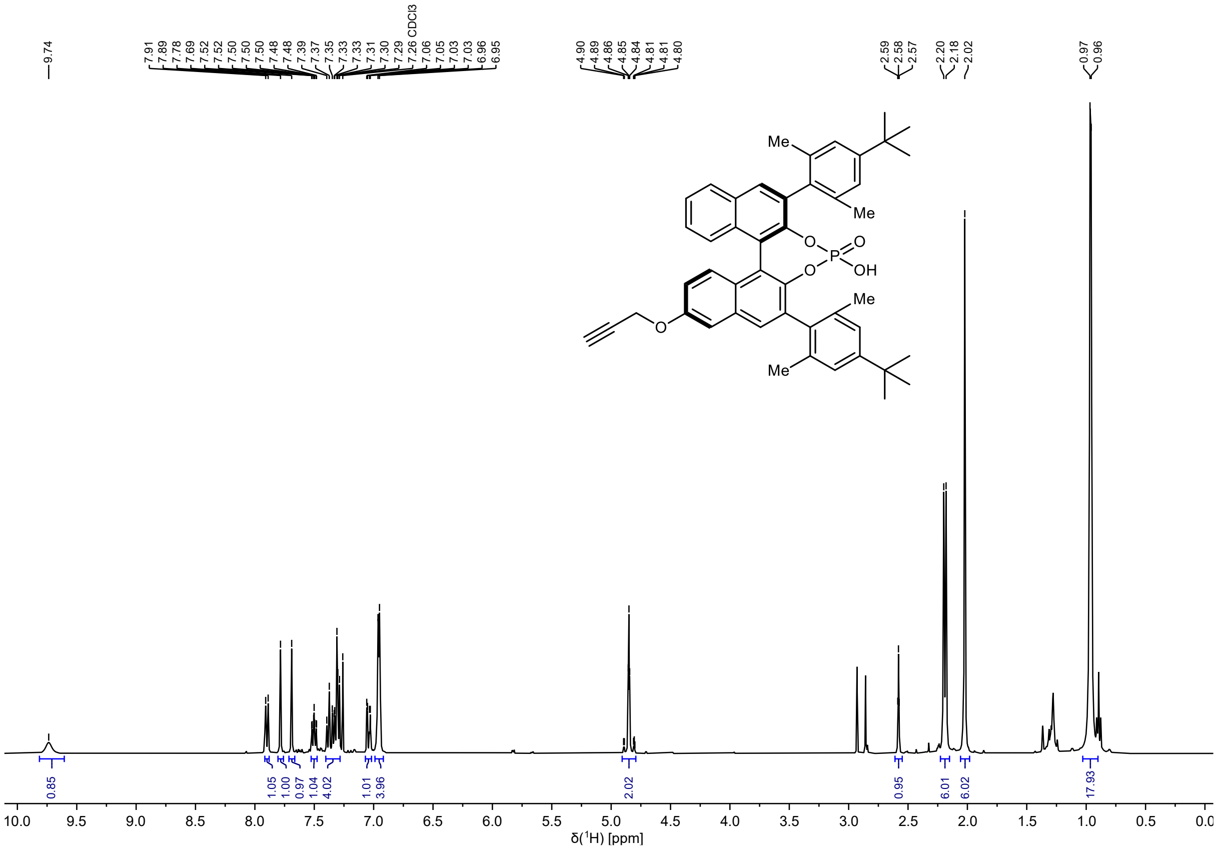

31P NMR (162 MHz, CDCl3) **SI-30a**

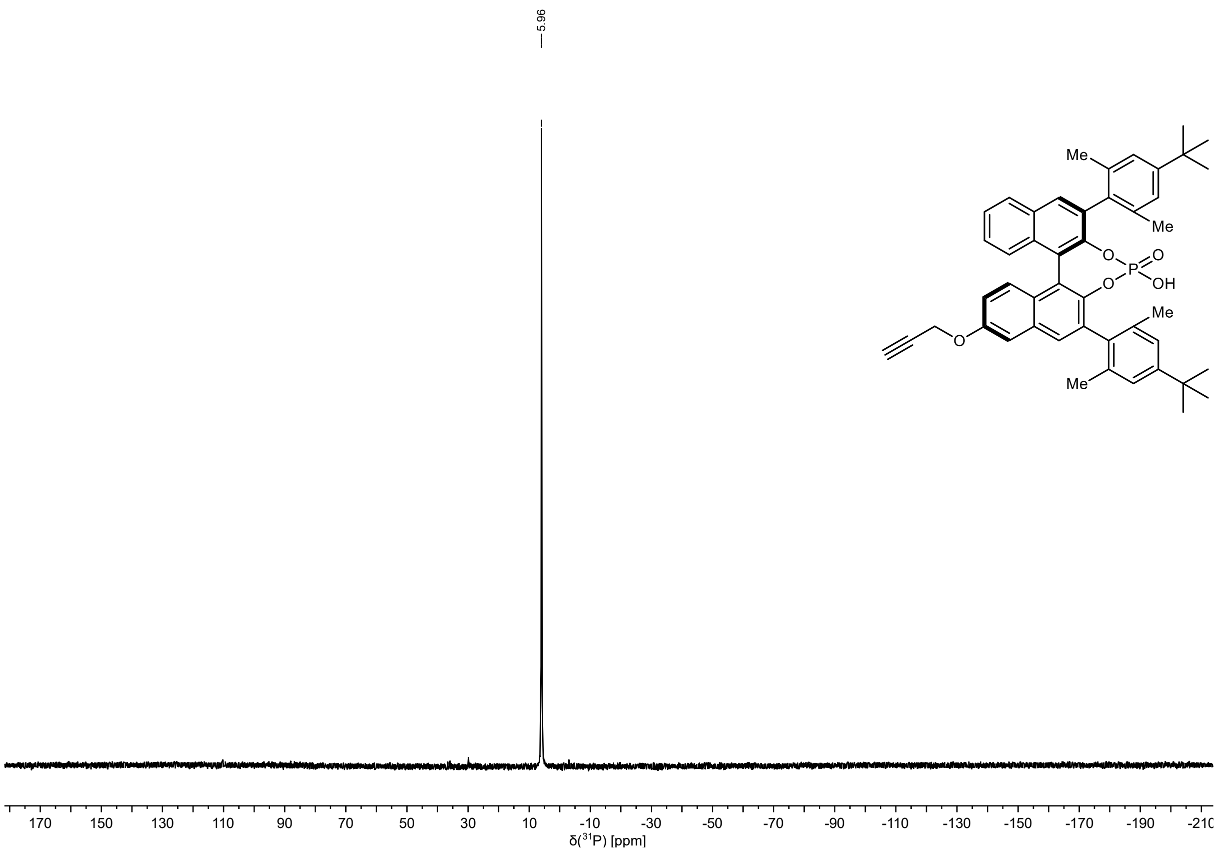

13C (APT) NMR (100 MHz, $CDCl_3$) **SI-30a**

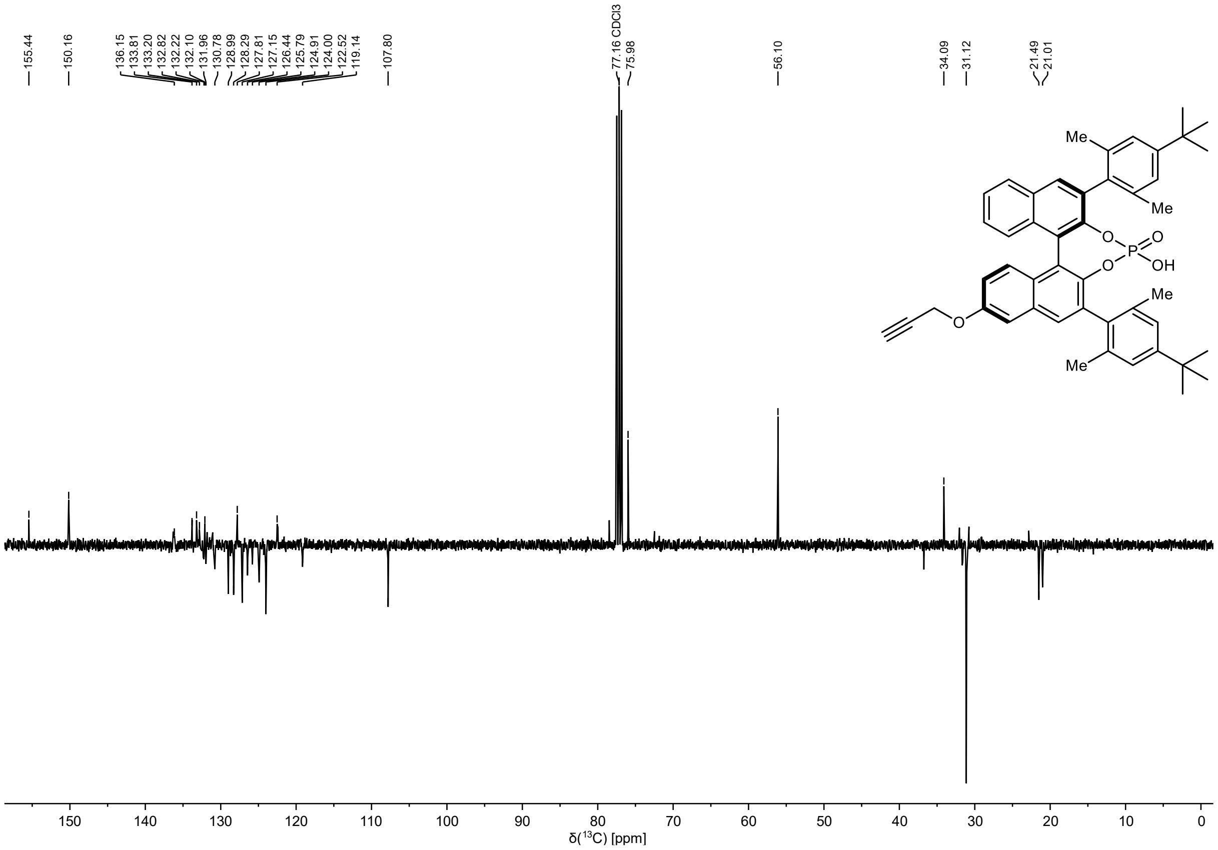

1H NMR (400 MHz, $CDCl_3$) **SI-3b**

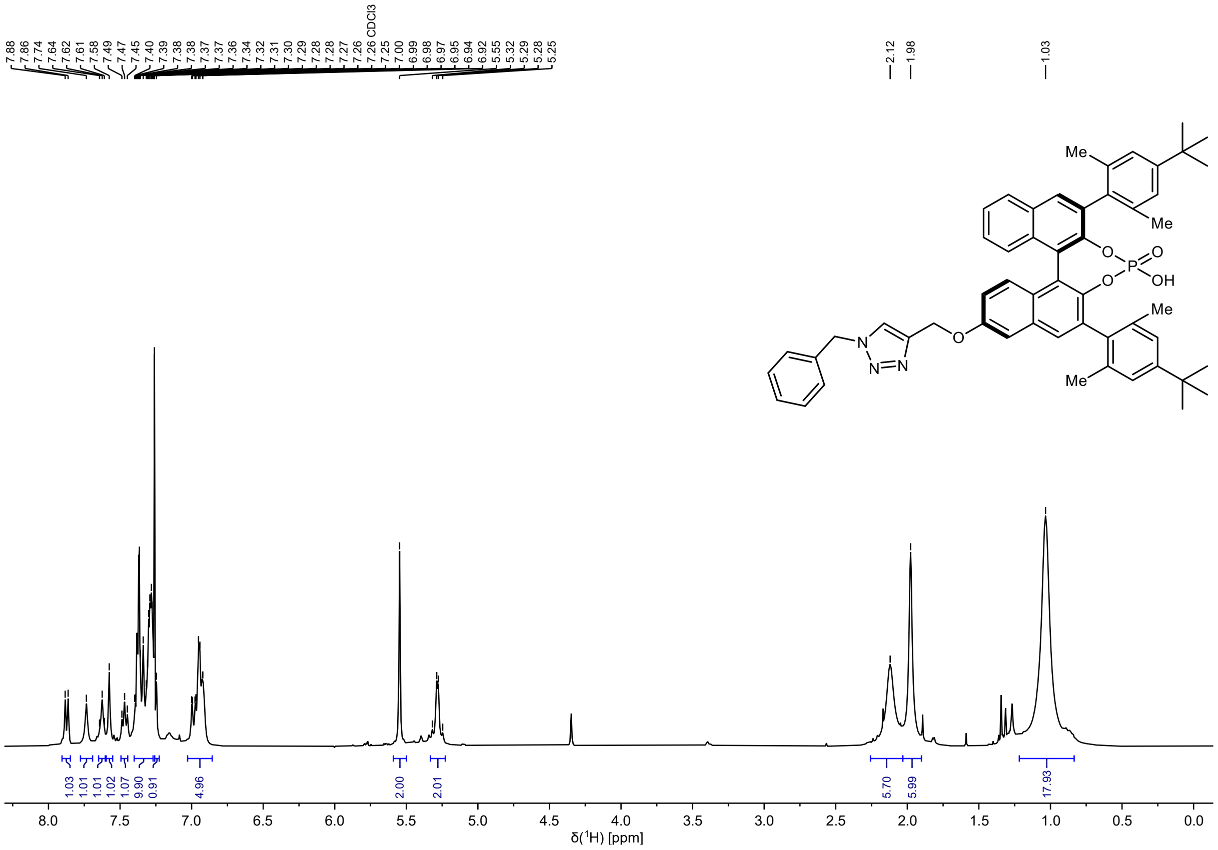

31P NMR (162 MHz, CDCl3) **SI-3b**

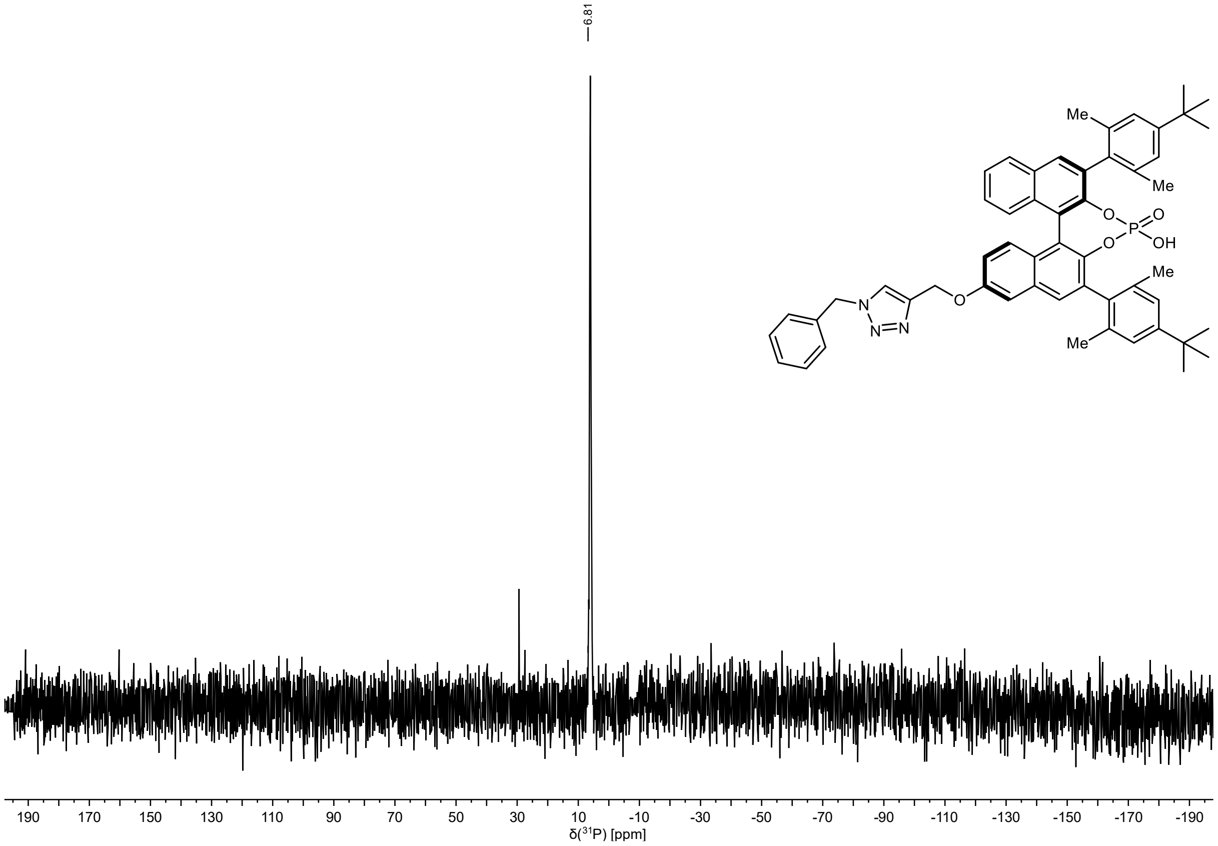

13C (APT) NMR (100 MHz, CDCl3) **SI-3b**

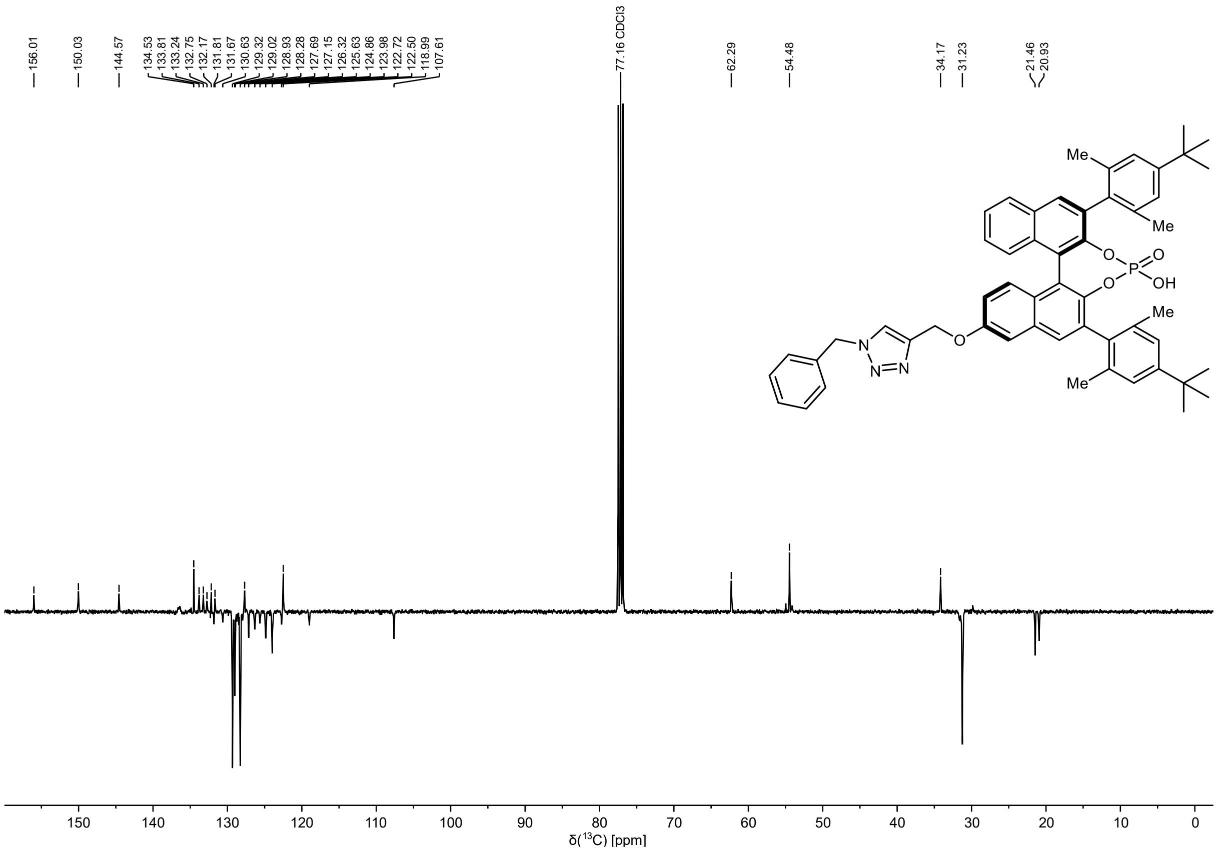

1H NMR (300 MHz, CDCl3) **SI-26c**

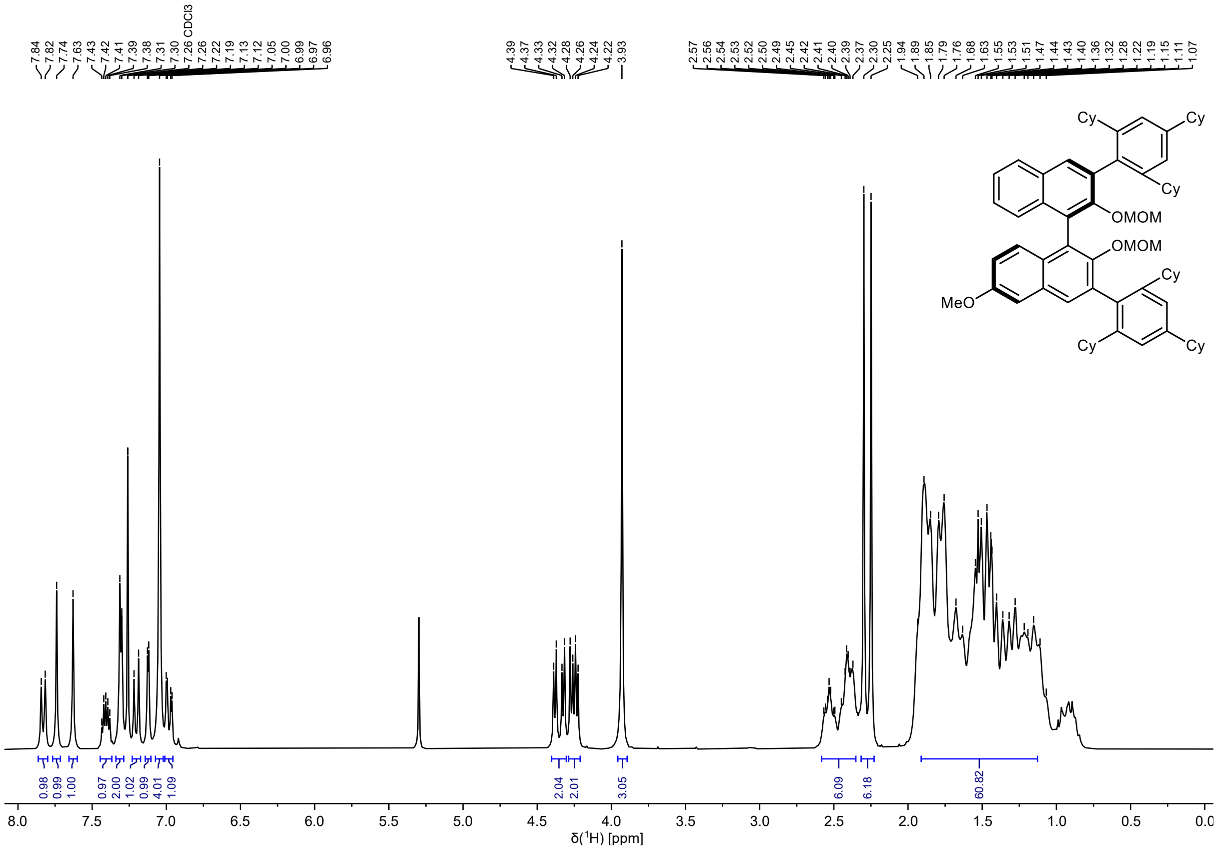

13C (APT) NMR (75 MHz, CDCl3) **SI-26c**

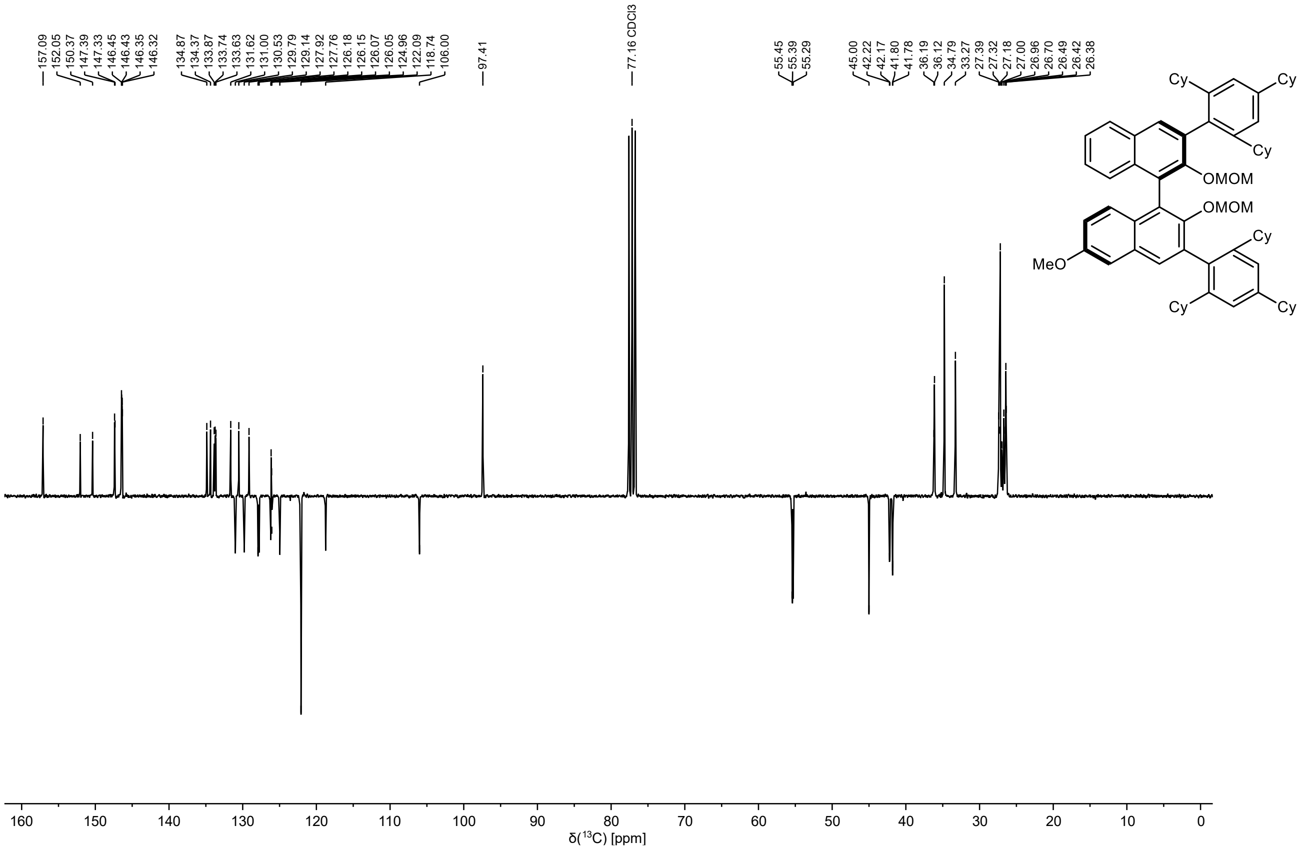

1H NMR (400 MHz, $CDCl_3$) **SI-27c**

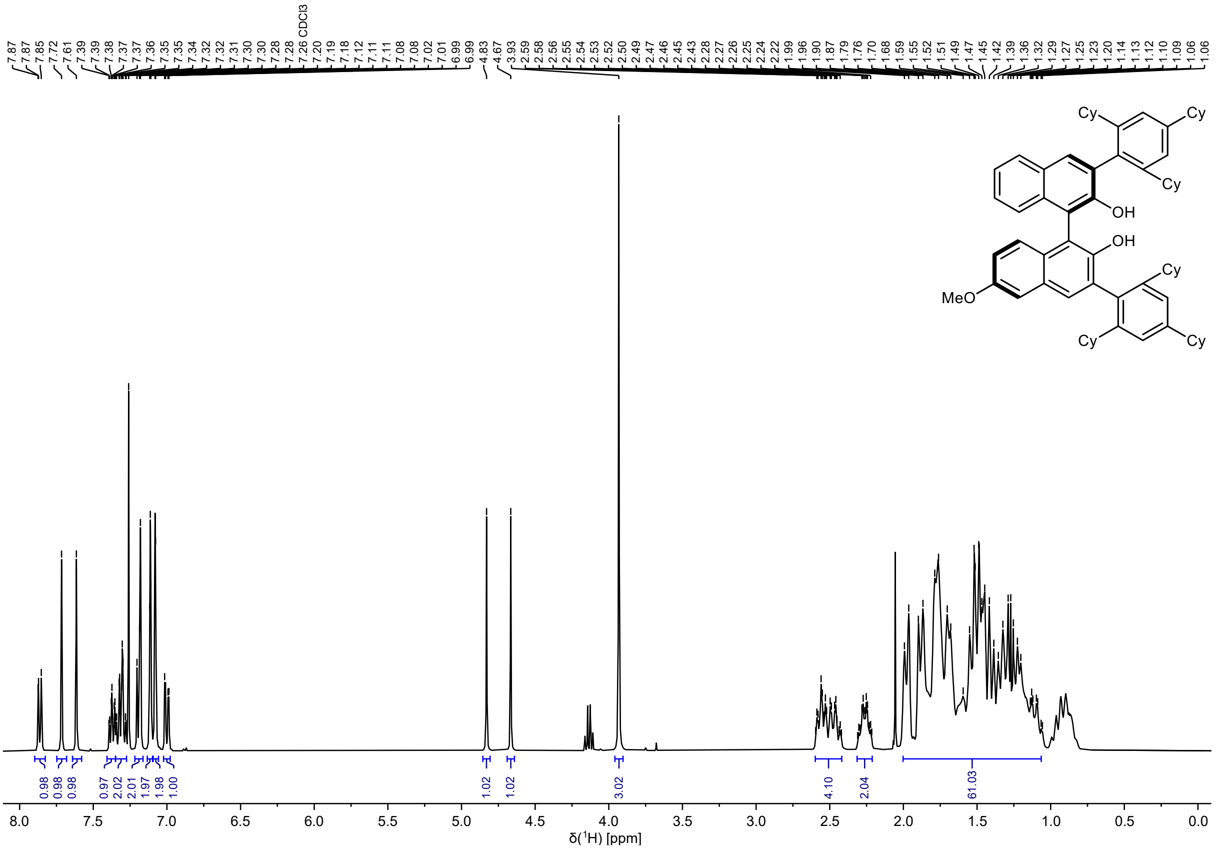

13C (APT) NMR (100 MHz, $CDCl_3$) **SI-27c**

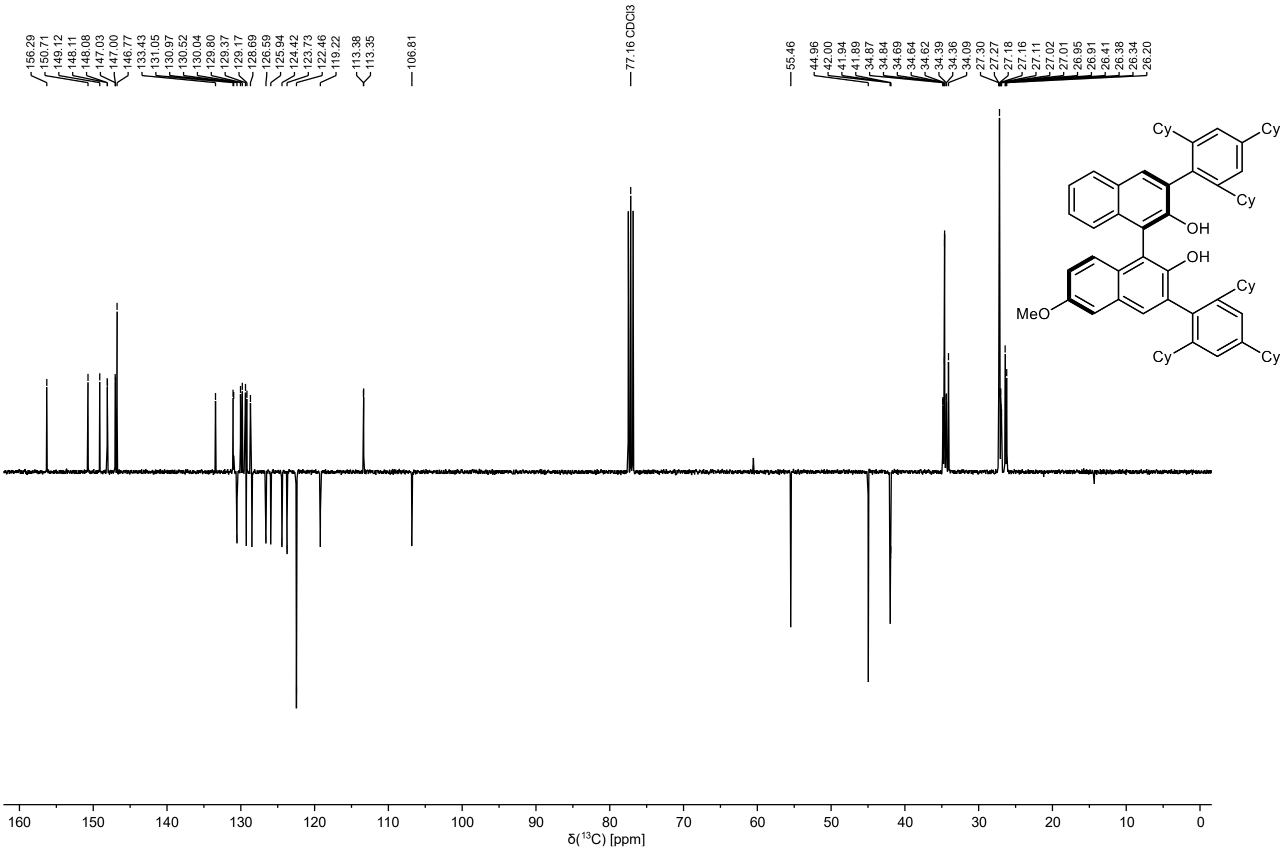

1H NMR (400 MHz, CDCl3) **SI-28c**

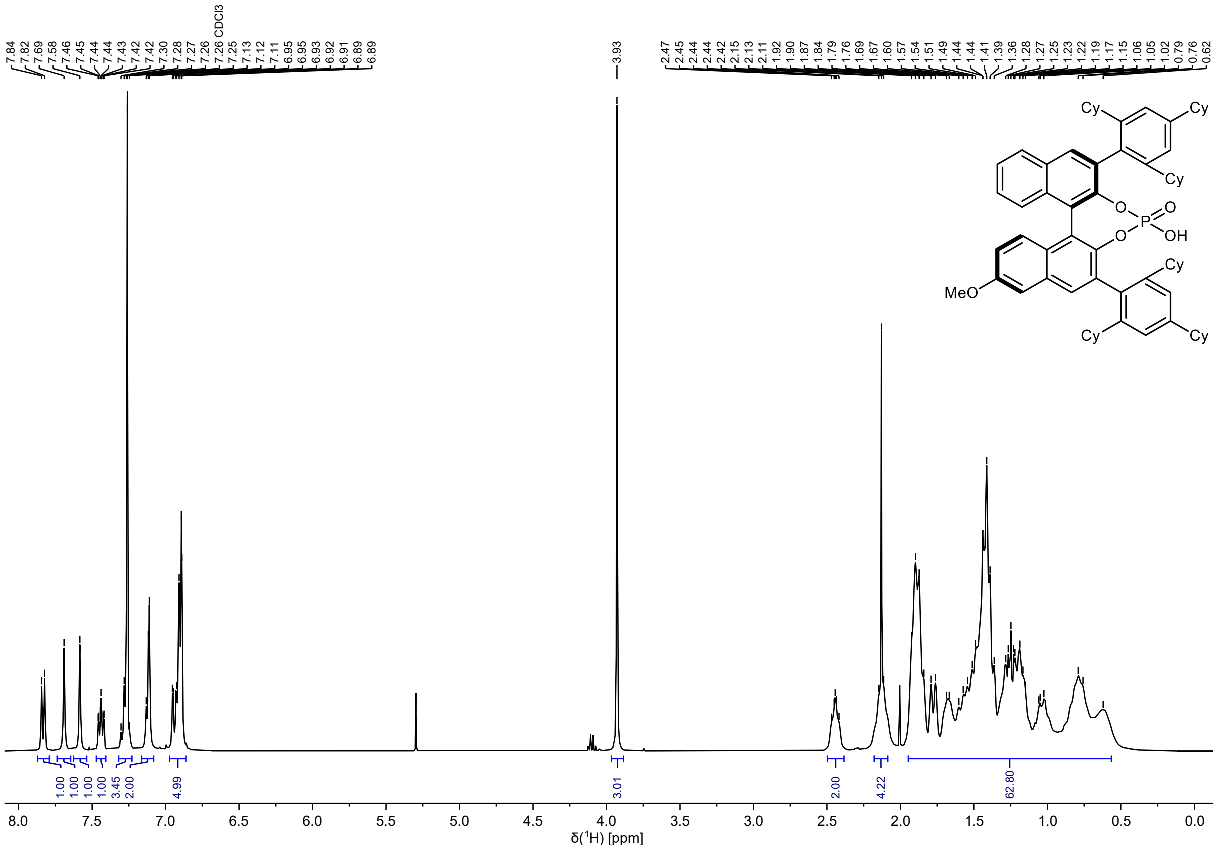

31P NMR (162 MHz, CDCl3) **SI-28c**

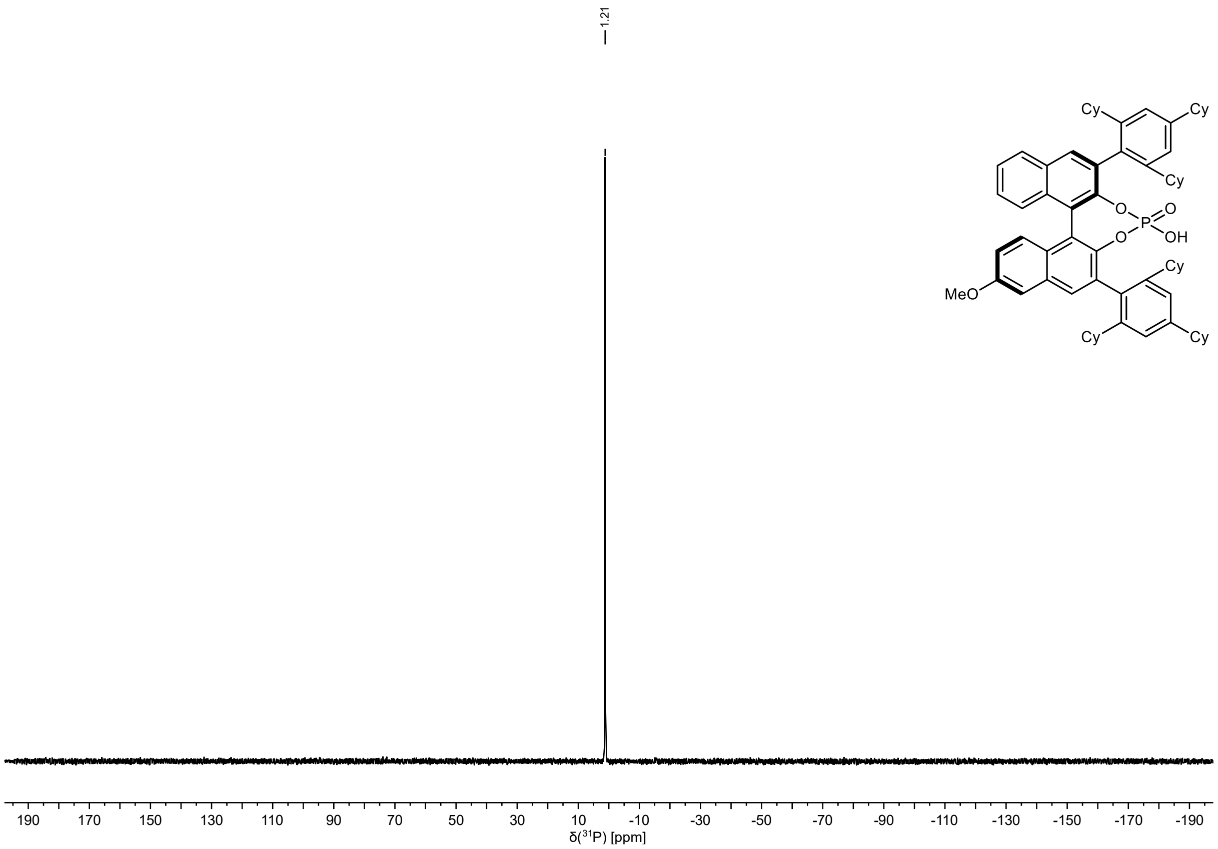

13C (APT) NMR (100 MHz, $CDCl_3$) **SI-28c**

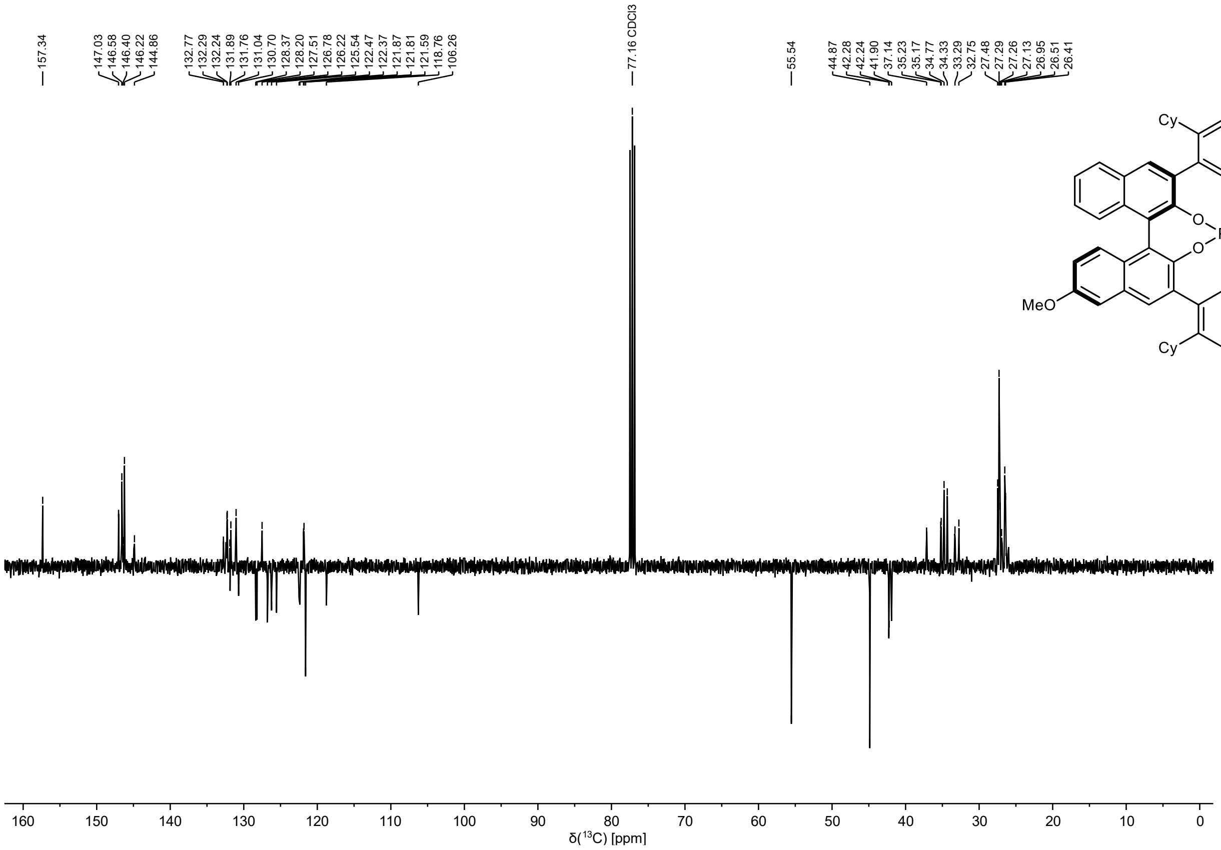

1H NMR (400 MHz, $CDCl_3$) **SI-29c**

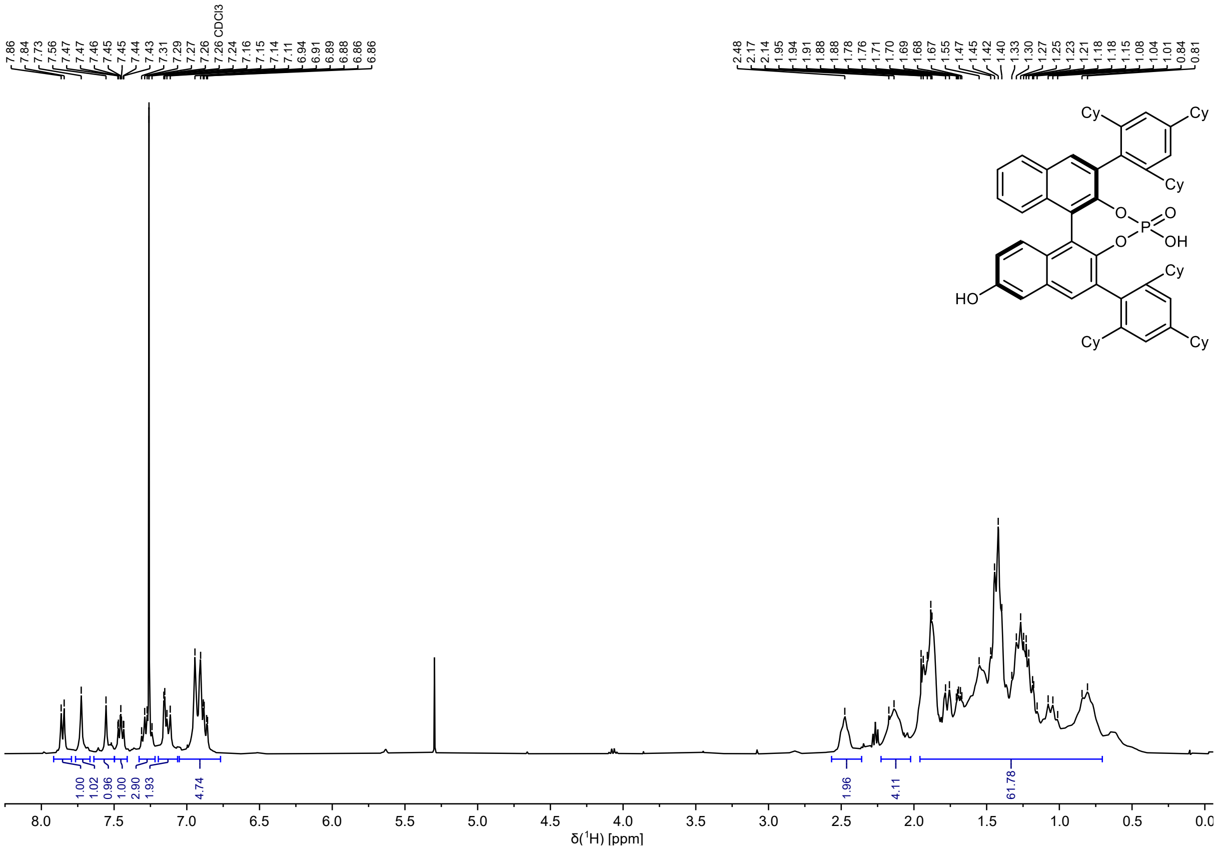

31P NMR (162 MHz, CDCl3) **SI-29c**

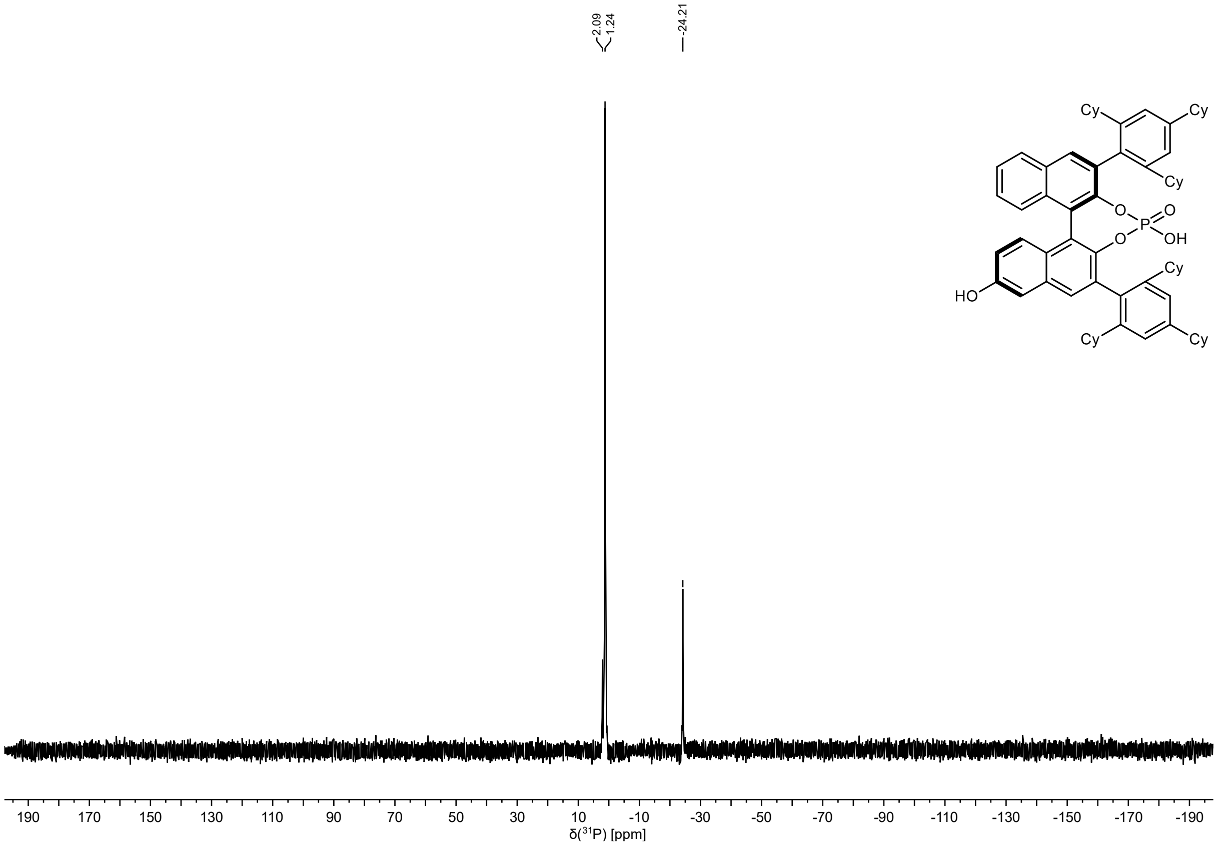

13C (APT) NMR (100 MHz, CDCl3) **SI-29c**

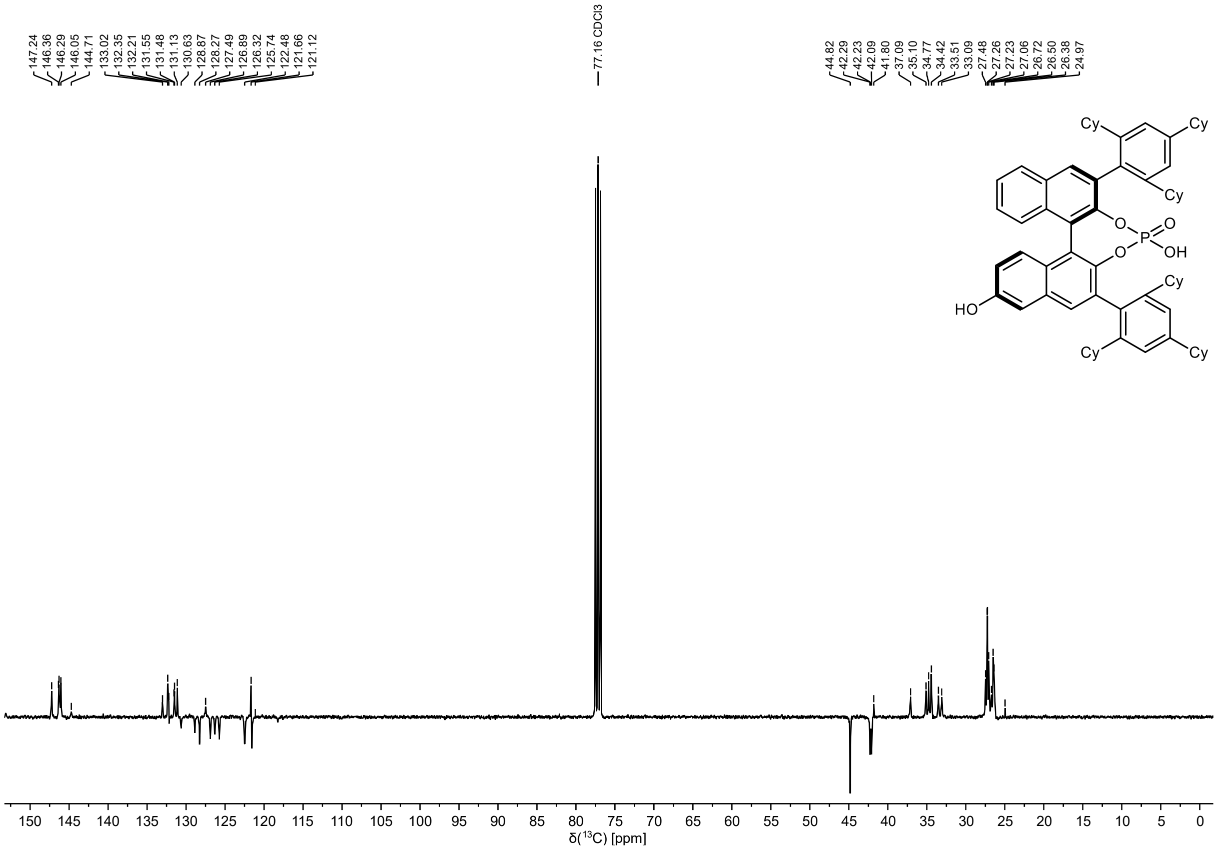

1H NMR (400 MHz, DMSO-d6) **SI-30c**

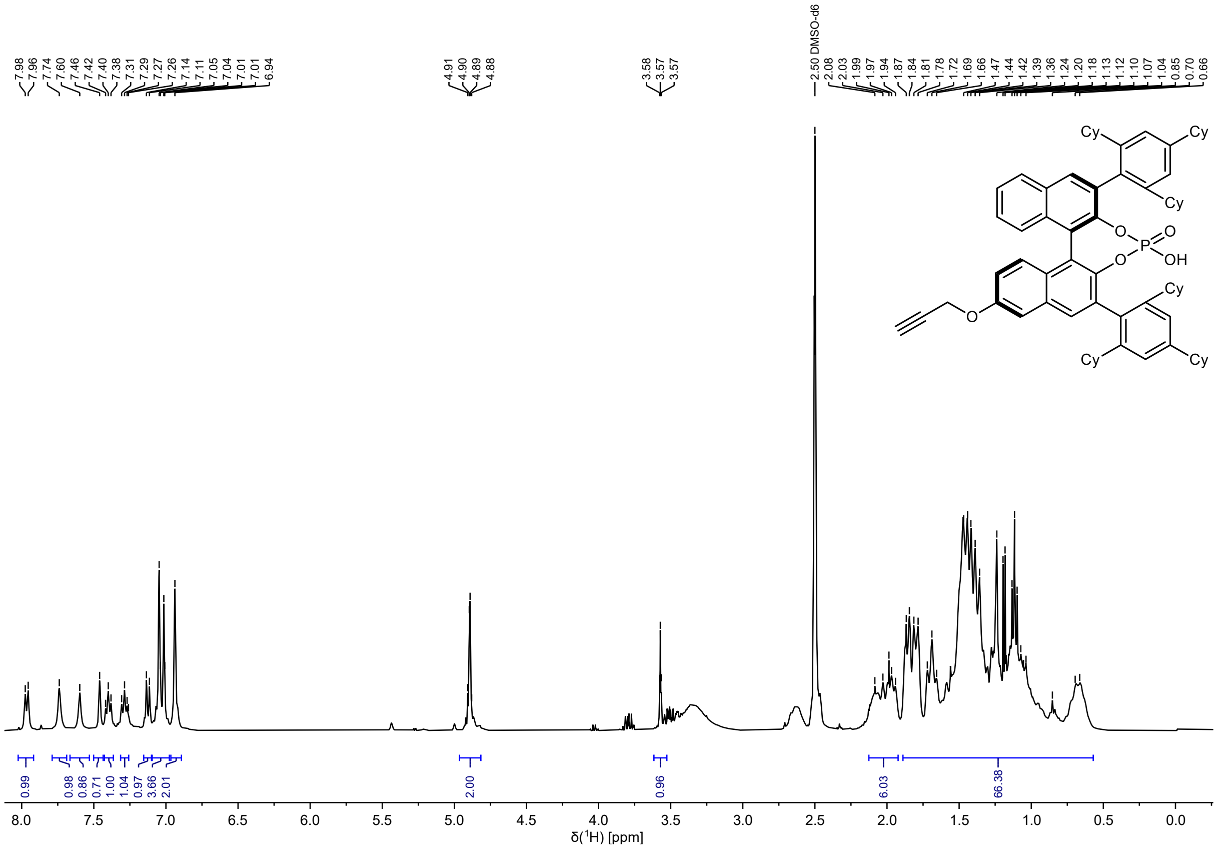

31P NMR (162 MHz, DMSO-d6) **SI-30c**

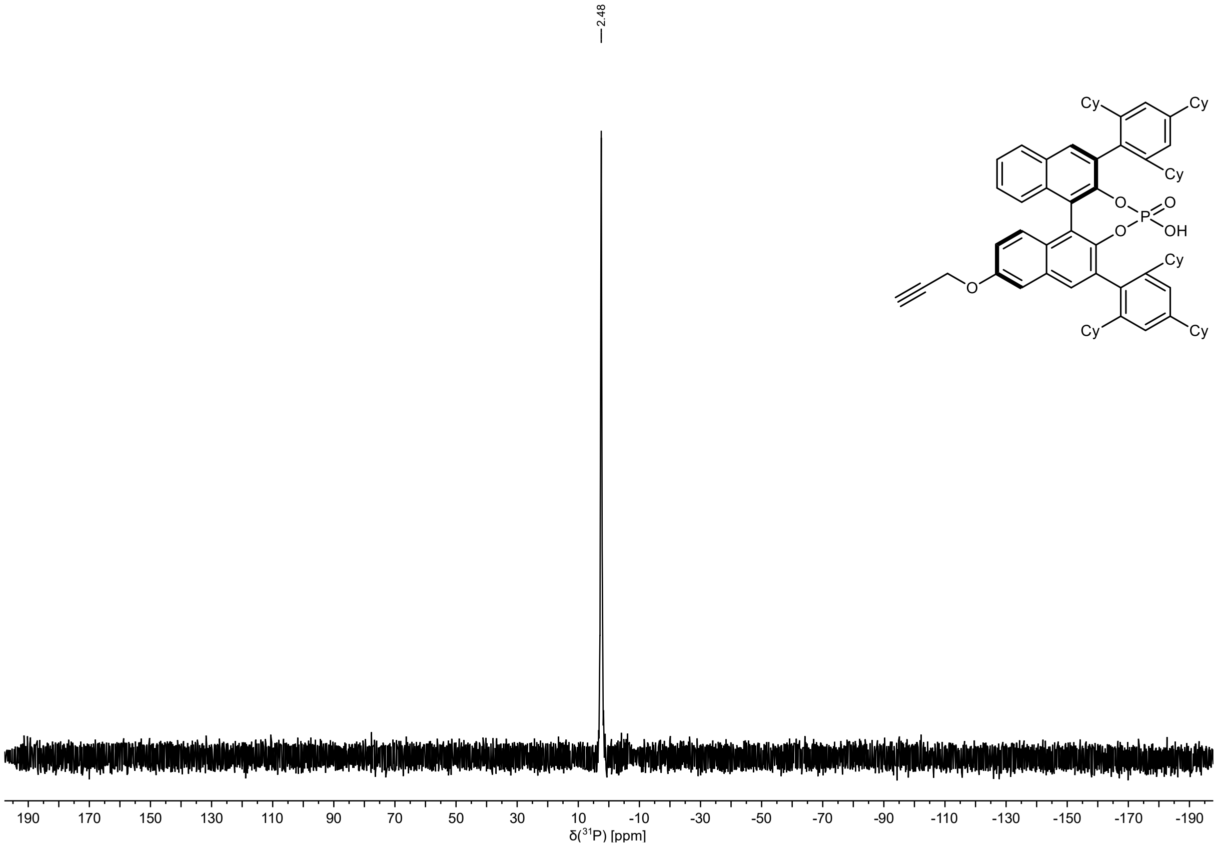

13C (APT) NMR (100 MHz, DMSO-d6) **SI-30c**

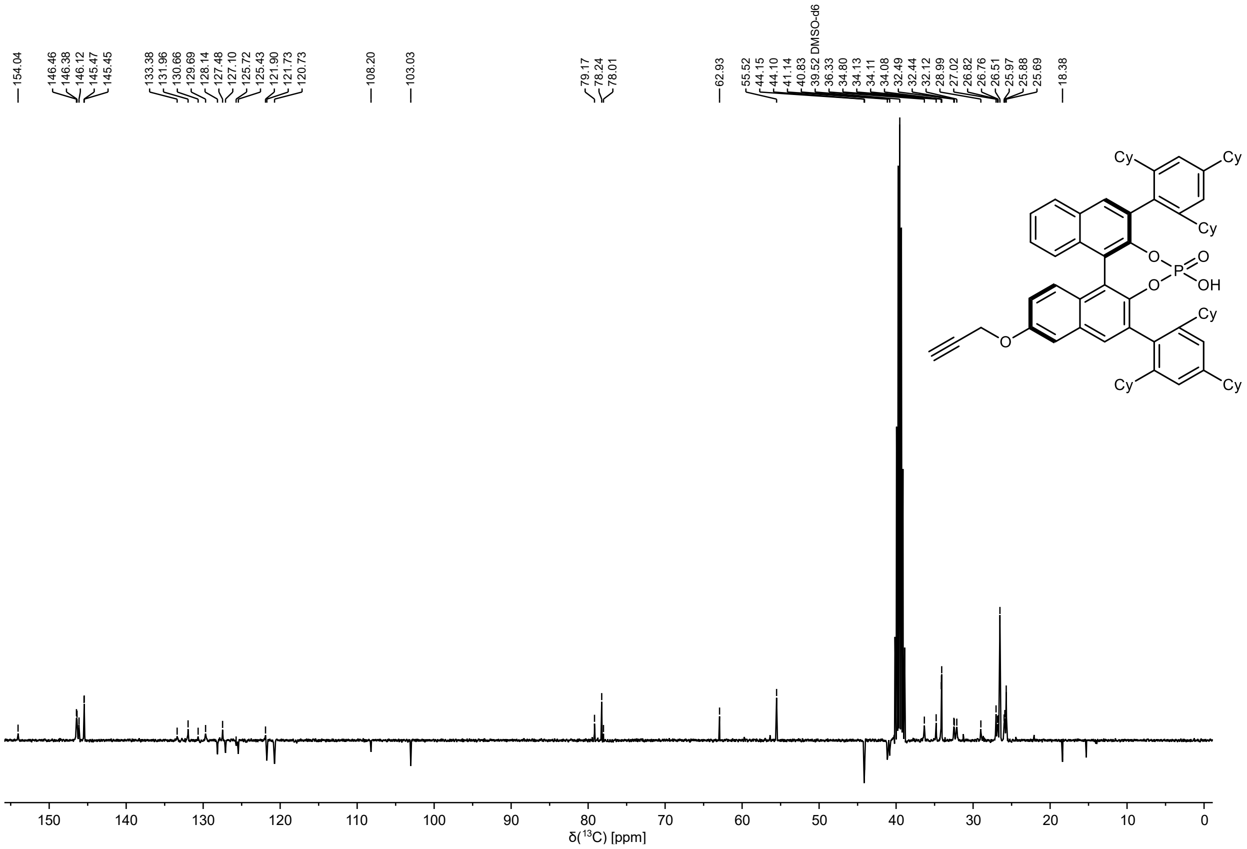

1H NMR (400 MHz, DMSO-d6) **SI-25**

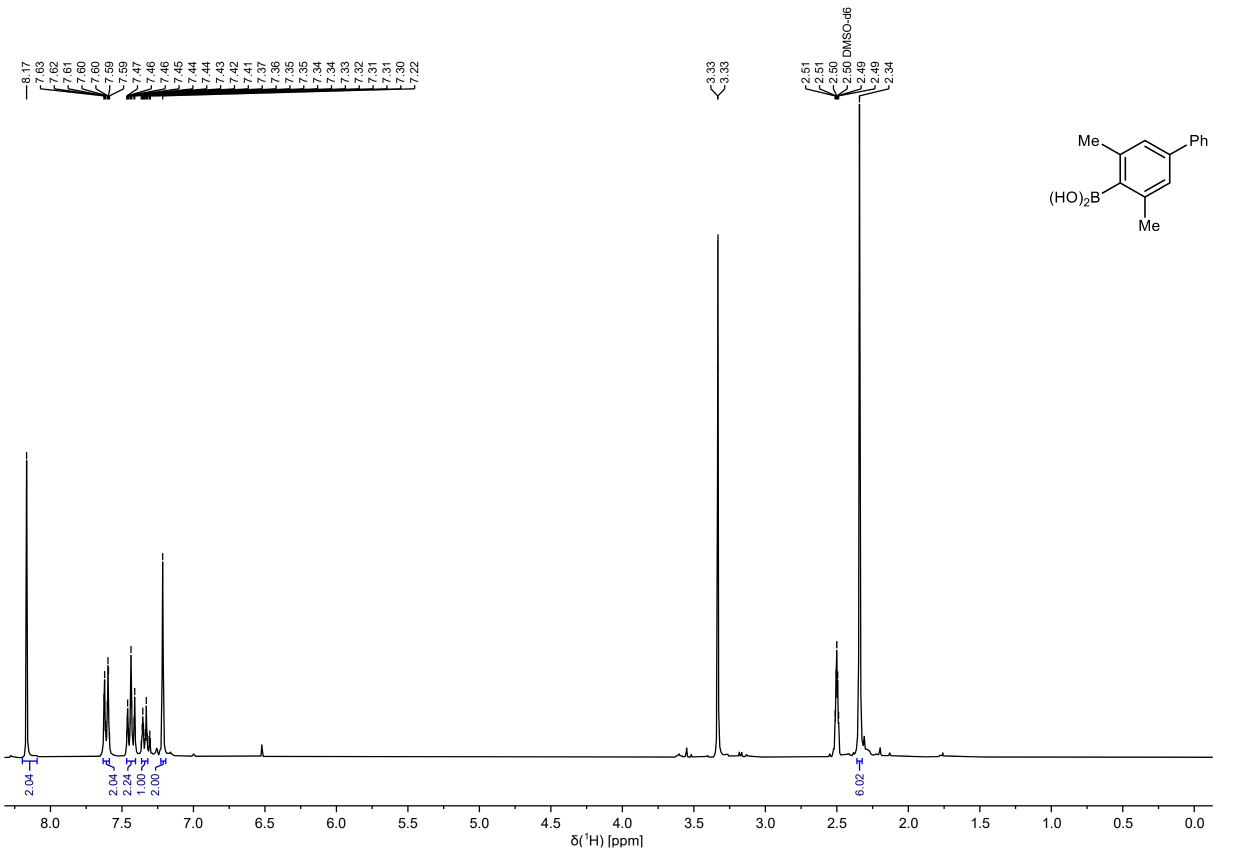

13C (APT) NMR (100 MHz, DMSO-d6) **SI-25**

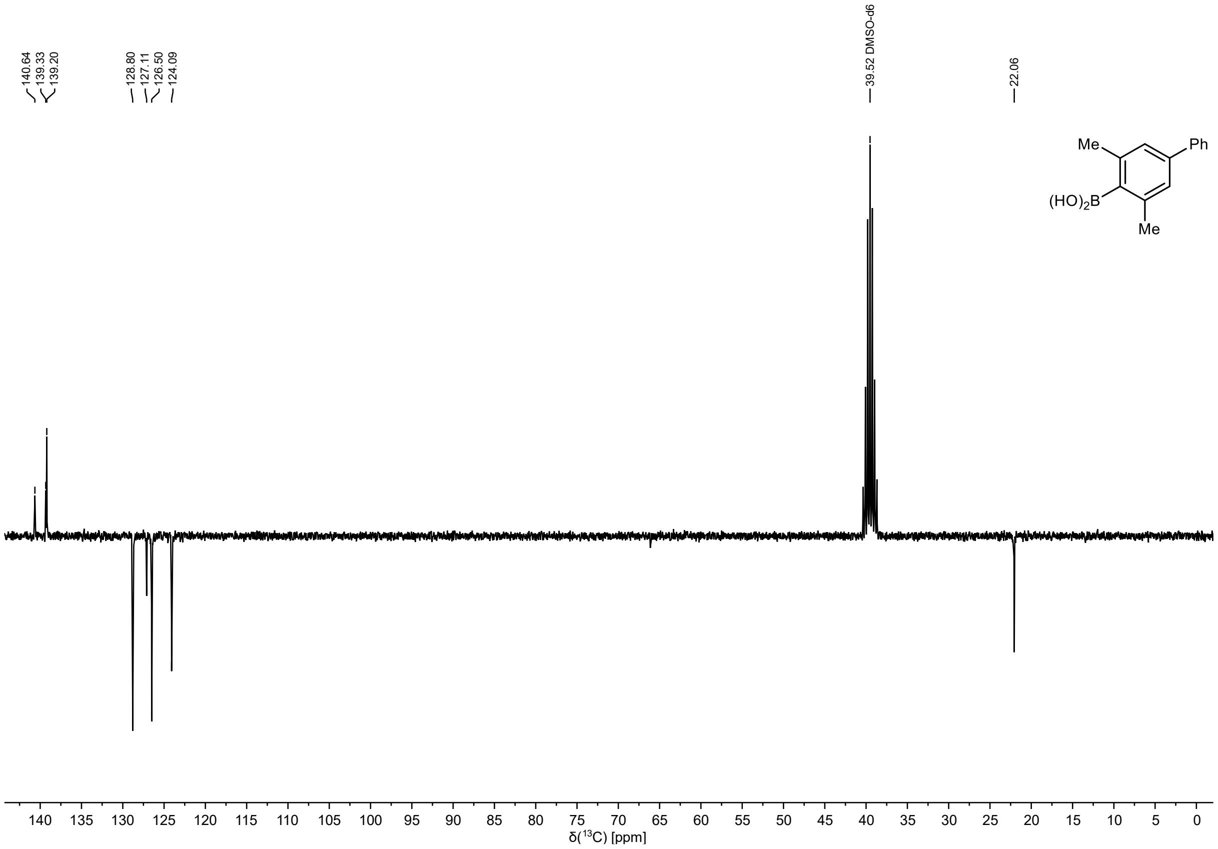

1H NMR (400 MHz, CDCl3) **SI-26b**

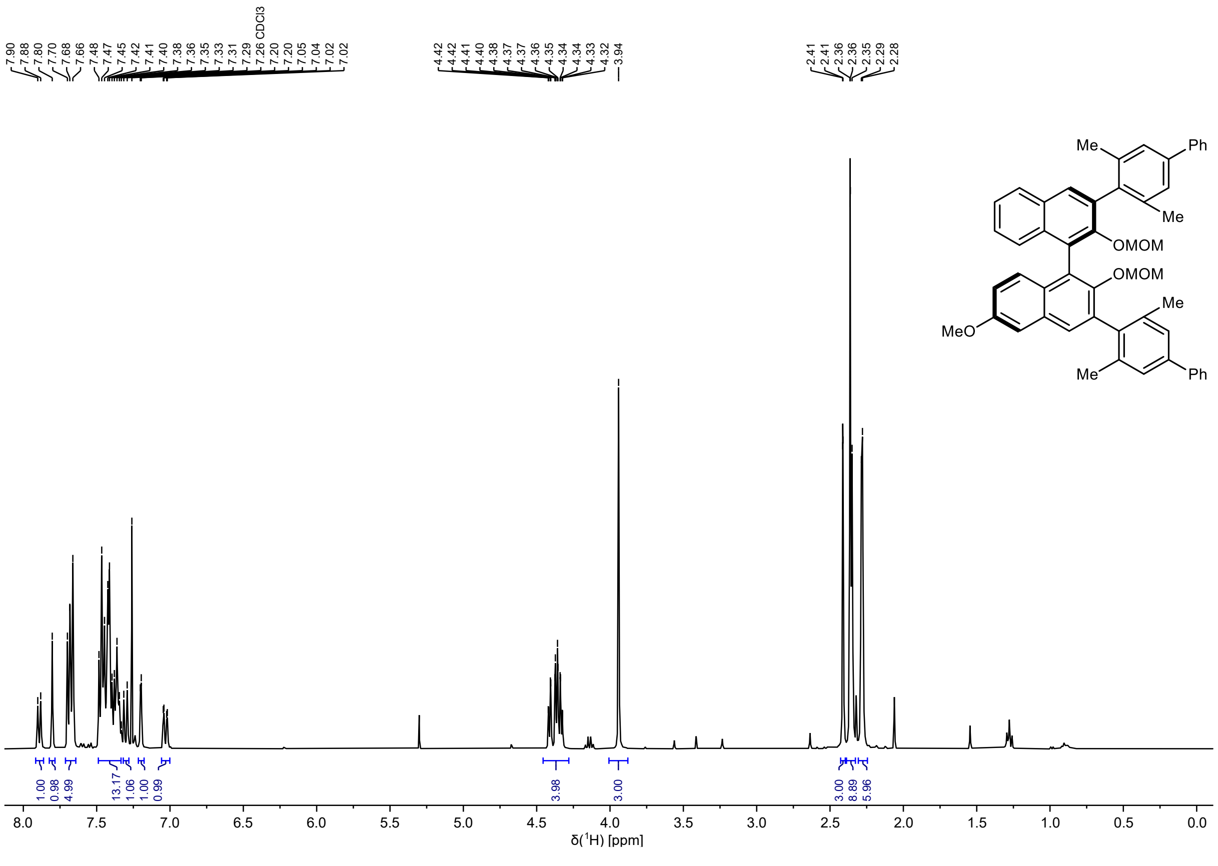

13C (APT) NMR (100 MHz, $CDCl_3$) **SI-26b**

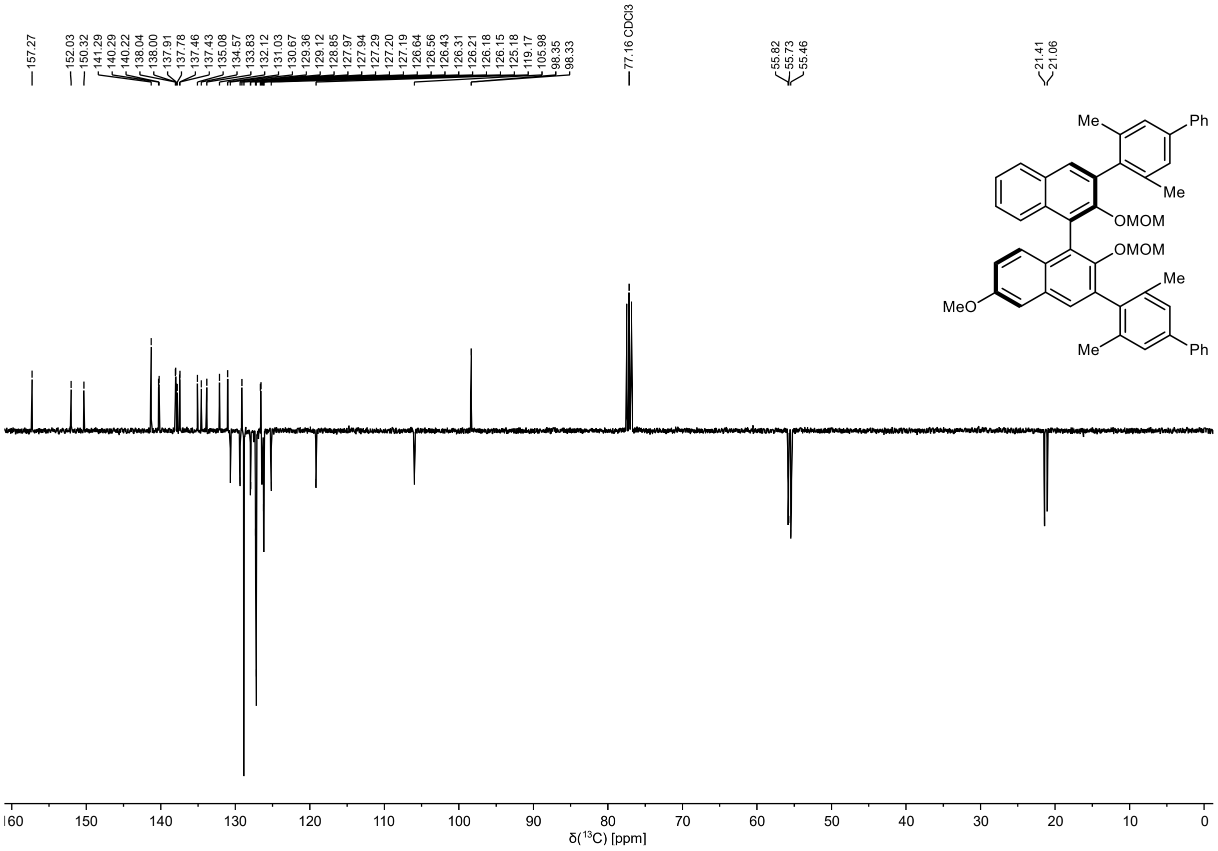

1H NMR (400 MHz, $CDCl_3$) **SI-27b**

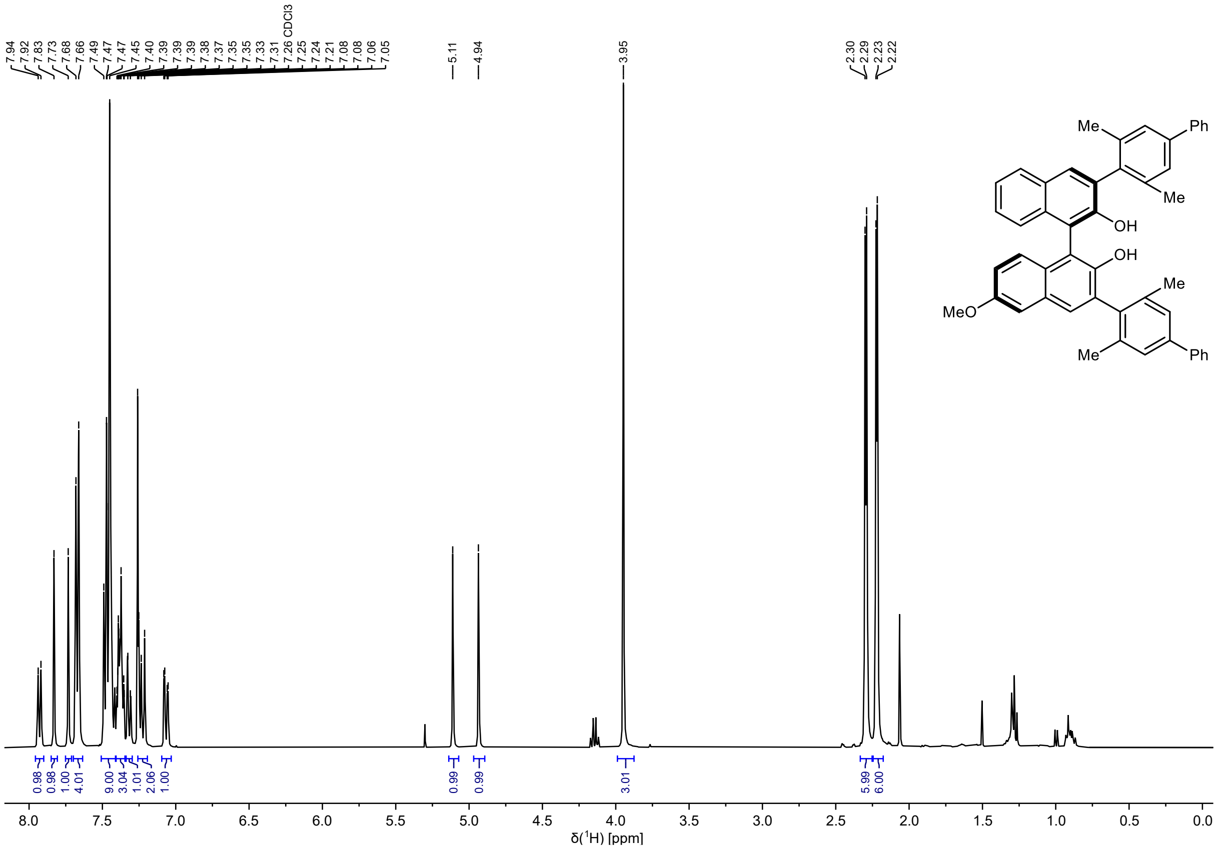

13C (APT) NMR (100 MHz, $CDCl_3$) **SI-27b**

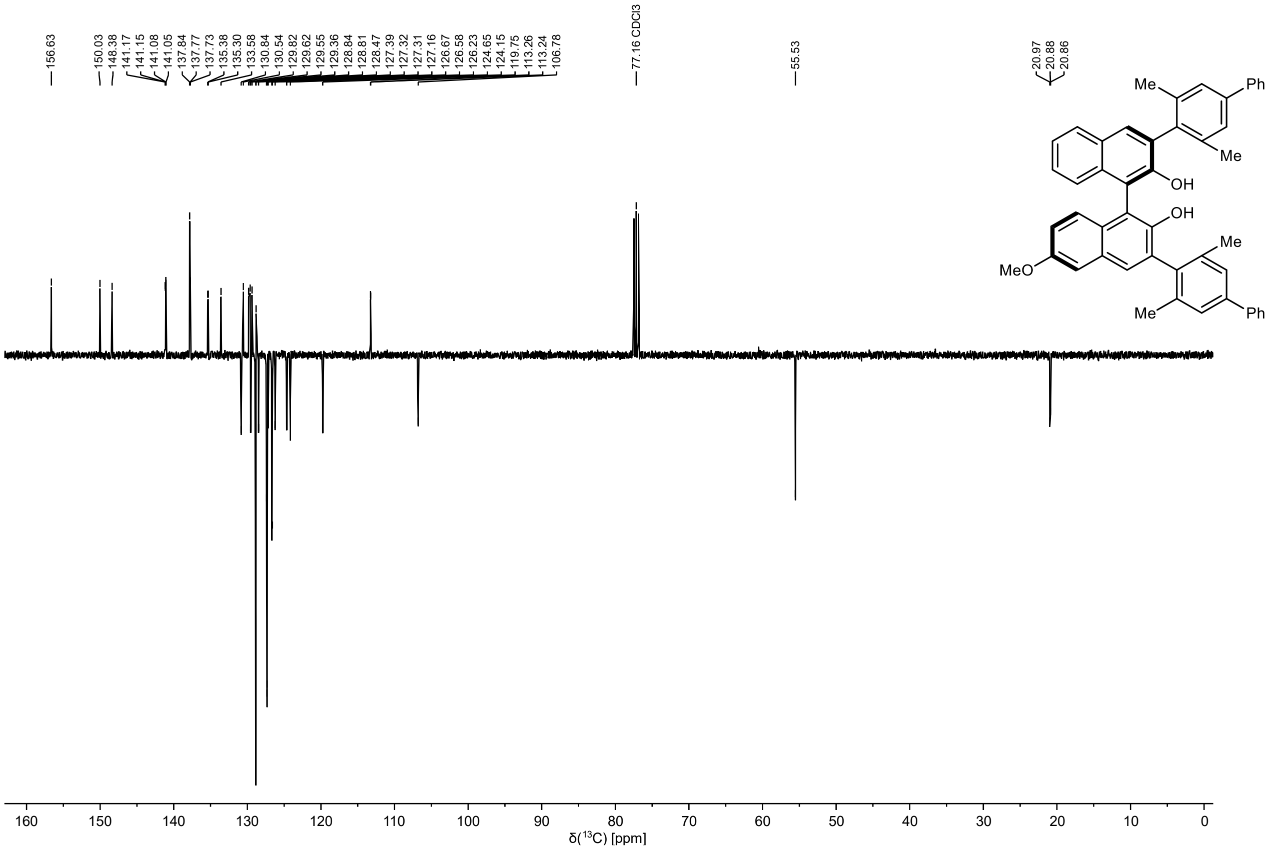

1H NMR (400 MHz, $CDCl_3$) **SI-28b**

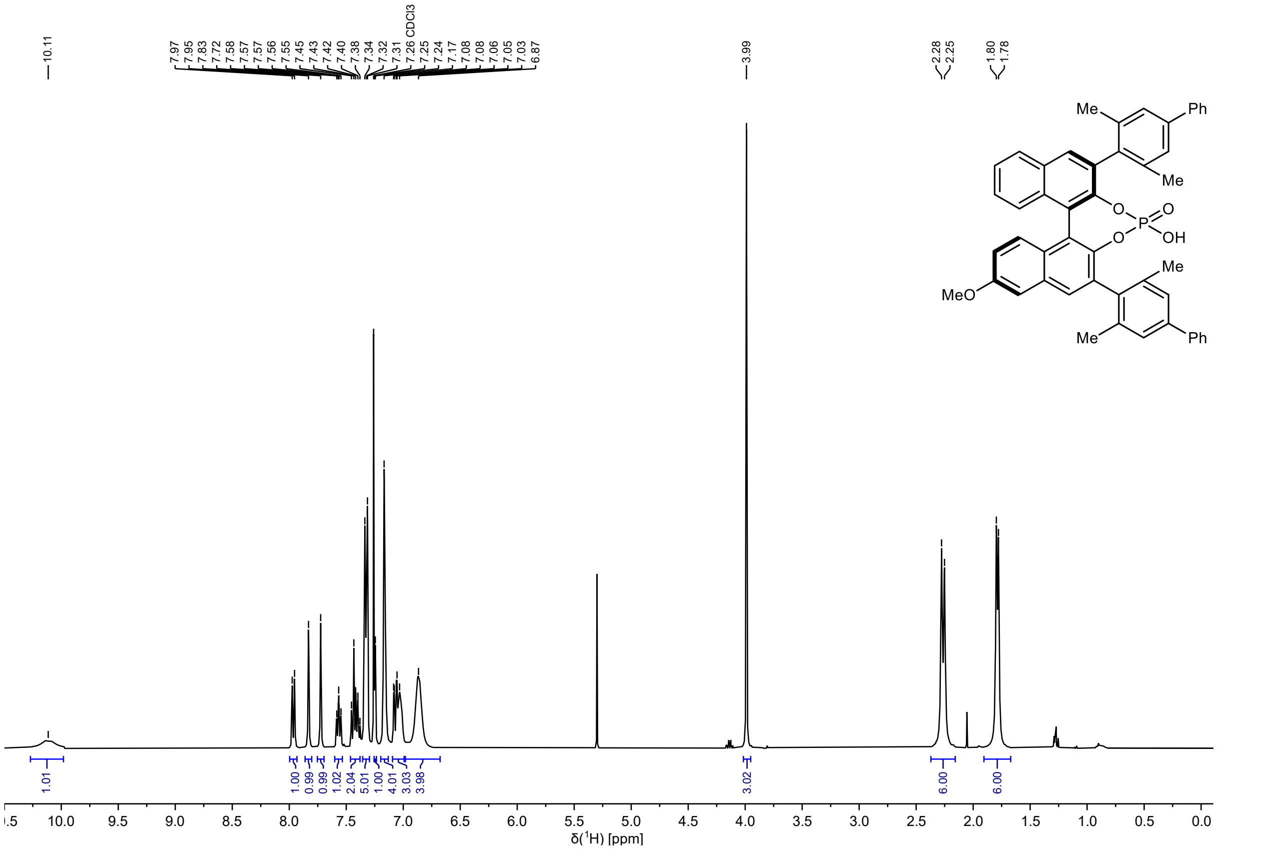

31P NMR (162 MHz, CDCl3) **SI-28b**

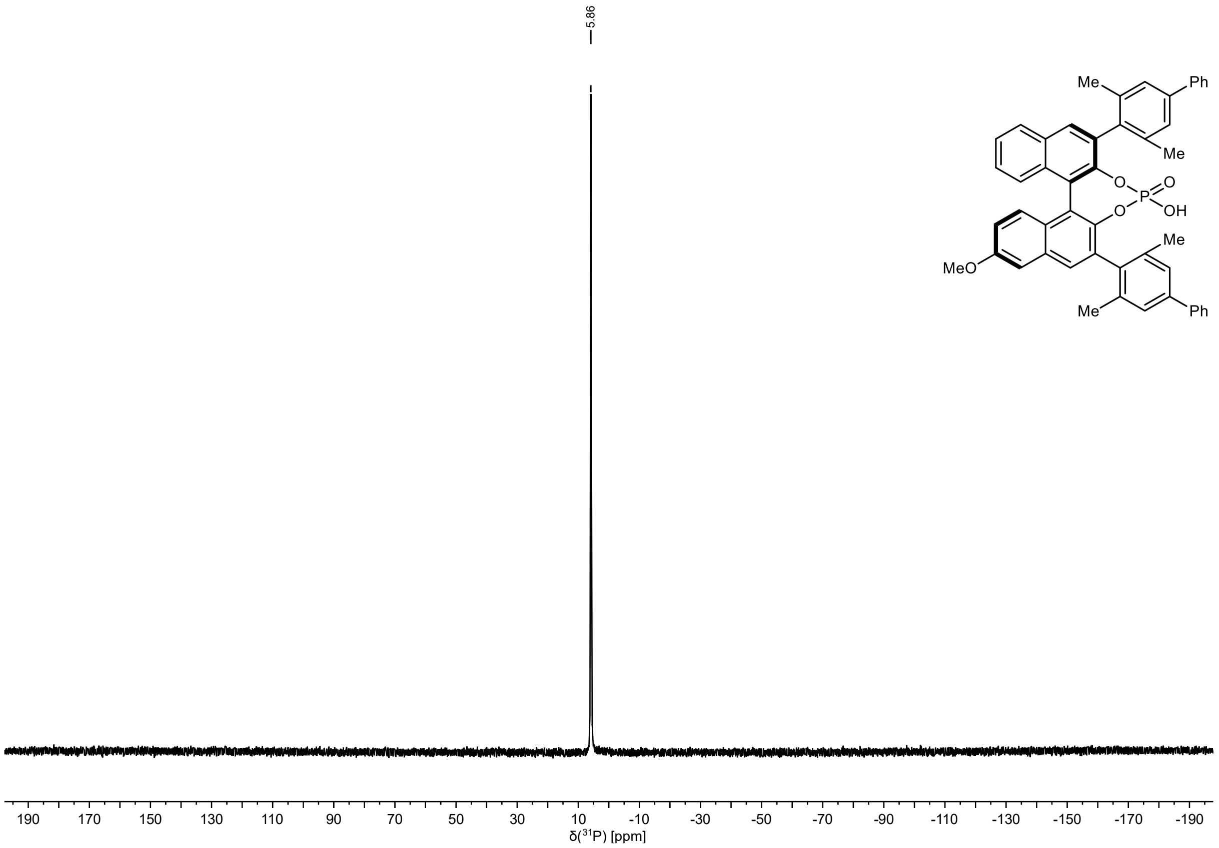

13C (APT) NMR (100 MHz, CDCl3) **SI-28b**

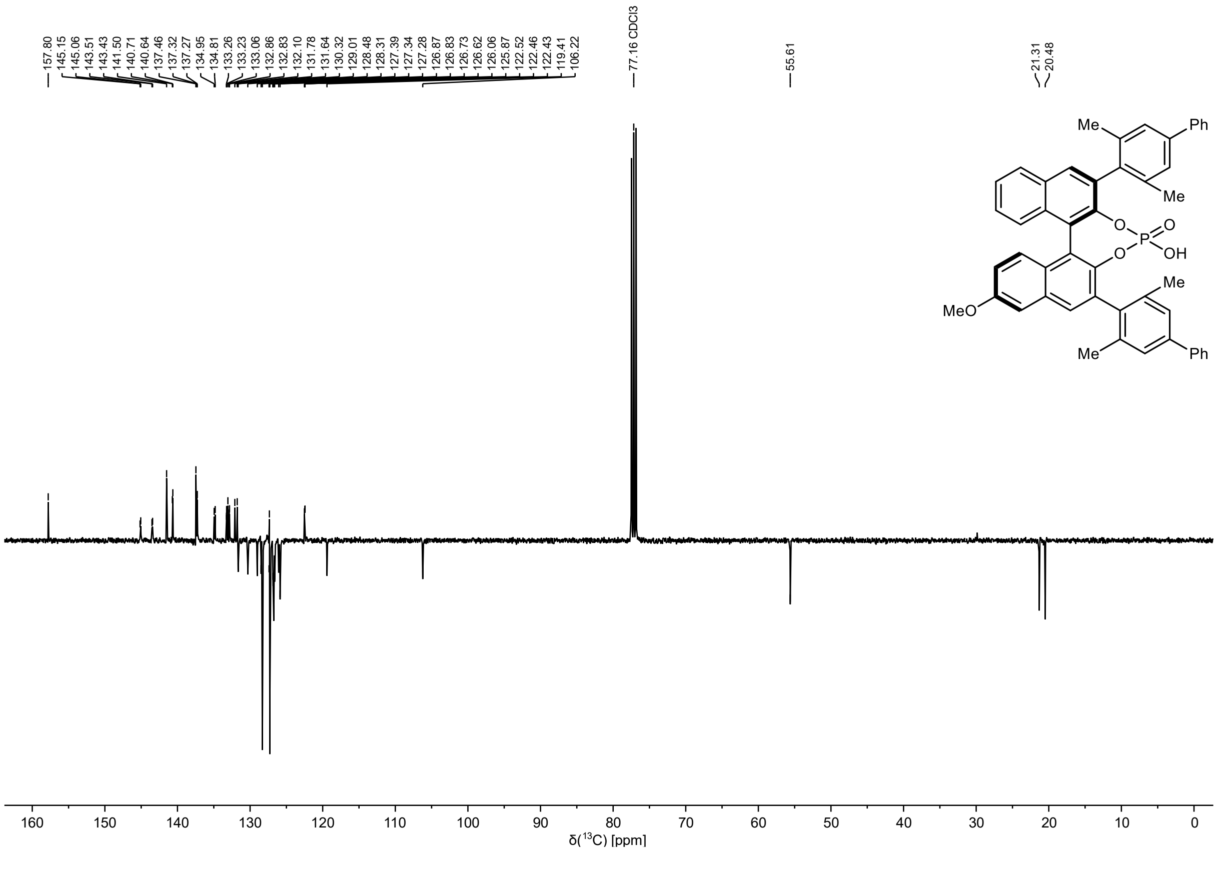

1H NMR (400 MHz, $CDCl_3$) **SI-29b**

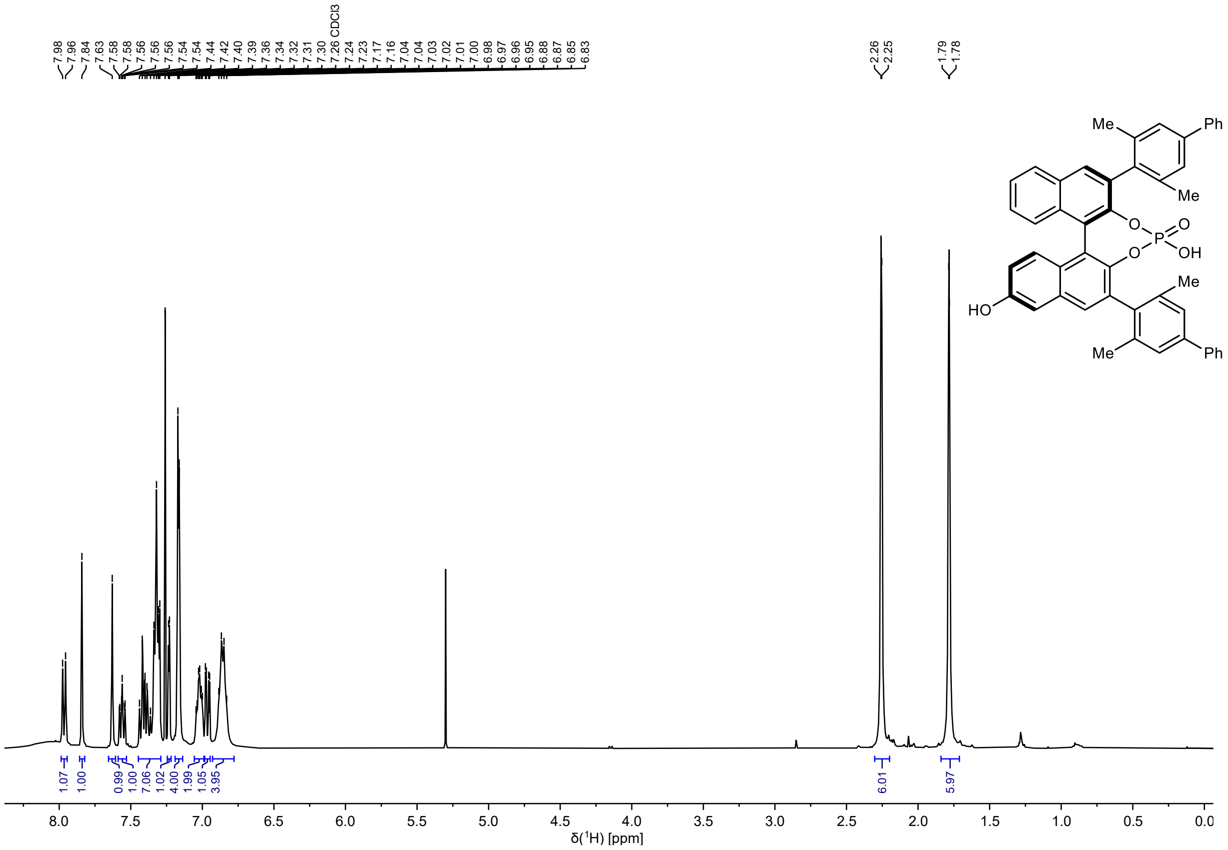

31P NMR (162 MHz, $CDCl_3$) **SI-29b**

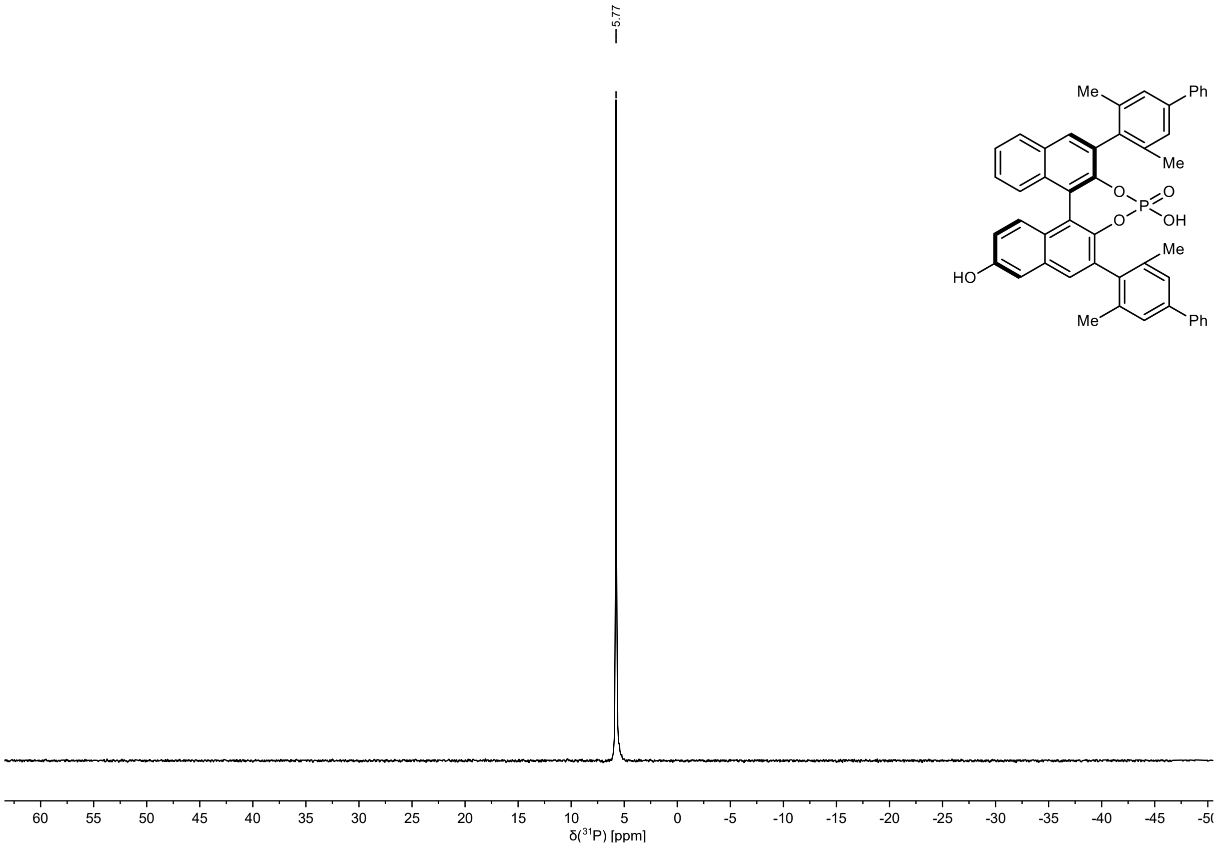

13C (APT) NMR (100 MHz, $CDCl_3$) **SI-29b**

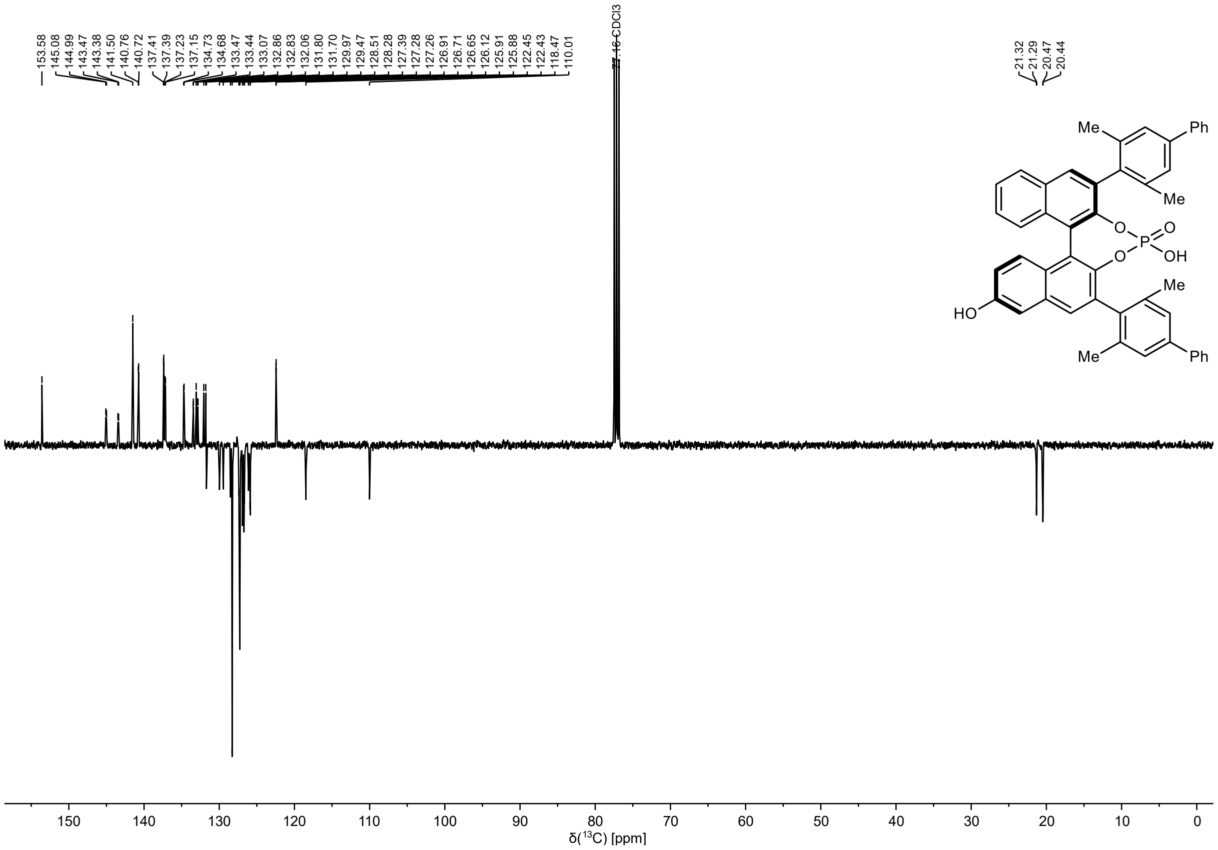

1H NMR (400 MHz, DMSO-d_6) **SI-30b**

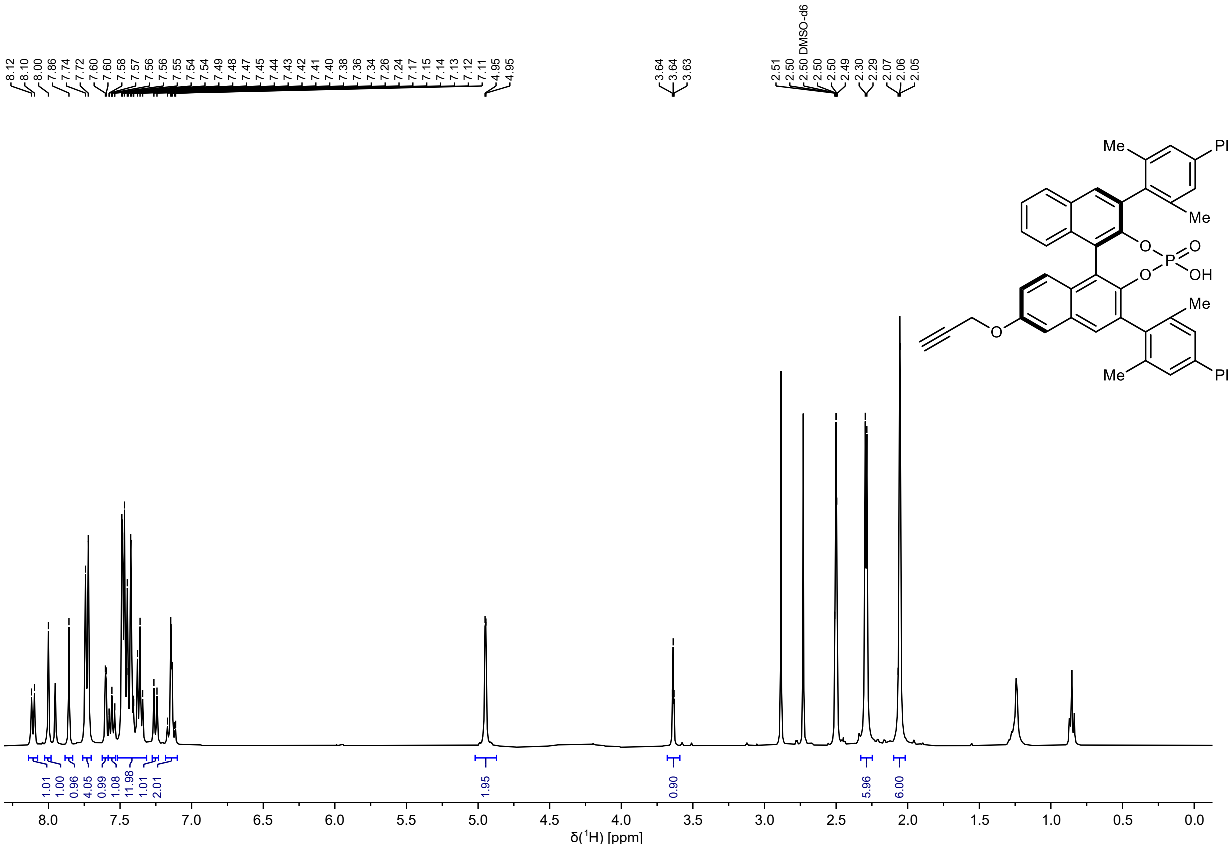

31P NMR (162 MHz, DMSO-d6) **SI-30b**

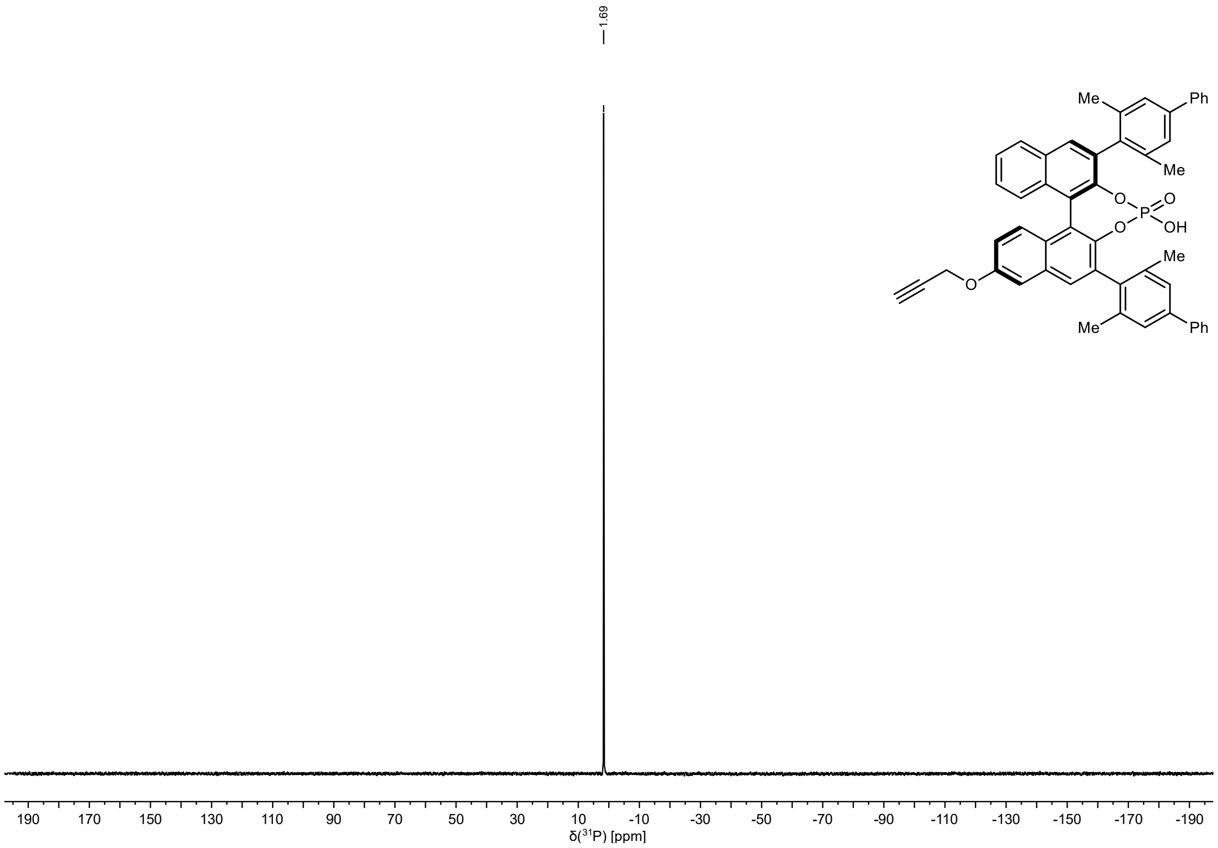

13C (APT) NMR (100 MHz, DMSO-d6) **SI-30b**

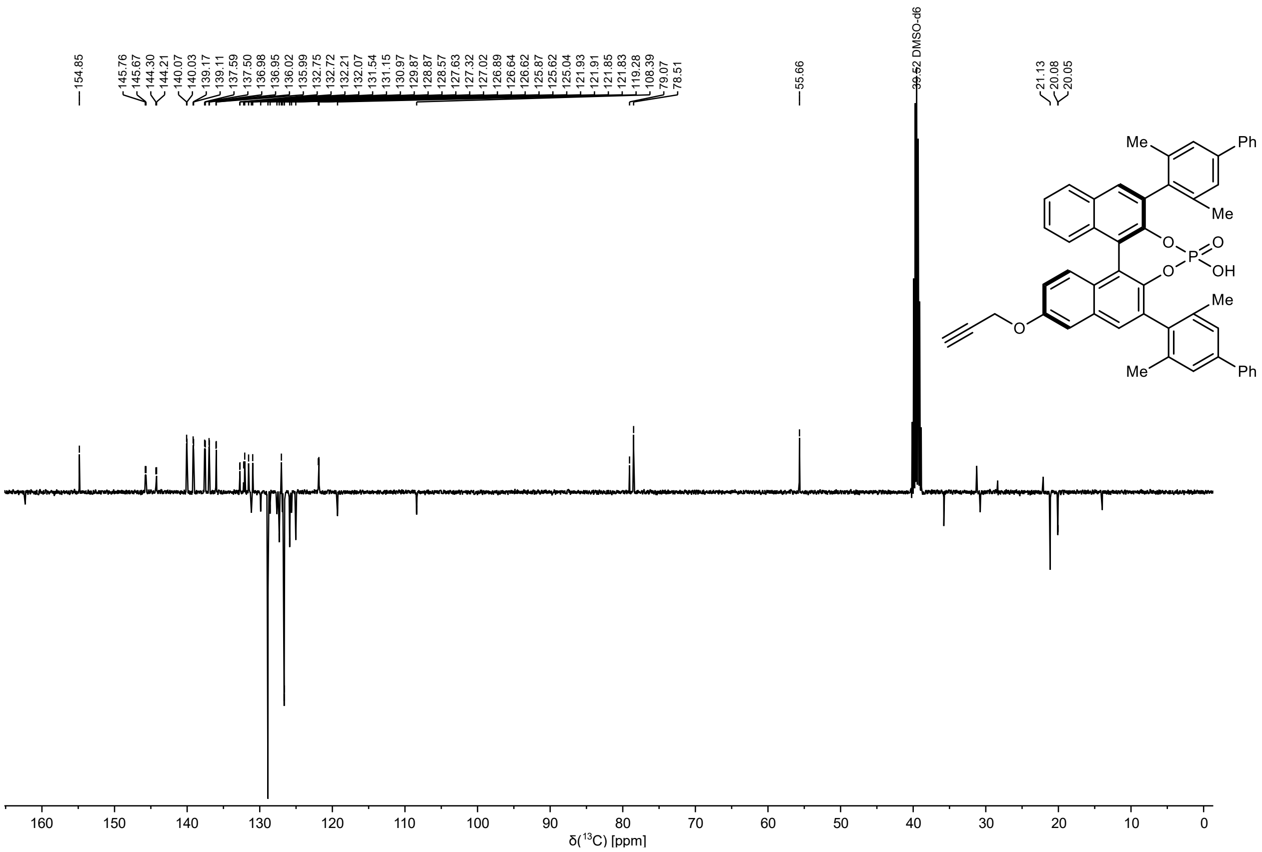

3. NMR Spectra of Products

1H NMR (400 MHz, CDCl3) **SI-5a**

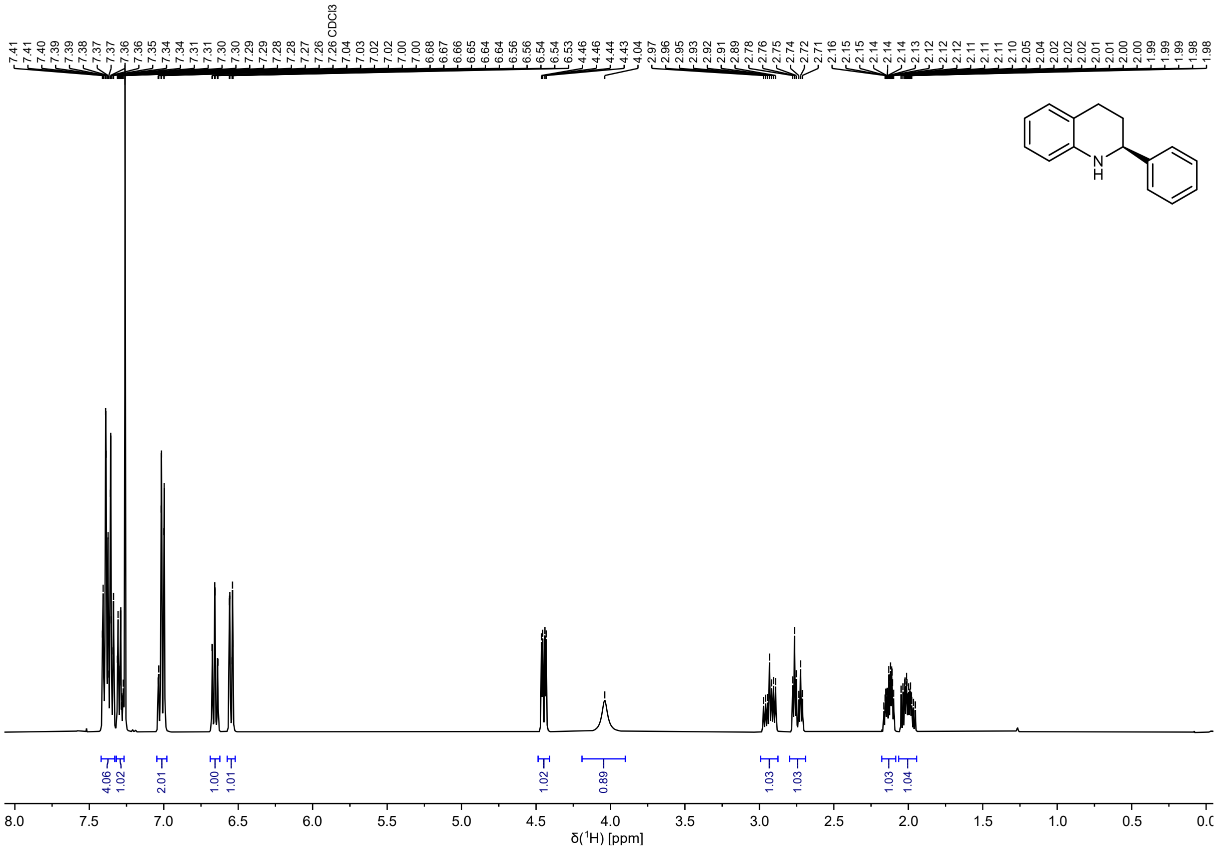

13C (APT) NMR (100 MHz, CDCl3) **SI-5a**

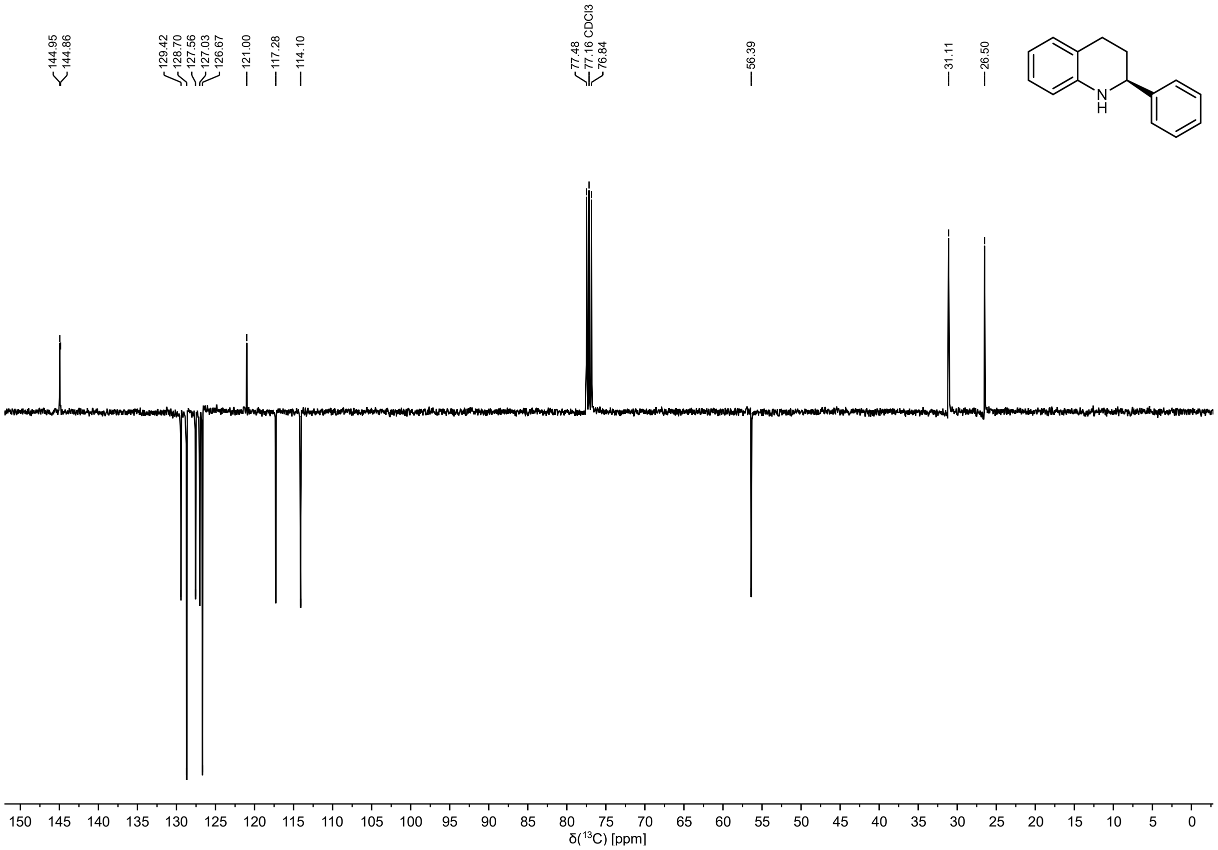

1H NMR (300 MHz, $CDCl_3$) **SI-5b**

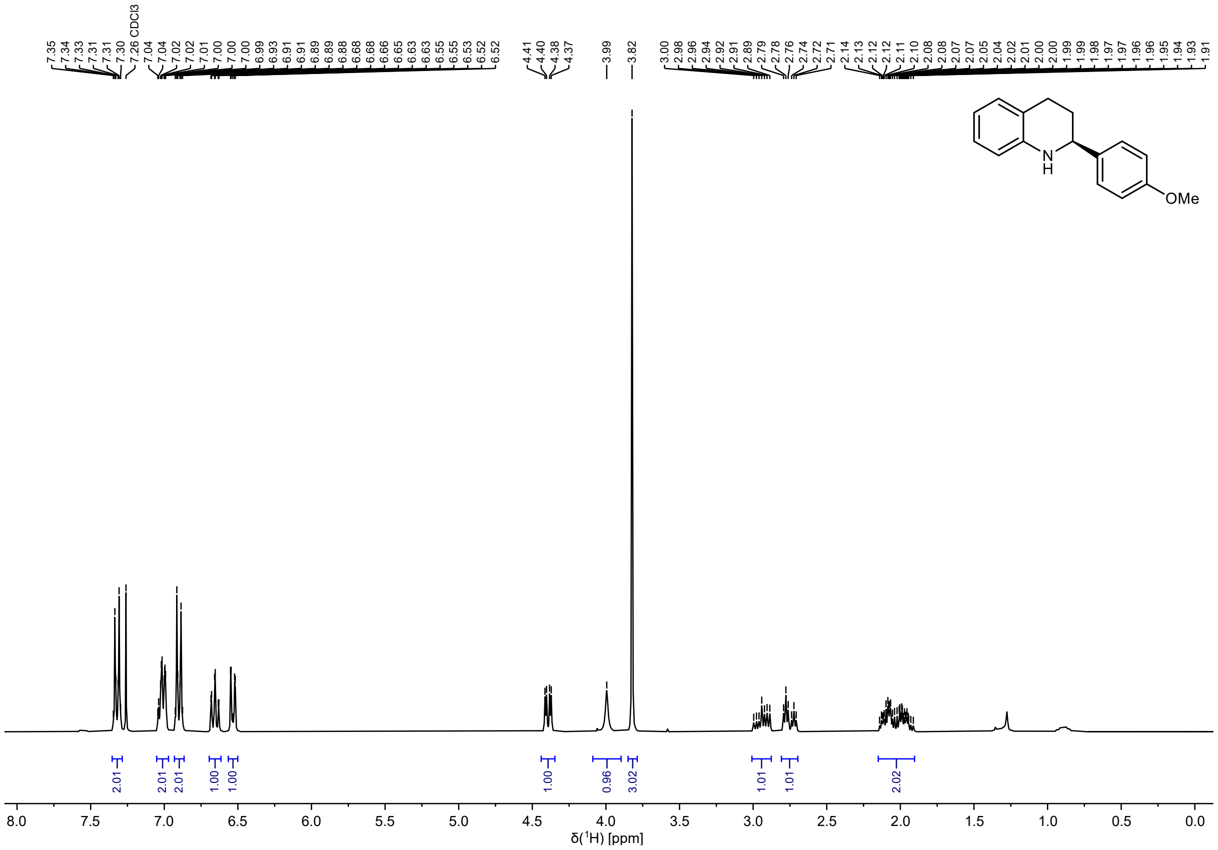

13C (APT) NMR (75 MHz, $CDCl_3$) **SI-5b**

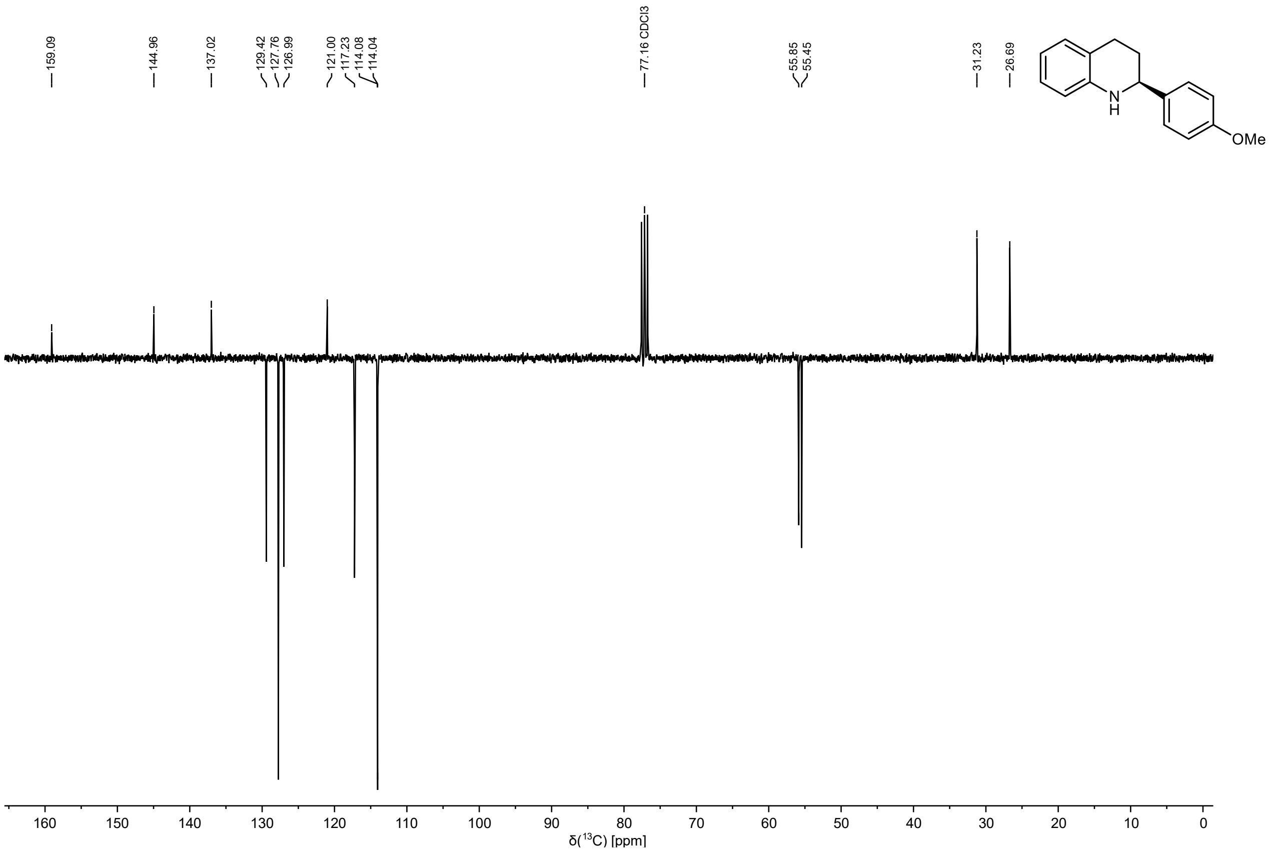

1H NMR (400 MHz, $CDCl_3$) **SI-5c**

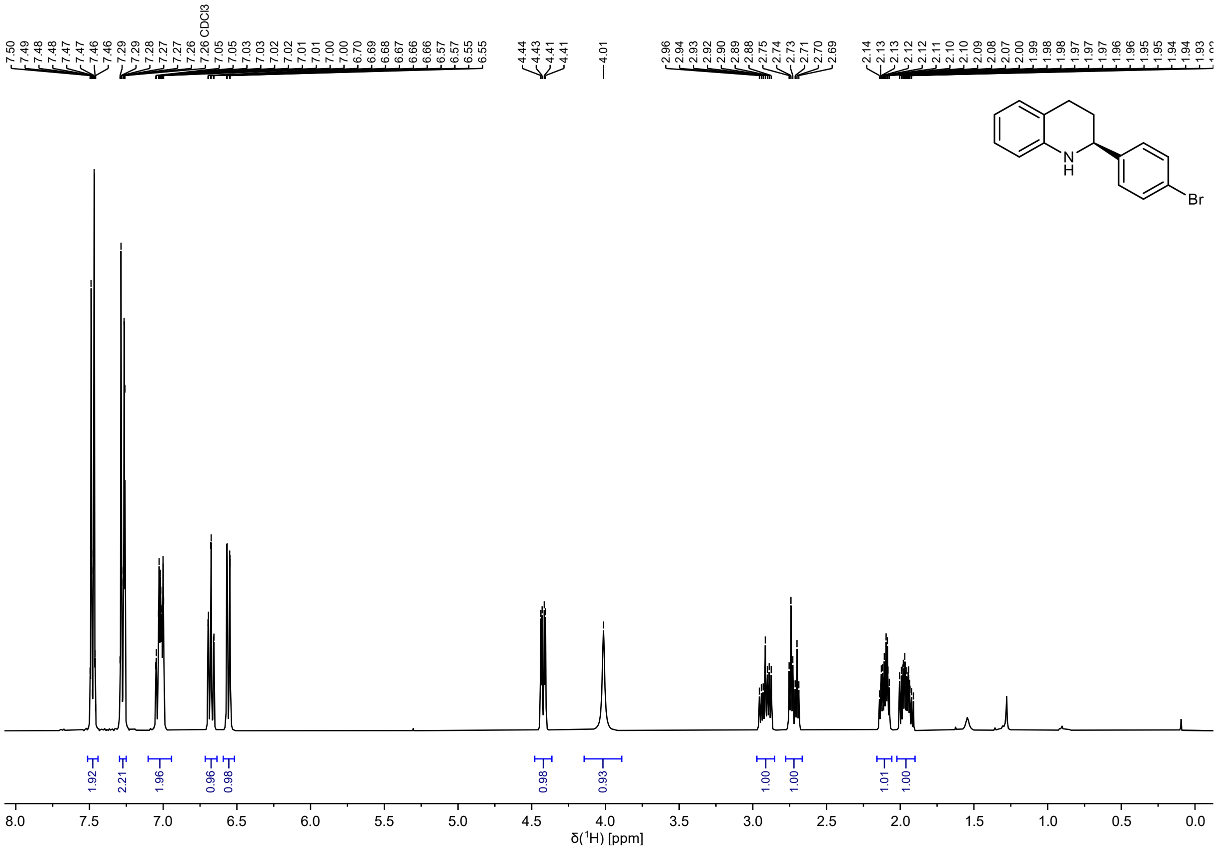

13C (APT) NMR (100 MHz, $CDCl_3$) **SI-5c**

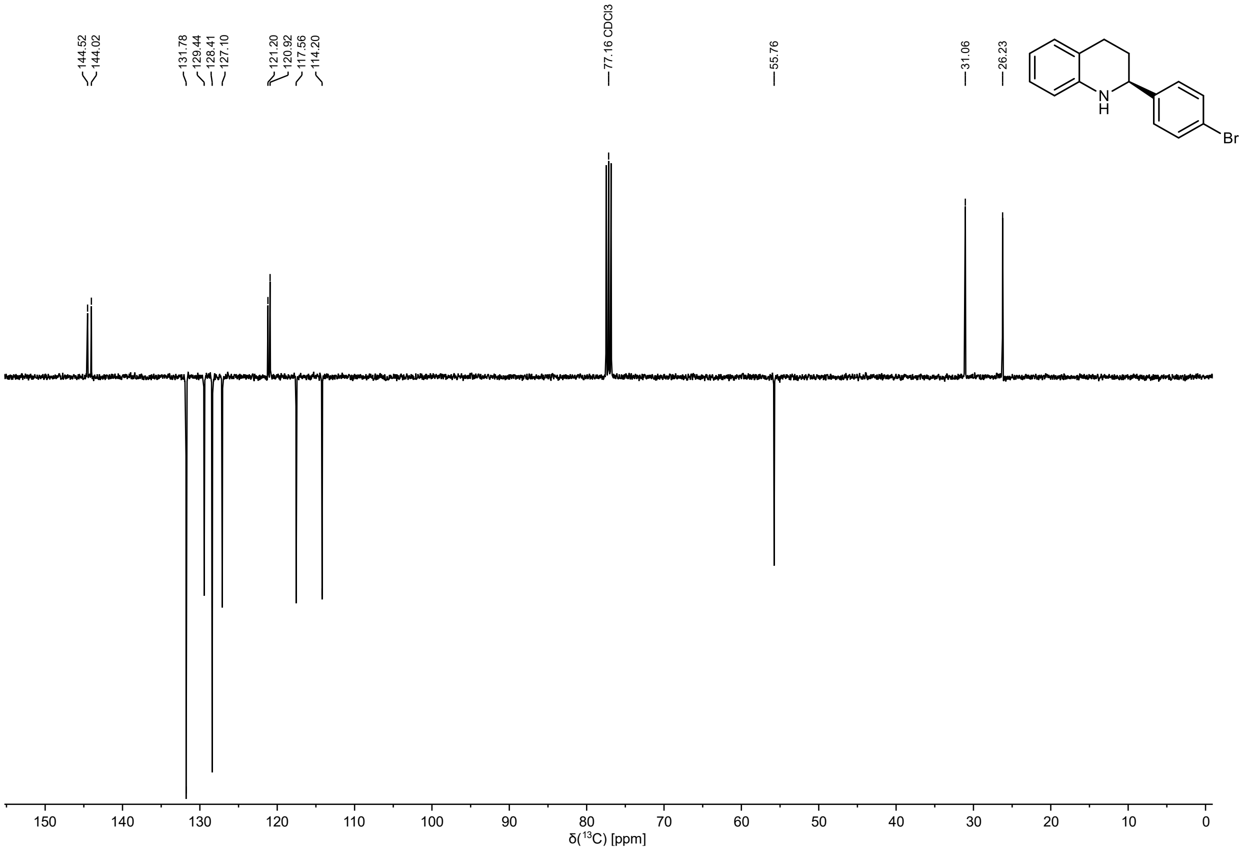

1H NMR (400 MHz, CDCl3) **SI-5d**

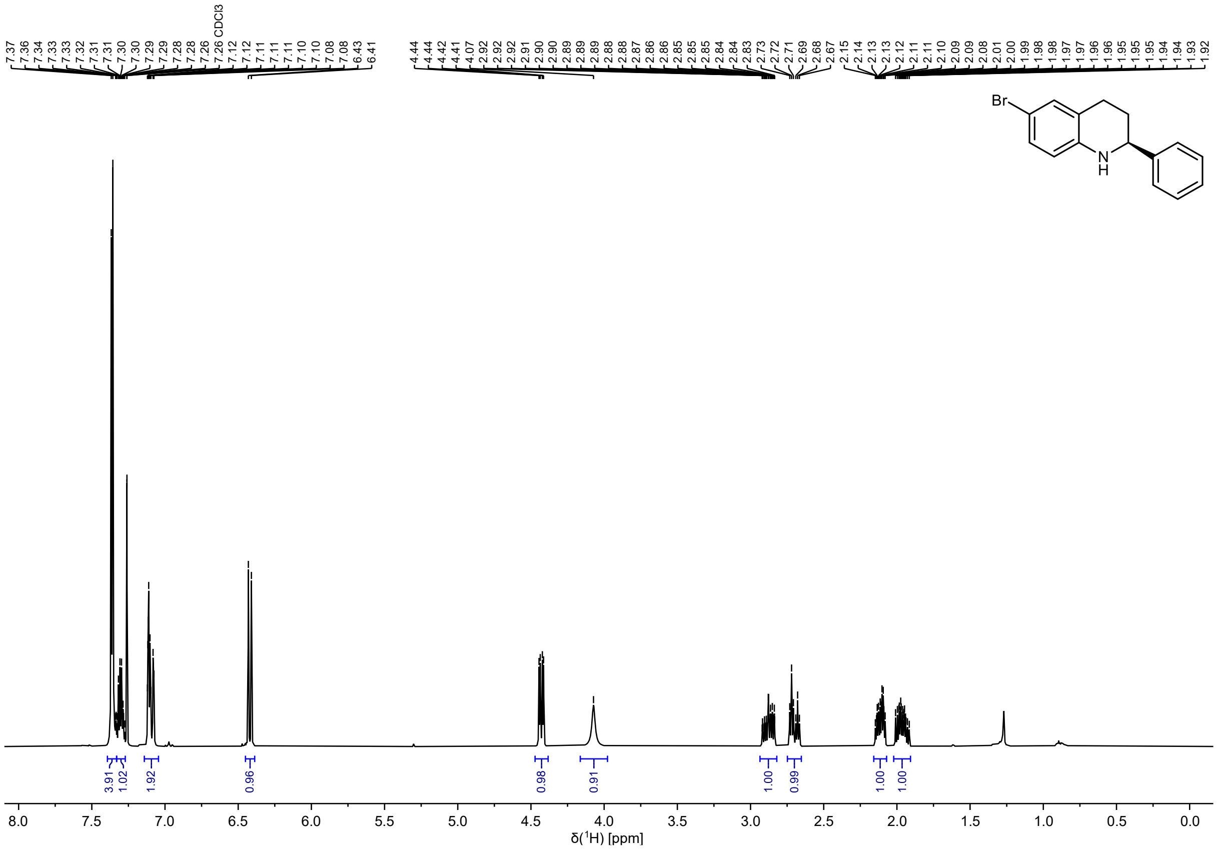

13C (APT) NMR (100 MHz, CDCl3) **SI-5d**

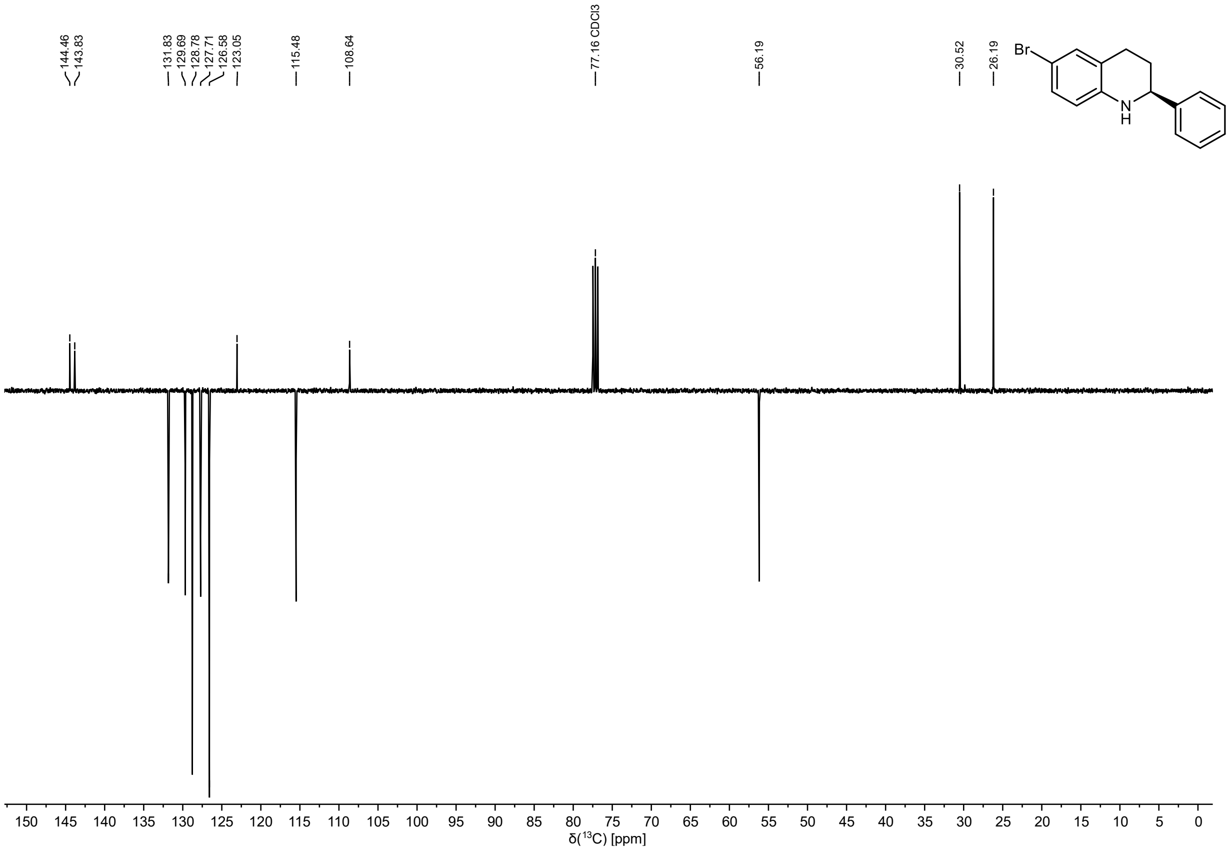

1H NMR (400 MHz, $CDCl_3$) **SI-32**

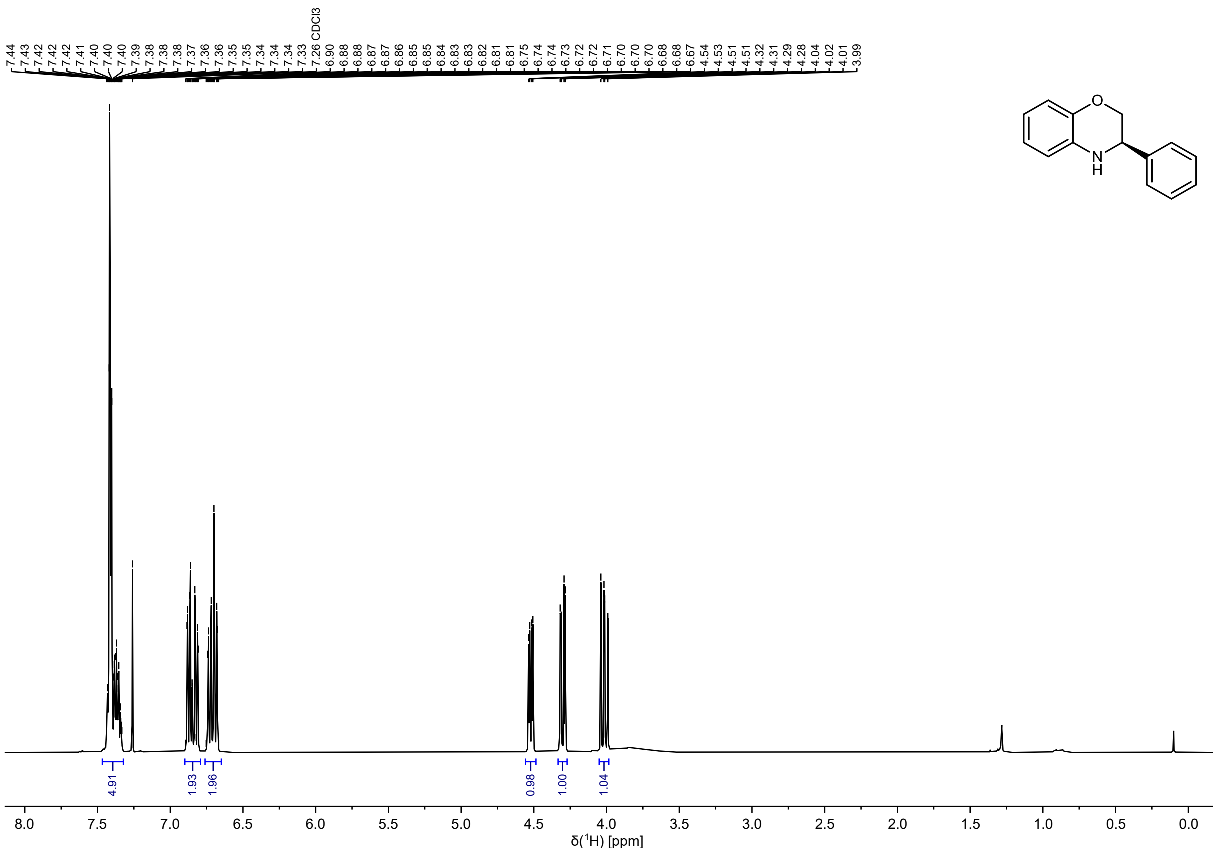

13C (APT) NMR (100 MHz, $CDCl_3$) **SI-32**

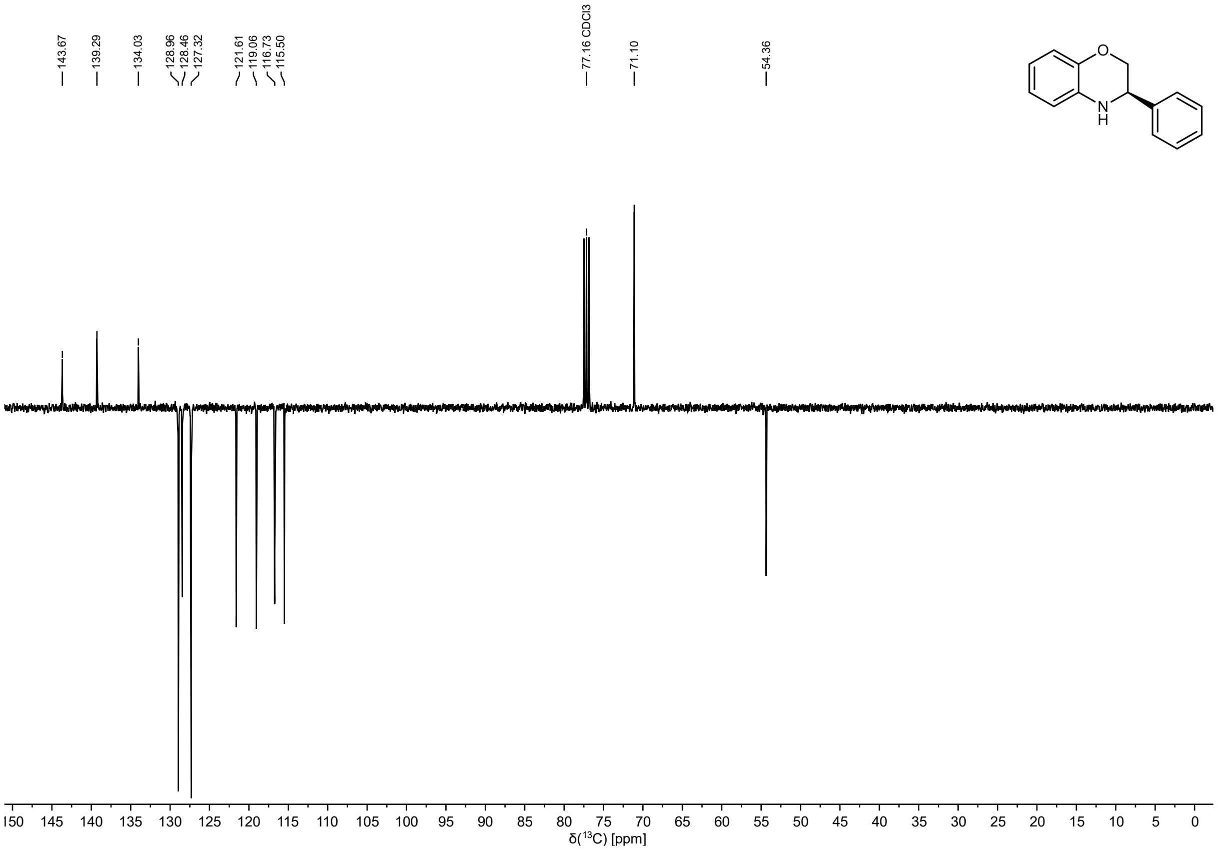

1H NMR (400 MHz, $CDCl_3$), major Diastereomer **SI-11a**

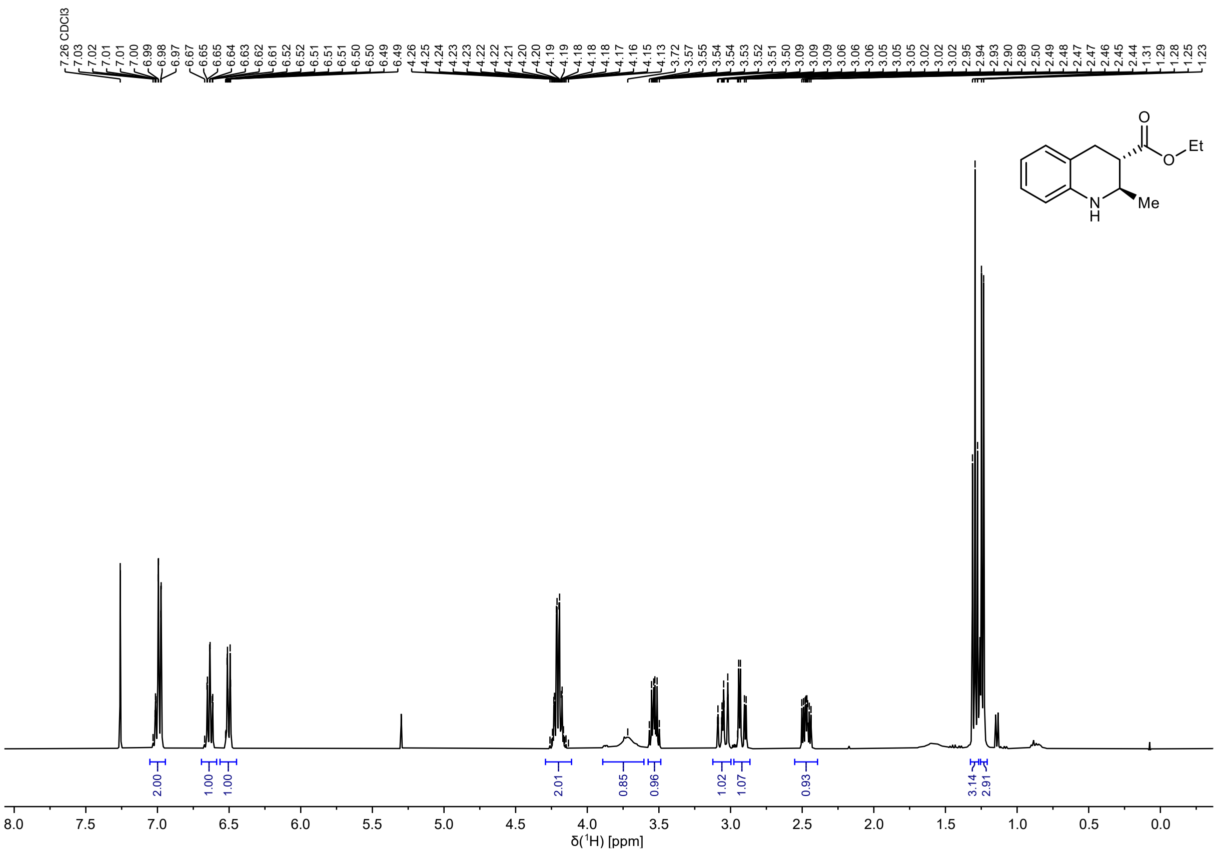

1H NMR (400 MHz, $CDCl_3$), minor Diastereomer **SI-11a**

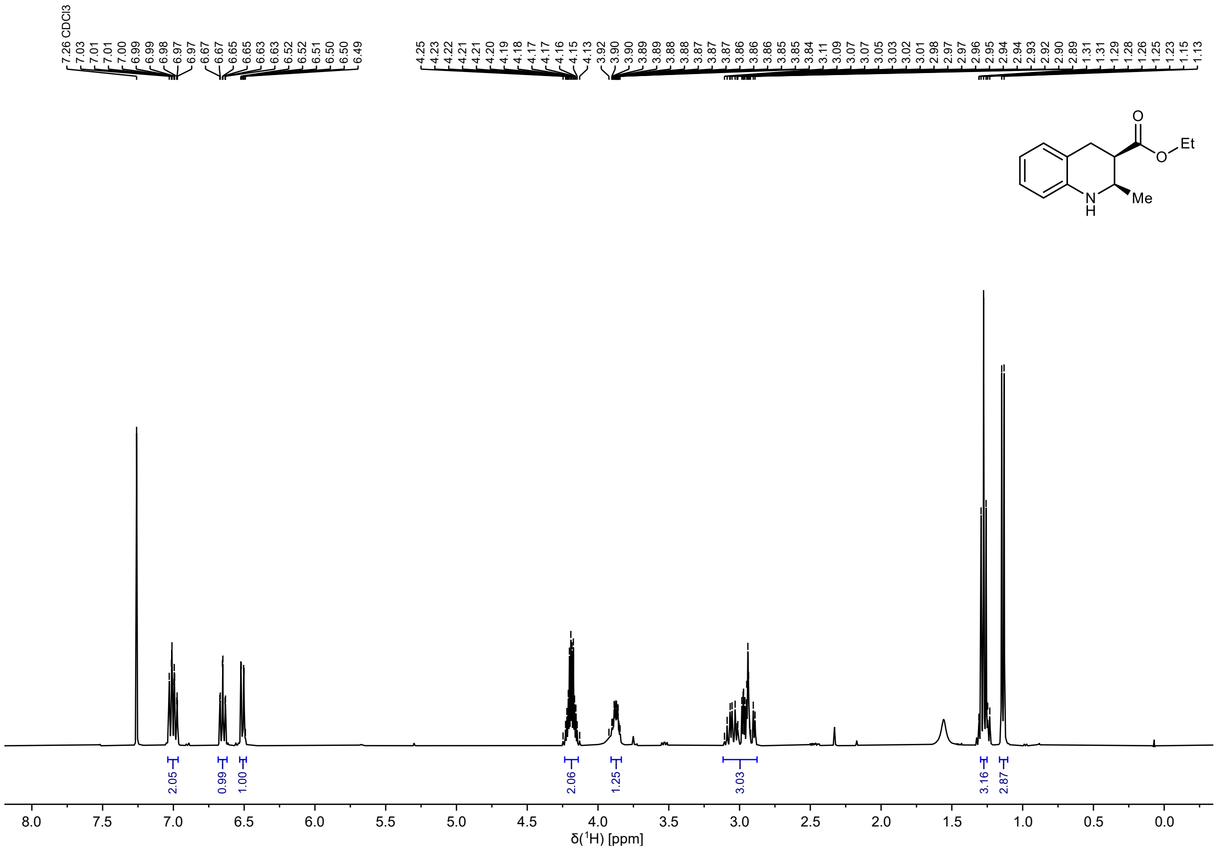

13C (APT) NMR (75 MHz, CDCl3) **SI-11a**

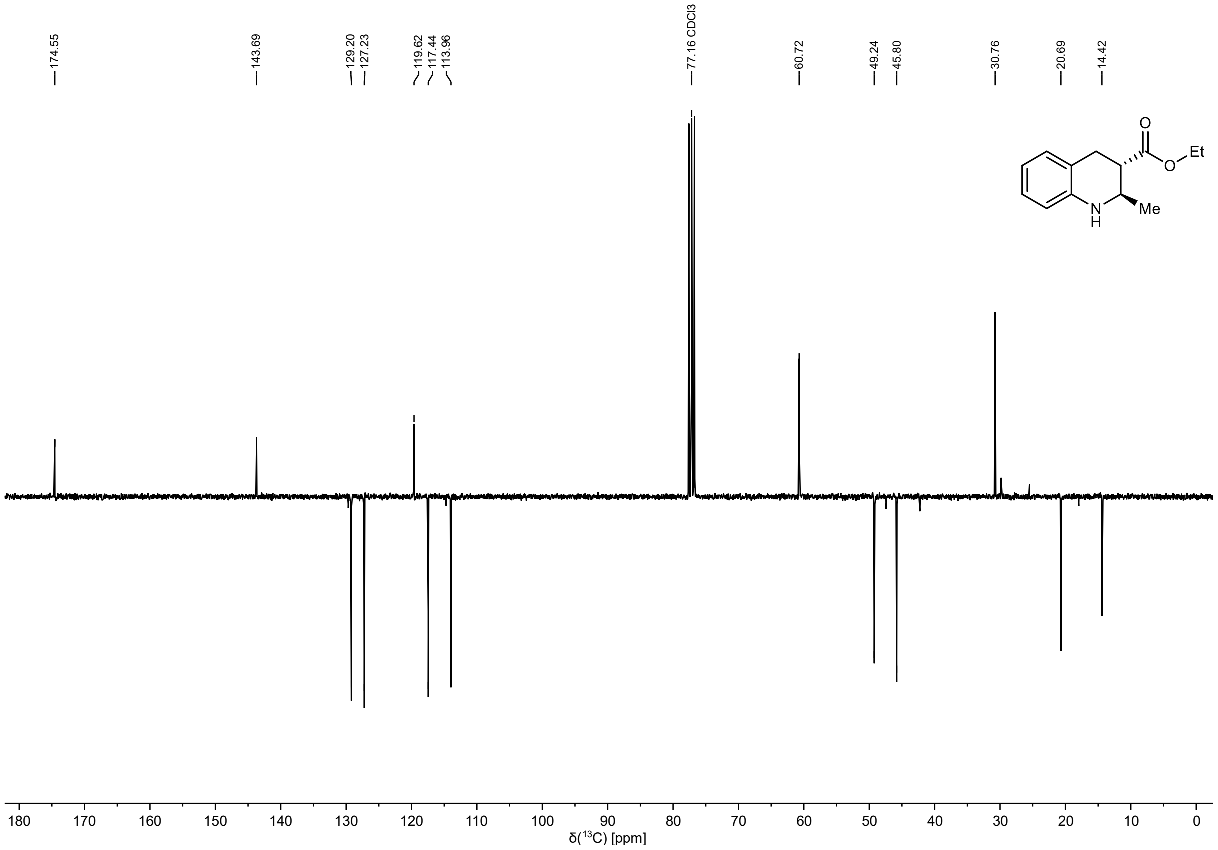

1H NMR (400 MHz, CDCl3) major Diastereomer **SI-11b**

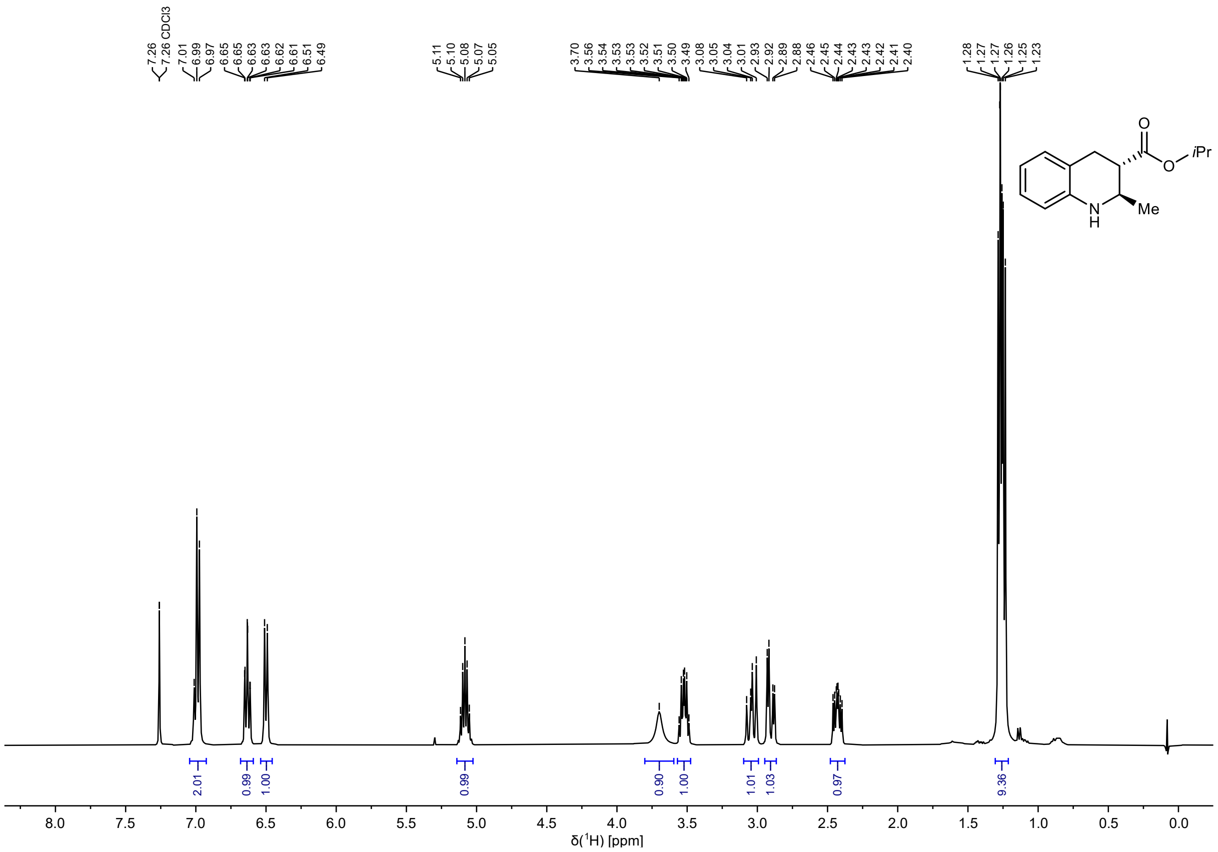

1H NMR (400 MHz, $CDCl_3$) minor (+ major Diastereomer) **SI-11b**

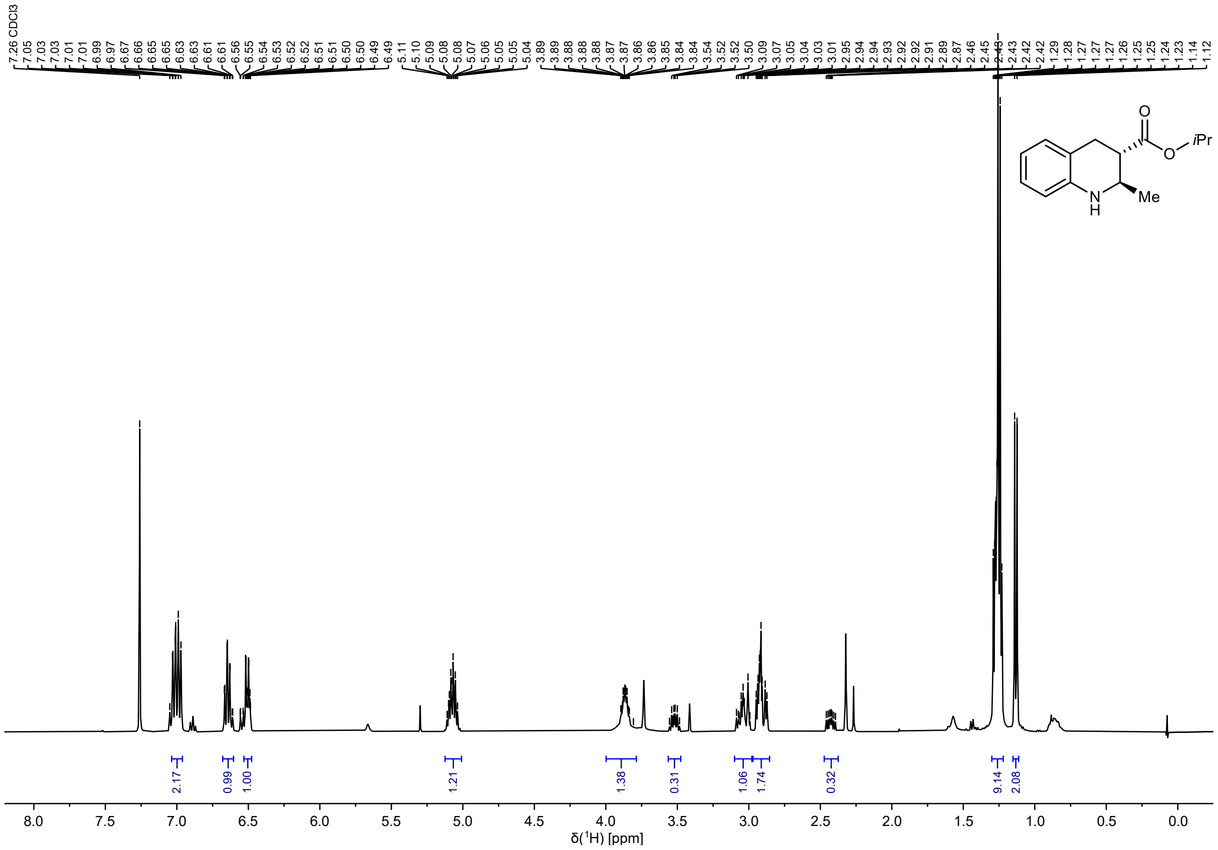

13C (APT) NMR (75 MHz, $CDCl_3$) **SI-11b**

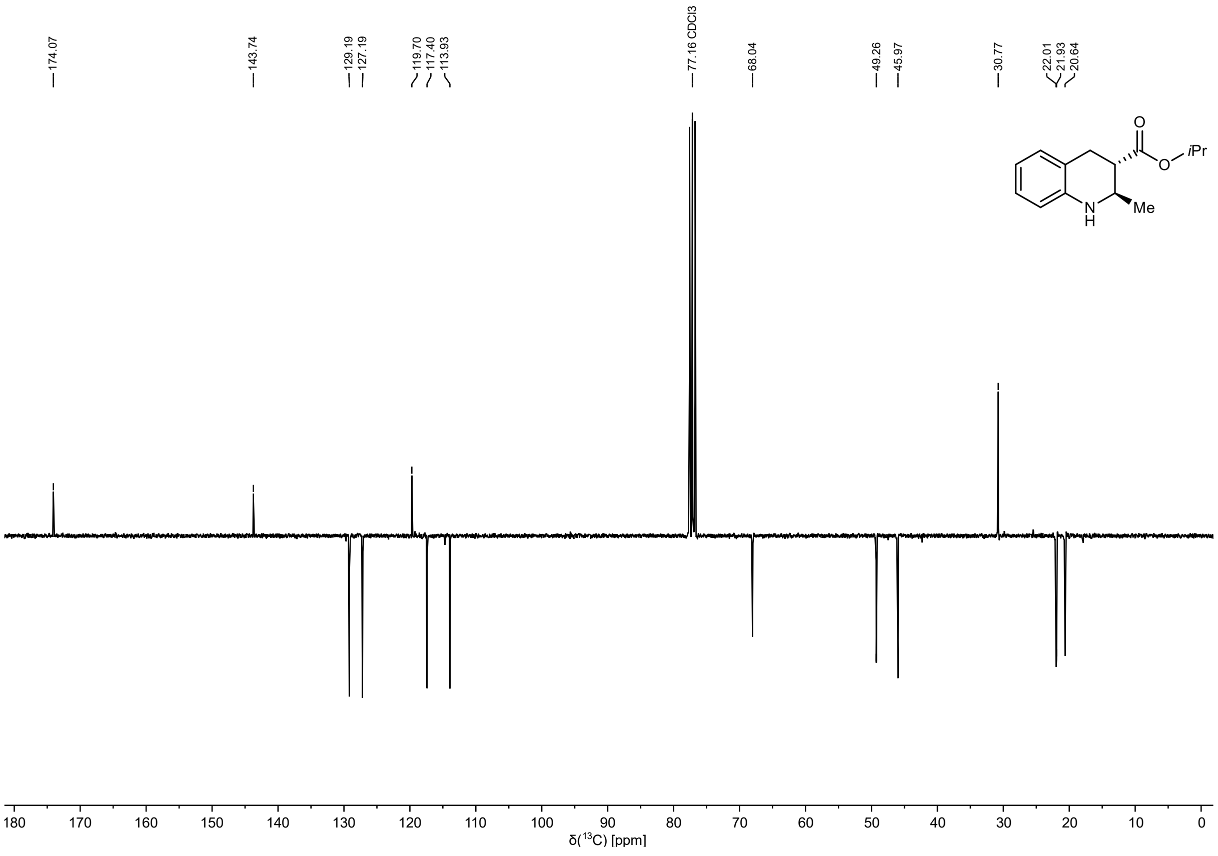

1H NMR (400 MHz, $CDCl_3$) major Diastereomer **SI-11c**

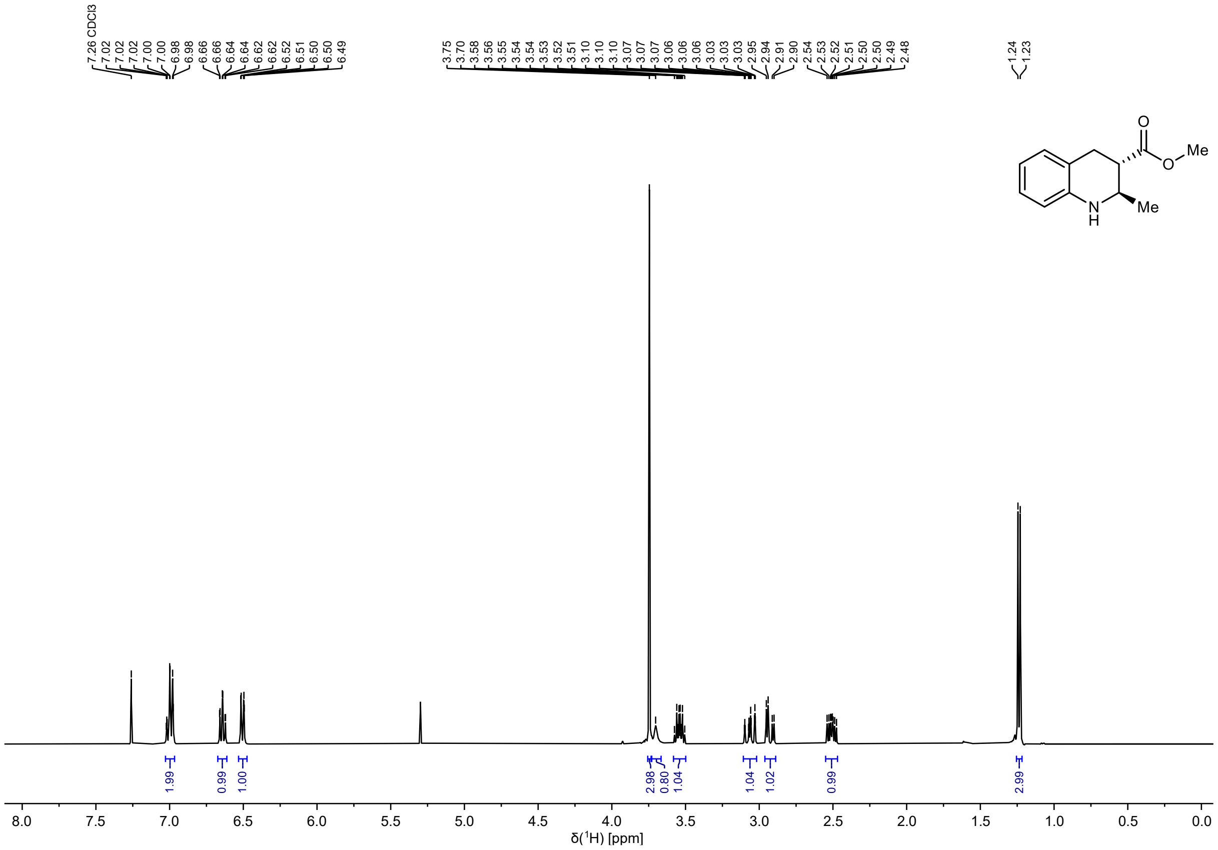

1H NMR (400 MHz, $CDCl_3$) minor Diastereomer **SI-11c**

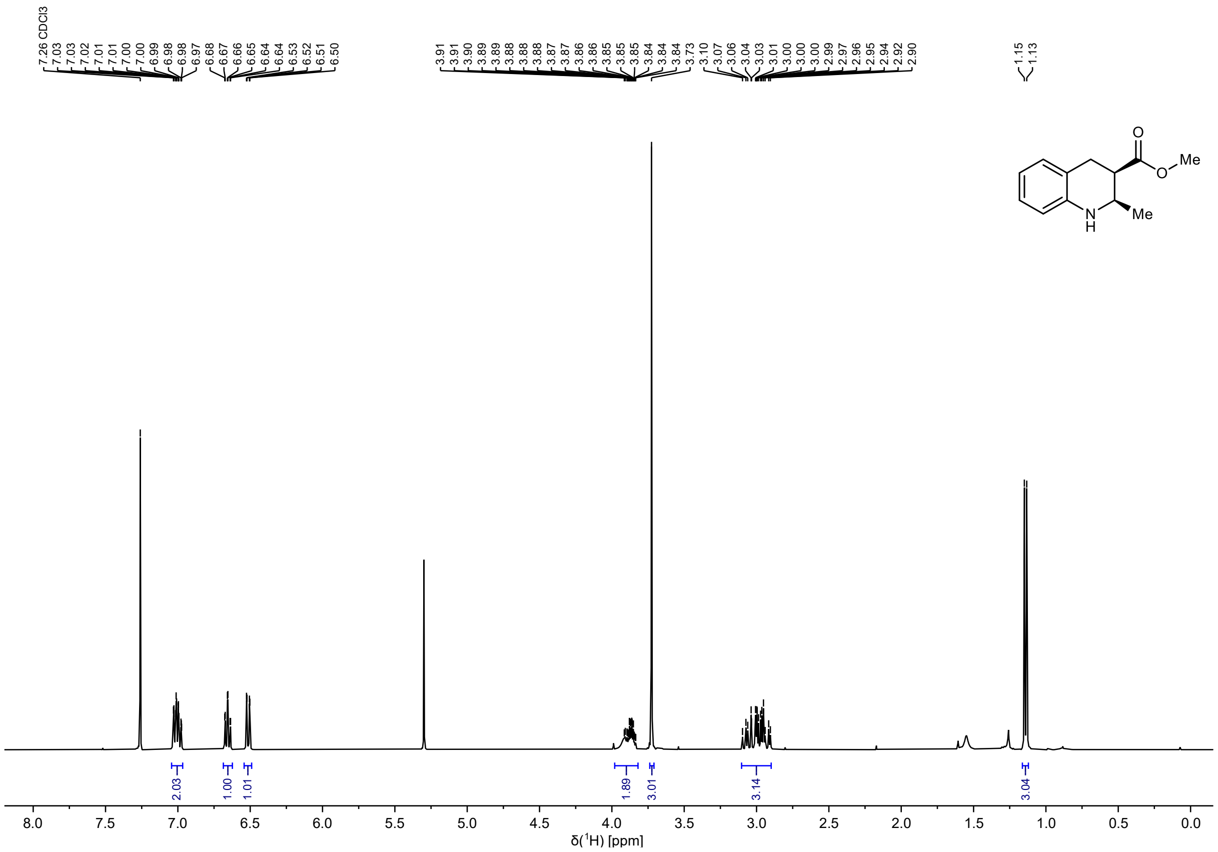

13C (APT) NMR (100 MHz, $CDCl_3$) **SI-11c**

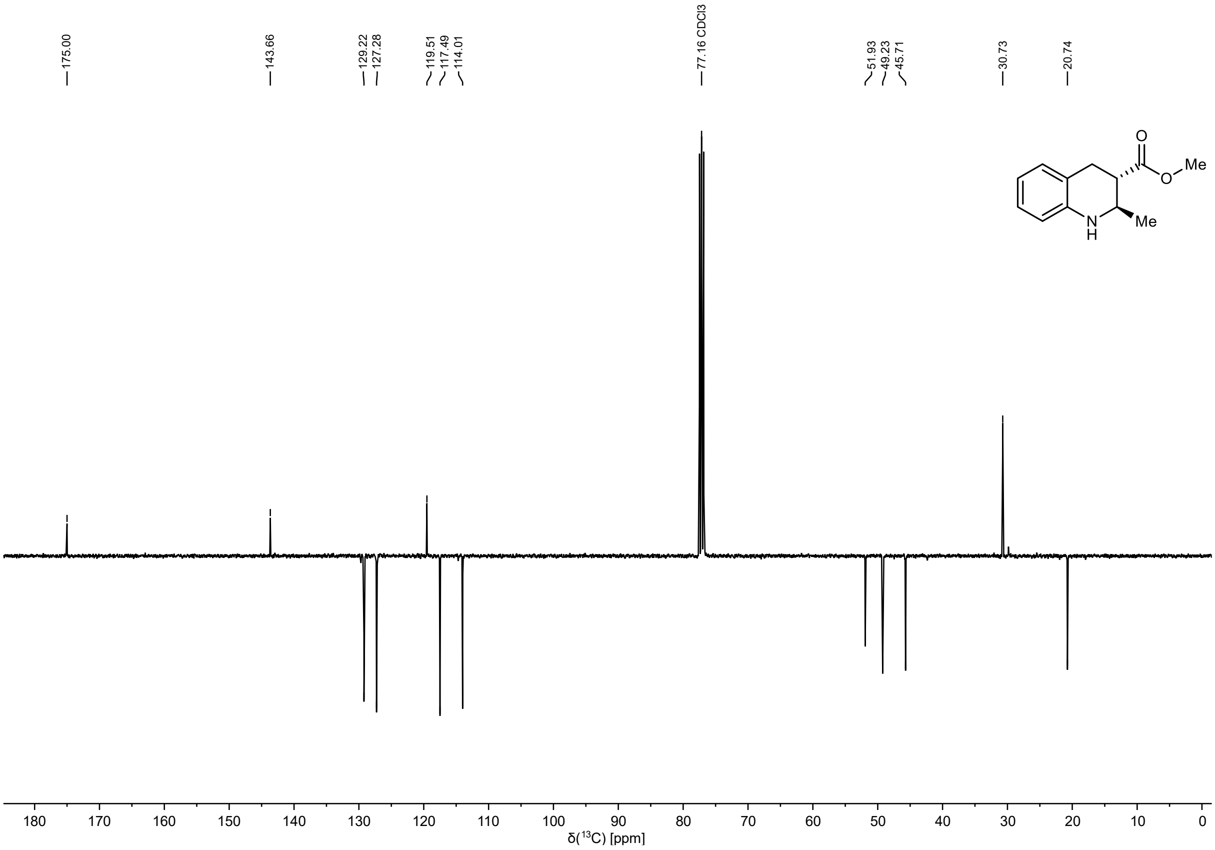

1H NMR (400 MHz, $CDCl_3$) major Diastereomer **SI-11d**

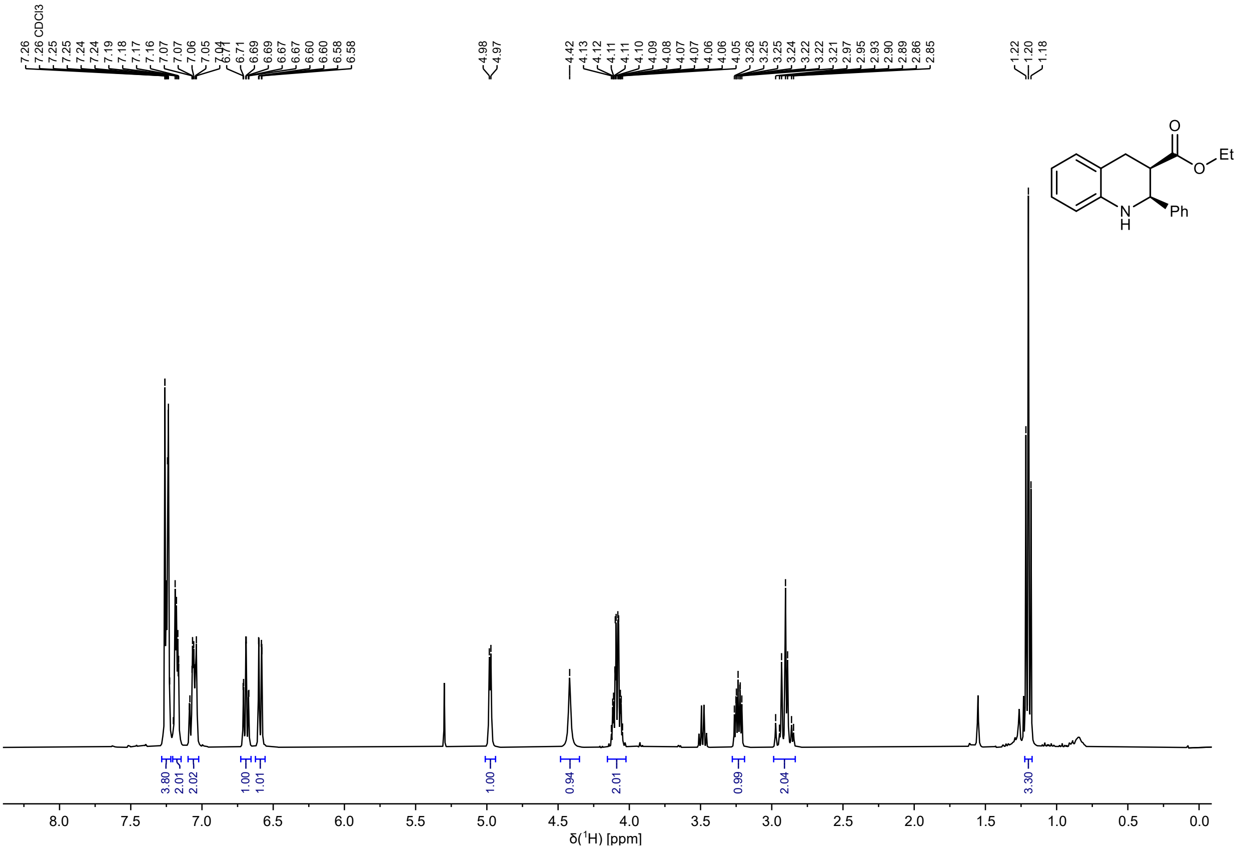

1H NMR (400 MHz, $CDCl_3$) minor Diastereomer **SI-11d**

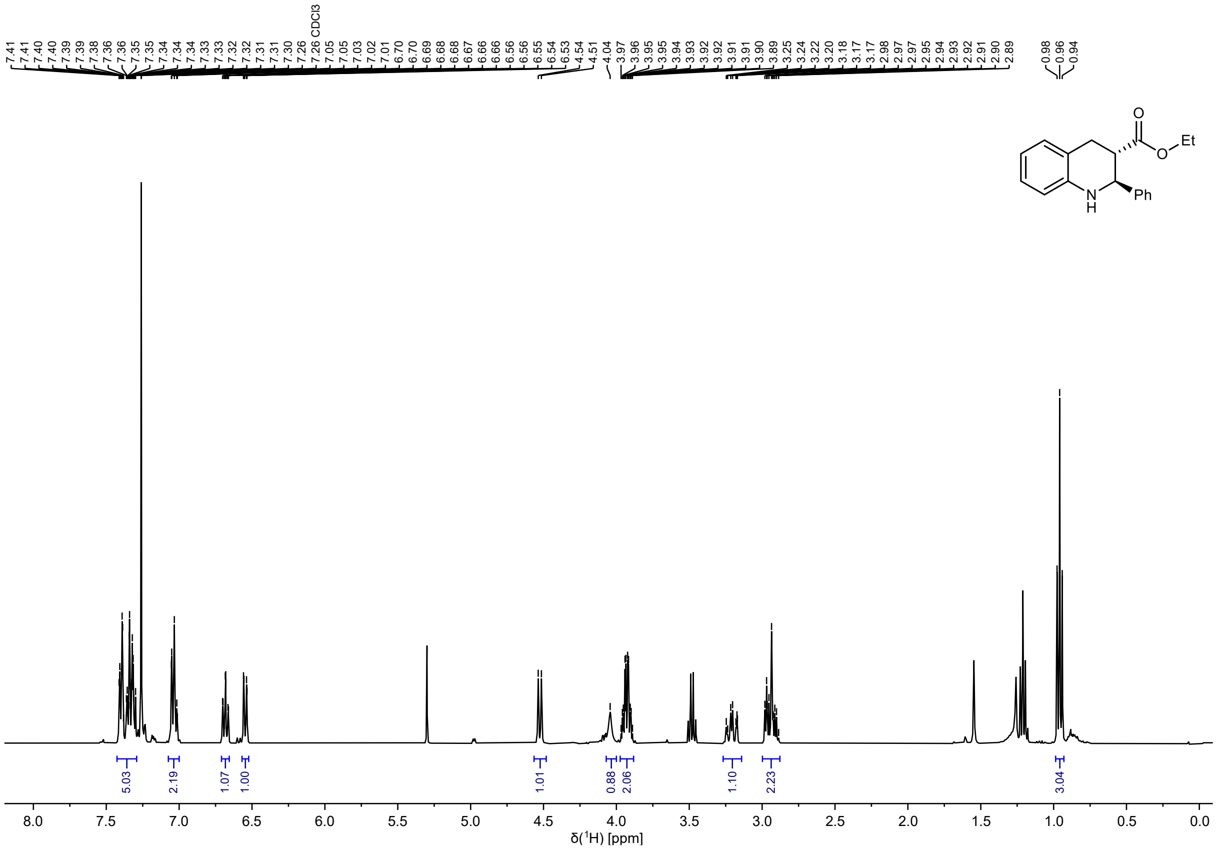

13C (APT) NMR (75 MHz, $CDCl_3$) **SI-11d**

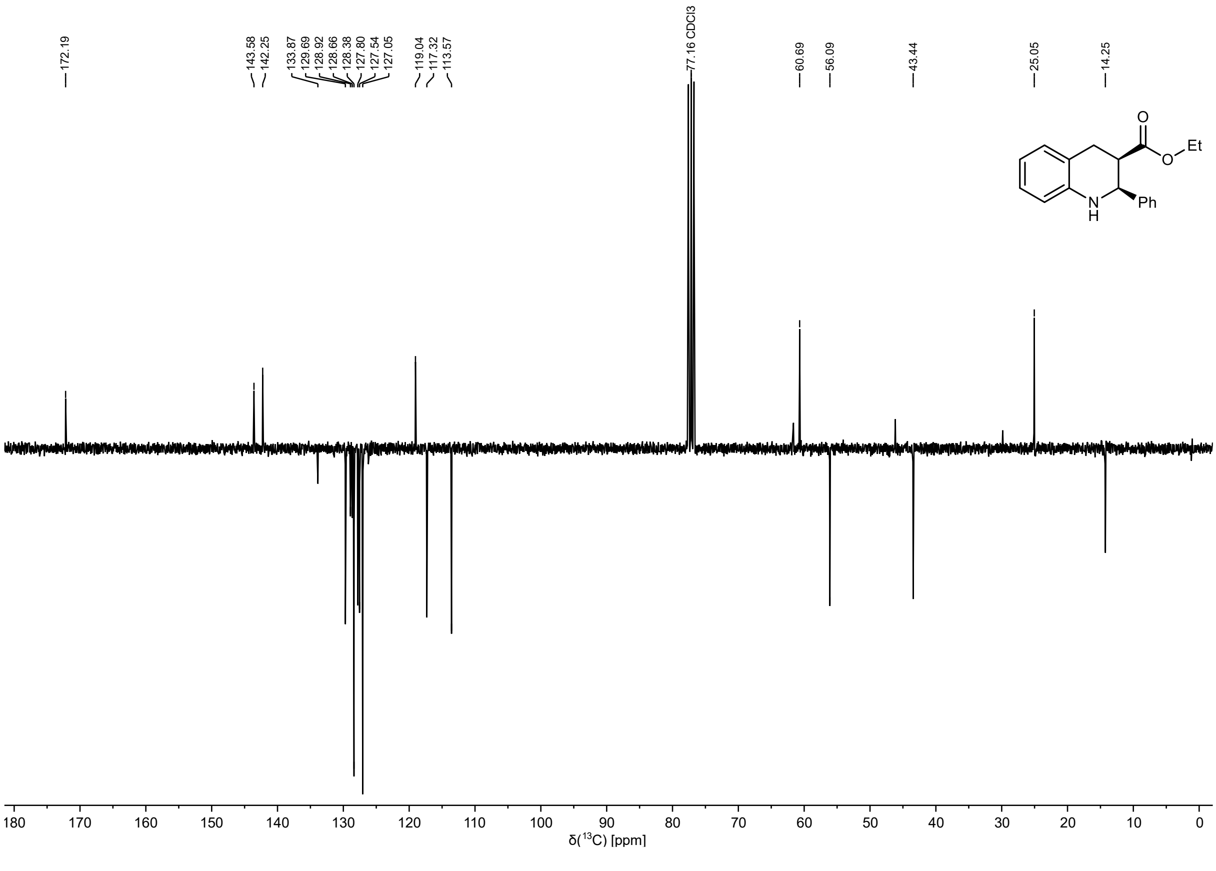

1H NMR (400 MHz, $CDCl_3$) major Diastereomer **SI-11e**

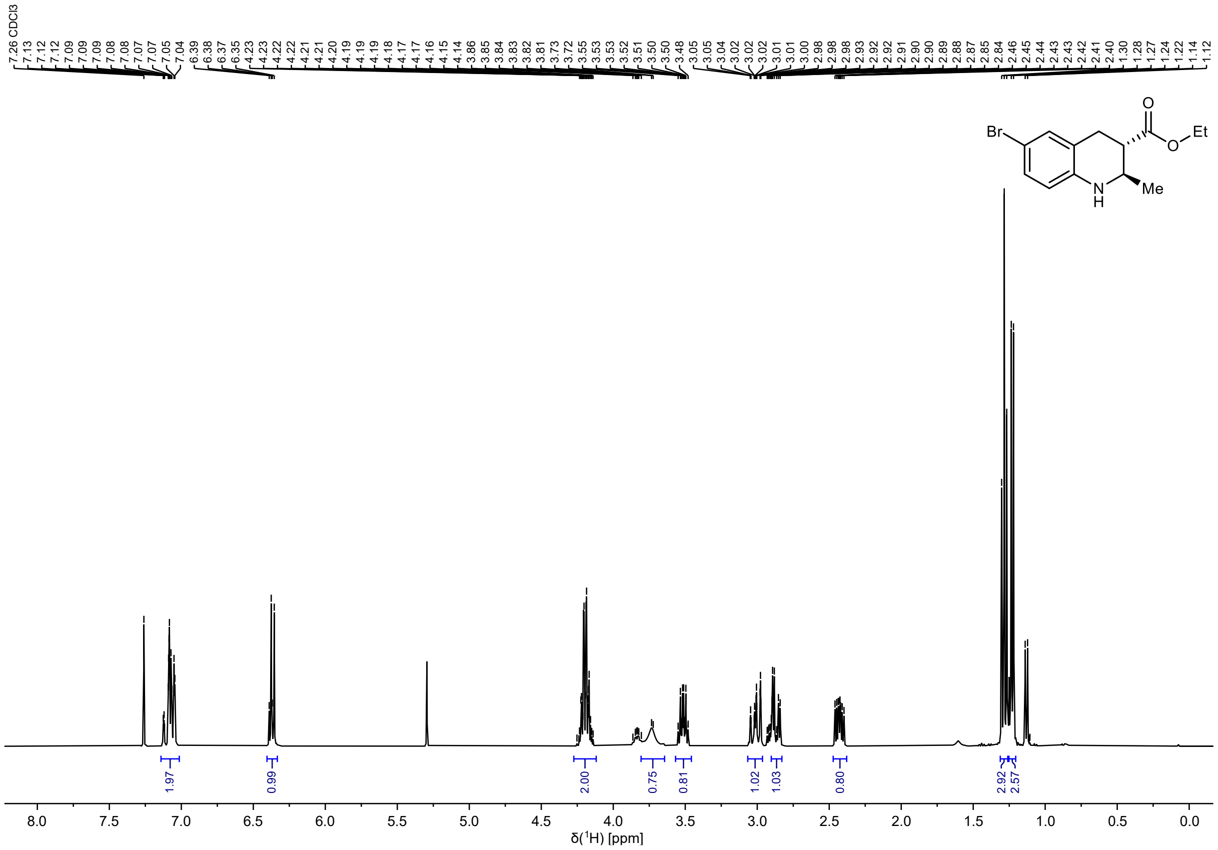

1H NMR (400 MHz, $CDCl_3$) minor Diastereomer **SI-11e**

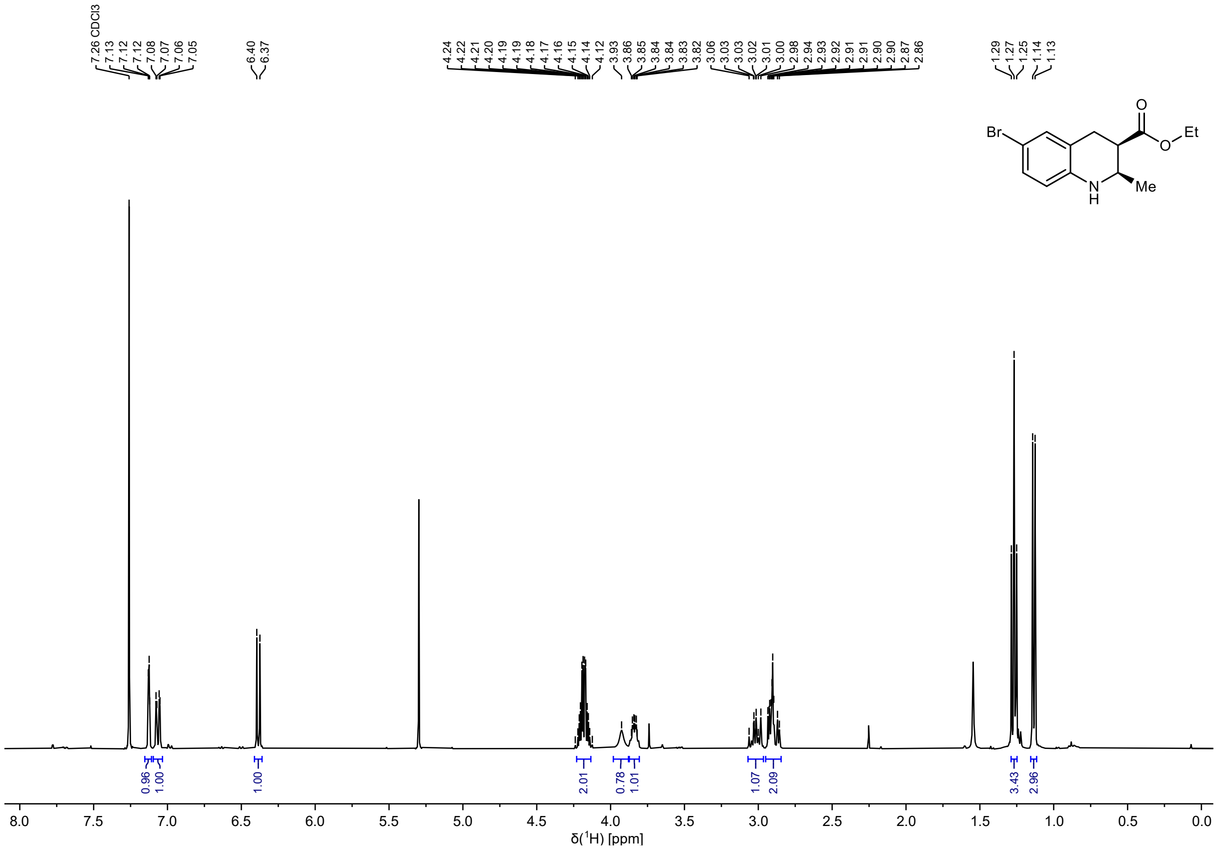

13C (APT) NMR (100 MHz, $CDCl_3$) **SI-11e**

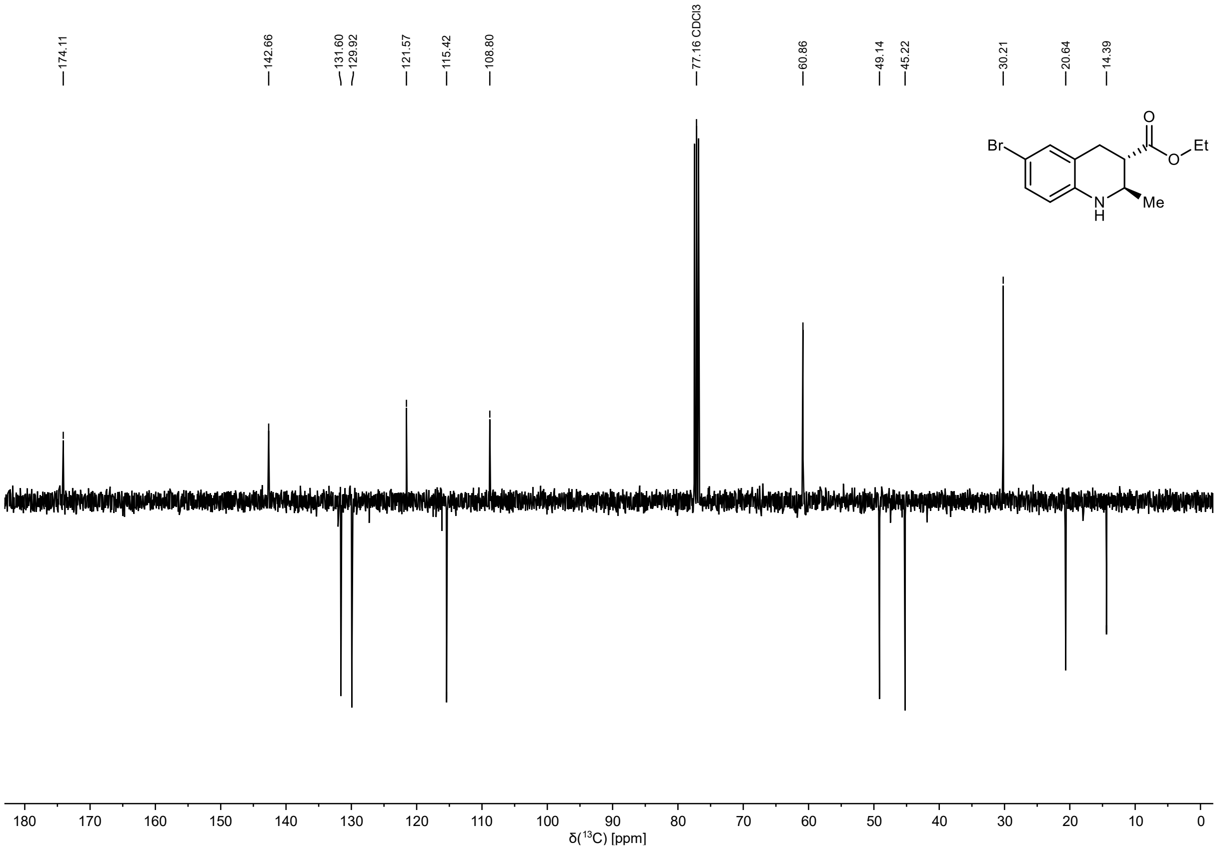

1H NMR (400 MHz, $CDCl_3$) **SI-15a**

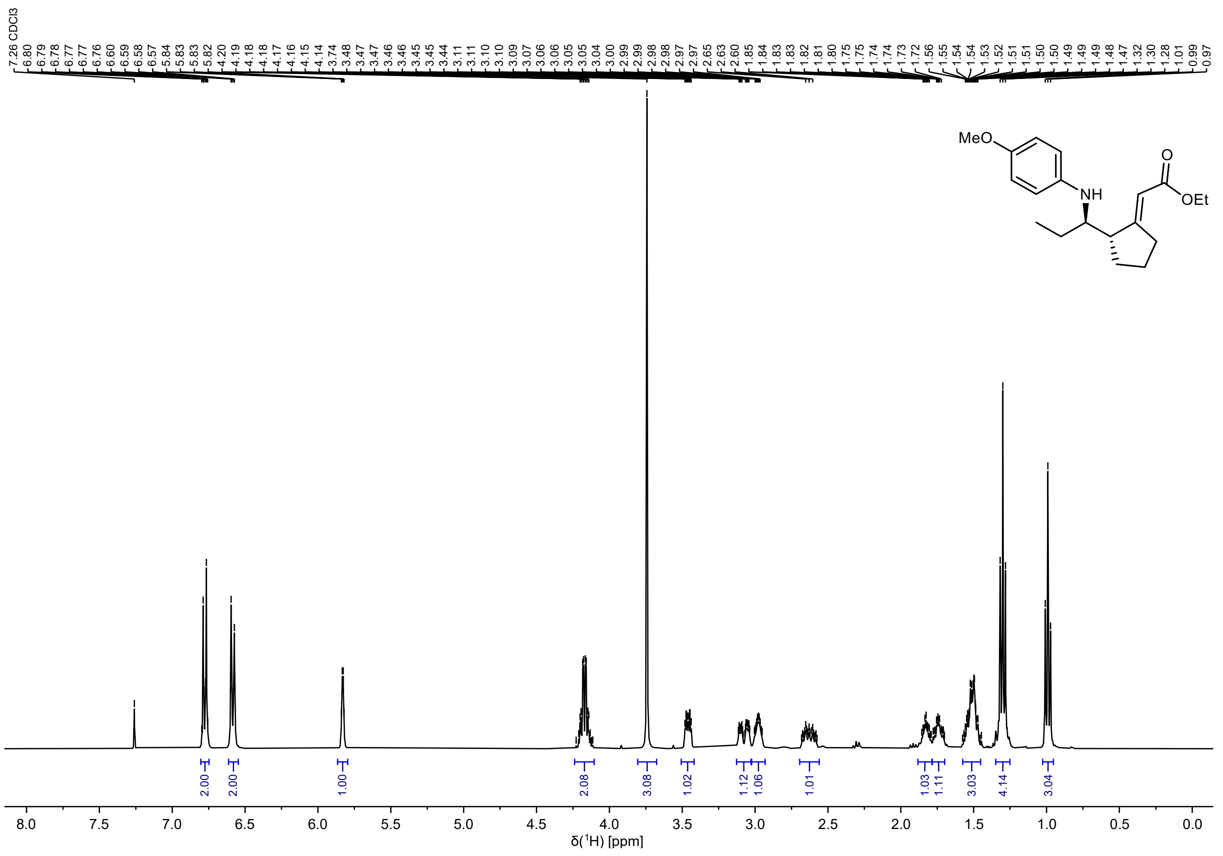

13C (APT) NMR (100 MHz, $CDCl_3$) **SI-15a**

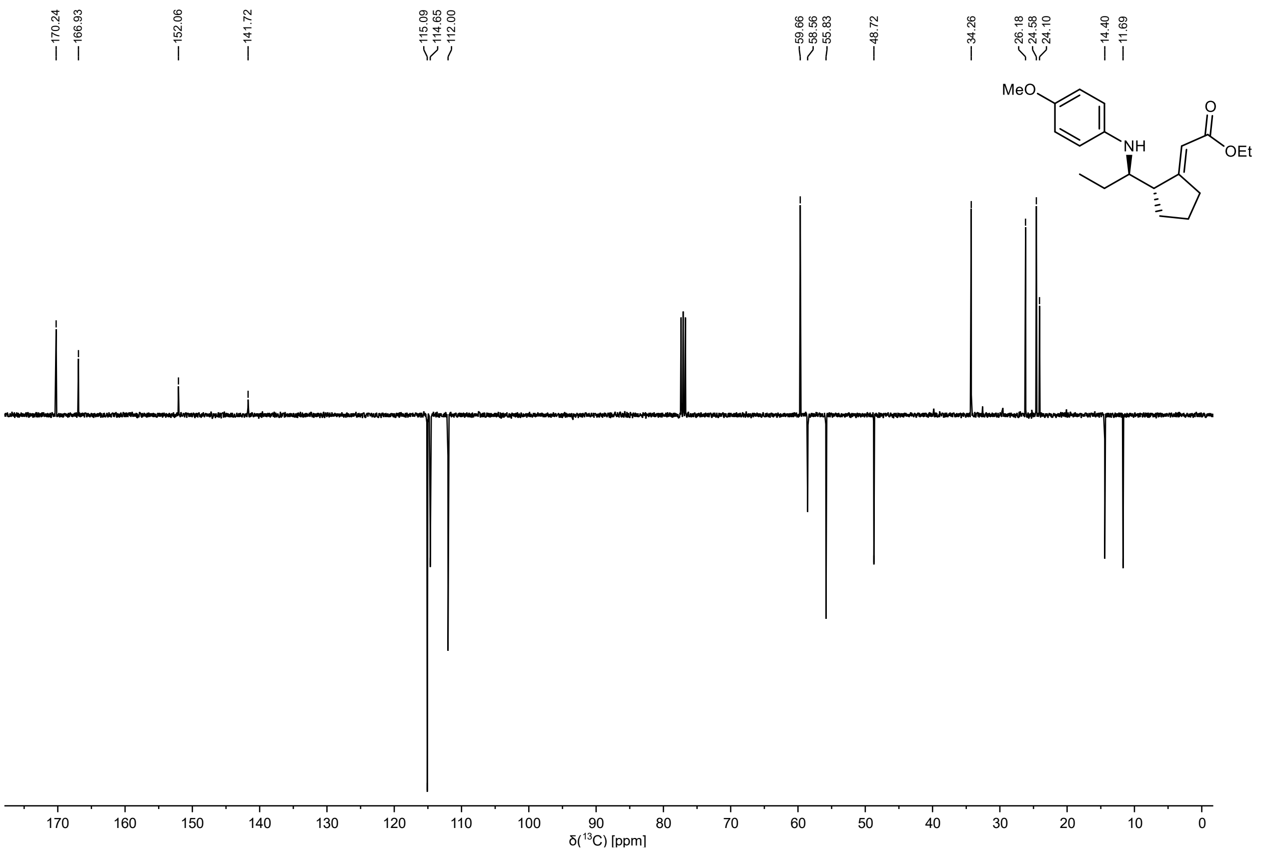

1H NMR (400 MHz, $CDCl_3$) **SI-15b**

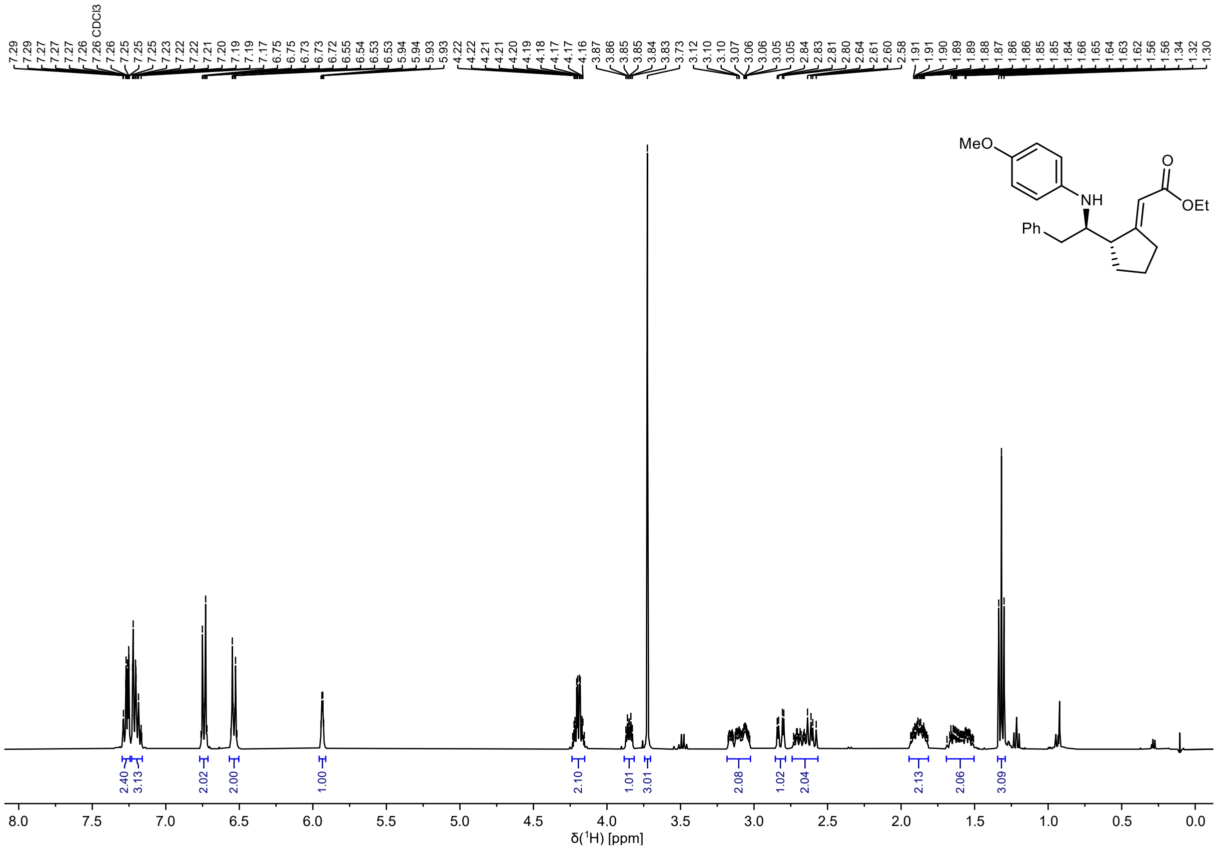

13C (APT) NMR (100 MHz, $CDCl_3$) **SI-15b**

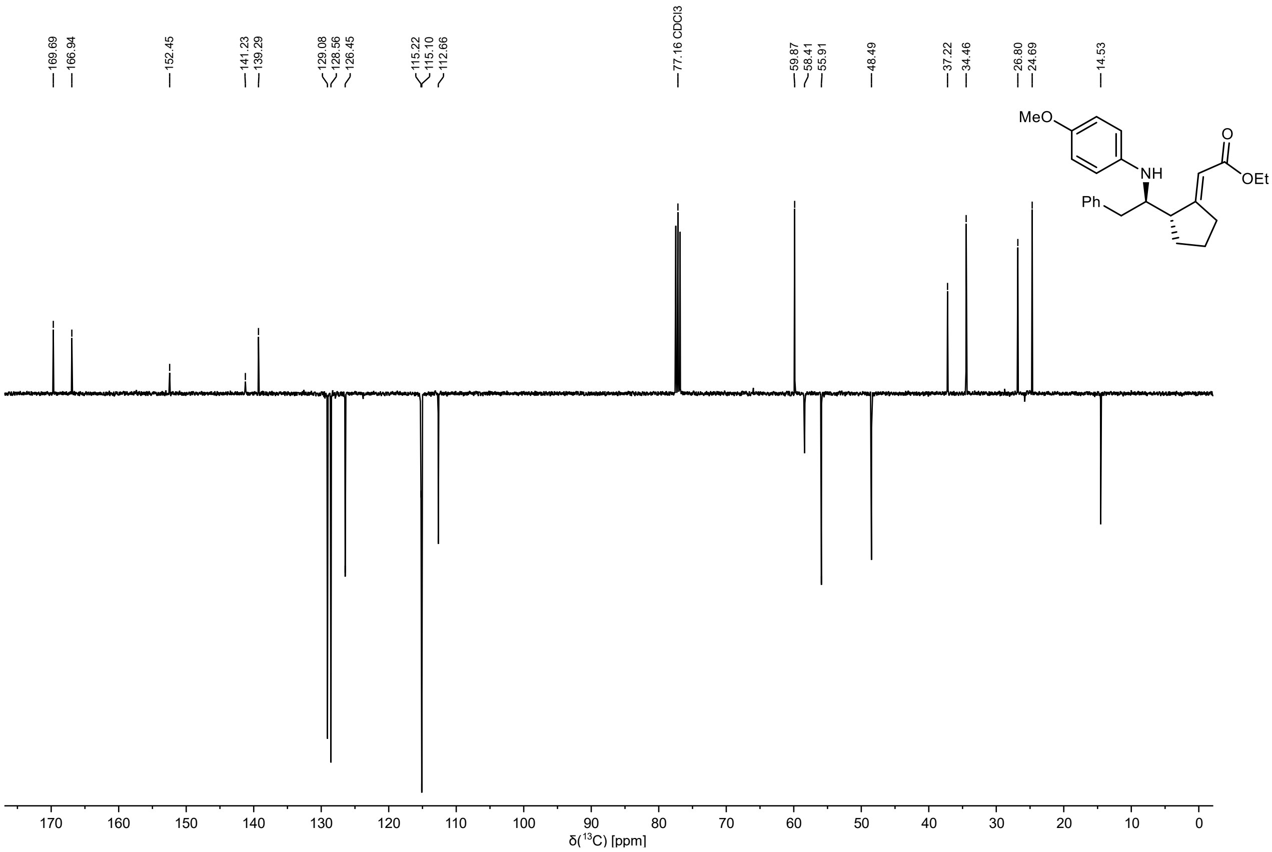

1H NMR (400 MHz, $CDCl_3$) **SI-15c**

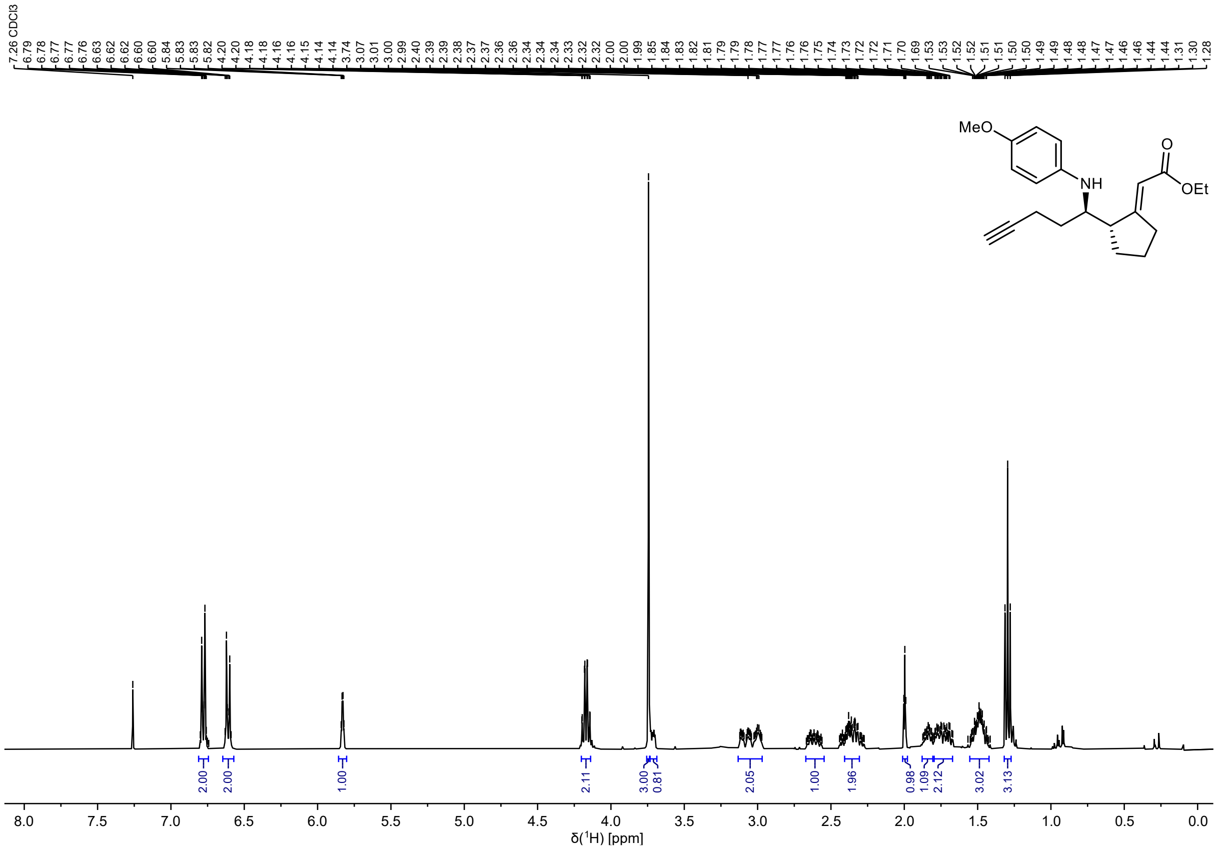

13C (APT) NMR (100 MHz, $CDCl_3$) **SI-15c**

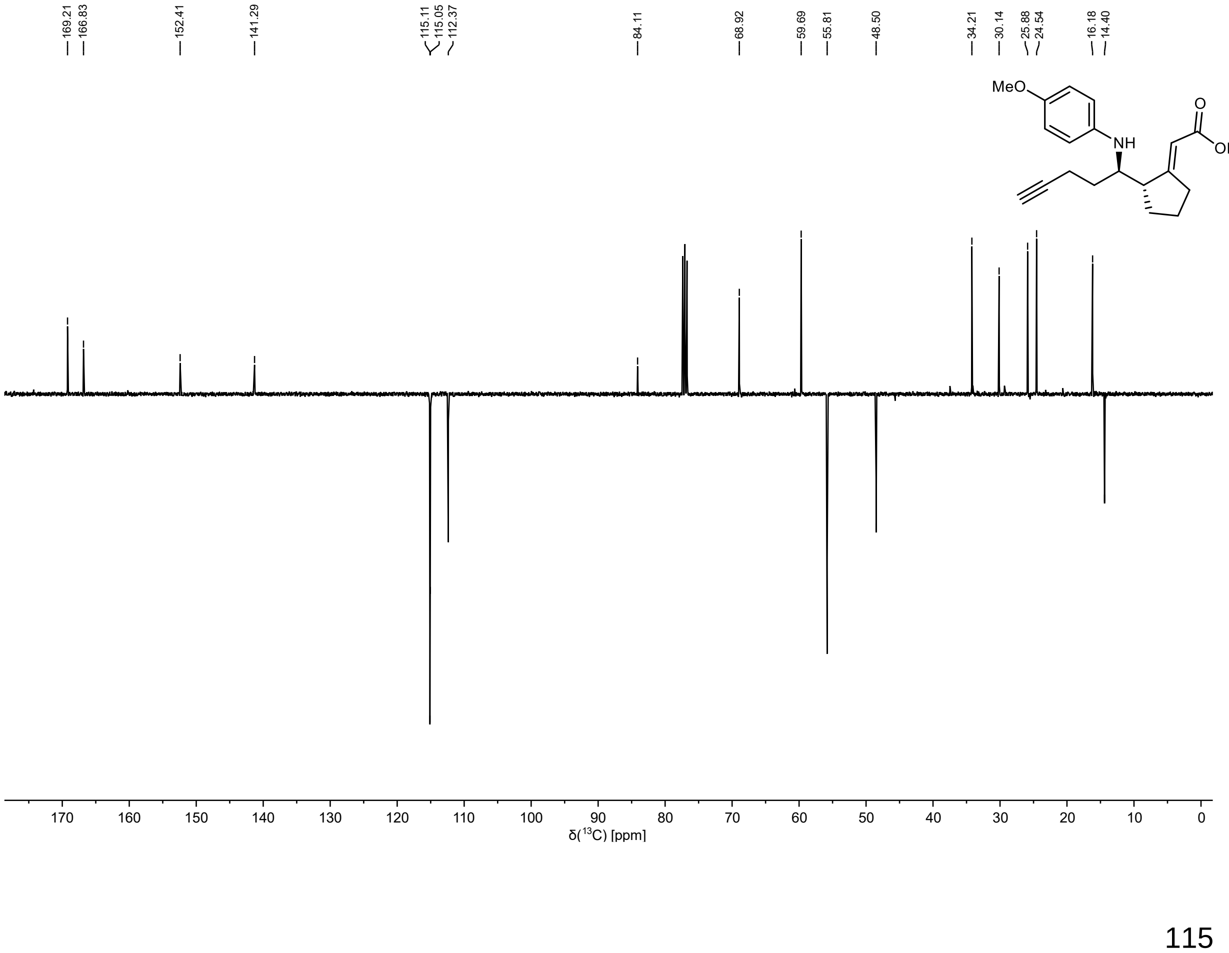

1H NMR (400 MHz, CDCl3) **SI-15d**

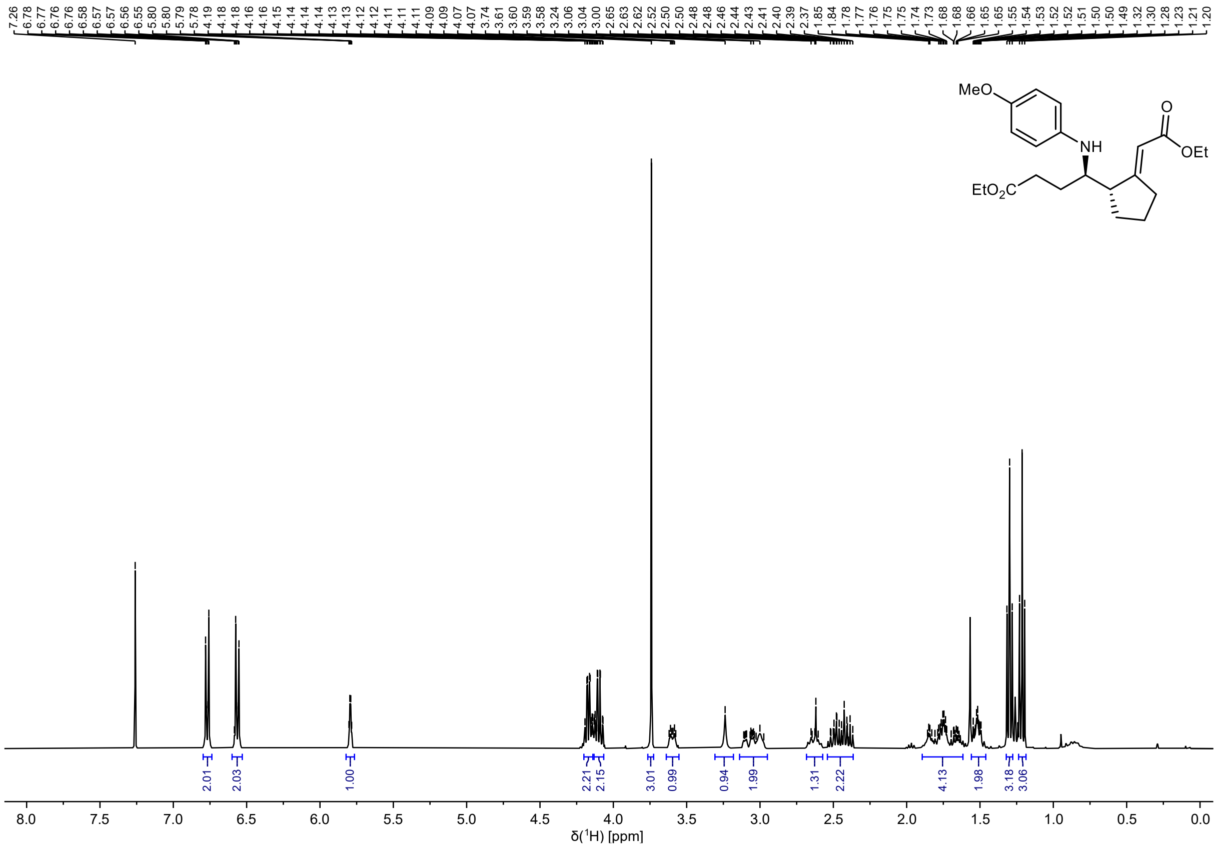

13C (APT) NMR (100 MHz, CDCl3) **SI-15d**

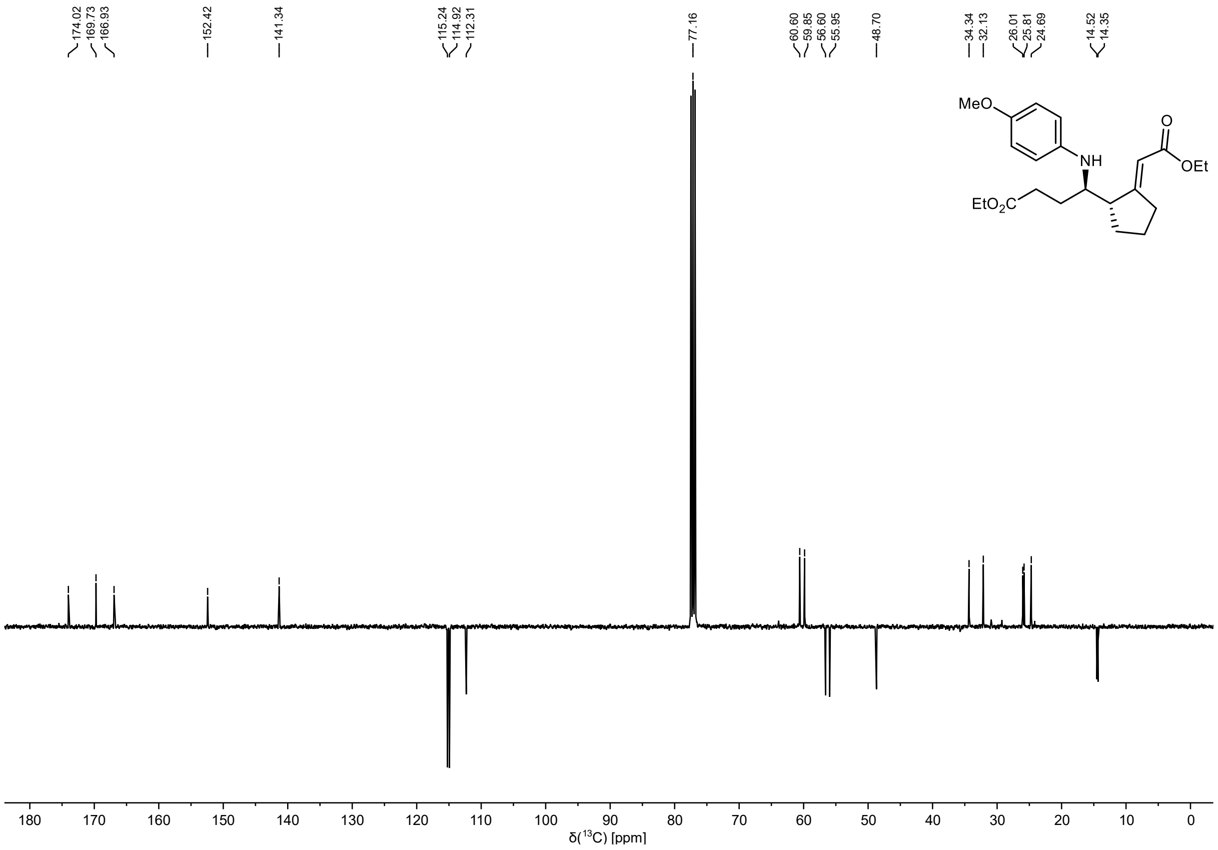

1H NMR (400 MHz, $CDCl_3$) **SI-15e**

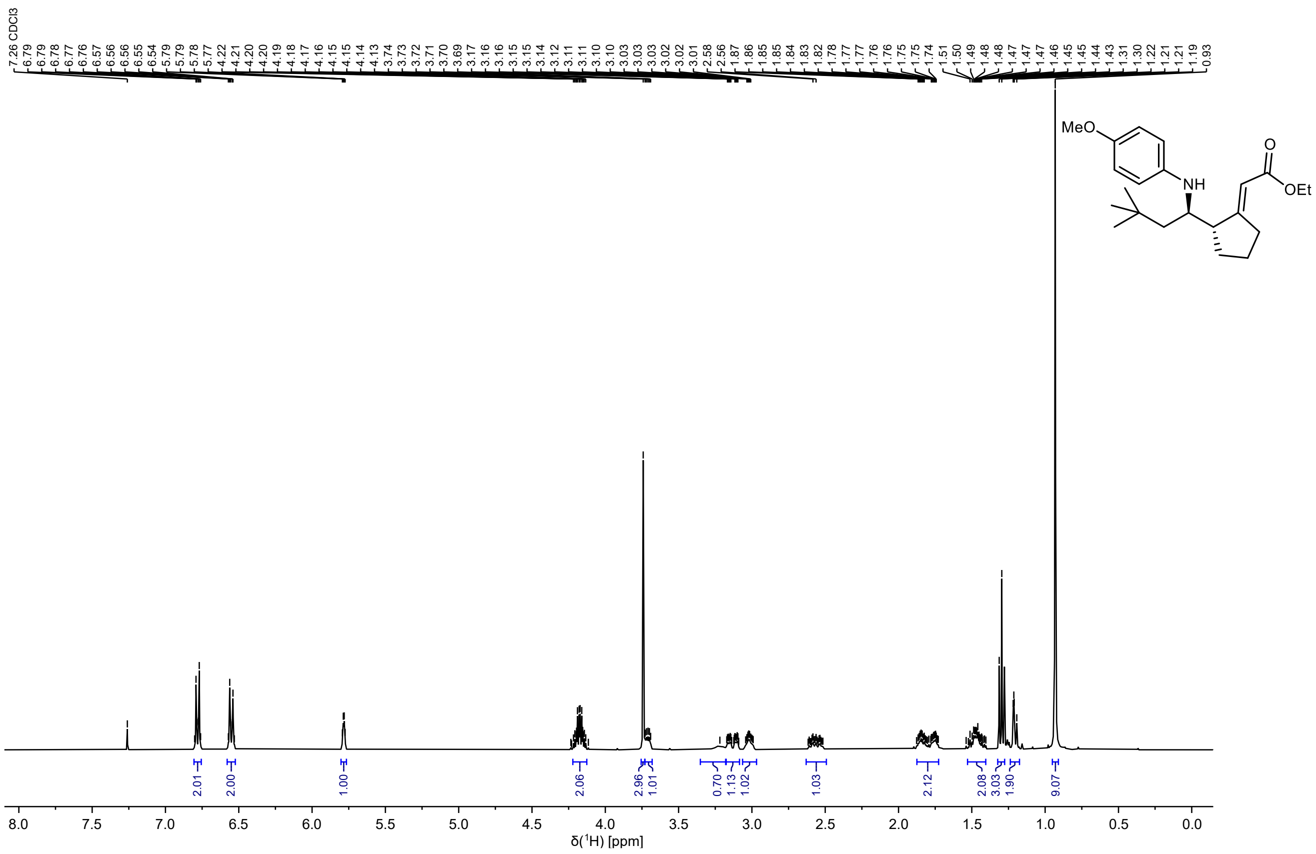

13C (APT) NMR (100 MHz, $CDCl_3$) **SI-15e**

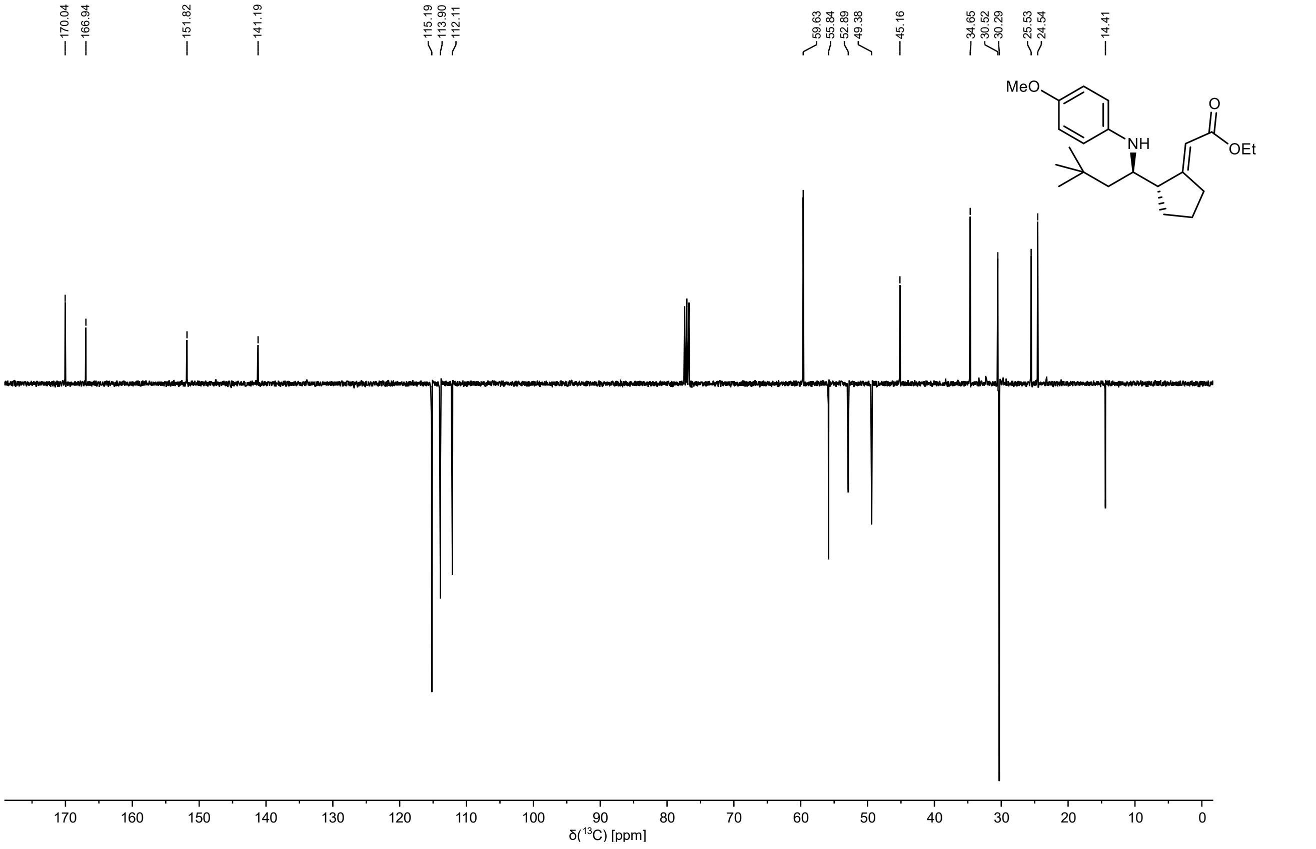

4. HPLC Chromatograms

rac- and (*S*)-2-Phenyl-1,2,3,4-tetrahydroquinoline **SI-5a**:

IB-column: Hex/ *i*PrOH 95:5 $^{v}/_{v}$, 0.5 ml/ min, λ = 300 nm

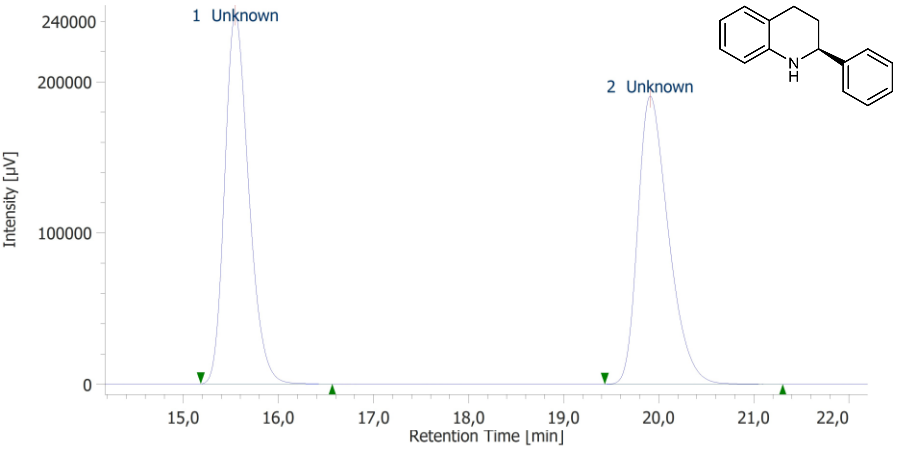

#	Peak Name	CH	tR [min]	Area [µV·sec]	Height [µV]	Area%	Height%	Quantity	NTP	Resolution	Symmetry Factor	Warning
1	Unknown	11	15,547	4295892	244508	50,112	56,223	N/A	18568	8,414	1,245	
2	Unknown	11	19,907	4276645	190381	49,888	43,777	N/A	18667	N/A	1,398	

Batch-process:

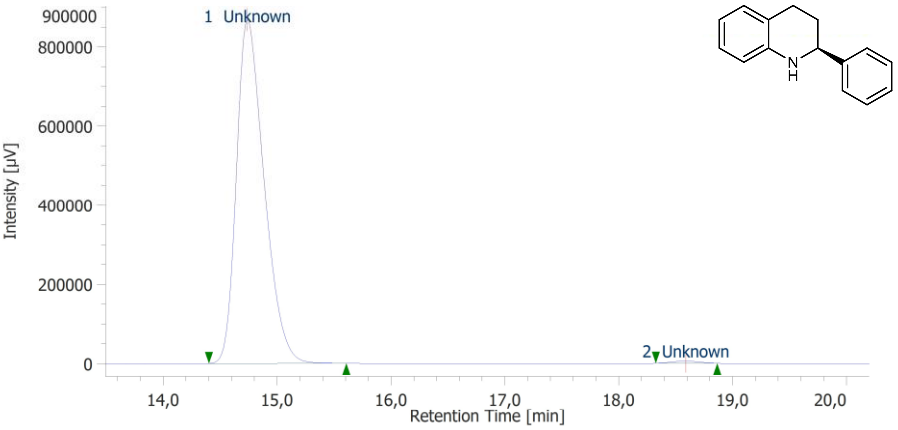

#	Peak Name	CH	tR [min]	Area [µV·sec]	Height [µV]	Area%	Height%	Quantity	NTP	Resolution	Symmetry Factor	Warning
1	Unknown	12	14,740	14643190	867042	99,345	99,336	N/A	17910	8,514	1,378	
2	Unknown	12	18,587	96512	5793	0,655	0,664	N/A	25516	N/A	1,022	

Flow-Process:

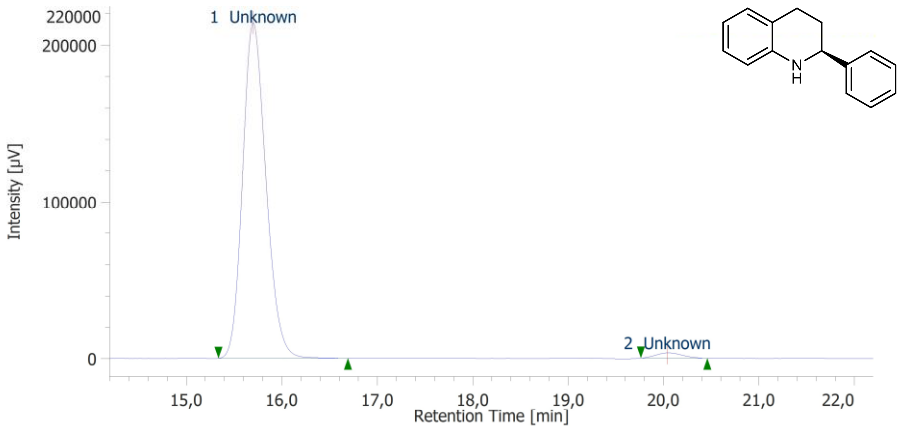

#	Peak Name	CH	tR [min]	Area [µV·sec]	Height [µV]	Area%	Height%	Quantity	NTP	Resolution	Symmetry Factor	Warning
1	Unknown	11	15,700	3624373	214051	98,239	98,440	N/A	20135	9,023	1,189	
2	Unknown	11	20,037	64970	3392	1,761	1,560	N/A	23641	N/A	1,181	

rac- and (*S*)-2-(4-Methoxyphenyl)-1,2,3,4-tetrahydroquinoline **SI-5b**:

IB-column: Hex/ *i*PrOH 90:10 $^{v}/_{v}$, 1.0 ml/ min, λ = 250 nm

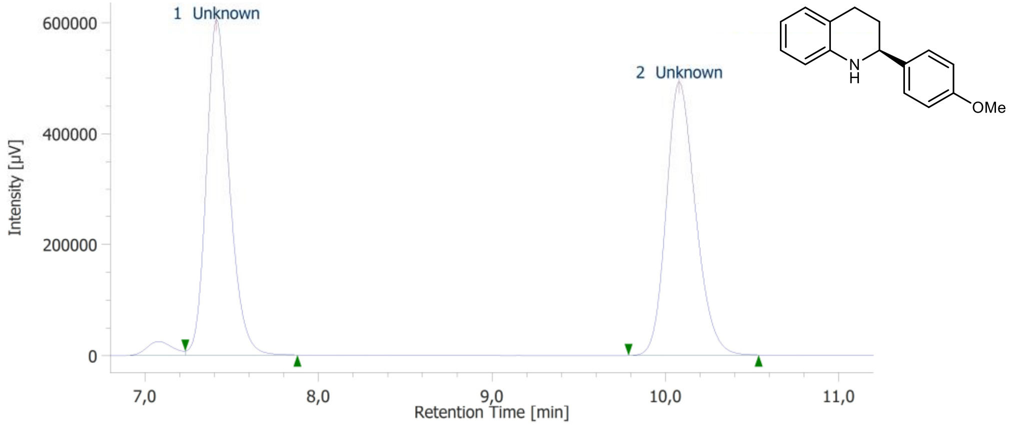

#	Peak Name	CH	tR [min]	Area [µV·sec]	Height [µV]	Area%	Height%	Quantity	NTP	Resolution	Symmetry Factor	Warning
1	Unknown	11	7,413	5599151	604814	49,244	55,129	N/A	15701	9,893	1,221	
2	Unknown	11	10,080	5771182	492277	50,756	44,871	N/A	17601	N/A	1,197	

Batch-process:

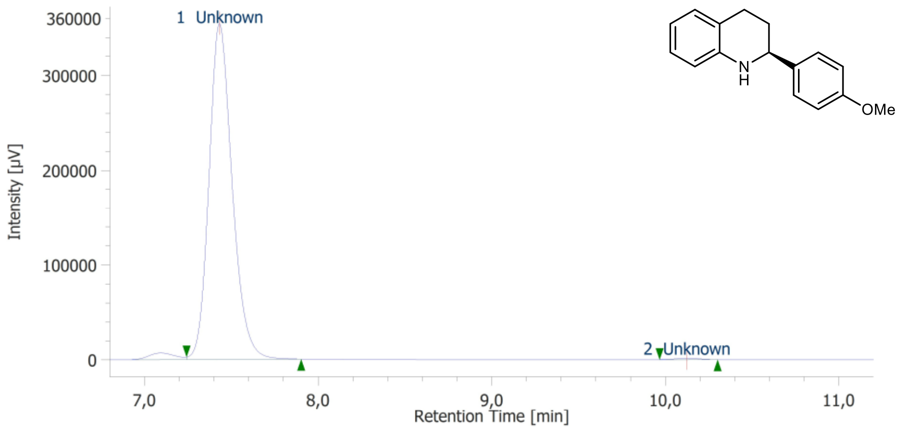

#	Peak Name	CH	tR [min]	Area [µV·sec]	Height [µV]	Area%	Height%	Quantity	NTP	Resolution	Symmetry Factor	Warning
1	Unknown	11	7,433	3284662	353862	99,558	99,595	N/A	15571	10,382	1,193	
2	Unknown	11	10,123	14578	1439	0,442	0,405	N/A	20723	N/A	1,064	

Flow-process:

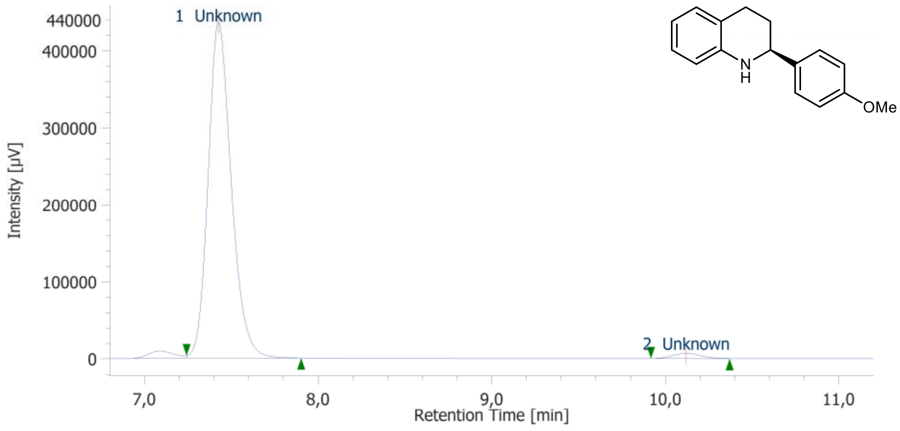

#	Peak Name	CH	tR [min]	Area [µV·sec]	Height [µV]	Area%	Height%	Quantity	NTP	Resolution	Symmetry Factor	Warning
1	Unknown	11	7,427	4040698	436341	98,162	98,470	N/A	15643	10,101	1,212	
2	Unknown	11	10,120	75654	6781	1,838	1,530	N/A	18552	N/A	1,077	

rac- and (*S*)-2-(4-Bromophenyl)-1,2,3,4-tetrahydroquinoline **SI-5c**:

IB-column: Hex/ *i*PrOH 90:10 $^{v}/_{v}$, 1.0 ml/ min, λ = 244 nm

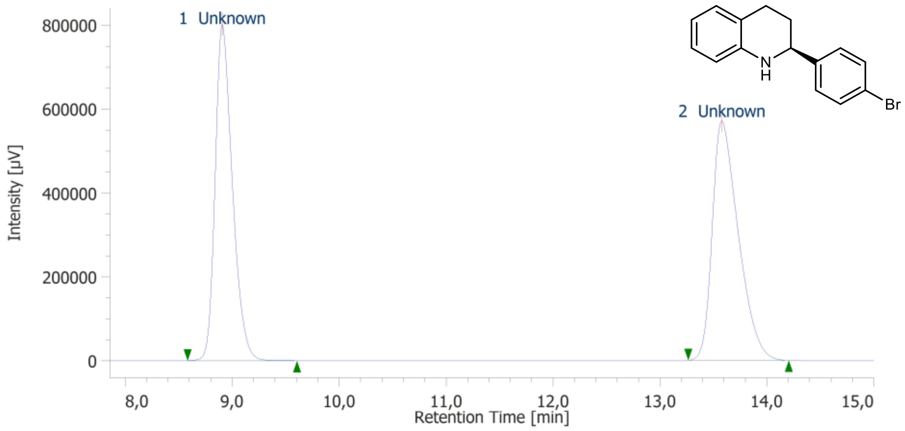

#	Peak Name	CH	tR [min]	Area [µV·sec]	Height [µV]	Area%	Height%	Quantity	NTP	Resolution	Symmetry Factor	Warning
1	Unknown	11	8,907	8990834	800590	48,201	58,371	N/A	15247	12,855	1,323	
2	Unknown	11	13,577	9661956	570965	51,799	41,629	N/A	15234	N/A	1,491	

Batch-process

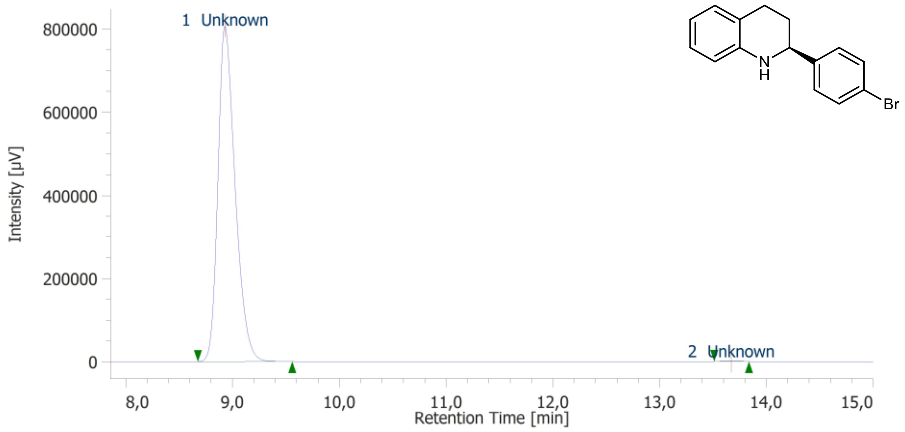

#	Peak Name	CH	tR [min]	Area [µV·sec]	Height [µV]	Area%	Height%	Quantity	NTP	Resolution	Symmetry Factor	Warning
1	Unknown	11	8,930	9013951	805564	99,816	99,817	N/A	15365	15,535	1,326	
2	Unknown	11	13,670	16599	1477	0,184	0,183	N/A	28538	N/A	1,022	

Flow-process

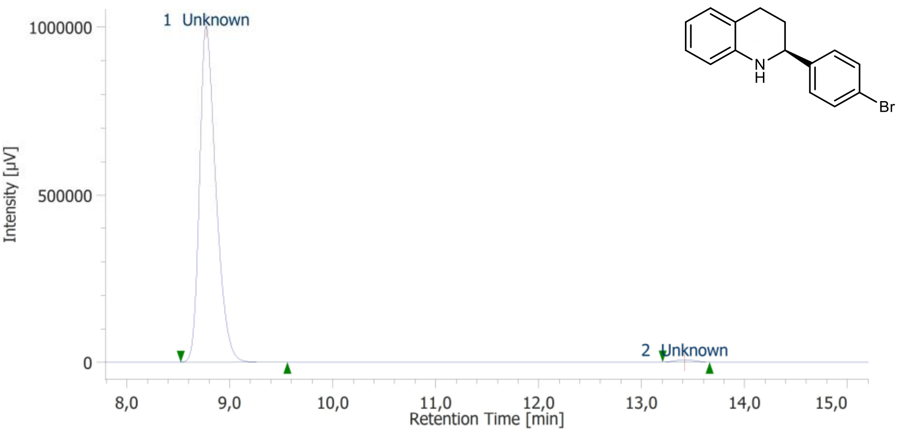

#	Peak Name	CH	tR [min]	Area [µV·sec]	Height [µV]	Area%	Height%	Quantity	NTP	Resolution	Symmetry Factor	Warning
1	Unknown	11	8,770	11080861	1002090	99,144	99,299	N/A	15274	14,187	1,370	
2	Unknown	11	13,417	95698	7077	0,856	0,701	N/A	20707	N/A	1,062	

rac- and (*S*)-6-Bromo-2-phenyl-1,2,3,4-tetrahydroquinoline **SI-5d**:

IB-column: Hex/ *i*PrOH 95:5 $^{v}/_{v}$, 1.0 ml/ min, λ = 256 nm

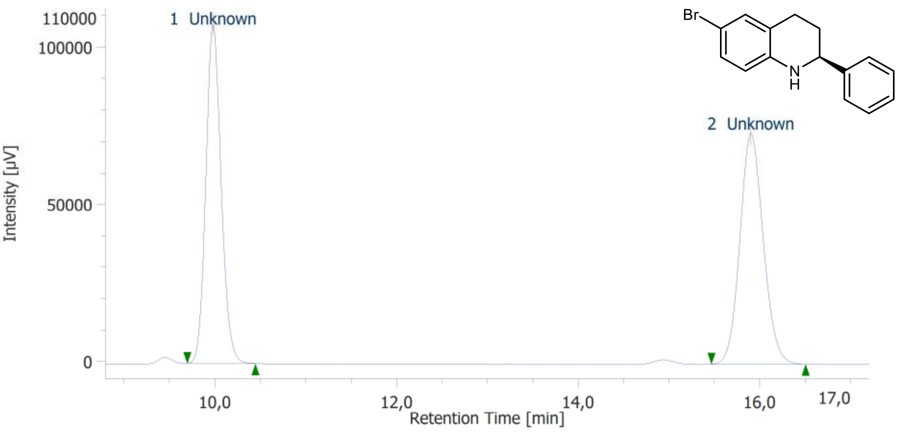

#	Peak Name	CH	tR [min]	Area [µV·sec]	Height [µV]	Area%	Height%	Quantity	NTP	Resolution	Symmetry Factor	Warning
1	Unknown	11	9,980	1261780	107777	48,997	59,487	N/A	17221	15,401	1,124	
2	Unknown	11	15,900	1313421	73399	51,003	40,513	N/A	18577	N/A	1,112	

Batch-process

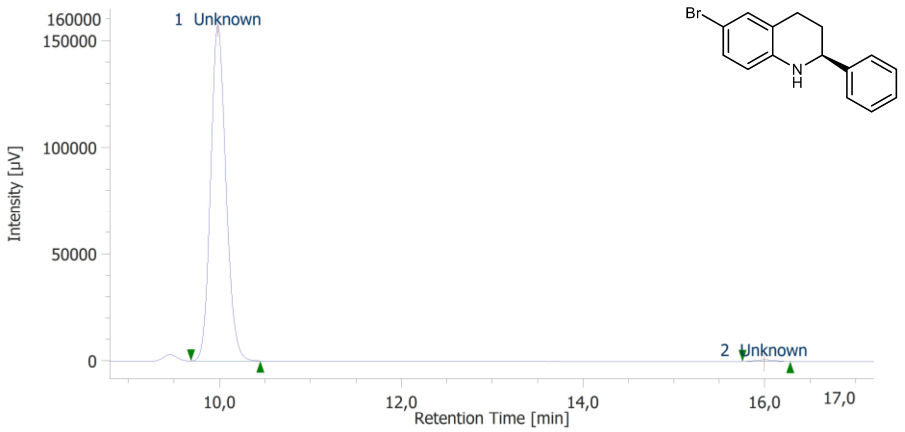

#	Peak Name	CH	tR [min]	Area [µV·sec]	Height [µV]	Area%	Height%	Quantity	NTP	Resolution	Symmetry Factor	Warning
1	Unknown	11	9,980	1843566	156904	99,324	99,486	N/A	17125	16,389	1,143	
2	Unknown	11	15,990	12542	811	0,676	0,514	N/A	22089	N/A	1,098	

Flow-process

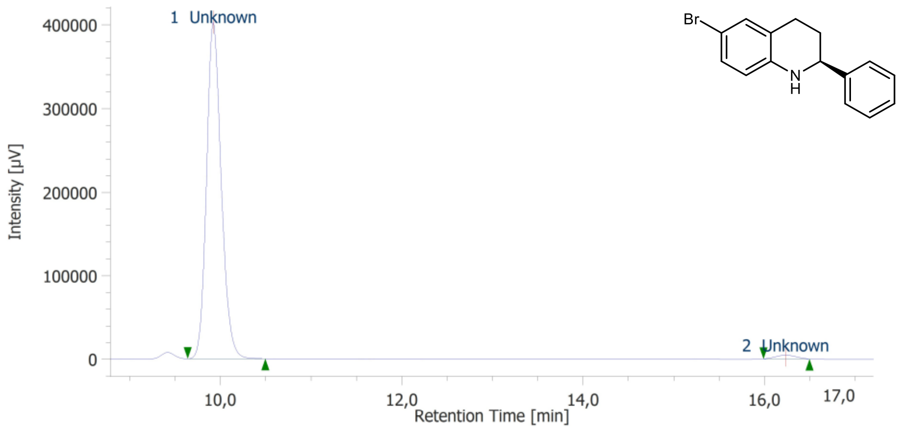

#	Peak Name	CH	tR [min]	Area [µV·sec]	Height [µV]	Area%	Height%	Quantity	NTP	Resolution	Symmetry Factor	Warning
1	Unknown	11	9,923	4540664	402178	98,504	98,901	N/A	18505	17,582	1,170	
2	Unknown	11	16,230	68960	4469	1,496	1,099	N/A	23059	N/A	1,038	

rac- and (*R*)-3-Phenyl-3,4-dihydro-2H-benzo[b][1,4]oxazine **SI-32**:

IB-column: Hex/ *i*PrOH 90:10 $^{v}/_{v}$, 1.0 ml/ min, λ = 246 nm

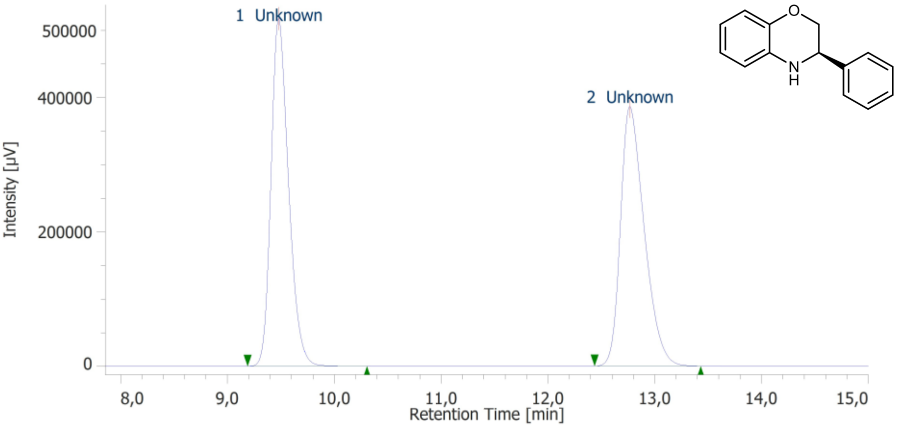

#	Peak Name	CH	tR [min]	Area [µV·sec]	Height [µV]	Area%	Height%	Quantity	NTP	Resolution	Symmetry Factor	Warning
1	Unknown	11	9,480	5891324	516718	49,732	57,247	N/A	16625	9,492	1,252	
2	Unknown	11	12,767	5954923	385899	50,268	42,753	N/A	16279	N/A	1,380	

Batch-process:

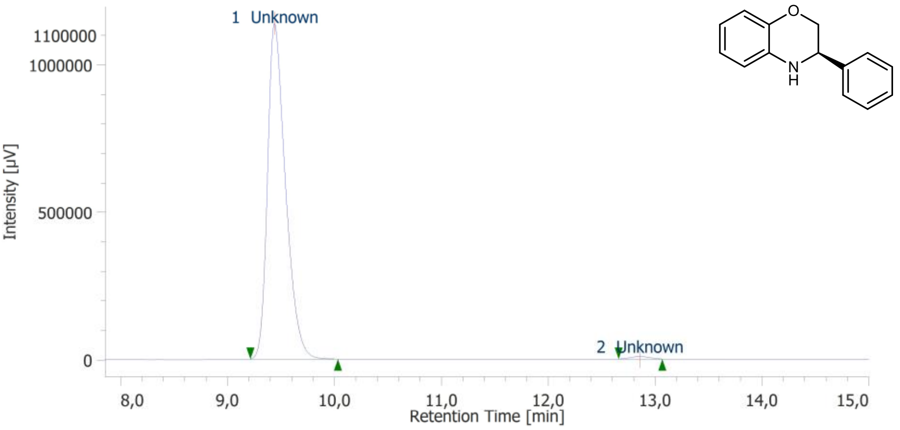

#	Peak Name	CH	tR [min]	Area [µV·sec]	Height [µV]	Area%	Height%	Quantity	NTP	Resolution	Symmetry Factor	Warning
1	Unknown	11	9,440	13286622	1136704	99,205	99,259	N/A	15789	10,556	1,407	
2	Unknown	11	12,857	106531	8489	0,795	0,741	N/A	21766	N/A	1,027	

Flow-process:

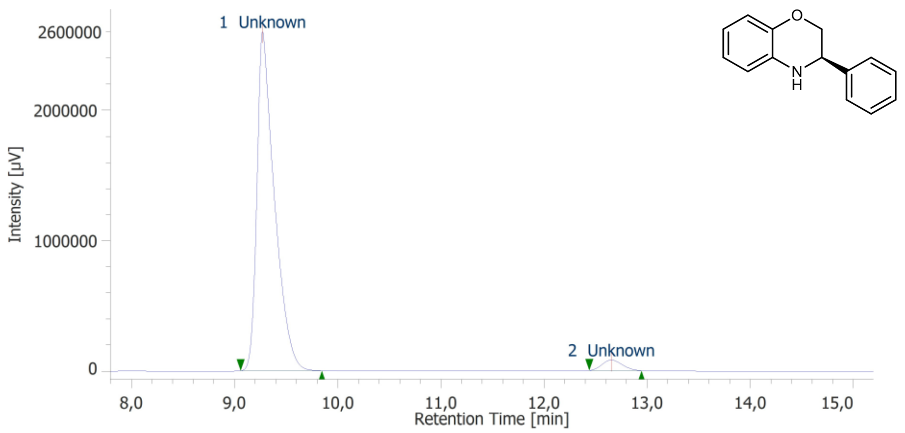

#	Peak Name	CH	tR [min]	Area [µV·sec]	Height [µV]	Area%	Height%	Quantity	NTP	Resolution	Symmetry Factor	Warning
1	Unknown	11	9,270	30658258	2595721	96,558	96,984	N/A	15481	10,223	1,720	
2	Unknown	11	12,650	1092831	80719	3,442	3,016	N/A	19220	N/A	1,158	

Flow-process, Up-Scaling:

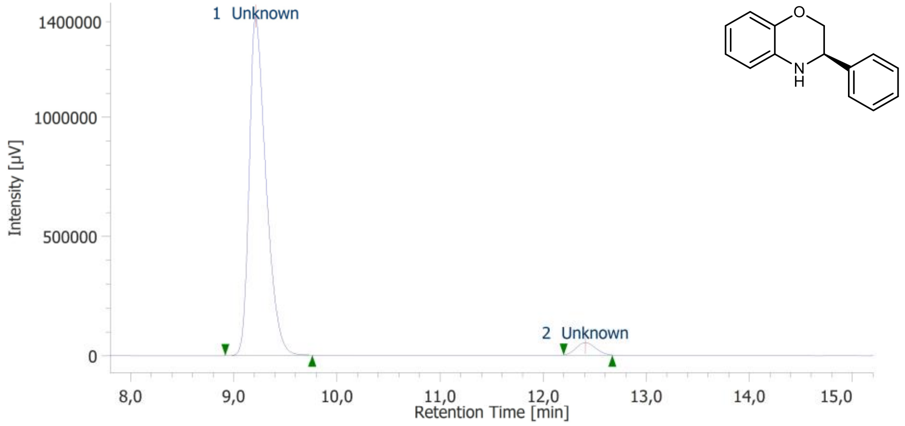

#	Peak Name	CH	tR [min]	Area [µV·sec]	Height [µV]	Area%	Height%	Quantity	NTP	Resolution	Symmetry Factor	Warning
1	Unknown	11	9,210	16045012	1420013	95,976	96,464	N/A	16178	10,028	1,454	
2	Unknown	11	12,407	672722	52058	4,024	3,536	N/A	20146	N/A	1,113	

rac- and (2*R*,3*S*) 3-Ethylcarboxylate 2-methyl-1,2,3,4-tetrahydroquinoline **SI-11a**:

IB-column: Hex/ *i*PrOH 95:5 $^{v}/_{v}$, 1.0 ml/ min, λ = 248 nm

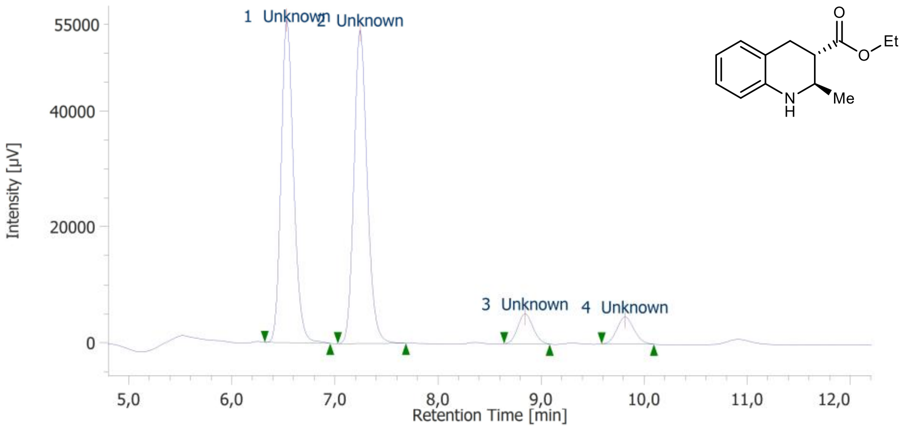

#	Peak Name	CH	tR [min]	Area [µV·sec]	Height [µV]	Area%	Height%	Quantity	NTP	Resolution	Symmetry Factor	Warning
1	Unknown	11	6,533	470595	55686	44,264	46,502	N/A	14411	3,174	1,190	
2	Unknown	11	7,243	484328	54133	45,556	45,205	N/A	15747	6,361	1,160	
3	Unknown	11	8,843	54150	5229	5,093	4,366	N/A	16730	3,375	1,094	
4	Unknown	11	9,813	54074	4702	5,086	3,927	N/A	16801	N/A	1,105	

Batch process:

major Diastereomer:

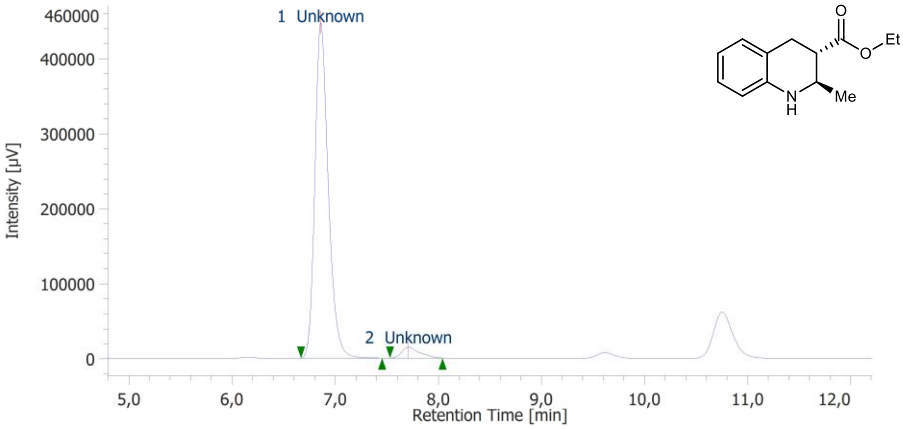

#	Peak Name	CH	tR [min]	Area [µV·sec]	Height [µV]	Area%	Height%	Quantity	NTP	Resolution	Symmetry Factor	Warning
1	Unknown	11	6,857	4024205	446904	95,903	96,874	N/A	14270	3,222	1,288	
2	Unknown	11	7,710	171924	14422	4,097	3,126	N/A	10464	N/A	1,457	

minor Diastereomer:

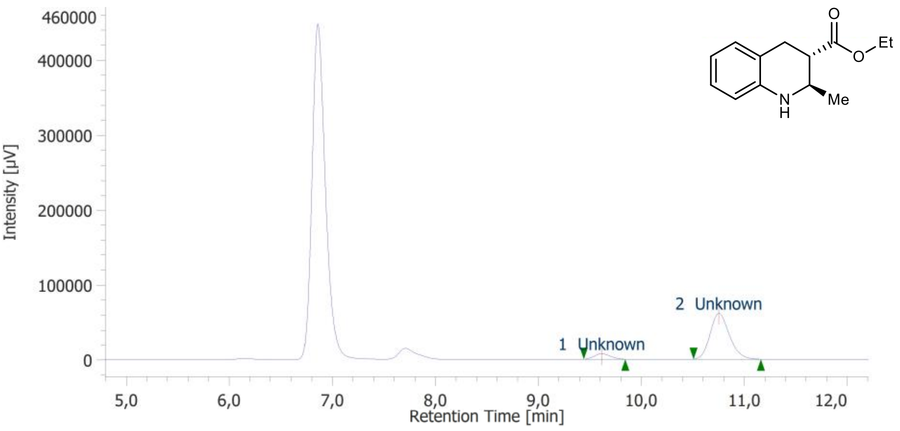

#	Peak Name	CH	tR [min]	Area [µV·sec]	Height [µV]	Area%	Height%	Quantity	NTP	Resolution	Symmetry Factor	Warning
1	Unknown	11	9,613	79816	7302	9,117	10,646	N/A	17019	3,602	1,139	
2	Unknown	11	10,750	795673	61285	90,883	89,354	N/A	16186	N/A	1,199	

Flow process:

major Diastereomer:

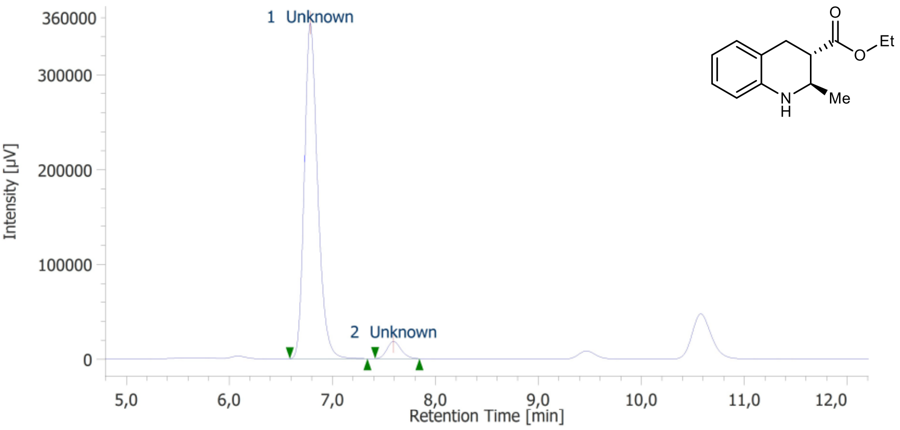

#	Peak Name	CH	tR [min]	Area [µV·sec]	Height [µV]	Area%	Height%	Quantity	NTP	Resolution	Symmetry Factor	Warning
1	Unknown	11	6,783	3169831	353687	94,859	95,114	N/A	14041	3,412	1,270	
2	Unknown	11	7,593	171785	18169	5,141	4,886	N/A	15105	N/A	1,163	

minor Diastereomer:

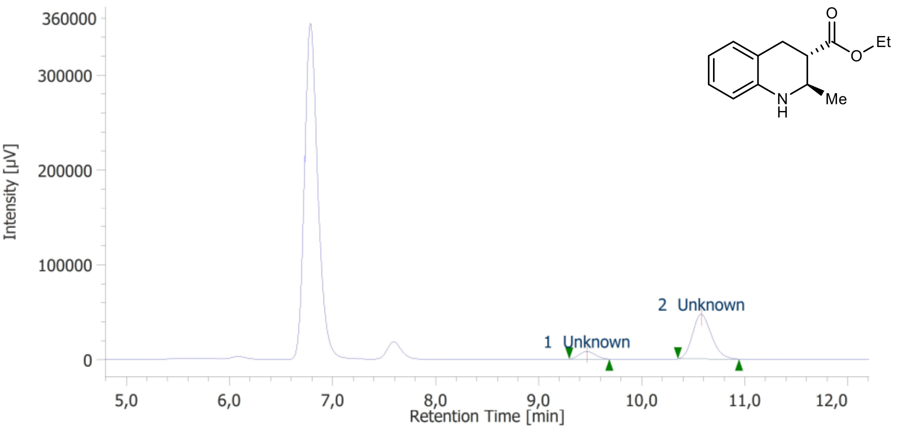

#	Peak Name	CH	tR [min]	Area [µV·sec]	Height [µV]	Area%	Height%	Quantity	NTP	Resolution	Symmetry Factor	Warning
1	Unknown	11	9,467	87026	8124	12,679	14,681	N/A	16995	3,574	1,113	
2	Unknown	11	10,577	599340	47214	87,321	85,319	N/A	16207	N/A	1,181	

rac- and (2*R*,3*S*) 3-*iso*-Propylcarboxylate 2-methyl-1,2,3,4-tetrahydroquinoline **SI-11b**:

IB-column: Hex/ *i*PrOH 95:5 v/v, 1.0 ml/ min, λ = 248 nm

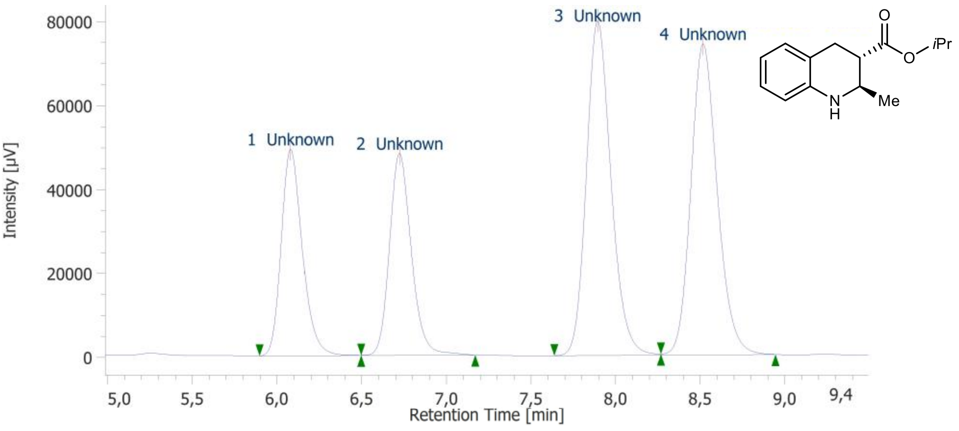

#	Peak Name	CH	tR [min]	Area [µV·sec]	Height [µV]	Area%	Height%	Quantity	NTP	Resolution	Symmetry Factor	Warning
1	Unknown	11	6,080	425529	49327	17,238	19,635	N/A	12072	2,867	1,249	
2	Unknown	11	6,723	430876	48167	17,455	19,173	N/A	13830	4,783	1,175	
3	Unknown	11	7,893	808626	79657	32,758	31,708	N/A	14539	2,306	1,185	
4	Unknown	11	8,517	803472	74067	32,549	29,483	N/A	14787	N/A	1,166	

Batch process:

major Diastereomer:

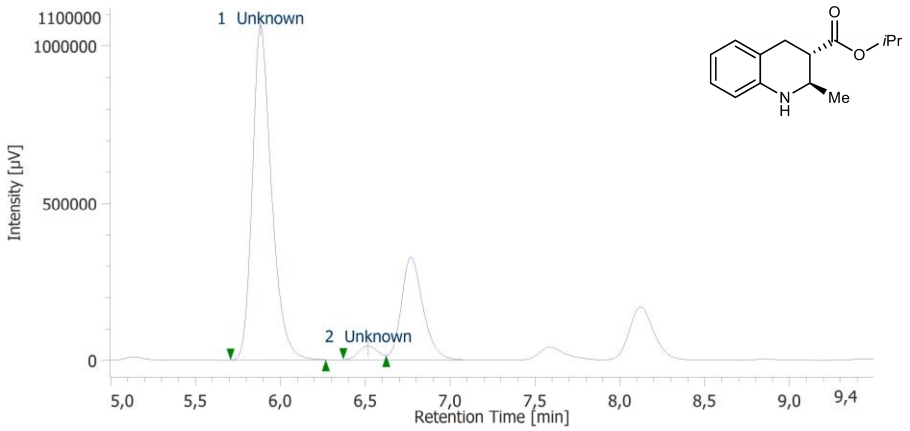

#	Peak Name	CH	tR [min]	Area [µV·sec]	Height [µV]	Area%	Height%	Quantity	NTP	Resolution	Symmetry Factor	Warning
1	Unknown	11	5,883	8365741	1065738	96,026	96,030	N/A	14080	3,069	1,300	
2	Unknown	11	6,517	346225	44061	3,974	3,970	N/A	14627	N/A	N/A	

minor Diastereomer:

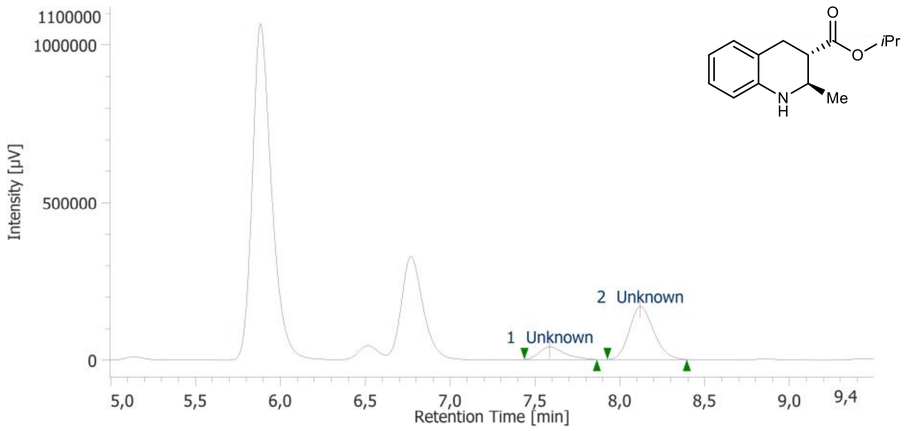

#	Peak Name	CH	tR [min]	Area [µV·sec]	Height [µV]	Area%	Height%	Quantity	NTP	Resolution	Symmetry Factor	Warning
1	Unknown	11	7,587	411570	40161	19,732	19,263	N/A	13762	2,060	1,397	
2	Unknown	11	8,120	1674199	168323	80,268	80,737	N/A	15535	N/A	1,176	

Flow process:

major Diastereomer:

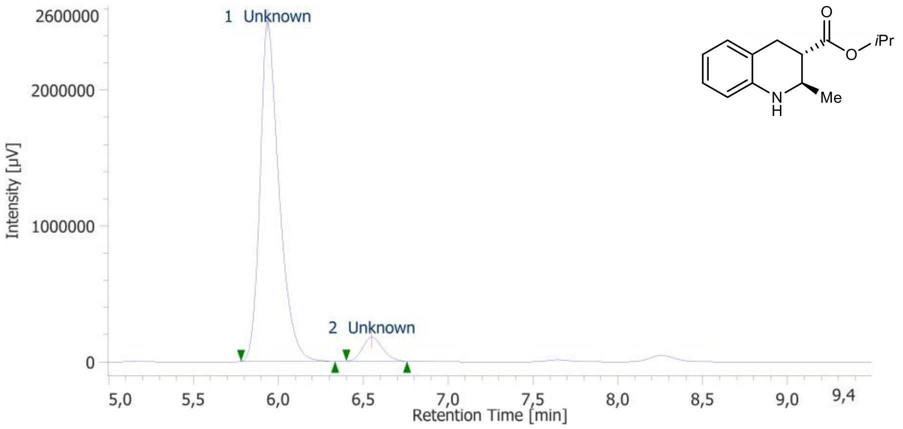

#	Peak Name	CH	tR [min]	Area [µV·sec]	Height [µV]	Area%	Height%	Quantity	NTP	Resolution	Symmetry Factor	Warning
1	Unknown	11	5,937	19532578	2508384	93,097	93,385	N/A	14961	3,005	1,371	
2	Unknown	11	6,550	1448326	177693	6,903	6,615	N/A	14823	N/A	1,159	

minor Diastereomer:

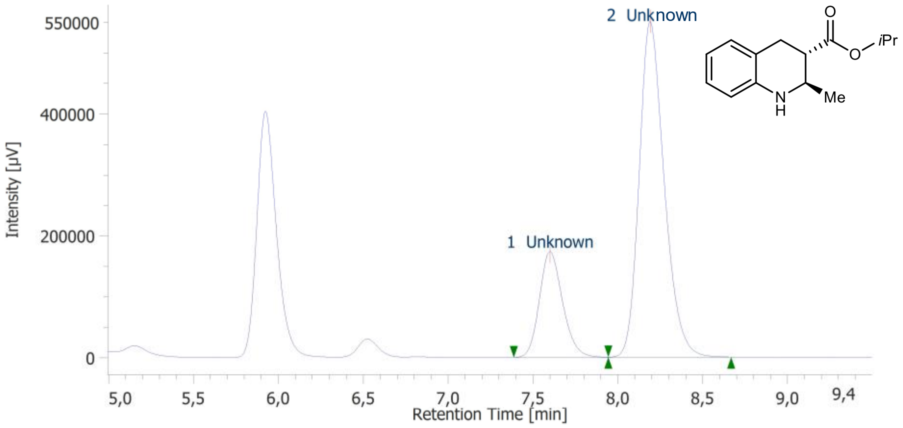

#	Peak Name	CH	tR [min]	Area [µV·sec]	Height [µV]	Area%	Height%	Quantity	NTP	Resolution	Symmetry Factor	Warning
1	Unknown	11	7,600	1648266	173465	22,574	23,961	N/A	15253	2,317	1,178	
2	Unknown	11	8,190	5653312	550474	77,426	76,039	N/A	15353	N/A	1,244	

rac- and (2*R*,3*S*) 3-Methylcarboxylate 2-methyl-1,2,3,4-tetrahydroquinoline **SI-11c**:

IB-column: Hex/ *i*PrOH 98:2 $^{v}/_{v}$, 1.0 ml/ min, λ = 248 nm

major Diastereomer:

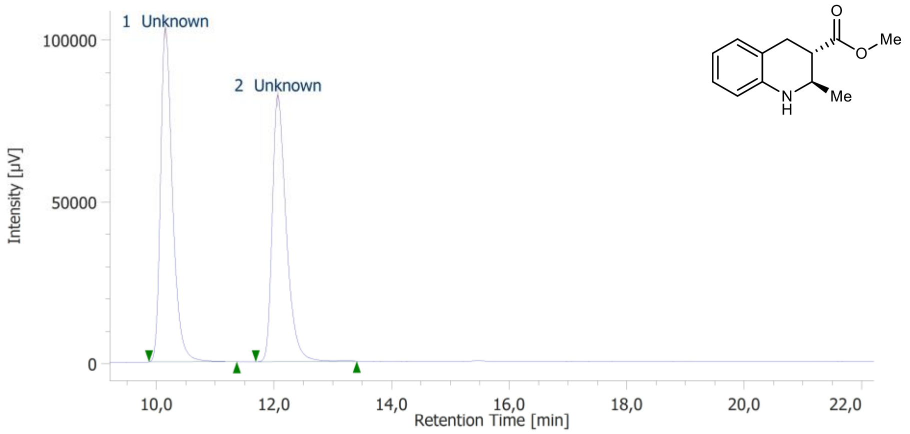

#	Peak Name	CH	tR [min]	Area [µV·sec]	Height [µV]	Area%	Height%	Quantity	NTP	Resolution	Symmetry Factor	Warning
1	Unknown	11	10,153	1457103	103225	51,712	55,631	N/A	13061	4,971	1,402	
2	Unknown	11	12,067	1360626	82329	48,288	44,369	N/A	13430	N/A	1,450	

minor Diastereomer:

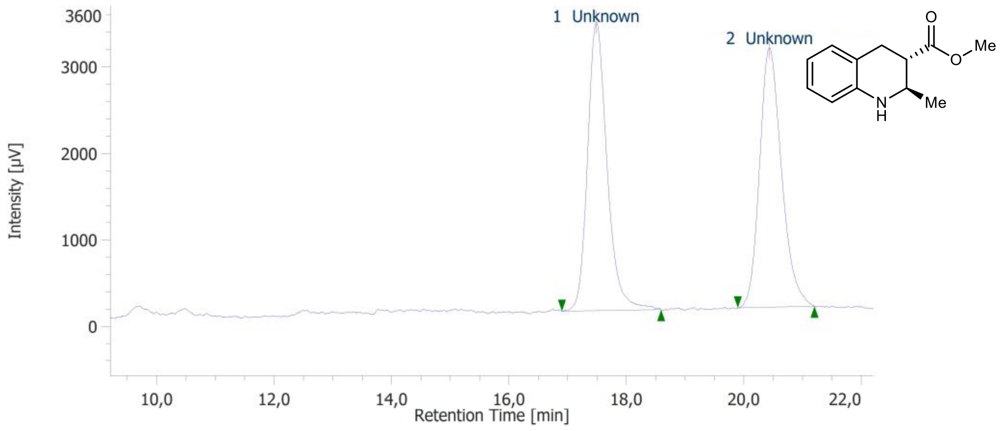

#	Peak Name	CH	tR [min]	Area [µV·sec]	Height [µV]	Area%	Height%	Quantity	NTP	Resolution	Symmetry Factor	Warning
1	Unknown	11	17,480	77060	3325	49,714	52,627	N/A	14486	4,727	1,260	
2	Unknown	11	20,433	77946	2993	50,286	47,373	N/A	14796	N/A	1,242	

Batch process:

major Diastereomer:

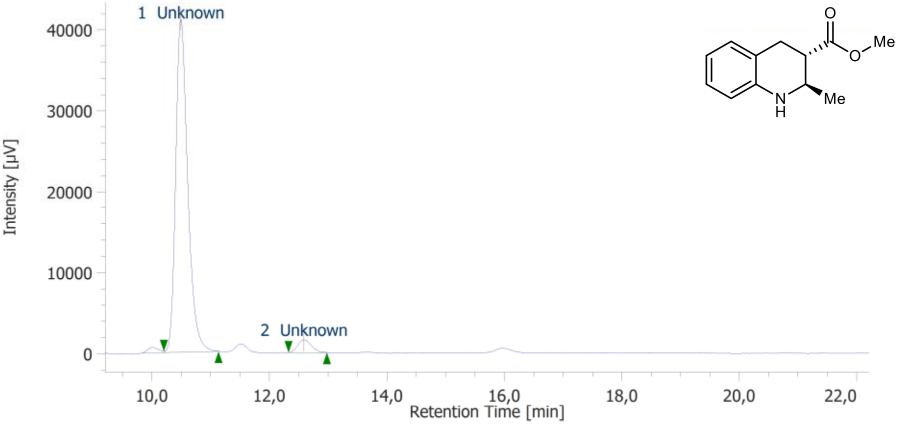

#	Peak Name	CH	tR [min]	Area [µV·sec]	Height [µV]	Area%	Height%	Quantity	NTP	Resolution	Symmetry Factor	Warning
1	Unknown	11	10,493	584795	41125	95,871	96,300	N/A	13466	5,375	1,335	
2	Unknown	11	12,590	25184	1580	4,129	3,700	N/A	14337	N/A	1,152	

minor Diastereomer:

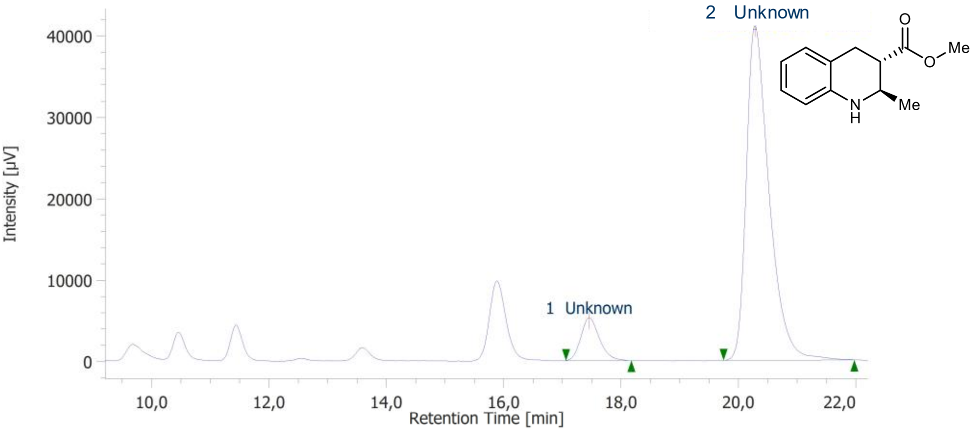

#	Peak Name	CH	tR [min]	Area [µV·sec]	Height [µV]	Area%	Height%	Quantity	NTP	Resolution	Symmetry Factor	Warning
1	Unknown	11	17,450	115785	5290	9,424	11,403	N/A	15308	4,551	1,233	
2	Unknown	11	20,277	1112767	41106	90,576	88,597	N/A	14172	N/A	1,524	

Flow process:

major Diastereomer:

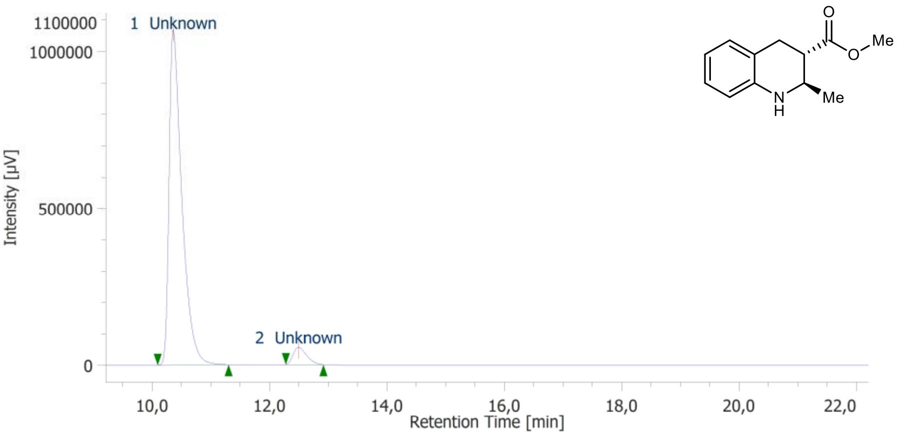

#	Peak Name	CH	tR [min]	Area [μV·sec]	Height [μV]	Area%	Height%	Quantity	NTP	Resolution	Symmetry Factor	Warning
1	Unknown	11	10,360	16171229	1068603	94,910	95,089	N/A	11970	5,399	1,850	
2	Unknown	11	12,497	867222	55194	5,090	4,911	N/A	14516	N/A	1,384	

minor Diastereomer:

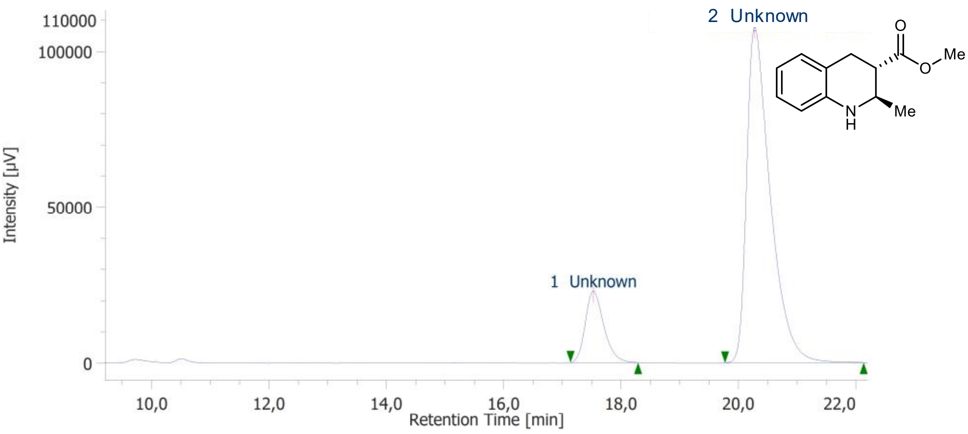

#	Peak Name	CH	tR [min]	Area [μV·sec]	Height [μV]	Area%	Height%	Quantity	NTP	Resolution	Symmetry Factor	Warning
1	Unknown	11	17,523	501391	22884	14,299	17,537	N/A	15376	4,354	1,287	
2	Unknown	11	20,273	3005056	107602	85,701	82,463	N/A	13375	N/A	1,745	

rac- and (2*S*,3*R*) 3-Ethylcarboxylate 2-phenyl-1,2,3,4-tetrahydroquinoline **SI-11d**:

IB-column: Hex/ *i*PrOH 98:2 v/v, 1.0 ml/ min, λ = 300 nm

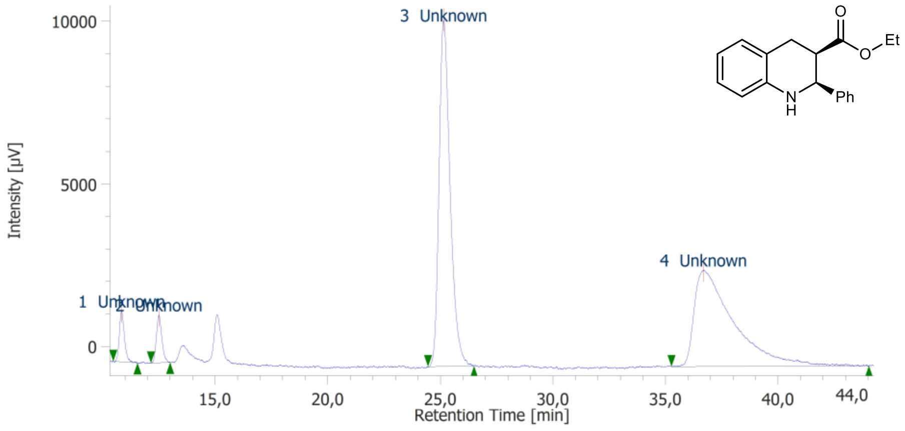

#	Peak Name	CH	tR [min]	Area [µV·sec]	Height [µV]	Area%	Height%	Quantity	NTP	Resolution	Symmetry Factor	Warning
1	Unknown	12	10,853	24569	1558	3,187	9,371	N/A	11758	4,014	1,252	
2	Unknown	12	12,510	24583	1469	3,189	8,837	N/A	13715	19,533	1,243	
3	Unknown	12	25,140	363014	10642	47,095	64,022	N/A	13380	6,183	1,373	
4	Unknown	12	36,677	358643	2954	46,528	17,770	N/A	2609	N/A	3,201	

Batch process:

minor Diastereomer:

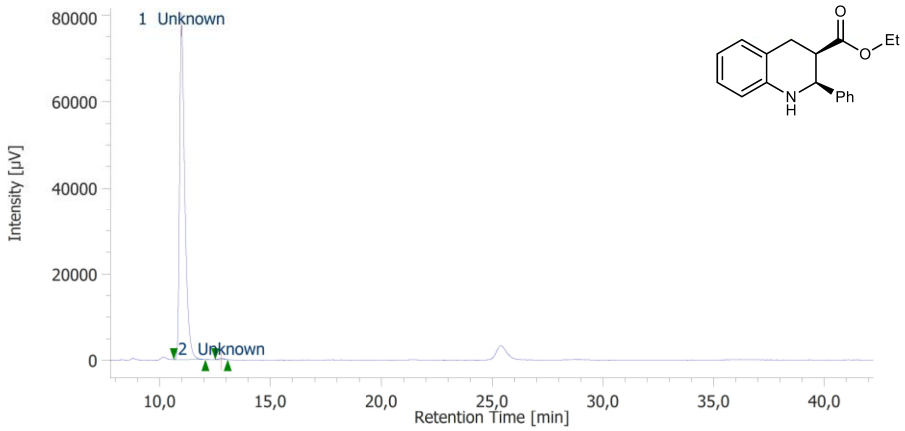

#	Peak Name	CH	tR [min]	Area [µV·sec]	Height [µV]	Area%	Height%	Quantity	NTP	Resolution	Symmetry Factor	Warning
1	Unknown	12	10,983	1293203	77583	99,628	99,651	N/A	11159	4,016	1,664	
2	Unknown	12	12,790	4833	272	0,372	0,349	N/A	11065	N/A	0,997	

major Diastereomer:

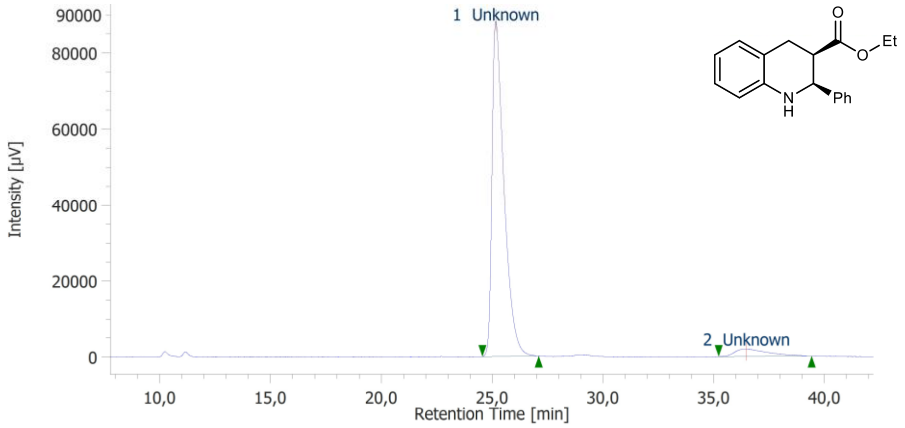

#	Peak Name	CH	tR [min]	Area [µV·sec]	Height [µV]	Area%	Height%	Quantity	NTP	Resolution	Symmetry Factor	Warning
1	Unknown	12	25,157	3306468	88160	94,281	97,875	N/A	11198	6,191	1,897	
2	Unknown	12	36,470	200585	1914	5,719	2,125	N/A	2890	N/A	1,748	

Flow process:

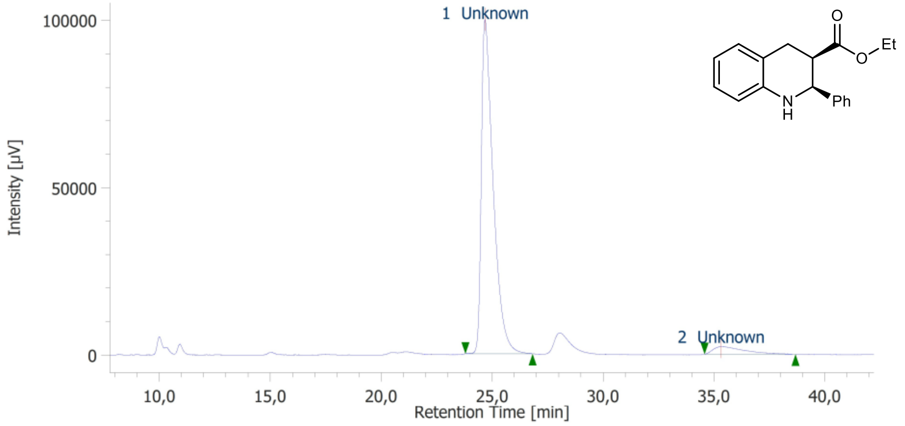

Peak Method (Manual)

#	Peak Name	CH	tR [min]	Area [µV·sec]	Height [µV]	Area%	Height%	Quantity	NTP	Resolution	Symmetry Factor	Warning
1	Unknown	12	24,670	3699877	99792	94,297	97,768	N/A	11258	6,193	1,958	
2	Unknown	12	35,317	223752	2278	5,703	2,232	N/A	3148	N/A	2,553	

rac- and (2*R*,3*S*) 6-Bromo 3-ethylcarboxylate 2-methyl-1,2,3,4-tetrahydroquinoline **SI-11e**:

IB-column: Hex/ *i*PrOH 95:5 $^{v}/_{v}$, 1.0 ml/ min, λ = 256 nm

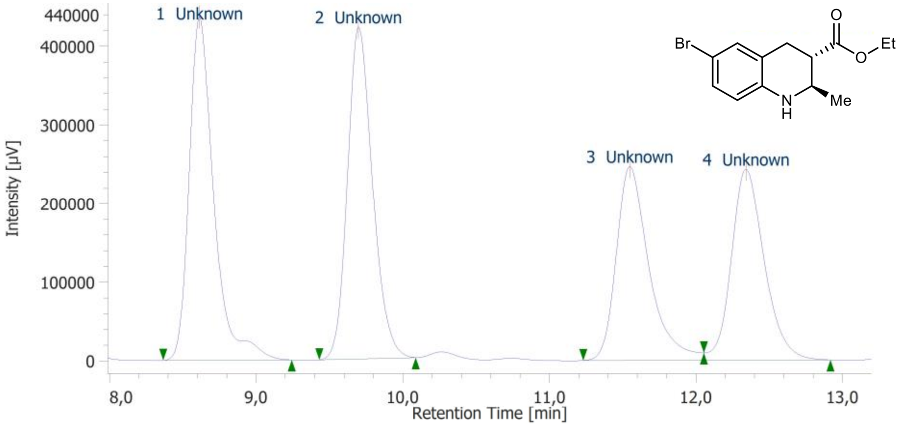

#	Peak Name	CH	tR [min]	Area [µV·sec]	Height [µV]	Area%	Height%	Quantity	NTP	Resolution	Symmetry Factor	Warning
1	Unknown	11	8,613	5093682	436053	28,752	32,370	N/A	14572	3,658	1,666	
2	Unknown	11	9,697	5025505	421594	28,367	31,297	N/A	15815	5,377	1,216	
3	Unknown	11	11,547	3822864	246622	21,579	18,308	N/A	14658	2,045	1,505	
4	Unknown	11	12,340	3773985	242806	21,303	18,025	N/A	15506	N/A	1,176	

Batch process:

major Diastereomer:

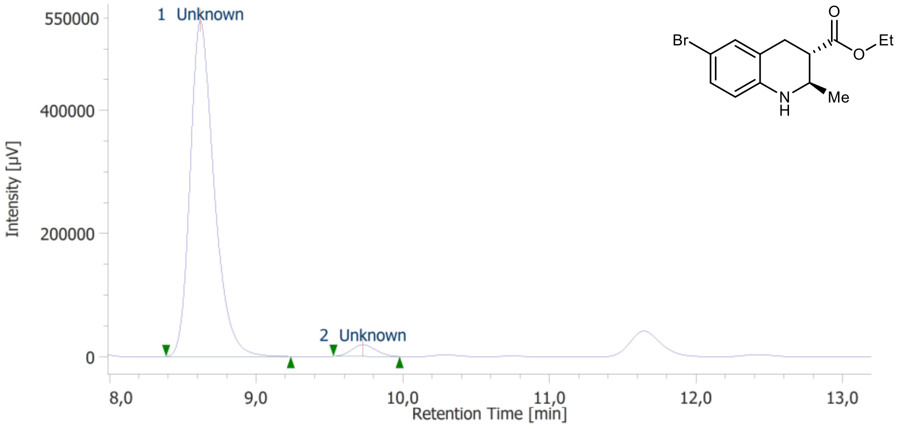

#	Peak Name	CH	tR [min]	Area [µV·sec]	Height [µV]	Area%	Height%	Quantity	NTP	Resolution	Symmetry Factor	Warning
1	Unknown	11	8,620	6170423	544625	96,627	96,645	N/A	14330	3,747	1,311	
2	Unknown	11	9,727	215400	18906	3,373	3,355	N/A	16358	N/A	1,112	

minor Diastereomer:

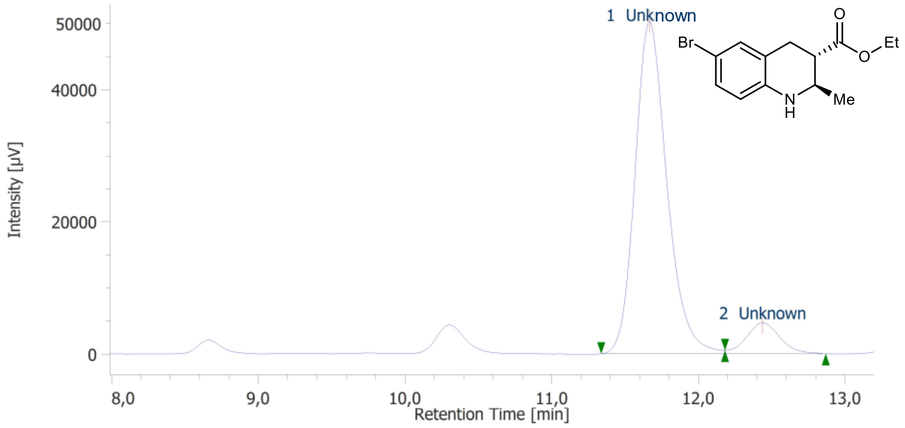

#	Peak Name	CH	tR [min]	Area [µV·sec]	Height [µV]	Area%	Height%	Quantity	NTP	Resolution	Symmetry Factor	Warning
1	Unknown	11	11,667	759216	50225	91,014	91,447	N/A	14628	1,945	1,212	
2	Unknown	11	12,437	74960	4697	8,986	8,553	N/A	14870	N/A	N/A	

Flow process:

major Diastereomer:

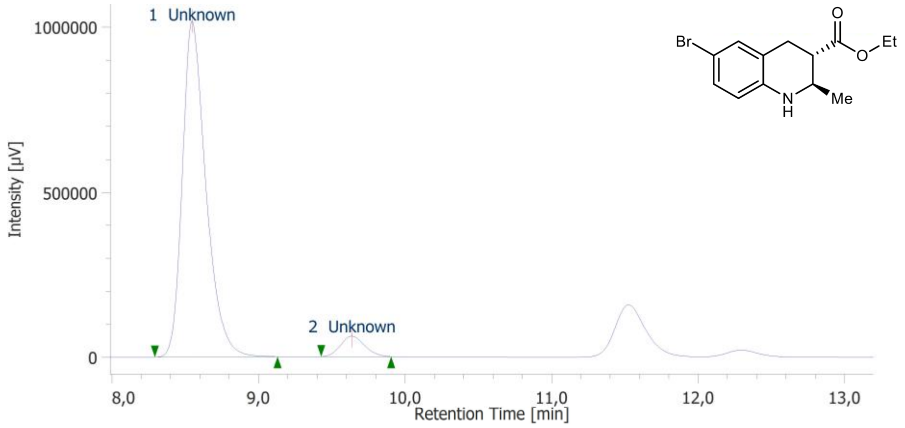

#	Peak Name	CH	tR [min]	Area [µV·sec]	Height [µV]	Area%	Height%	Quantity	NTP	Resolution	Symmetry Factor	Warning
1	Unknown	11	8,547	11369483	1016467	94,168	94,278	N/A	14544	3,731	1,360	
2	Unknown	11	9,637	704160	61692	5,832	5,722	N/A	16256	N/A	1,117	

minor Diastereomer:

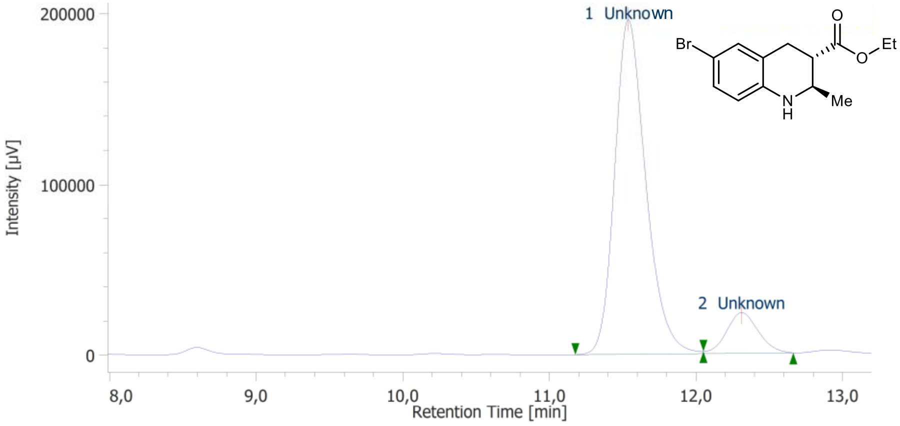

#	Peak Name	CH	tR [min]	Area [µV·sec]	Height [µV]	Area%	Height%	Quantity	NTP	Resolution	Symmetry Factor	Warning
1	Unknown	11	11,537	2891924	195887	89,050	89,136	N/A	14876	2,020	1,264	
2	Unknown	11	12,310	355603	23875	10,950	10,864	N/A	15992	N/A	1,016	

(*E*)-2-((*S*)-2-((*R*)-1-((4-Methoxyphenyl)amino)propyl) cyclopentylidene) ethylacetate **SI-15a**:

IE-column: Hex/ *i*PrOH 95:5 $^{v}/_{v}$, 0.5 ml/ min, λ = 300 nm

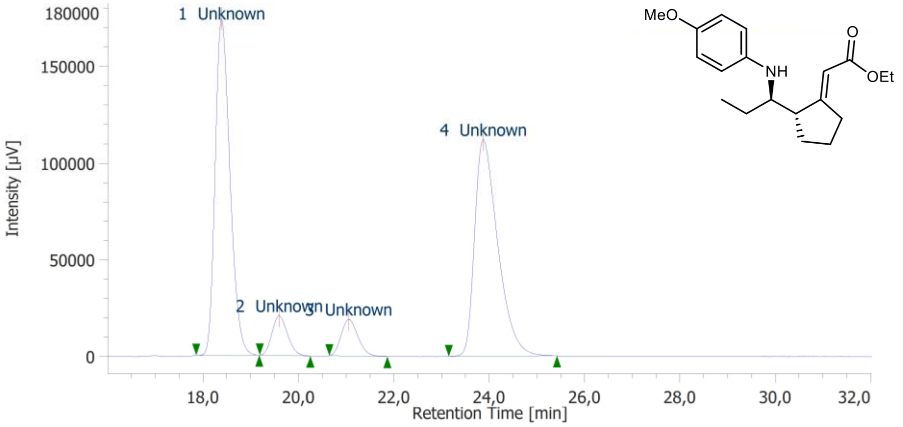

#	Peak Name	CH	tR [min]	Area [µV·sec]	Height [µV]	Area%	Height%	Quantity	NTP	Resolution	Symmetry Factor	Warning
1	Unknown	12	18,390	3751659	173777	44,402	53,411	N/A	16820	2,083	1,279	
2	Unknown	12	19,597	460671	20510	5,452	6,304	N/A	17393	2,358	1,180	
3	Unknown	12	21,050	462224	19052	5,471	5,856	N/A	17231	3,747	1,193	
4	Unknown	12	23,873	3774687	112021	44,675	34,430	N/A	12064	N/A	1,558	

Batch process:

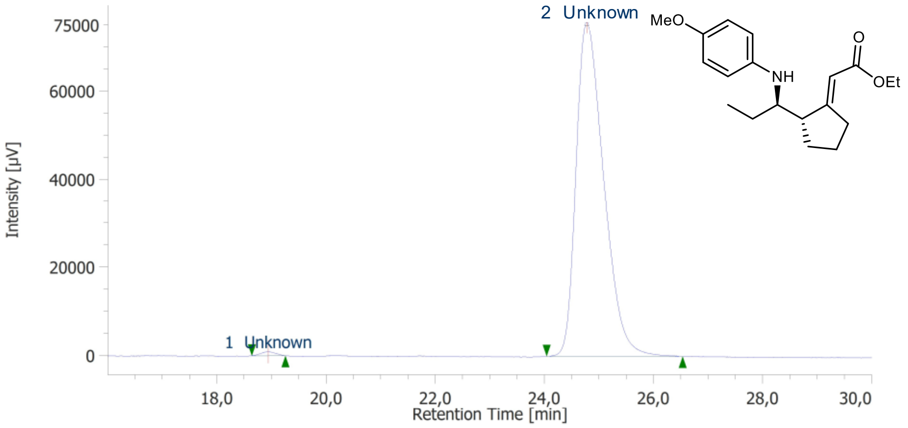

#	Peak Name	CH	tR [min]	Area [µV·sec]	Height [µV]	Area%	Height%	Quantity	NTP	Resolution	Symmetry Factor	Warning
1	Unknown	12	18,940	17106	885	0,637	1,153	N/A	19202	7,973	1,042	
2	Unknown	12	24,773	2666404	75842	99,363	98,847	N/A	11589	N/A	1,436	

Flow process:

2-Me-THF:

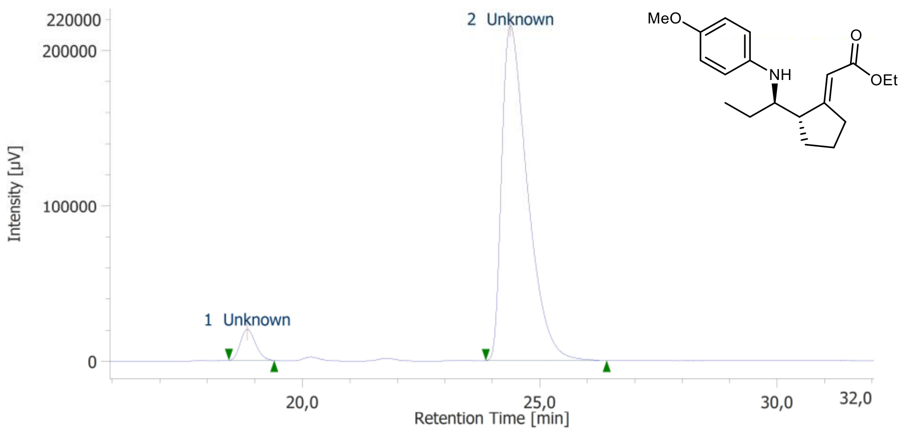

#	Peak Name	CH	tR [min]	Area [µV·sec]	Height [µV]	Area%	Height%	Quantity	NTP	Resolution	Symmetry Factor	Warning
1	Unknown	12	18,840	446078	20242	5,216	8,585	N/A	16734	7,132	1,195	
2	Unknown	12	24,380	8105503	215539	94,784	91,415	N/A	10002	N/A	1,811	

THF:

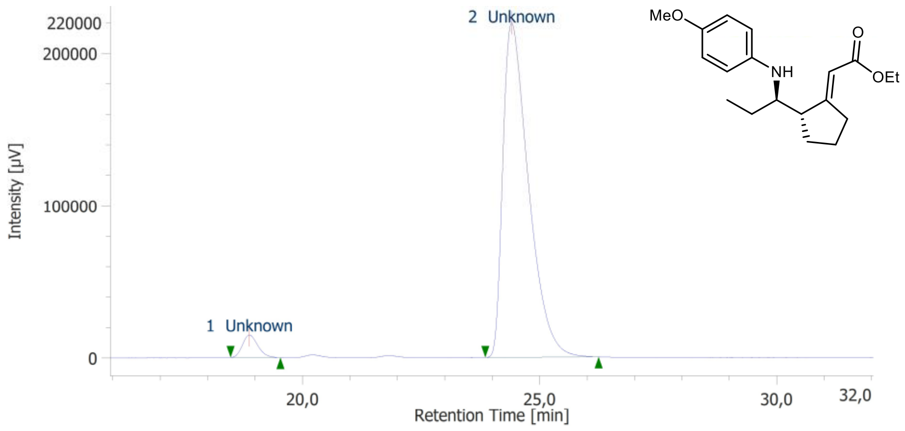

#	Peak Name	CH	tR [min]	Area [µV·sec]	Height [µV]	Area%	Height%	Quantity	NTP	Resolution	Symmetry Factor	Warning
1	Unknown	12	18,880	331353	14786	3,861	6,317	N/A	16457	7,075	1,210	
2	Unknown	12	24,407	8250547	219270	96,139	93,683	N/A	9970	N/A	1,785	

(*E*)-2-((*S*)-2-((*R*)-1-((4-Methoxyphenyl)amino)-2-phenylethyl) cyclopentylidene) ethylacetate **SI-15b**:

IE-column: Hex/ *i*PrOH 95:5 $^{v}/_{v}$, 0.5 ml/ min, λ = 250 nm

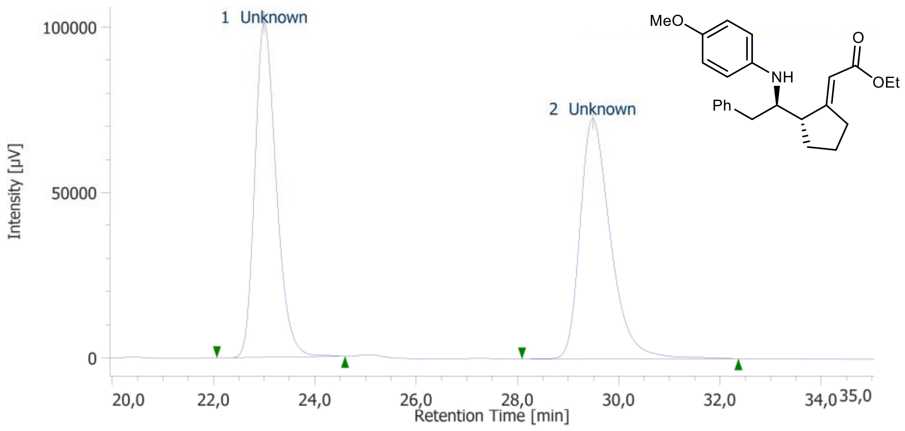

#	Peak Name	CH	tR [min]	Area [µV·sec]	Height [µV]	Area%	Height%	Quantity	NTP	Resolution	Symmetry Factor	Warning
1	Unknown	11	23,000	2968473	101217	49,461	58,226	N/A	14723	7,163	1,267	
2	Unknown	11	29,480	3033137	72617	50,539	41,774	N/A	12473	N/A	1,387	

Batch process:

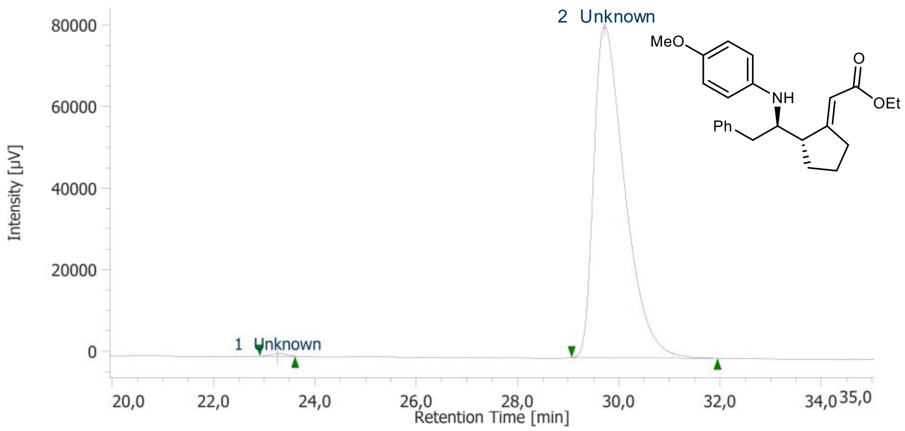

#	Peak Name	CH	tR [min]	Area [µV·sec]	Height [µV]	Area%	Height%	Quantity	NTP	Resolution	Symmetry Factor	Warning
1	Unknown	11	23,267	13848	641	0,400	0,779	N/A	21828	7,529	1,022	
2	Unknown	11	29,720	3452039	81624	99,600	99,221	N/A	11919	N/A	1,705	

Flow process:

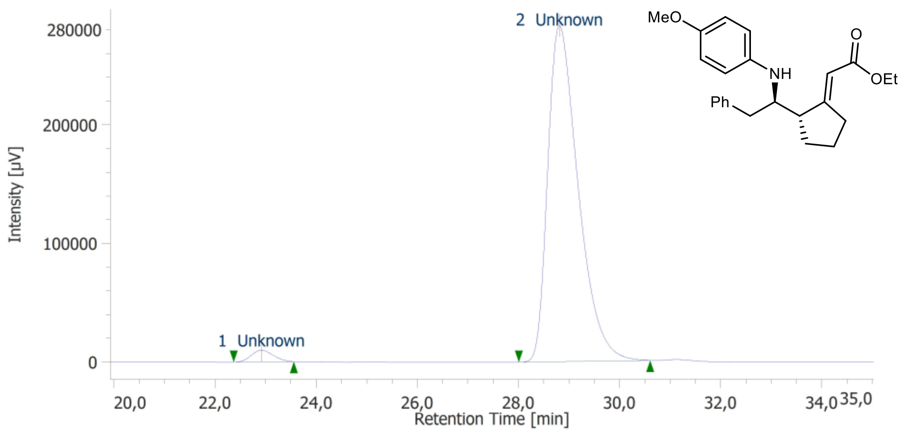

#	Peak Name	CH	tR [min]	Area [µV·sec]	Height [µV]	Area%	Height%	Quantity	NTP	Resolution	Symmetry Factor	Warning
1	Unknown	11	22,917	298530	9878	2,459	3,372	N/A	12807	6,236	1,138	
2	Unknown	11	28,807	11843752	283056	97,541	96,628	N/A	11299	N/A	1,538	

(*E*)-2-((*S*)-2-((*R*)-1-((4-Methoxyphenyl)amino)pent-4-yn-1-yl) cyclopentylidene) ethylacetate **SI-15c**:

IB -column: Hex/ *i*PrOH 95:5 $^{v}/_{v}$, 0.5 ml/ min, λ = 250 nm

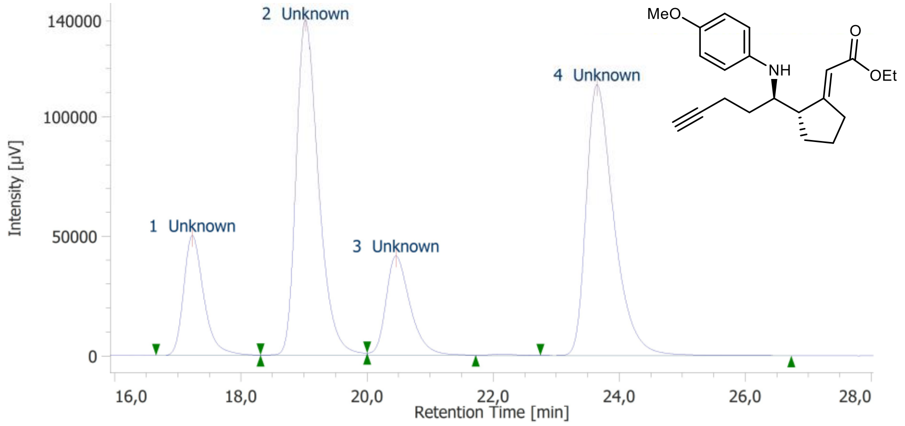

#	Peak Name	CH	tR [min]	Area [µV·sec]	Height [µV]	Area%	Height%	Quantity	NTP	Resolution	Symmetry Factor	Warning
1	Unknown	11	17,230	1117440	50127	12,205	14,528	N/A	15131	3,023	1,358	
2	Unknown	11	19,023	3454373	140078	37,728	40,598	N/A	14626	2,201	1,369	
3	Unknown	11	20,457	1120144	41635	12,234	12,067	N/A	14611	4,404	1,358	
4	Unknown	11	23,643	3463951	113197	37,833	32,807	N/A	14921	N/A	1,506	

Batch process:

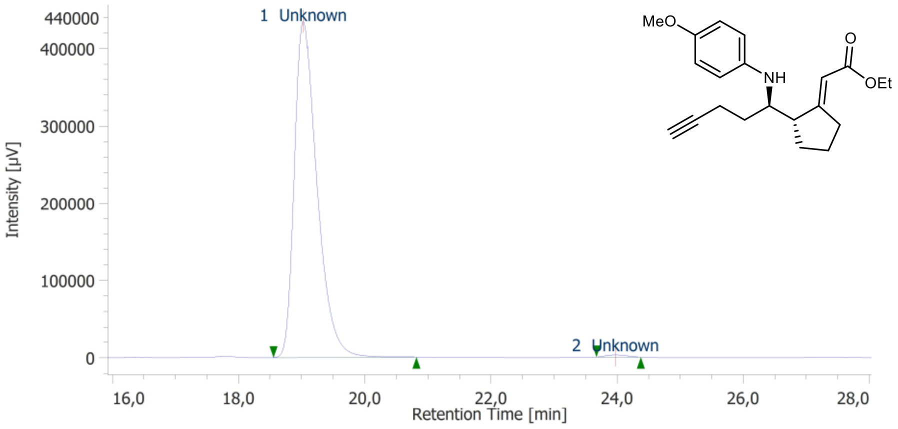

#	Peak Name	CH	tR [min]	Area [µV·sec]	Height [µV]	Area%	Height%	Quantity	NTP	Resolution	Symmetry Factor	Warning
1	Unknown	11	19,023	10777959	434469	99,400	99,357	N/A	14365	7,684	1,483	
2	Unknown	11	23,970	65086	2810	0,600	0,643	N/A	21355	N/A	1,159	

Flow process:

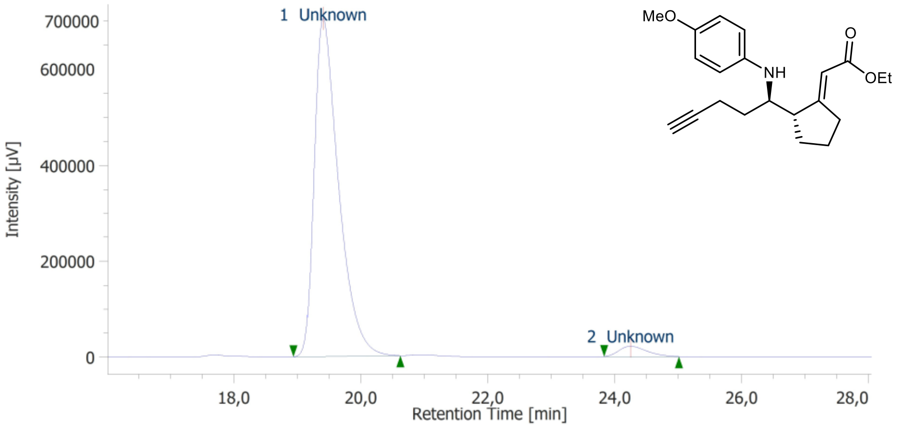

#	Peak Name	CH	tR [min]	Area [µV·sec]	Height [µV]	Area%	Height%	Quantity	NTP	Resolution	Symmetry Factor	Warning
1	Unknown	11	19,403	18694587	702966	96,647	97,043	N/A	13170	6,560	1,621	
2	Unknown	11	24,243	648634	21422	3,353	2,957	N/A	14572	N/A	1,363	

(*R*)-4-((*S*,*E*)-2-(2-Ethoxy-2-oxoethylidene)cyclopentyl)-4-((4-methoxyphenyl)amino) ethylbutanoate **SI-15d**:

IB -column: Hex/ *i*PrOH 95:5 v/v, 0.5 ml/ min, λ = 250 nm

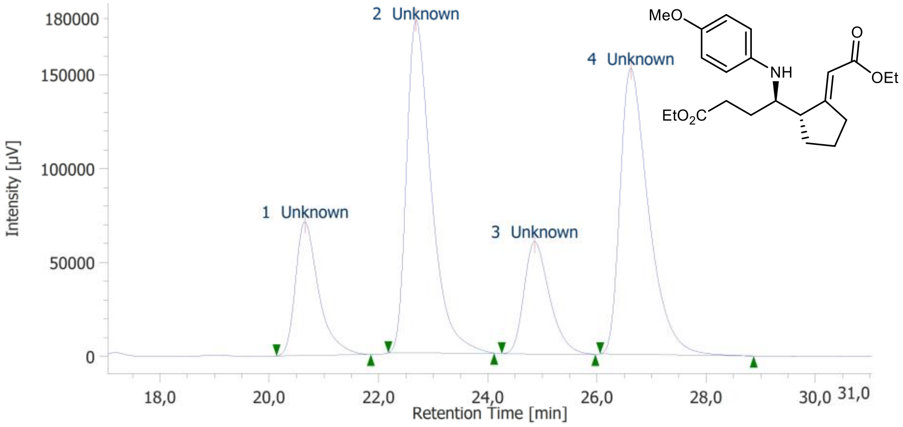

#	Peak Name	CH	tR [min]	Area [µV·sec]	Height [µV]	Area%	Height%	Quantity	NTP	Resolution	Symmetry Factor	Warning
1	Unknown	13	20,650	2077311	71095	13,735	15,452	N/A	13097	2,690	1,529	
2	Unknown	13	22,683	5577270	177173	36,876	38,508	N/A	13062	2,670	1,575	
3	Unknown	13	24,857	1938421	59693	12,817	12,974	N/A	14061	2,005	1,352	
4	Unknown	13	26,617	5531212	152138	36,572	33,066	N/A	13331	N/A	1,600	

Batch process:

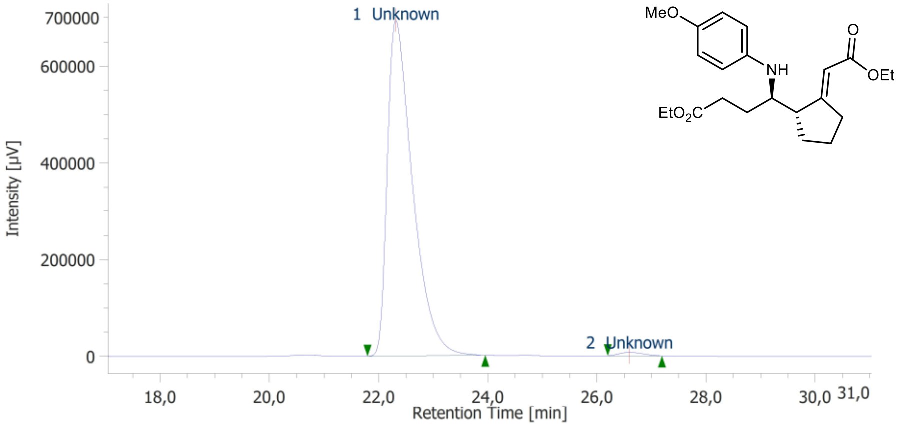

#	Peak Name	CH	tR [min]	Area [µV·sec]	Height [µV]	Area%	Height%	Quantity	NTP	Resolution	Symmetry Factor	Warning
1	Unknown	11	22,313	22319943	693292	99,037	98,974	N/A	11773	5,185	1,811	
2	Unknown	11	26,597	216925	7190	0,963	1,026	N/A	16274	N/A	1,217	

Flow process:

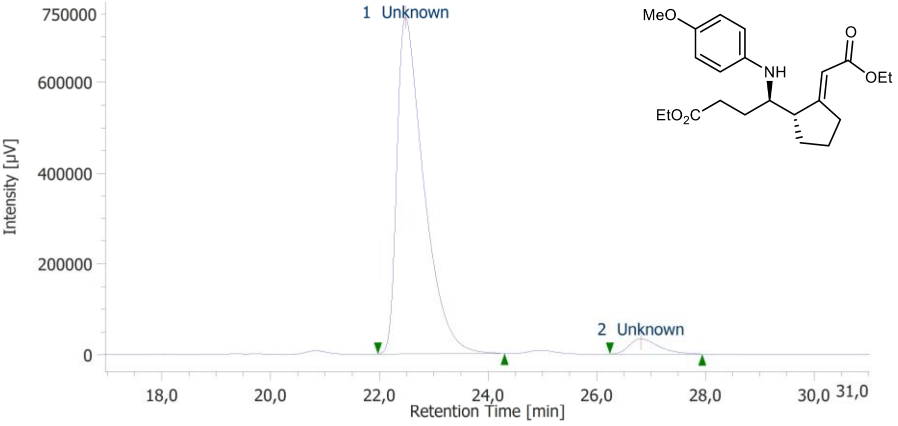

#	Peak Name	CH	tR [min]	Area [µV·sec]	Height [µV]	Area%	Height%	Quantity	NTP	Resolution	Symmetry Factor	Warning
1	Unknown	11	22,480	25415570	740693	95,298	95,649	N/A	10670	4,752	1,912	
2	Unknown	11	26,810	1253870	33692	4,702	4,351	N/A	12565	N/A	1,462	

(*E*)-2-((*S*)-2-((*R*)-1-((4-Methoxyphenyl)amino)-3,3-dimethylbutyl) cyclopentylidene) ethylacetate **SI-15e**:

IE -column: Hex/ *i*PrOH 98:2 $^{v}/_{v}$, 0.5 ml/ min, λ = 250 nm

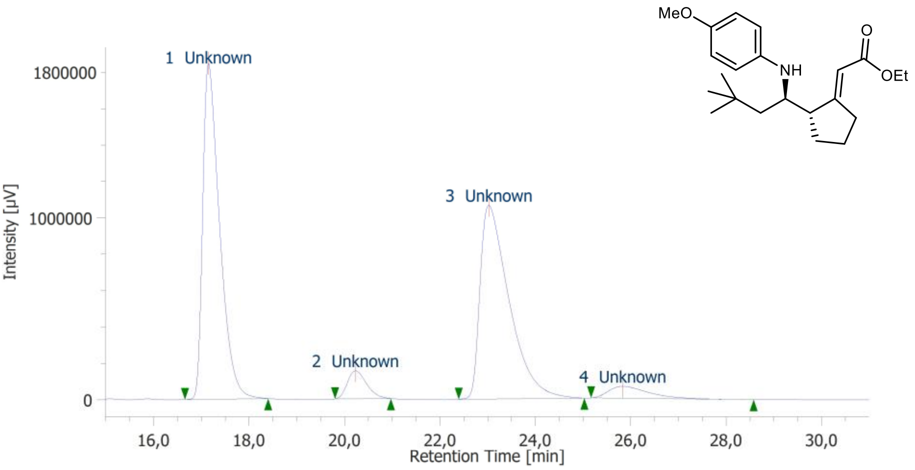

#	Peak Name	CH	tR [min]	Area [µV·sec]	Height [µV]	Area%	Height%	Quantity	NTP	Resolution	Symmetry Factor	Warning
1	Unknown	11	17,150	45530803	1844238	46,159	58,965	N/A	11678	4,502	1,734	
2	Unknown	11	20,227	4293148	154104	4,352	4,927	N/A	12098	3,091	1,312	
3	Unknown	11	23,023	45001568	1063899	45,623	34,016	N/A	7285	2,176	2,097	
4	Unknown	11	25,830	3813241	65416	3,866	2,092	N/A	4699	N/A	1,727	

Batch process:

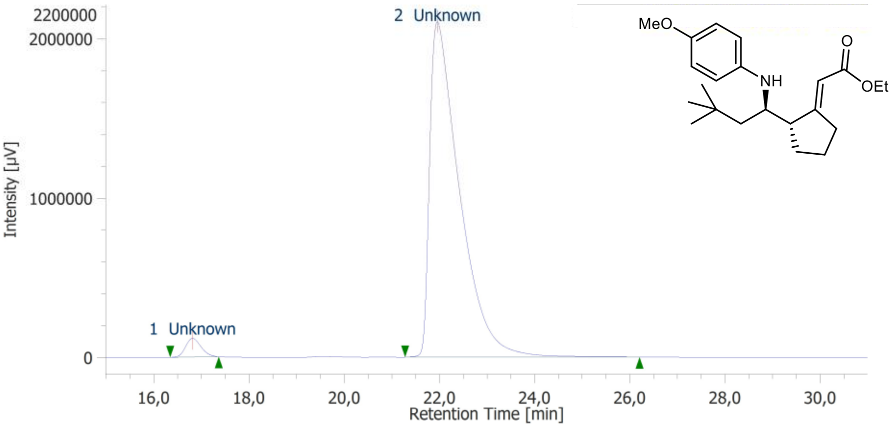

#	Peak Name	CH	tR [min]	Area [µV·sec]	Height [µV]	Area%	Height%	Quantity	NTP	Resolution	Symmetry Factor	Warning
1	Unknown	11	16,813	2615121	117014	2,776	5,268	N/A	12985	6,118	1,198	
2	Unknown	11	21,953	91592204	2104404	97,224	94,732	N/A	6437	N/A	2,571	

Flow process:

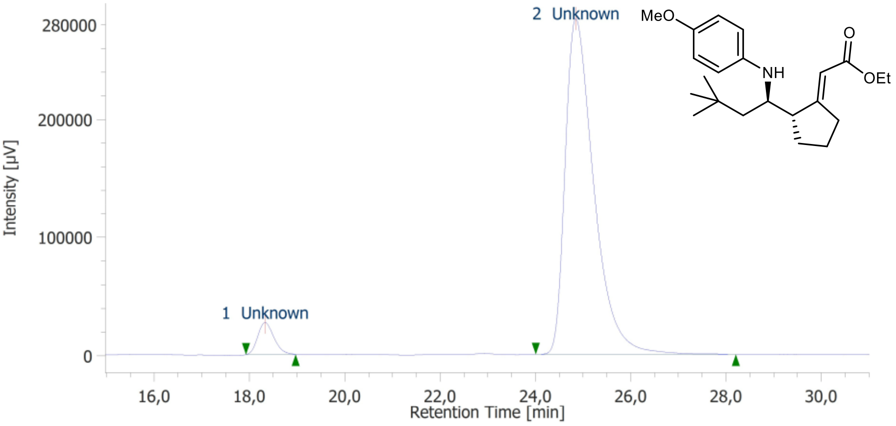

#	Peak Name	CH	tR [min]	Area [µV·sec]	Height [µV]	Area%	Height%	Quantity	NTP	Resolution	Symmetry Factor	Warning
1	Unknown	11	18,327	636071	27190	5,066	8,739	N/A	14183	7,801	1,209	
2	Unknown	11	24,847	11920308	283954	94,934	91,261	N/A	8782	N/A	1,623	

5. Crystallographic Data

Single crystals (98% *ee*) of **SI-11e** were obtained by crystallization of hot *i*PrOH as solvent. Data was then collected on a Gemini diffractometer (Rigaku Oxford Diffraction) using Mo-K α radiation (λ = 0.71073 Å) and ω scan rotation. The structure was solved with SHELXT-2018 (dual-space method). Anisotropic refinement of all non-hydrogen atoms was done using SHELXL-2018. All Hydrogen atoms were located on difference Fourier maps calculated at the final stage of the structure refinement. Structure solution without any problems.

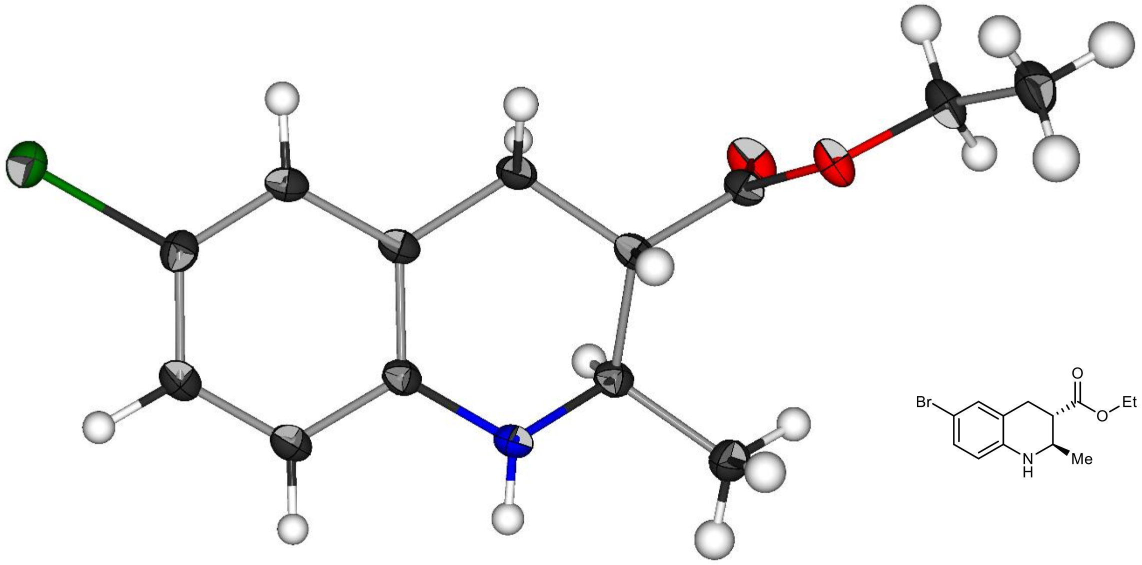

Supplementary figure SF31: X-Ray crystal structure of **SI-11e**. Thermal ellipsoids are drawn at 50% probability.

Supplementary table S10: Crystal data and structure refinement for **SI-11e**.

Identification code	**SI-11e**	
Empirical formula	C13 H16 Br N O2	
Formula weight	298.18	
Temperature	130(2) K	
Wavelength	71.073 pm	
Crystal system	Orthorhombic	
Space group	P 21 21 21	
Unit cell dimensions	a = 695.780(10) pm	α= 90°.
	b = 908.900(10) pm	β= 90°.
	c = 2014.84(2) pm	γ = 90°.
Volume	1.27417(3) nm^3	
Z	4	
Density (calculated)	1.554 Mg/m^3	
Absorption coefficient	3.216 mm^{-1}	
F(000)	608	
Crystal size	0.40 x 0.30 x 0.27 mm^3	
Theta range for data collection	2.458 to 32.386°.	
Index ranges	-10<=h<=10, -13<=k<=13, -29<=l<=30	
Reflections collected	31099	
Independent reflections	4353 [R(int) = 0.0301]	
Completeness to theta = 30.510°	100.0 %	
Absorption correction	Semi-empirical from equivalents	
Max. and min. transmission	1.00000 and 0.89817	
Refinement method	Full-matrix least-squares on F^2	
Data / restraints / parameters	4353 / 0 / 218	
Goodness-of-fit on F^2	1.058	
Final R indices [I>2sigma(I)]	R1 = 0.0222, wR2 = 0.0469	
R indices (all data)	R1 = 0.0270, wR2 = 0.0491	
Absolute structure parameter	-0.014(3)	
Largest diff. peak and hole	0.262 and -0.487 e.Å^{-3}	

Comments: Structure solution with SHELXT-2018 (dual-space method).[11] Anisotropic refinement of all non-hydrogen atoms with SHELXL-2018. All H atoms were located on difference Fourier maps calculated at the final stage of the structure refinement. Structure solution without any problems.

Supplementary table S11: Atomic coordinates (x 10^4) and equivalent isotropic displacement parameters (pm^2x 10^{-1}) for **SI-11e**. U(eq) is defined as one third of the trace of the orthogonalized U^{ij} tensor.

	x	y	z	U(eq)
Br(1)	1152(1)	5645(1)	9251(1)	27(1)
O(1)	9038(2)	4429(2)	6258(1)	25(1)
O(2)	7993(2)	6346(1)	5665(1)	21(1)
N(1)	8414(2)	7615(2)	7848(1)	20(1)
C(1)	9167(2)	6719(2)	7311(1)	18(1)
C(2)	7506(3)	6450(2)	6822(1)	18(1)
C(3)	5916(2)	5580(2)	7167(1)	19(1)
C(4)	5499(3)	6160(2)	7856(1)	17(1)
C(5)	3821(3)	5736(2)	8182(1)	18(1)
C(6)	3429(3)	6261(2)	8811(1)	20(1)
C(7)	4673(3)	7218(2)	9130(1)	21(1)
C(8)	6341(3)	7643(2)	8813(1)	20(1)
C(9)	6776(3)	7128(2)	8172(1)	17(1)
C(10)	10868(3)	7494(2)	6993(1)	22(1)
C(11)	8256(2)	5616(2)	6229(1)	18(1)
C(12)	8726(3)	5640(2)	5069(1)	25(1)
C(13)	8329(4)	6650(3)	4500(1)	30(1)

Supplementary table S12: Bond lengths [pm] and angles [°] for **SI-11e**.

Br(1)-C(6)	190.03(18)
O(1)-C(11)	121.0(2)
O(2)-C(11)	133.0(2)
O(2)-C(12)	145.2(2)
N(1)-C(9)	138.6(2)
N(1)-C(1)	145.1(2)
N(1)-H(1N)	85(3)
C(1)-C(10)	152.0(3)
C(1)-C(2)	153.9(2)
C(1)-H(1)	98(3)
C(2)-C(11)	150.7(3)
C(2)-C(3)	152.7(3)

C(2)-H(2)	100(3)
C(3)-C(4)	151.2(2)
C(3)-H(3A)	95(3)
C(3)-H(3B)	98(2)
C(4)-C(5)	139.4(3)
C(4)-C(9)	140.4(3)
C(5)-C(6)	138.2(2)
C(5)-H(5)	92(3)
C(6)-C(7)	138.5(3)
C(7)-C(8)	138.1(3)
C(7)-H(7)	103(2)
C(8)-C(9)	140.5(2)
C(8)-H(8)	92(2)
C(10)-H(10A)	97(3)
C(10)-H(10B)	96(3)
C(10)-H(10C)	93(3)
C(12)-C(13)	149.5(3)
C(12)-H(12A)	97(3)
C(12)-H(12B)	96(3)
C(13)-H(13A)	95(3)
C(13)-H(13B)	92(3)
C(13)-H(13C)	99(3)

C(11)-O(2)-C(12)	115.98(14)
C(9)-N(1)-C(1)	117.97(15)
C(9)-N(1)-H(1N)	116.3(16)
C(1)-N(1)-H(1N)	113.7(17)
N(1)-C(1)-C(10)	109.58(15)
N(1)-C(1)-C(2)	107.22(14)
C(10)-C(1)-C(2)	112.84(15)
N(1)-C(1)-H(1)	110.7(15)
C(10)-C(1)-H(1)	110.1(15)
C(2)-C(1)-H(1)	106.3(15)
C(11)-C(2)-C(3)	110.56(15)
C(11)-C(2)-C(1)	109.14(15)
C(3)-C(2)-C(1)	109.51(14)
C(11)-C(2)-H(2)	108.0(16)
C(3)-C(2)-H(2)	111.0(16)
C(1)-C(2)-H(2)	108.6(16)

C(4)-C(3)-C(2)	112.13(15)
C(4)-C(3)-H(3A)	109.2(15)
C(2)-C(3)-H(3A)	109.3(16)
C(4)-C(3)-H(3B)	112.2(13)
C(2)-C(3)-H(3B)	105.5(13)
H(3A)-C(3)-H(3B)	108(2)
C(5)-C(4)-C(9)	119.26(16)
C(5)-C(4)-C(3)	119.81(16)
C(9)-C(4)-C(3)	120.92(16)
C(6)-C(5)-C(4)	120.15(17)
C(6)-C(5)-H(5)	118.5(16)
C(4)-C(5)-H(5)	121.3(16)
C(5)-C(6)-C(7)	121.30(17)
C(5)-C(6)-Br(1)	119.39(14)
C(7)-C(6)-Br(1)	119.30(14)
C(8)-C(7)-C(6)	119.07(17)
C(8)-C(7)-H(7)	121.6(14)
C(6)-C(7)-H(7)	119.3(14)
C(7)-C(8)-C(9)	120.88(17)
C(7)-C(8)-H(8)	120.2(15)
C(9)-C(8)-H(8)	118.9(15)
N(1)-C(9)-C(4)	120.39(16)
N(1)-C(9)-C(8)	120.25(17)
C(4)-C(9)-C(8)	119.32(17)
C(1)-C(10)-H(10A)	113.8(17)
C(1)-C(10)-H(10B)	109.9(15)
H(10A)-C(10)-H(10B)	107(2)
C(1)-C(10)-H(10C)	112.3(16)
H(10A)-C(10)-H(10C)	107(2)
H(10B)-C(10)-H(10C)	107(2)
O(1)-C(11)-O(2)	123.21(16)
O(1)-C(11)-C(2)	124.48(16)
O(2)-C(11)-C(2)	112.30(16)
O(2)-C(12)-C(13)	107.32(17)
O(2)-C(12)-H(12A)	108.1(16)
C(13)-C(12)-H(12A)	112.0(17)
O(2)-C(12)-H(12B)	110.2(15)
C(13)-C(12)-H(12B)	112.8(16)
H(12A)-C(12)-H(12B)	106(2)

C(12)-C(13)-H(13A)	110.2(17)
C(12)-C(13)-H(13B)	115.7(19)
H(13A)-C(13)-H(13B)	103(3)
C(12)-C(13)-H(13C)	109.6(18)
H(13A)-C(13)-H(13C)	108(2)
H(13B)-C(13)-H(13C)	110(3)

Symmetry transformations used to generate equivalent atoms:

Supplementary table S13: Anisotropic displacement parameters (pm^2x 10^{-1}) for **SI-11e**. The anisotropic displacement factor exponent takes the form: $-2\pi^2[h^2a^{*2}U^{11} + ... + 2 h k a^* b^* U^{12}]$

	U^{11}	U^{22}	U^{33}	U^{23}	U^{13}	U^{12}
Br(1)	25(1)	31(1)	27(1)	-2(1)	6(1)	-4(1)
O(1)	32(1)	20(1)	21(1)	-2(1)	-3(1)	10(1)
O(2)	29(1)	22(1)	14(1)	-1(1)	-1(1)	8(1)
N(1)	22(1)	22(1)	16(1)	-3(1)	-2(1)	-5(1)
C(1)	19(1)	17(1)	16(1)	1(1)	-3(1)	0(1)
C(2)	21(1)	17(1)	15(1)	-1(1)	-4(1)	3(1)
C(3)	18(1)	21(1)	18(1)	-4(1)	-3(1)	-1(1)
C(4)	19(1)	16(1)	17(1)	-1(1)	-4(1)	1(1)
C(5)	18(1)	17(1)	20(1)	-1(1)	-4(1)	1(1)
C(6)	18(1)	21(1)	20(1)	3(1)	1(1)	1(1)
C(7)	25(1)	20(1)	17(1)	-1(1)	-2(1)	2(1)
C(8)	24(1)	18(1)	17(1)	-2(1)	-3(1)	-1(1)
C(9)	19(1)	17(1)	15(1)	1(1)	-3(1)	0(1)
C(10)	23(1)	23(1)	21(1)	2(1)	-1(1)	-3(1)
C(11)	18(1)	19(1)	17(1)	-1(1)	-3(1)	2(1)
C(12)	32(1)	29(1)	16(1)	-4(1)	2(1)	5(1)
C(13)	33(1)	39(1)	18(1)	1(1)	0(1)	3(1)

Supplementary table S14: Hydrogen coordinates (x 10^4) and isotropic displacement parameters (pm^2x 10^{-1}) for **SI-11e**.

	x	y	z	U(eq)
H(1N)	9280(40)	8000(30)	8089(12)	22(6)
H(1)	9560(30)	5750(30)	7476(12)	25(6)
H(2)	7020(40)	7420(30)	6664(13)	31(7)
H(3A)	4780(40)	5630(30)	6909(12)	25(6)
H(3B)	6360(30)	4560(20)	7174(11)	17(5)
H(5)	2950(40)	5110(30)	7987(13)	24(6)
H(7)	4330(30)	7600(30)	9596(12)	22(6)
H(8)	7200(40)	8260(30)	9022(12)	20(6)
H(10A)	10530(40)	8400(30)	6767(14)	35(7)
H(10B)	11460(40)	6860(30)	6670(12)	24(6)
H(10C)	11810(40)	7730(30)	7300(14)	33(7)
H(12A)	8070(40)	4700(30)	5020(14)	33(7)
H(12B)	10070(40)	5420(30)	5120(12)	26(6)
H(13A)	6990(50)	6820(30)	4460(14)	37(8)
H(13B)	8820(50)	7580(40)	4538(15)	46(8)
H(13C)	8770(50)	6190(30)	4082(15)	44(8)

6. DFT Calculations

DFT calculations presented in this paper were carried out with the molecular ADF program, version 2018.107.[12] Molecular geometries were optimized in gas phase using the PBE functional[13] in conjunction with the D3 version of Grimme's dispersion correction with Becke-Johnson damping.[14] The TZP basis set was used for all atoms during geometry optimization, frequency analysis and for single point calculations. Frequency calculations were performed to verify the nature of all stationary points and to calculate Gibbs free energies at 298.15 K.

Supplementary figure SF32: Structure (graphic created with: CylView20; Legault, C. Y., Université de Sherbrooke, 2020 (http://www.cylview.org) of representative CPA:

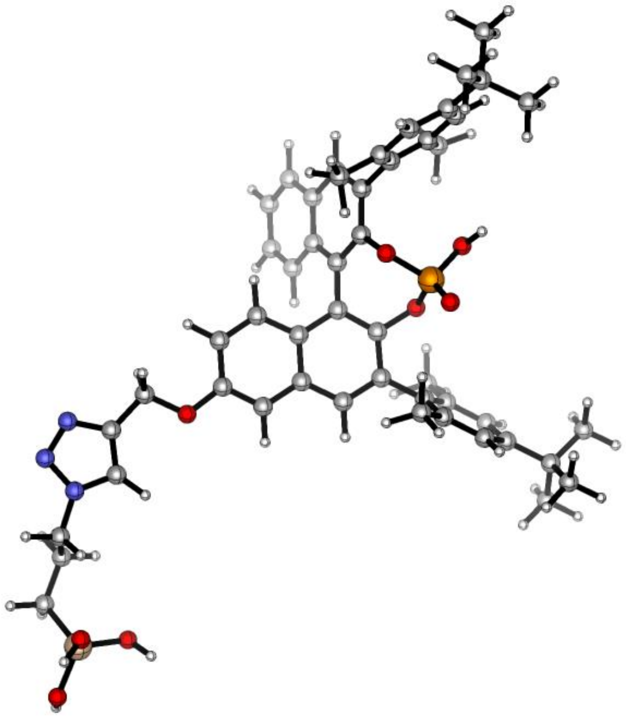

TBE in hartree = -27.675841, with zero imaginary frequencies.

Supplementary table S15: Coordinates:

O	-2.60142348	-2.70564883	-0.01275313
P	-1.19380785	-2.95285206	0.35540181
O	-0.73325756	-4.49317716	0.47599364
O	-0.84429939	-2.22426682	1.77000932
C	0.48924147	-2.21411508	2.20635817
C	1.39466200	-1.37513173	1.57165840

C	0.94562925	-0.52123329	0.44424796
C	1.15203380	0.89901163	0.43430022
C	1.66601783	1.60948125	1.54705708
C	1.85243582	2.97751810	1.51109591
C	1.52989941	3.70751276	0.33942623
O	1.70053032	5.06422452	0.21199806
C	2.20572503	5.77300895	1.35064711
C	2.29478474	7.21729532	0.98672034
C	1.78354601	7.89365191	-0.10380376
N	2.14717317	9.18752647	0.10656898
C	1.95839585	10.34194138	-0.76235164
C	3.07284245	10.46060583	-1.80630495
C	2.92211798	11.71078969	-2.68964811
Si	1.44638737	11.73461417	-3.84310603
O	1.56452093	10.31725085	-4.70182021
O	1.40817873	13.10410220	-4.79442866
O	-0.01603920	11.76316193	-3.04732981
N	2.85200205	9.30971722	1.25843553
N	2.93857163	8.11147480	1.79394490
C	0.99860322	3.05027460	-0.75725452
C	0.78465238	1.65352144	-0.73211882
C	0.19205145	0.98297177	-1.83389038
C	-0.09076341	-0.36835911	-1.79980357
C	-0.74615094	-1.05298528	-2.94969265
C	-2.12738765	-0.90278169	-3.17102779
C	-2.99448497	-0.11911050	-2.22019702
C	-2.70861910	-1.54443864	-4.27452723
C	-1.96512192	-2.33017365	-5.15894416
C	-2.59126848	-3.04167059	-6.36526522
C	-1.91078099	-2.54876463	-7.65925745
C	-4.09893414	-2.77547985	-6.48217805
C	-2.37861721	-4.56325174	-6.22206460
C	-0.59162905	-2.46390727	-4.90763860
C	0.03002703	-1.84599058	-3.82113664
C	1.50665262	-2.03287465	-3.58553506
C	0.28447729	-1.08892909	-0.63594483

O	0.00967994	-2.47221038	-0.62013541
C	2.76334153	-1.40859127	2.00458155
C	3.11250043	-2.21389315	3.14148336
C	4.46063944	-2.23418639	3.58980677
C	5.43768409	-1.52127240	2.92902579
C	5.10388748	-0.76758833	1.77949995
C	3.80092399	-0.71184814	1.32873851
C	2.11917800	-3.00241844	3.77307744
C	0.81815173	-3.04695308	3.30948709
C	-0.20270752	-3.95270978	3.90646523
C	-0.09690795	-5.34836454	3.70947284
C	1.05017028	-5.95182494	2.93719643
C	-1.09022307	-6.18033428	4.23363816
C	-2.18126949	-5.68396515	4.96238230
C	-3.24526715	-6.65152591	5.49615214
C	-3.89543491	-7.39078436	4.30758612
C	-4.35144754	-5.92868615	6.27794046
C	-2.57934539	-7.67861393	6.43536635
C	-2.24502332	-4.30232982	5.16264427
C	-1.27957010	-3.42734010	4.64527831
C	-1.41385388	-1.94560901	4.88081032
H	-1.48948241	-5.02352237	0.79834594
H	1.91423869	1.06482189	2.45739223
H	2.24400074	3.48320982	2.39248286
H	1.53051294	5.63381043	2.21420823
H	3.20436038	5.39872745	1.63548077
H	1.21756048	7.56930601	-0.96895431
H	0.96866070	10.26197503	-1.23201836
H	1.94923000	11.22298323	-0.10614750
H	4.03490564	10.48973815	-1.27228201
H	3.07871085	9.55648966	-2.43389123
H	2.88659701	12.62094586	-2.06708141
H	3.82273659	11.81429796	-3.31817546
H	0.78447019	10.09933657	-5.24221073
H	2.15655973	13.21936151	-5.40443691
H	-0.48945587	12.61049956	-3.11776854

H	0.72922397	3.62548637	-1.64481837
H	-0.07051403	1.55633891	-2.72575278
H	-2.58043518	0.87620538	-2.00725932
H	-4.00514346	0.00546347	-2.63058292
H	-3.07879911	-0.65297568	-1.26072436
H	-3.78136772	-1.41947490	-4.42414611
H	-2.05210206	-1.46529744	-7.78385506
H	-0.83061936	-2.74916465	-7.64980406
H	-2.34333664	-3.05721044	-8.53455092
H	-4.31356974	-1.70436360	-6.60927562
H	-4.49786933	-3.30377797	-7.36034640
H	-4.64441209	-3.13566984	-5.59797880
H	-1.31131110	-4.81920195	-6.17343101
H	-2.85708254	-4.93679177	-5.30525557
H	-2.81683219	-5.09184010	-7.08254700
H	0.02667550	-3.06925661	-5.57480194
H	1.97428685	-2.55723320	-4.42905625
H	2.01860398	-1.06880586	-3.44850310
H	1.68789354	-2.62533871	-2.67511777
H	4.70969159	-2.84049987	4.46378504
H	6.47089337	-1.54962547	3.27973664
H	5.88516429	-0.22821689	1.24089852
H	3.55752321	-0.13213244	0.43920395
H	2.39949992	-3.61415225	4.63357790
H	1.22455830	-5.41824566	1.99307520
H	1.98952354	-5.90845100	3.50907147
H	0.84815784	-7.00693905	2.70921764
H	-0.99924225	-7.25533526	4.05993822
H	-4.37968016	-6.67791330	3.62438723
H	-3.15331253	-7.96194646	3.73275061
H	-4.65966170	-8.09542107	4.66929110
H	-4.88609493	-5.20253802	5.64865185
H	-5.08587254	-6.66338173	6.63835193
H	-3.94985516	-5.39942544	7.15427682
H	-1.80207809	-8.25737411	5.91784651
H	-2.11079046	-7.17450771	7.29296894

H	-3.33053598	-8.38603870	6.81828653
H	-3.06772216	-3.87037872	5.73323015
H	-2.13465982	-1.74160772	5.68325593
H	-0.45012165	-1.49235179	5.15393579
H	-1.76596079	-1.43605511	3.97078708

References

1. Rueping, M., Antonchick, A. P. & Theissmann, T. Eine hoch enantioselektive Brønsted-Säure-katalysierte Kaskadenreaktion: organokatalytische Transferhydrierung von Chinolinen und deren Anwendung in der Synthese von Alkaloiden. *Angew. Chem.* **118,** 3765–3768; 10.1002/ange.200600191 (2006).
2. Pathak, K. *et al.* Enantioselective addition of diethylzinc to aldehydes using immobilized chiral BINOL–Ti complex on ordered mesoporous silicas. *Tetrahedron: Asymmetry* **17,** 1506–1513; 10.1016/j.tetasy.2006.05.017 (2006).
3. Sickert, M. *et al.* The Brønsted acid catalyzed, enantioselective vinylogous Mannich reaction. *Chemistry (Weinheim an der Bergstrasse, Germany)* **16,** 2806–2818; 10.1002/chem.200902537 (2010).
4. Romanov-Michailidis, F., Romanova-Michaelides, M., Pupier, M. & Alexakis, A. Enantioselective halogenative semi-pinacol rearrangement: extension of substrate scope and mechanistic investigations. *Chemistry (Weinheim an der Bergstrasse, Germany)* **21,** 5561–5583; 10.1002/chem.201406133 (2015).
5. Medina-Juárez, O., García-Sánchez, M. Á., Arellano-Sánchez, U., Kornhauser-Straus, I. & Rojas-González, F. Optimal Surface Amino-Functionalization Following Thermo-Alkaline Treatment of Nanostructured Silica Adsorbents for Enhanced CO_2 Adsorption. *Materials (Basel, Switzerland)* **9**; 10.3390/ma9110898 (2016).
6. Xi, L.-Y., Zhang, R.-Y., Zhang, L., Chen, S.-Y. & Yu, X.-Q. An efficient synthesis of quinolines via copper-catalyzed C-N cleavage. *Organic & biomolecular chemistry* **13,** 3924–3930; 10.1039/c5ob00075k (2015).
7. Zhang, Y., Zhao, R., Bao, R. L.-Y. & Shi, L. Highly Enantioselective SPINOL-Derived Phosphoric Acid Catalyzed Transfer Hydrogenation of Diverse C=N-Containing Heterocycles. *Eur. J. Org. Chem.* **2015,** 3344–3351; 10.1002/ejoc.201500330 (2015).
8. Maier, A. F. G. *et al.* Frustrated Lewis Pair Catalyzed Dehydrogenative Oxidation of Indolines and Other Heterocycles. *Angewandte Chemie (International ed. in English)* **55,** 12219–12223; 10.1002/anie.201606426 (2016).
9. Wang, Y., Ji, K., Lan, S. & Zhang, L. Rapid access to chroman-3-ones through gold-catalyzed oxidation of propargyl aryl ethers. *Angewandte Chemie (International ed. in English)* **51,** 1915–1918; 10.1002/anie.201107561 (2012).
10. Chen, Z.-P., Ye, Z.-S., Chen, M.-W. & Zhou, Y.-G. Enantioselective Synthesis of Endocyclic β-Amino Acids with Two Contiguous Stereocenters via Hydrogenation of 3-Alkoxycarbonyl-2-Substituted Quinolines. *Synthesis* **45,** 3239–3244; 10.1055/s-0033-1339849 (2013).
11. Sheldrick, G. M. A short history of SHELX. *Acta crystallographica. Section A, Foundations of crystallography* **64,** 112–122; 10.1107/S0108767307043930 (2008).
12. te Velde, G. *et al.* Chemistry with ADF. *J. Comput. Chem.* **22,** 931–967; 10.1002/jcc.1056 (2001).
13. Ernzerhof, M. & Scuseria, G. E. Assessment of the Perdew–Burke–Ernzerhof exchange-correlation functional. *The Journal of Chemical Physics* **110,** 5029–5036; 10.1063/1.478401 (1999).
14. Grimme, S. Accurate description of van der Waals complexes by density functional theory including empirical corrections. *J. Comput. Chem.* **25,** 1463–1473; 10.1002/jcc.20078 (2004).
15. Riente, P., Yadav, J. & Pericàs, M. A. A click strategy for the immobilization of MacMillan organocatalysts onto polymers and magnetic nanoparticles. *Org. Lett.* **14,** 3668–3671; 10.1021/ol301515d (2012).
